# Acetylene containing 2-(2-hydrazinyl)thiazole derivatives: design, synthesis, and *in vitro* and *in silico* evaluation of antimycobacterial activity against *Mycobacterium tuberculosis*†

**DOI:** 10.1039/d2ra00928e

**Published:** 2022-03-21

**Authors:** Lakshmi Haritha Bharathi Maganti, Deepthi Ramesh, Balaji Gowrivel Vijayakumar, Mohd Imran K. Khan, Arunkumar Dhayalan, Jayabal Kamalraja, Tharanikkarasu Kannan

**Affiliations:** Department of Chemistry, Pondicherry University Kalapet Puducherry-605014 India tharani.che@pondiuni.edu.in +91-413-265 6740 +91-413-265 4411; Department of Biotechnology, Pondicherry University Kalapet Puducherry-605014 India

## Abstract

*Mycobacterium tuberculosis* resistance to commercially available drugs is increasing day by day. To address this issue, various strategies were planned and are being implemented. However, there is a need for new drugs and rapid diagnostic methods. For this endeavour, in this paper, we present the synthesis of acetylene containing 2-(2-hydrazinyl) thiazole derivatives and *in vitro* evaluation against the H37Rv strain of *Mycobacterium tuberculosis*. Among the developed 26 acetylene containing 2-(2-hydrazinyl) thiazole derivatives, eight compounds inhibited the growth of *Mycobacterium tuberculosis* with MIC values ranging from 100 μg ml^−1^ to 50 μg ml^−1^. The parent acetylene containing thiosemicarbazones showed promising antimycobacterial activity by inhibiting up to 75% of the *Mycobacterium* at 50 μg ml^−1^. In addition, *in silico* studies were employed to understand the binding mode of all the novel acetylene-containing derivatives against the KasA protein of the *Mycobacterium*. Interestingly, the KasA protein interactions with the compounds were similar to the interactions of KasA protein with thiolactomycin and rifampicin. Cytotoxicity study results indicate that the compounds tested are non-toxic to human embryonic kidney cells.

## Abstract 2

In an attempt to find novel anti-mycobacterial agents, novel acetylene containing 2-(2-hydrazinyl)thiazole derivatives have been synthesized and evaluated against *Mycobacterium tuberculosis*.

## Graphical abstract

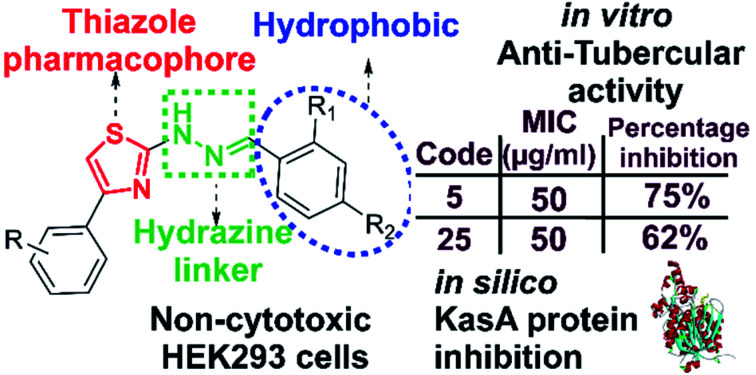

## 1. Introduction

*Tuberculosis* (Tb), caused by the pathogenic bacteria *Mycobacterium tuberculosis* (M. Tb) in the Mycobacteriaceae family is an airborne infectious disease and is ubiquitous, with universal widespread infection^1^ Tb has long been a crucial health risk owing to its contagious nature, chronic progression and complex immunological response, and due to this, there is a necessity for extended treatment.^2^ History indicates that Tb incidence increased in the 18th century and became pandemic during the industrial revolution. This sudden rise in Tb cases is attributed to poor living conditions and a rise in population.^3^ With the improvement in health conditions and by the introduction of the BCG (Bacillus Calmette–Guerin) vaccine in 1921 and with the development of antimicrobial drugs, there has been a decline in Tb cases. However, with the rise in viral infections that weaken immune systems, there has been an upsurge in Tb cases.^3^ The gravity of the scenario can be realized in the 2021 Global Tuberculosis report from the WHO which states that more than 5.8 million people were newly diagnosed with Tb and 1.3 million and 214 000 deaths were reported among HIV-negative and HIV-positive people respectively.^4^ Anti-Tb therapy aims to prevent hurdles and mortality by effectively curing the patient, avoid recurrences, and restrict the appearance of drug-resistant strains. Hence, the Tb treatment regime employs several medications.^5^ The current treatment method for drug-sensitive Tb incorporates isoniazid (INH), rifampicin (RIF) and pyrazinamide in the induction stage and ethambutol is also used here, if there is a case of any unidentified resistance to the other three medications. In the following consolidation stage, isoniazid and rifampicin are used for an extra four months.^6,7^ The treatment is successful in drug-sensitive Tb patients, but, for drug-resistant Tb like multi-drug-resistant Tb (MDR-Tb), extensively-drug-resistant Tb (XDR-Tb) and totally-drug-resistant Tb (TDR-Tb), the treatment shows only a 50% success rate.^8^ There are reports of drug toxicity like hepatotoxic influences, arthralgia, gastrointestinal disorders, and allergic responses associated with existing Tb drugs. The complications associated with the disease and treatment like carrying out the drug sensitivity tests and improper diagnosis, are other major hurdles associated with Tb treatment.^3^ Hence, finding a novel cure for the disease with shorter treatment duration and lower toxicity is crucial and essential.

**Fig. 1:**
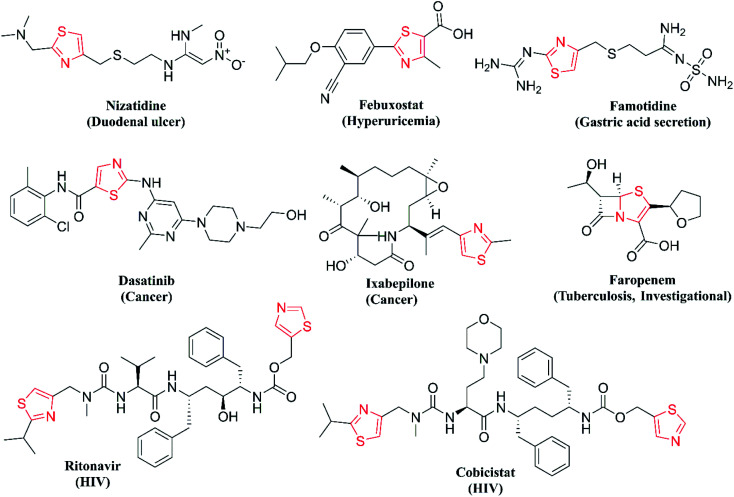
Thiazole pharmacophore-containing drugs.

One of the major classes of organic compounds showing promising anti-Tb activity is N-heterocycles, with isoniazid being the best example. Thiazole is important nitrogen and sulphur containing heterocycle showing a wide range of properties like anticancer,^9^ anti-HIV,^10^ anti-fungal,^11^ antibacterial,^11^ anti-parasitic,^12^ anti-inflammatory^13^ and antitubercular activity.^14^ Thiazoles are seen in a wide diversity of natural products, marine algae and alkaloids. Thiazole derivatives isolated from *Mechercharimyces asporophorigenens* are showing anti-lung cancer activity at a lower concentration of 12 nM and another derivative isolated from *Oscillatoria* sp. inhibits the growth of *Plasmodium falciparum* at 8.2 μM.^15^ On the other hand, the 2-aminothiazole moiety is structurally similar to thiolactomycin and is one of the well-studied thiazole compounds as the anti-Tb agent. Thiolactomycin disturbs mycolic acid biosynthesis for bacterial cell walls by the inhibition of the β-Ketoacyl-ACP Synthase (KasA) protein.^16^ Thiazole derivatives can also coordinate metal ions and are important pharmacophores for the synthesis of novel biologically relevant entities. Hence, in the database published by the Food and Drug Administration (FDA) in 2015, thiazole core ranks 6th among the frequent N-heterocycles in approved drugs.^17^ The structures of important drugs with thiazole core are given in Fig. 1.

**Fig. 2:**
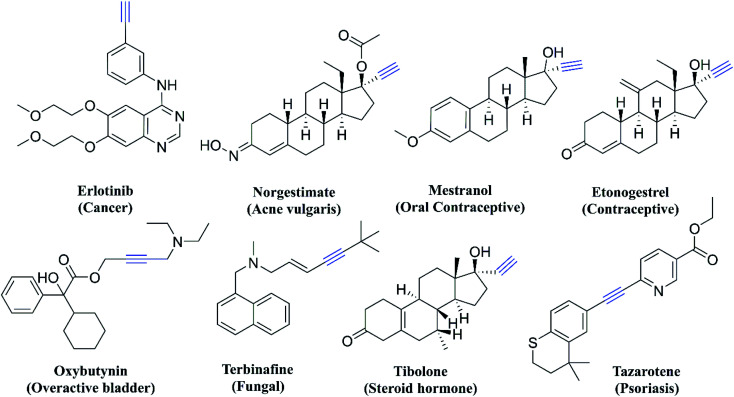
Acetylene pharmacophore-containing drugs.

Thiazole derivatives are usually synthesized from yet another important pharmacophore, thiosemicarbazone. Thiosemicarbazones also have a wide range of bioactivities which is often related to their ability to coordinate metal ions. They can chelate metal atoms by coordinating through the hydrazine nitrogen atom and sulphur.^18^ Thiosemicarbazones are also known to show antitubercular,^19^ antimalarial,^20^ antifungal,^21^ antiviral,^22^ anti-trypanosomal,^23^ and antileishmanial activities.^24^ Even though there are different modifications of thiosemicarbazone and thiazole scaffolds, so far there has been no report of incorporation of an acetylene moiety along with these two pharmacophores for testing as an anti-Tb agent. The acetylene group is important in medicinal chemistry as there are many drugs either approved or in the clinical/preclinical stage and some of the examples are given in Fig. 2. The acetylene group containing derivatives show antimalarial,^25^ antitubercular,^25^ kinase inhibition,^26^ anticancer,^27^ and antimicrobial^27^ activities. Acetylenic metabolites from nature such as *Hydnum repandum*, *Polyporus biformis*, *Drosophila subatrata*, *etc.*, also show anticancer, antibacterial and antimicrobial properties.^28^

These pharmacophores mentioned above offer many avenues of drug development especially for treating Tb. These pharmacophores can enhance the bioactivity either through binding to receptor binding pocket or by irreversibly inhibiting the target protein or by directing pharmacophores to bind in a favourable geometry or by changing the drug pharmacodynamic profile.^29^ There are two important approaches in the discovery of anti-Tb drugs such as the synthesis of analogous pre-existing drugs and the discovery of novel derivatives.^30^ Going along this strategy to synthesize novel antitubercular agents, molecular hybridisation in drug design is used in the present investigation. This attempt can develop compounds with modified selectivity, lesser aftereffects, higher solubility and bioavailability.^31^ We aim to combine and enhance the desirable traits of the aforementioned pharmacophores. Hence, in our constant attempt to synthesize novel anti-Tb agents,^32,33^ here, we have designed and synthesized a hybrid molecule containing thiazole moiety and (ethynyloxy)benzene connected *via* hydrazine linker. There are very few reports of thiazole drugs having acetylene moiety^34–36^ as given in Fig. S1,† but nothing has been reported with anti-Tb activity so far. Interestingly, there are no reports on acetylene containing 2-(2-hydrazinyl)thiazole derivatives and hence, these derivatives were designed considering their physicochemical properties and were synthesized thereafter. The *in vitro* antitubercular activity of the acetylene containing 2-(2-hydrazinyl)thiazole derivatives is analysed against the H37Rv strain of M. Tb, along with the *in silico* analysis against KasA protein and the results are discussed in this paper.

## 2. Experimental section

### 2.1. Materials and methods

The aldehydes were purchased from Sigma-Aldrich, USA and Avra Synthesis, Hyderabad, India. Propargyl bromide, potassium carbonate, dimethylformamide (DMF), thiosemicarbazide, ethanol and glacial acetic acid were purchased from Avra Synthesis, Hyderabad, India. Phenacyl bromide derivatives were obtained from Avra Synthesis, Hyderabad, India, Spectrochem Pvt. Ltd, Mumbai, India and Sigma-Aldrich, USA. All the chemicals and solvents received were used as such without purifying further. Dulbecco's Modified Eagle Medium (DMEM) (Himedia, Cat. No. AL111), fetal bovine serum (FBS) (Himedia, Cat No. RM9955) and l-glutamine–penicillin–streptomycin solution (Himedia, Cat. No. A007) were used as received. Human Embryonic Kidney 293 (HEK293) cell lines were purchased from the National Centre for Cell Sciences (NCCS), Pune, India. The structures of the developed compounds were confirmed by using ^1^H (400 MHz) and ^13^C (101 MHz) Fourier Transform Nuclear Magnetic Resonance (NMR) spectrometer (Make: Bruker, Model: Avance-II) with tetramethylsilane as an internal standard. The mass of the compounds was evaluated using High Resolution Mass Spectrometry (Brand: Agilent Technologies, Model: 6530B Accurate Mass QTOF-LC/MS). Then, the functional groups of the prepared compounds were identified using Fourier transform infrared (FT-IR) spectroscopy (Thermo Nicolet 6700). The pharmacokinetic properties of the compounds were analysed using online servers such as Molinspiration (https://www.molinspiration.com/cgi-bin/properties),^37^ pkCSM^38^ and SwissADME.^39^

### 2.2. Synthesis of acetylene containing aldehydes (1–3)

The hydroxy aldehydes (1 mmol) and potassium carbonate (1.5 mmol) were taken in a round-bottomed (RB) flask along with DMF as a solvent and were stirred at room temperature to which 1.1 mmol of propargyl bromide was added dropwise. The reaction was monitored by thin-layer chromatography (TLC) using an ethyl acetate/hexane solvent mixture. At the end of the reaction, crushed ice was added and stirred to obtain the precipitate. The precipitate was filtered and dried at reduced pressure. Yield: 85–90%.

### 2.3. Synthesis of acetylene containing thiosemicarbazones (4–6)

The acetylene containing aldehydes (1 mmol), thiosemicarbazide (1 mmol), ethanol (5 ml), and glacial acetic acid (5–10 drops) were taken in an RB flask and stirred at reflux conditions. TLC was used to monitor the reaction status and after the completion, the reaction mixture was cooled to room temperature. The resulting precipitate was filtered and dried to obtain the crude product which was further recrystallized in ethanol. Yield: 75–80%.

### 2.4. Synthesis of acetylene containing 2-(2-hydrazinyl)thiazoles (7–32)

Acetylene containing thiosemicarbazones thus obtained (1 mmol) and appropriate phenacyl bromide derivatives (1 mmol) were dissolved in ethanol and continued stirring at reflux conditions. Completion of the reaction was confirmed using TLC and at the end of the reaction, the reaction mixture was poured onto crushed ice. The resulting crude product was filtered, dried and recrystallized using ethanol. Yield: 70–75%.

### 2.5. *In vitro* anti-tubercular activity

The *in vitro* anti-tubercular studies were performed using standard Luciferase reporter mycobacteriophages (LRP) assay as given in ESI.† Here, compounds with 50% RLU reduction and above when compared with control were considered as active against M. Tb. The Tb drug RIF has been taken as a reference compound for the current investigation.

### 2.6. Molecular docking

Proteins structure (PDB: 2WGD)^40^ was obtained from RCSB and prepared using MGL tools v1.5.6,^41^ removing irrelevant co-crystallized ligands or solvent. Polar hydrogens alone were considered and charges assigned (Gaussian and Gasteiger) to a macromolecule. Energy minimized ligands were prepared using PerkinElmer Chem3D (v15.0) Blind docking was carried out on Autodock Vina (v.1.1.2)^42^ at exhaustiveness set to 8 with PyRx (v0.8)^43^ to assist. Binding affinities were noted for RMSD < 2 and post-docking analysis for amino acid interactions was done *via* Biovia Discovery Studio Visualizer (v19.1) (https://discover.3ds.com/discovery-studio-visualizer-download).^44^

### 2.7. Cytotoxicity studies

HEK293 cells were grown in DMEM media containing 10% FBS and l-glutamine–penicillin–streptomycin solution at 37 °C in 5% CO_2_ condition. To perform MTT assay, 96 well tissue culture plate was seeded with 103 number of cells. After 16 hours of seeding, cells were successfully attached to the culture plate and were treated with the active chemical compounds dissolved in DMSO at different concentrations as follows: 25 μg ml^−1^, 50 μg ml^−1^, 75 μg ml^−1^ and 100 μg ml^−1^. The cells were also treated with the DMSO to exclude the solvent-induced toxicity and used as an internal control. After 48 hours post active chemical compounds treatment, the MTT assay was performed as described previously.^45^ MG-132, a potent *proteasomal* inhibitor and an inducer of apoptosis^46,47^ was used as a positive control in the assay. Wells with media lacking any cells were as used as a negative control in the assay.

### 2.8. Statistical analysis

Quantitative data are performed in triplicates and are presented as mean ± standard deviation (SD). The one-way analysis of variance (ANOVA) was used to analyse the difference between the specific means and the data was given in Tables S7 and S8.† The criterion for statistical significance was *P* < 0.05.

## 3. Results and discussion

**Fig. 3:**
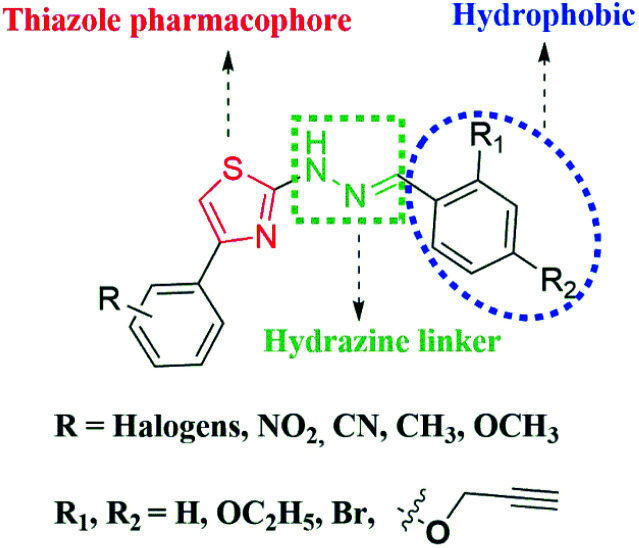
Design of the lead structure for the present investigation.

In our continuous endeavour to find out novel compounds,^32,33^ in the present investigation, we intentionally introduced acetylenic moiety to study its effect on anti-Tb activity and pharmacokinetic properties. Hence, a library of novel acetylene containing 2-(2-hydrazinyl)thiazoles was designed, synthesized and screened against M. Tb H37Rv strain. To figure out the plausible binding mode of the acetylene containing compounds, *in silico* studies of the derivatives were carried out against KasA protein of M. Tb. The structural features of the acetylene containing 2-(2-hydrazinyl)thiazole derivatives were taken into account while designing novel compounds and the lead structure can be split into three parts as given in Fig. 3.

### 3.1. Design of acetylene containing 2-(2-hydrazinyl)thiazole derivatives

The drug molecule once administered can interact with different biopolymers in the extracellular fluid, cells and the cell membrane. The extent and type of interaction will depend on the number of reactive functional groups and the polarity of the drug.^48^ The drug-like character of the compounds such as a molecular weight of 350–400 with necessary solubility to dissolve in an aqueous medium and lipophilicity to pass through lipid membranes should be evaluated for drug effectiveness.^49^ Hence, the physicochemical properties and the drug pharmacokinetics including their absorption, distribution, metabolism and excretion (ADME) properties were considered for effective designing of the thiazole derivatives. The drug-like molecular (DLM) properties of the acetylene containing derivatives were analysed using Lipinski^50^ and Veber rules.^51^ The acetylenic moiety was retained in all thiazole derivatives and various substitutions were introduced in the respective aromatic rings to understand their influence on M. Tb inhibition. The thiazole derivatives, hence, designed along with their parent acetylene containing aldehyde derivatives and acetylene containing thiosemicarbazones are given in Table S1.† The DLM properties were obtained using an online Molinspiration server^37^ and the results are given in Table S2.† The molecular weight of all the compounds is in the allowed range for drug efficiency. Acetylene containing aldehydes, thiosemicarbazones and acetylene containing 2-(2-hydrazinyl)thiazoles show molecular weight in the range of 160.17–204.33 g mol^−1^, 233.29–312.19 g mol^−1^ and 333.41–491.20 g mol^−1^ respectively. The log *P* values of the compounds vary from 1.90–6.20 and these values are higher than the acceptable value of 5 for seven thiazoles with acetylenic moiety. Since approved anti-Tb drugs have higher values of log *P* for increased cell permeability, the violation of Rule of 5 can be justified.^52^ Except for the log *P* value, other parameters like the number of hydrogen bond donors and acceptors are within the acceptable limits. The parameters for satisfying the Veber rule were evaluated. The topological polar surface area (TPSA) values fall in the range of 46.52 to 101.58 and the sum of rotatable bonds are within the 6–8 range for the thiazoles. These parameters indicate promising bioavailability of the designed thiazole derivatives.

### 3.2. ADME properties of acetylene containing 2-(2-hydrazinyl)thiazole derivatives

The ADME properties of acetylene containing 2-(2-hydrazinyl)thiazole derivatives listed in Table S1† are given in Table S3.† The water solubility of compounds was indicated by log *S* and these values for acetylene containing 2-(2-hydrazinyl)thiazole derivatives range from −6.49 to −4.78 whereas the parent acetylene containing thiosemicarbazones have higher water solubility with log *S* values from −2.41 to −2.85. The presence of hydrophobic acetylene^53^ may decrease the water solubility of thiazole derivatives while the thiosemicarbazones are showing better solubility because of coordination through the sulphur and nitrogen atom of the hydrazine group. The percentage absorption of acetylene containing 2-(2-hydrazinyl)thiazole derivatives and parent compounds were evaluated by considering the Caco-2 permeability. The compounds should indicate *P*_app_ value > 8 × 10^−6^ cm s^−1^ for moderate to higher cell permeability during oral absorption. Interestingly, except for one acetylene containing 2-(2-hydrazinyl)thiazole derivative, 12, all the other compounds showed cell permeability greater than the approved Tb drugs INH and RIF. As the drug absorption occurs in the intestine, the percentage of absorption for each derivative was calculated. All the compounds showed moderate to higher intestinal absorption ranging from 73% to 99%. Surprisingly, the acetylene containing aldehyde derivatives showed higher intestinal absorption than the Tb drug INH. All the acetylene containing 2-(2-hydrazinyl)thiazole derivatives also showed higher absorption ranging from 89% to 94% which are higher than Tb drug, RIF.

The bodily distribution of drug after absorption should be monitored and this is predicted using a volume of distribution (VDss), fraction unbound and blood–brain barrier (BBB) permeability. A higher VDss value given by log VDss > 0.45 shows that the drug is highly dispersed in the tissues. However, except for compound 19, all other compounds have lower VDss values indicating drug distribution in plasma. The values of fraction unbound also indicate that compounds are less attached to blood proteins and will diffuse freely. The BBB permeability is an important factor which determines the capability of a drug to cross over the brain barrier and is useful in treating *tuberculosis meningitis*.^54^ However, 25 acetylene containing 2-(2-hydrazinyl)thiazole derivatives and all thiosemicarbazones were BBB permeable. On the contrary, all the acetylene containing aldehydes (1, 2 and 3) and compound 7 could penetrate BBB. The metabolism and total renal clearance of the compounds were also analysed. Drug clearance rate depends on the bioavailability of the compound and is indicative of dosage rate also.^51^ All the 2-(2-hydrazinyl)thiazole derivatives have renal clearance in the range of 0.021 log ml min^−1^ kg^−1^ to 0.376 log ml min^−1^ kg^−1^ and are better than the Tb drug, RIF. All the 2-(2-hydrazinyl)thiazole derivatives have good ADME properties to be considered as lead candidates and hence can be synthesized.

### 3.3. Synthesis and characterization of acetylene containing 2-(2-hydrazinyl)thiazoles

**Scheme 1:**
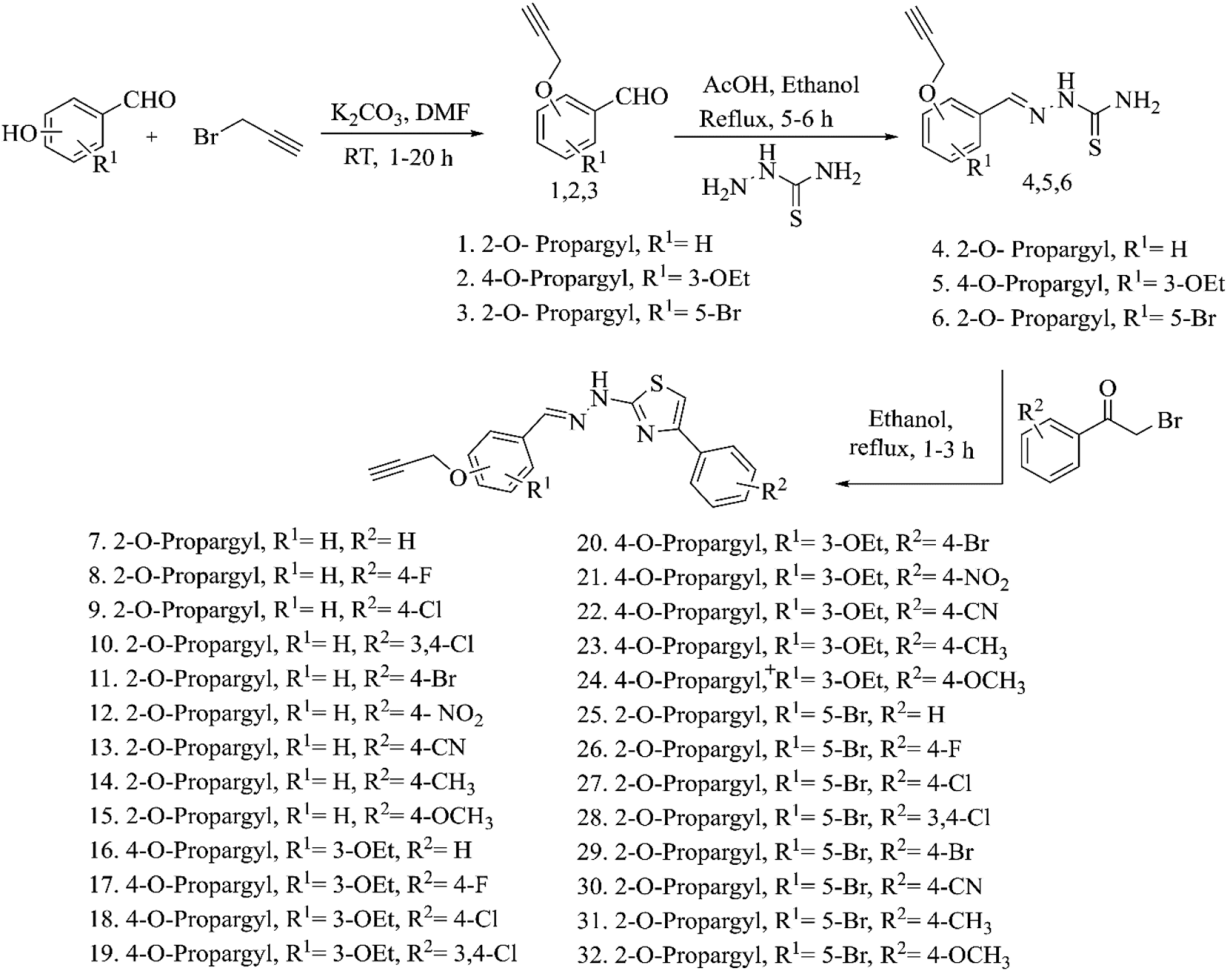
Synthesis of acetylene containing 2-(2-hydrazinyl)thiazole derivatives.

The acetylene containing compounds showed drug-like properties and hence were synthesized based on existing literature procedures^32,55^ as given in Scheme 1. The compounds 1–32 were synthesized by reacting hydroxyl group-containing aldehydes with propargyl bromide (1–3) followed by thiosemicarbazide (4–6) and phenacyl bromides (7–32). The structure of the propargyl group-containing benzaldehyde compounds (1–3) was confirmed by the presence of a characteristic aldehyde peak at 9.85–10.35 ppm in ^1^H NMR spectra and 187–192 ppm in ^13^C NMR spectra. In FT-IR spectra of these compounds, aldehyde C–H stretching vibrations appears at 2876 cm^−1^ and aldehyde C=O stretching vibrations appears at 1680 cm^−1^. When the aldehydes are getting converted into corresponding thiosemicarbazones, the characteristic aldehyde peaks disappeared from all spectra and the presence of thiosemicarbazone (4–6) compounds is confirmed with the existence of –HC=N– at 8.14–8.40 ppm in ^1^H NMR spectra, 1530 cm^−1^ in FT-IR spectra and –NH_2_ at 8 ppm in ^1^H NMR spectra. In compounds 7–32, the peaks corresponding to NH_2_ disappear due to the formation of a thiazole ring which is confirmed by the presence of a single –NH peak at 7 ppm in ^1^H NMR spectra and 3400 cm^−1^ in FT-IR spectra. The main acetylene linker present in all the compounds (1–32) was confirmed by the presence of acetylene proton at 3.55–3.65 ppm in ^1^H NMR spectra and acetylene carbons at 78 ppm and 79 ppm in ^13^C NMR spectra. Additionally, the –CH_2_ protons also appear at 4.80–5 ppm in ^1^H NMR spectra and –CH_2_ carbons at 55–57 ppm in ^13^C NMR spectra. In FT-IR spectra, the presence of stretching frequencies at 2117.6 cm^−1^ (alkyne –C≡C– stretch), 3236 cm^−1^ (alkyne C–H stretch) and 1228 cm^−1^ (C–O stretch) further establishes the existence of acetylene moiety. The molecular weight of the synthesized compounds was confirmed from the mass spectroscopy results and the experimental mass values match with the theoretical mass values. Spectra and spectral data of all the synthesized acetylene containing compounds (1 to 32) are provided in the ESI.†

### 3.4. Single-crystal X-ray diffraction analysis

**Fig. 4:**
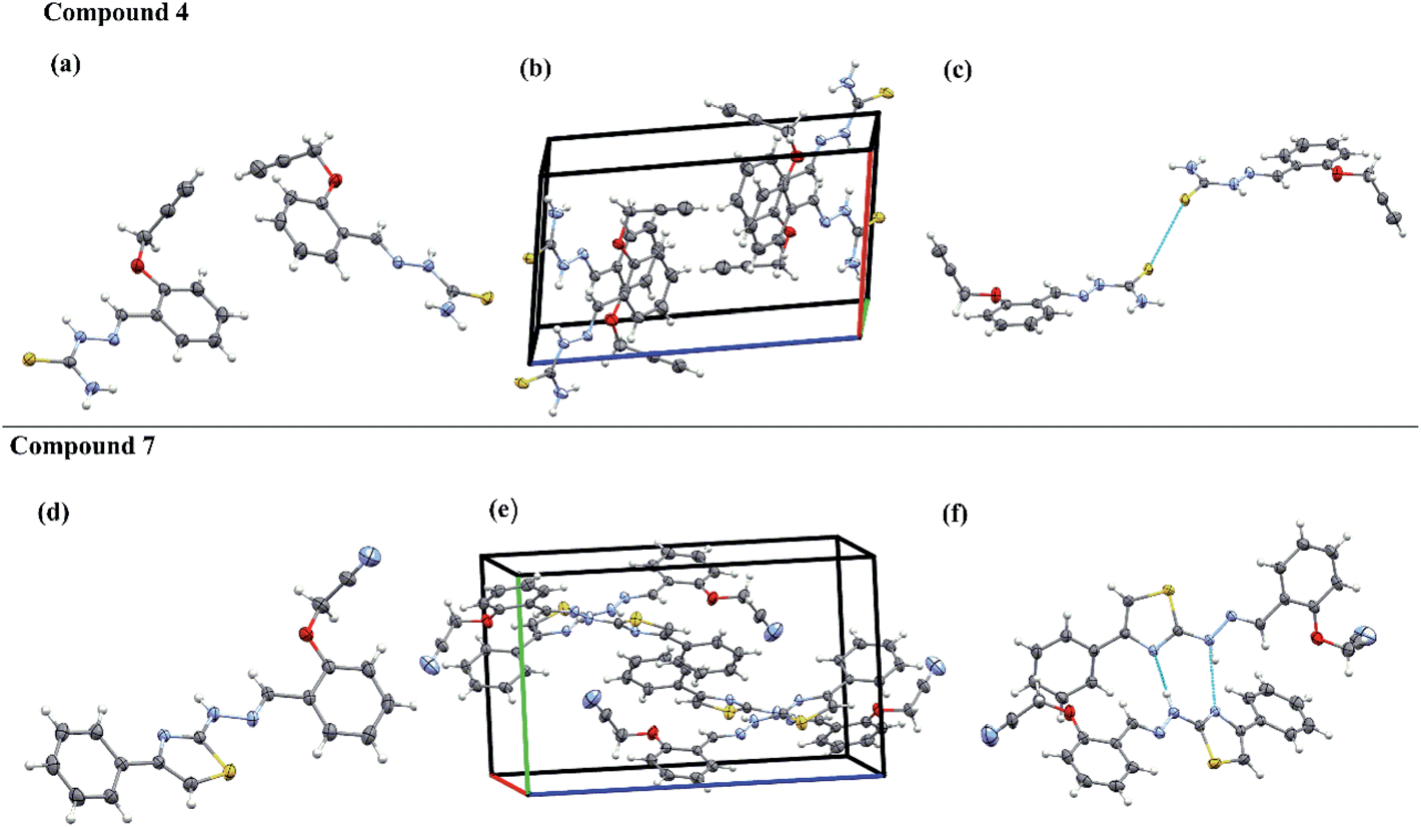
Single-crystal XRD data. The ORTEP diagrams (a and d), packing diagrams (b and e) and hydrogen bond interaction diagrams (c and f) of compounds 4 and 7.

The aromatic rings and thiazole rings attached to C=N can have either *E* or *Z* configuration and an insight into their exact configuration is crucial for drug design. The spatial arrangement of the compounds helps in designing the lead compounds with enhanced therapeutic properties. The acetylene containing thiosemicarbazone have one phenyl ring system and acetylene containing 2-(2-hydrazinyl)thiazole derivatives have one thiazole and two phenyl ring systems respectively. Hence, in an attempt to understand the structural diversity, two representative single crystals of compounds, one from acetylene containing thiosemicarbazone (4) and another from acetylene containing 2-(2-hydrazinyl)thiazole derivatives (7) were crystallized in DMSO and the solved XRD patters are given in Fig. 4. The XRD data indicate that both the compounds are non-planar with the acetylene carbons positioned out of the plane at an angle of 111°. The intermolecular S-bonding between different crystals of 4 are visible in the packing diagram. The S–S bonding is between C=S moieties of thiosemicarbazone crystals at a distance of 1.6764 Å and 1.6821 Å respectively. There is no visible H-bonding between –NH groups of different thiosemicarbazones or –NH groups with any other functional groups. The presence of S bonding stabilises the crystal structure as seen from the packing diagram and the crystals are packed in a single unit cell in head to head overlapped fashion.

In the case of the acetylene containing 2-(2-hydrazinyl)thiazole derivatives (7), the crystals are having H bonding rather than S-bonding as in the case of its predecessor. Here, the hydrazine NH is above the plane and the thiazole S atom is positioned below the plane, indicating that the compound is not planar. The H-bonding is between –N of thiazole and NH of hydrogen and each crystal is having two H-bonding between different crystals of the same compound. Compound 7 shows a displaced head to tail overlap in a unit cell. The crystal parameters of both the compounds are given in Table S4.†

### 3.5. *In vitro* antitubercular activity of acetylene containing 2-(2-hydrazinyl)thiazoles

The acetylene containing 2-(2-hydrazinyl)thiazole derivatives were analysed for *in vitro* antimycobacterial activity against the H37Rv strain of M. Tb, after considering their DLM properties. The minimum inhibitory concentration (MIC) of thiazoles giving 50% inhibition of M. Tb was considered to have antitubercular activity. The reference compound for the LRP assay was rifampicin and the results of *in vitro* anti-tubercular analysis are given in Table S2.† In the case of the acetylene containing aldehyde derivatives, all the compounds (1–3) were inactive against M. Tb. The aldehydes were precursors for the synthesis of bioactive thiazoles and were not modified further to enhance the Tb activity. The acetylene containing thiosemicarbazones, however, showed interesting activity against M. Tb. Compound 4 showed no inhibitory potential at 50 μg ml^−1^. Compound 5 having an ethoxy substitution at *ortho* to acetylenic moiety showed high inhibitory potential with 77.75 ± 2.33% inhibition at 100 μg ml^−1^. Even with a decrease in concentration to 50 μg ml^−1^, it has retained its bioactivity with 74.58 ± 2.23% inhibition. At 50 μg ml^−1^, when the ethoxy group was substituted by 4-Br (6), there was a sudden decrease in the inhibitory potential. The percentage inhibition of 6 decreased from 56.86 ± 1.70% to 51.21 ± 1.53% with a decrease in concentration from 100 to 50 μg ml^−1^. The presence of the ethoxy group has a greater influence on bioactivity and the inhibitory potential for the thiosemicarbazones decreases as 2-OC_2_H_5_ > 4-Br > phenyl.

In acetylene containing 2-(2-hydrazinyl)thiazole derivatives, 7 to 15, (ethynyloxy)benzene ring is unsubstituted and change of functional groups is only at phenyl ring linked to thiazole. In compound 7, both the rings are unsubstituted and at 50 μg ml^−1^, 7 shows inhibition of 51.66 ± 1.54%. To understand the influence of substitutions in the phenyl ring, different acetylene containing 2-(2-hydrazinyl)thiazole derivatives were synthesized and analysed. The effect of 4-F substitution is visible with a drop in inhibition to 38.01 ± 1.14%, making compound 8 inactive against M. Tb. When the F is substituted with Cl moiety (9), the per cent inhibition increases to 50.81 ± 1.52%. The log *P* value of 8 however increased after the change in halo-substitution from 2.29 to 4.80. However, in the case of 3,4-Cl substitution as in compound 10, the inhibitory potential increases to 52.00 ± 1.56%. The increase in inhibition is related to an increase in the log *P* value of 5.41. There was a slight drop in inhibition to 49.13 ± 1.47% when substituted with a 4-Br group (11) making the compound inactive. In the case of substitution with a highly electronegative nitro group (12), the activity went down to 29.06 ± 0.87% and hence making the compound inactive. Surprisingly, 4-CN substitution in compound 13 showed promising inhibition of 54.33 ± 1.62% and a lower log *P* value of 3.88. However, compound 14 with 4-CH_3_ substitution showed only 38.18 ± 1.14% inhibition, unlike its bioisostere 4-Cl in compound 9. In a similar pattern, electron-donating 4-OCH_3_ in compound 15 only showed 10.26 ± 0.30% inhibition, the lowest among the discussed series of compounds. Here, the activity can be correlated with log *P* values. Except for compound 10, for the other three active compounds, the activity was directly proportional to the decreasing of log *P* values. The activity order of the acetylene containing 2-(2-hydrazinyl)thiazole derivatives discussed here are as follows: 4-CN > 3,4-Cl > phenyl > 4-Cl. Also, it is interesting to note that electronegative substituents in the phenyl ring increase the antitubercular activity except for compound 7.

In acetylene containing 2-(2-hydrazinyl)thiazole derivatives, 16 to 24, (ethynyloxy)benzene ring has ethoxy substitution and changes are at the functional groups of phenyl ring linked to thiazole. In compound 16, the phenyl ring is unsubstituted and the compound shows no inhibition at both 50 and 100 μg ml^−1^. The presence of 4-F substitution does not contribute towards activity and compound 17 shows only 2.06 ± 0.06% inhibition at 50 μg ml^−1^. In contrast to compound 9, the presence of 4-Cl does not enhance the activity for 18 and the compound is inactive at both 100 and 50 μg ml^−1^. The same pattern is observed for other halo substituted derivatives 3,4-Cl (19) and 4-Br (20). The nitro substitution in compound 21 slightly induces inhibition of 50.85 ± 1.52% at 100 μg ml^−1^, but, the compound is inactive at 50 μg ml^−1^. As expected from the previous result, the 4-CN group in compound 22 augments the activity at both 100 and 50 μg ml^−1^ with a % inhibition of 57.18 ± 1.71% and 50.86 ± 1.52% respectively. The electron-donating 4-CH_3_ (23) and 4-OCH_3_ (24) groups show the same pattern as before with no inhibition. The electron-withdrawing 4-CN and 4-NO_2_ are only active in this category and activity are proportional to log *P* values for these compounds. The compounds, 21 and 22 show log *P* values as 4.10 and 3.89 respectively and hence, activity is in the order of 4-CN > 4-NO_2_.

**Fig. 5:**
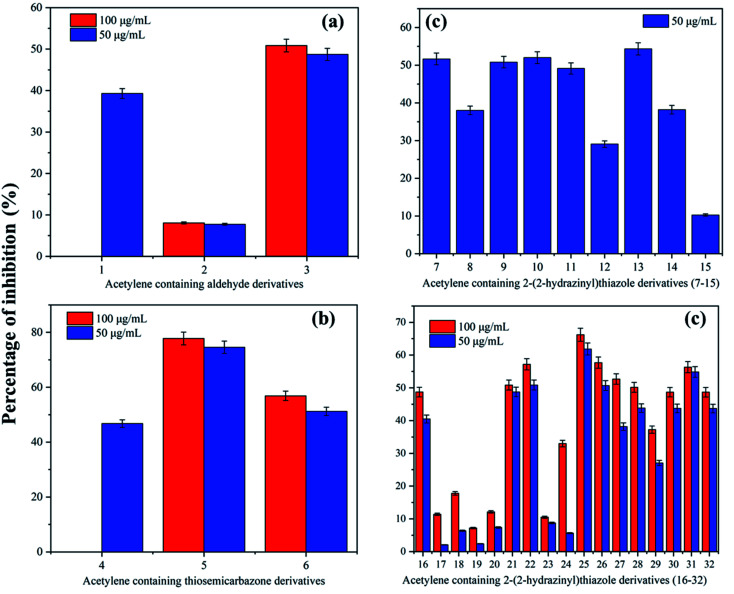
Correlation diagram of percentage inhibition (mean ± SD) with (a) acetylene containing aldehydes, (b) thiosemicarbazones and (c) acetylene containing 2-(2-hydrazinyl)thiazole derivatives.

In acetylene containing 2-(2-hydrazinyl)thiazole derivatives, 25 to 32, (ethynyloxy)benzene ring has 4-bromo substitution and changes are at the functional groups attached to the phenyl ring linked to thiazole. The compound 25 with no substitution in phenyl ring and lowest log *P* value in this set, shows promising anti-Tb activity with MIC of 50 μg ml^−1^. The striking factor is that the inhibition remains only slightly varied from 66% to 61% with a decrease in concentration from 100 μg ml^−1^ to 50 μg ml^−1^. Interestingly, 4-F substitution (26) shows inhibition at 100 μg ml^−1^ and 50 μg ml^−1^ with 66% and 61% of inhibition respectively. Here also, the change in concentration does not affect the activity much. However, other halo substitutions were inactive at 50 μg ml^−1^ when compared to 4-F. In the case of 4-Cl and 3,4-Cl substitutions (27 and 28), the compounds show 50% inhibition at 100 μg ml^−1^, but as the concentration decreases to 50 μg ml^−1^, the activity also decreases. On the contrary, 4-Br substitution in compound 29 shows no inhibition even at 100 μg ml^−1^. If we consider the activity of halogen derivatives in this set, it follows the order: 4-F > 4-Cl > 3,4-Cl > 4-Br. The activity is directly proportional to the size of halogens. Moving on to the highly electronegative 4-CN group, surprisingly, the previously active 4-CN group (13), showed only 48.69 ± 1.46% inhibition by compound 30 even at 100 μg ml^−1^. The 4-CH_3_ group in compound 31 however showed a promising inhibitory potential of 54% even at 50 μg ml^−1^. This is unexpected as CH_3_ moiety remained inactive in other compounds. The effect of 4-OCH_3_ substitution did not contribute towards anti-Tb activity and compound 32 was inactive at both 100 and 50 μg ml^−1^. The activity of compounds in this set follows the order: phenyl > 4-F > 4-CH_3_ > 4-Cl > 3,4-Cl. The activity as described previously is directly correlated to lower log *P* values. The effect of compounds on inhibition of M. Tb is given in Fig. 5.

The summary of active compounds is given in Table S5.† In the first case, compound 4 is inactive as the acetylenic moiety is hydrophobic and is not contributing towards effective H-bonding with thiosemicarbazone. When 4 is converted to corresponding thiazoles, only electron-withdrawing derivatives are active. Here, two chlorine derivatives (9, 10) are showing >50% inhibition at 50 μg ml^−1^. Chlorine is a moderately active halogen bond acceptor and increases the interaction of compounds with hydrophobic pockets of the target protein.^56^ Compound 5 is showing the highest inhibition rate among all the synthesized derivatives. The presence of the ethoxy group near thiosemicarbazone increases the non-covalent interaction and hence increases the activity. The interaction is decreased in corresponding thiazole moieties (16–23) and only electron-withdrawing derivatives (22, 21) are active at 50 μg ml^−1^. In the case of compound 6, even though electron-withdrawing Br substituent is present, the bulky acetylene moiety ortho to thiosemicarbazone decreases the activity. In the corresponding thiazole derivatives, slightly electron-withdrawing halogens are active along with methyl substituent. In a nutshell, the presence of electron-withdrawing substituents and halogens are enhancing the activity of thiazole derivatives with inhibitory potential ranging from 50–60% at both concentrations. The halogens can improve oral absorption and increases blood–brain barrier permeability which is important in tubercular meningitis.^57,58^

From the above discussions, it is well understood that acetylene containing 2-(2-hydrazinyl)thiazole derivatives having electron-withdrawing substituents are inhibiting M. Tb in LRP assay and can be further modified for enhancing the activity. The parent acetylene containing thiosemicarbazones 5 with 74.58 ± 2.23% of inhibition is the top active compound followed by thiazole derivative 25 showing 61.86 ± 1.85% of inhibition. Out of 26 acetylene containing 2-(2-hydrazinyl)thiazole derivatives prepared, 8 compounds show 50–60% inhibition of *Mycobacterium* at 50 μg ml^−1^. Two acetylene containing thiosemicarbazones are also active with 74.58 ± 2.23% (5) and 51.21 ± 1.53% (6) inhibition at 50 μg ml^−1^. The further modification of the synthesized novel acetylene containing 2-(2-hydrazinyl)thiazole derivatives can contribute to the enhancement in the inhibitory potential.

### 3.6. Docking studies

**Fig. 6:**
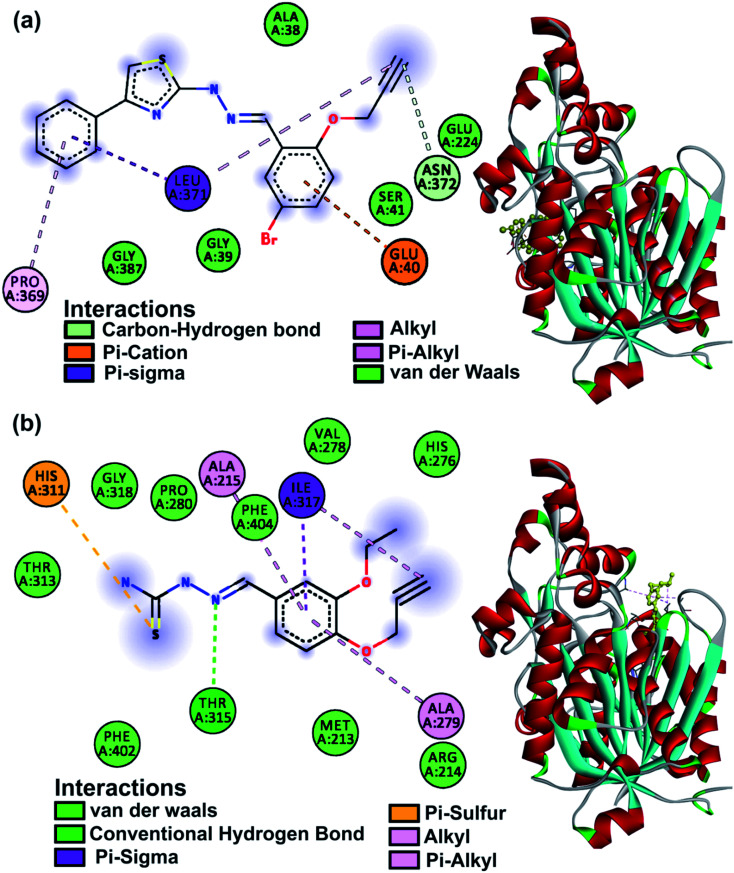
KasA protein 2D amino acid interactions and 3D binding mode of (a) compound 25 and (b) compound 5.

The KasA protein (PDB: 2WGD) was selected as the parent compounds are known to follow the KasA FAS-II inhibitory pathway.^40^ Isoniazid and rifampicin were used as a reference and the results of the study are listed in Table S6.† Compounds ranged in their binding affinity from −7.7 to −5.2 kcal mol^−1^. The post docking analysis for amino acid interactions was expected to explain the activity or lack thereof. The main catalytic triad on KasA includes His345, His311 and Cys171 amino acids which are part of the acyl channel, the gatekeeper to which is residue Phe404.^59^ Interactions at these sites are expected to contribute to the inhibitory activity. Compounds 1, 3, 5, 6, 12, 14, 19 and 21 all had at least one such interaction. This was not well reflected in *in vitro* activity with the noted exception of 5 which was seen to interact with His311 with a low binding affinity of −5.2 kcal mol^−1^. Residues 212–216 are responsible for phosphopantetheine pocket accessibility, and the activity of thiolactomycin anti-Tb drug is tied to them.^59^ Compounds 2, 3, 4, 5, 6, 8, 11, 12, 14, 19 and 21 had interactions at these sites and were largely false promises according to the *in vitro* results. The binding mode of 25 perplexingly was beyond the notable residues, but its significant bioactivity warrants a distinctive pathway. The *in silico* studies of 25 involve the interaction with Leu371 and the presence of Glu224 within the binding region showing similarity to rifampicin having Glu224 and Leu371 as interacting amino acids.^60^ Favourable non-covalent interactions were mostly by the heteroatomic functional groups present on the molecules. The binding modes of the two most active compounds 5 and 25 are shown in Fig. 6.

### 3.7. Cell cytotoxicity assay

**Fig. 7:**
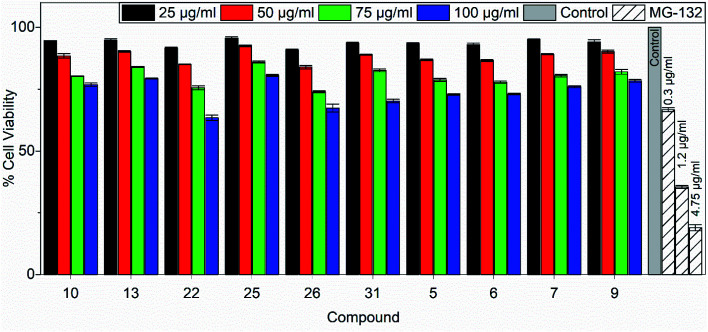
Percentage cell viability of HEK293 cells against 10 tested compounds post 48 hours of treatment. Data are represented as mean ± s.e.m.

Tetrazolium-based calorimetric cytotoxicity (MTT) assay is an important method to evaluate the viability of cells and it was performed for all the active chemical compounds using HEK293. To illustrate the percentage cell viability, the HEK293 cells were incubated with different concentrations of all active chemical compounds for 48 h. The compounds showing the viability of less than 50% of the cells were considered cytotoxic compounds. We observed that maximum compounds (such as 7, 9, 10, 13 and 25) show more than 75% of the cell viability at the highest concentration *i.e.* 100 μg ml^−1^ for 48 hours of the treatment. Similarly, other compounds (such as 5, 6, 22, 26 and 31) show 70% cell viability upon 48 hours of the treatment. Overall, data and results for all the compounds show that the tested compounds are not toxic to HEK293 cells when treated for up to 48 hours. MG-132, a potent proteasomal inhibitor and an inducer of apoptosis was used as a positive control in the assay. The percentage cell viability of HEK293 cells against 10 tested compounds post 48 hours of treatment is shown in Fig. 7.

## 4. Conclusion

A library of novel acetylene containing 2-(2-hydrazinyl)thiazole derivatives was designed based on physicochemical properties, synthesized and its potency against M. Tb H37Rv strain is studied. The parent acetylene containing aldehydes and thiosemicarbazones were also analyzed along with novel acetylene containing 2-(2-hydrazinyl)thiazole derivatives. Out of three acetylene containing thiosemicarbazones, two compounds are showing promising antitubercular activity with the most potent (*E*)-2-(3-ethoxy-4 (ethynyloxy)benzylidene)hydrazine-1-carbothioamide (5) inhibiting 74.58 ± 2.23% *Mycobacterium* at 50 μg ml^−1^. Out of 26 novel acetylene containing 2-(2-hydrazinyl)thiazole derivatives prepared, 8 compounds show 50–60% inhibition of *Mycobacterium* at 50 μg ml^−1^. The most promising derivative from this category was (*E*)-4-(2-(2-(5-bromo-2-(ethynyloxy)benzylidene)hydrazinyl)thiazol-4-yl)benzonitrile (25) with 61.86 ± 1.85% inhibition at 50 μg ml^−1^. The anti-Tb activity depends on the electronegativity of substituents and lipophilicity. The acetylene containing 2-(2-hydrazinyl)thiazole derivatives having electron-withdrawing substituents are showing better inhibition against M. Tb in LRP assay. The activity is also directly correlated to lower log *P* values. Halogens emerged out to be clear winners as 5 out of 8 active compounds were halo substituents. The docking studies run to understand the binding modes indicate that compound 5 interacts with His311, one of the amino acids in the main catalytic triad on KasA. In the case of the binding mode of 25, it interacts with Leu371 as in the case of rifampicin. Cytotoxicity studies results affirmed that the compounds tested compounds are not toxic to HEK293 cells when treated for up to 48 hours. Altogether, all these studies imply acetylene containing 2-(2-hydrazinyl)thiazole derivatives show promising anti-Tb activity. The derivatives need to be modified further for enhancing the anti-Tb activity and this work is under progress in our laboratory.

## Funding

Lakshmi Haritha Bharathi Maganti and Balaji Gowrivel Vijayakumar thank Pondicherry University for University Research Fellowship (URF). Deepthi Ramesh thanks DST (No. DST/INSPIRE Fellowship/IF170273 dated 5 January 2018) for the DST-INSPIRE fellowship (JRF). Authors thank the University Grants Commission (UGC), New Delhi for their financial support under Special Assistance Program, stage DSA-I to Department of Chemistry, Pondicherry University (No. F.540/6/DSA-1/2016/(SAP-1) dated 31-10-2018).

## Conflicts of interest

All the authors confirm that there are no conflicts of interest to declare.

## Supplementary Material

RA-012-D2RA00928E-s001

RA-012-D2RA00928E-s002
