# Supplementary material for: Acetylene containing 2-(2-hydrazinyl)thiazole derivatives: design, synthesis, and in vitro and in silico evaluation of antimycobacterial activity against Mycobacterium tuberculosis
Source: RSC Adv. 2022 Mar 21;12(14):8771–82. doi: 10.1039/d2ra00928e (PMC8984819; doi:10.1039/d2ra00928e)
Supplement: RA-012-D2RA00928E-s001 [file RA-012-D2RA00928E-s001.pdf]

Electronic Supplementary Material (ESI) for RSC Advances.
This journal is © The Royal Society of Chemistry 2022

Acetylene containing 2-(2-hydrazinyl)thiazole derivatives: Design, Synthesis, *in vitro* and *in silico* evaluation of antimycobacterial activity against *Mycobacterium tuberculosis*

Lakshmi Haritha Bharathi Maganti[a], Deepthi Ramesh[a], Balaji Gowrivel Vijayakumar[a], Mohd Imran K. Khan[b], Arunkumar Dhayalan[b], Jayabal Kamalraja[a], Tharanikkarasu Kannan[a,*]

[a]Department of Chemistry, Pondicherry University, Kalapet, Puducherry-605014, India.

[b]Department of Biotechnology, Pondicherry University, Kalapet, Puducherry-605014, India.

*Corresponding author: Tel: +91-413-265 4411; Fax: +91-413-265 6740.

Email address: tharani.che@pondiuni.edu.in

Contents

A. Procedure for *in vitro* anti-tubercular activity studies

B. Spectroscopic data (^{1}H and ^{13}C NMR, FT-IR and Mass) of compounds from **1** to **32**

C. Spectra (Mass, ^{1}H and ^{13}C NMR, and FT-IR) of compounds from **1** to **32**

D. Table S1. Library of acetylene containing 2-(2-hydrazinyl)thiazole derivatives used in the present investigation

E. Table S2. Pharmacokinetic analysis and *in vitro* mycobacterial analysis results of acetylene containing 2-(2-hydrazinyl)thiazole derivatives

F. Table S3. ADME properties of acetylene containing 2-(2-hydrazinyl)thiazole derivatives

G. Table S4. Single-crystal XRD data

H. Table S5. Summary of active compounds % inhibition at 50 µg/ml

I. Table S6. *In silico* studies of active compounds with KasA protein

J. Table S7. Summary of the analysis of variance (ANOVA) data at 50 µg/ml

K. Table S8. Summary of the analysis of variance (ANOVA) data at 100 µg/ml

L. Fig. S1. Reported biological compounds containing thiazole and acetylene pharmacophores

A In vitro anti-tubercular activity study

1. Preparation of sample

The stock solution was made by dissolving 10 mg of a sample in 1mL of DMSO. The working stock solutions of 1 mg/mL and 0.5 mg/mL were further prepared from this stock solution by the addition of an adequate volume of Middlebrook 7H9 broth. This was yet again sterilized by filtration with 0.45 µ filter.

2. Luciferase reporter mycobacteriophages (LRP) assay

Four cryovials per set (two for control and two for 100 µg/mL and 50 µg/mL concentrations) were taken. 400 µl of Middlebrook 7H9 broth was added into first two vials and 350 µl in the third and fourth vial. By the addition of 50 µl of 1mg/mL stock solution to the 3rd and 4th vials respectively, a total concentration of 400 µl was achieved. *M.tb*H_{37}Rv cell suspension of 100 µl concentration was added to the vials and these vials are incubated at 37 °C for 72 h. Then, 50 µl of phage phAE202 and 40 µl of 0.1M $CaCl_2$ were introduced to all the vials making the cell-phage mixture, and incubated at 37 °C for 4 h. After incubation, 100 µl of the cell-phage mixture was moved to a luminometer cuvette. 100 µl of D-luciferin was added and the relative light unit (RLU) was taken in a luminometer (Berthold) at 10S integration. The percentage of reduction in RLU of the test compared to control was calculated by using the following equation,

$$Percentage\ of\ Reduction\ in\ RLU = \frac{Control\ RLU - Test\ RLU}{Control\ RLU} \times 100$$

Compounds with 50% RLU reduction and above when compared with control were considered as active against *M.Tb*.

B. Spectroscopic data (^{1}H and ^{13}C NMR, FT-IR and Mass) of compounds from 1 to 32.

1. 2-(Prop-2-yn-1-yloxy)benzaldehyde (1)

White solid, Yield: 80%. M.P: 67-69°C. ^{1}H NMR (400 MHz, DMSO) δ 10.35 (s, 1H), 7.72 (d, *J* = 7.7 Hz, 1H), 7.68 (t, *J* = 7.0 Hz, 1H), 7.30 (d, *J* = 8.4 Hz, 1H), 7.13 (t, *J* = 7.5 Hz, 1H), 5.00 (s, 2H), 3.66 (s, 1H). ^{13}C NMR (101 MHz, DMSO) δ 188.99, 159.50, 136.19, 127.88, 124.79, 121.53, 114.27, 79.06, 78.63, 56.43. FT-IR (KBr, cm^{-1}): 3270.2 (alkyne C-H stretch), 2973.3 (aromatic C-H), 2876 (aldehyde C-H stretch), 2116.1 (alkyne -C≡C- stretch), 1684.6 (aldehyde C=O stretch), 1598 (aromatic C=C), 1264.5 (C-O stretch). ESI-HRMS: Calculated for $C_{10}H_8O_2$ = 160.0524; $[M+H]^+$ found = 161.0599.

2. 3-Ethoxy-4-(prop-2-yn-1-yloxy)benzaldehyde (2)

Pale yellow solid, Yield: 78%. M.P: 74-75°C. ^{1}H NMR (400 MHz, DMSO) δ 9.82 (s, 1H), 7.53 (d, *J* = 6.7 Hz, 1H), 7.39 (s, 1H), 7.21 (d, *J* = 8.3 Hz, 1H), 4.92 (s, 2H), 4.07 (d, *J* = 7.0 Hz, 2H), 3.50 (s, 1H), 1.33 (d, *J* = 7.0 Hz, 3H). ^{13}C NMR (101 MHz, DMSO) δ 191.79, 152.04, 148.80, 130.47, 125.45, 113.27, 111.15, 78.99, 78.82, 64.13, 56.20, 14.72. FT-IR (KBr, cm-1): 3253.8 (alkyne C-H stretch), 2988.2 (aromatic C-H), 2878.8 (aldehyde C-H stretch), 2736.3 (aldehyde C-H stretch), 2110 (alkyne -C≡C- stretch), 1688.2

(aldehyde C=O stretch), 1590.1 (aromatic C=C), 1268.1 (C-O stretch). ESI-HRMS: Calculated for $C_{12}H_{12}O_3$ = 204.0786; $[M+H]^+$ found = 205.0855.

3. 5-Bromo-2-(prop-2-yn-1-yloxy)benzaldehyde (3)

Light brown solid, Yield: 75%. M.P: 92-93°C. ^{1}H NMR (400 MHz, DMSO) δ 10.24 (s, 1H), 7.84 (d, *J* = 8.9 Hz, 1H), 7.76 (s, 1H), 7.28 (d, *J* = 8.9 Hz, 1H), 5.00 (s, 2H), 3.67 (s, 1H). ^{13}C NMR (101 MHz, DMSO) δ 187.99, 158.56, 138.36, 130.14, 126.36, 117.09, 113.47, 79.48, 78.33, 56.89. FT-IR (KBr, cm-1): 3237.4 (alkyne C-H stretch), 2980.7 (aromatic C-H), 2880.1 (aldehyde C-H stretch), 2769.3 (aldehyde C-H stretch), 2116.7 (alkyne -C≡C- stretch), 1680.9 (aldehyde C=O stretch), 1590.2 (aromatic C=C), 1273.5 (C-O stretch), 693 (C-Br stretch). ESI-HRMS: Calculated for $C_{10}H_7BrO_2$ = 237.9629; $[M+H]^+$ found = 238.9701.

4. (*E*)-2-(2-(Prop-2-yn-1-yloxy)benzylidene)hydrazine-1-carbothioamide (4)

White solid, Yield: 75%. M.P: 174-176°C. 1H NMR (400 MHz, DMSO) δ 11.47 (s, 1H), 8.40 (s, 1H), 8.17 (s, 1H), 8.11 (s, 1H), 7.96 (s, 1H), 7.38 (s, 1H), 7.13 (s, 1H), 7.00 (s, 1H), 4.89 (s, 2H), 3.62 (s, 1H). ^{13}C NMR (101 MHz, DMSO) δ 177.90, 155.75, 137.67, 131.07, 126.33, 122.91, 121.41, 113.29, 79.05, 78.68, 56.14. FT-IR (KBr, cm-1): 3385.8 (N-H stretch), 3270.2 (alkyne C-H stretch), 3023.2 (aromatic C-H), 2118.2 (alkyne -C≡C- stretch), 1595 (aromatic C=C), 1535.2 (C=N stretch), 1358.5 (C-N stretch), 1266 (C-O stretch). ESI-HRMS: Calculated for $C_{11}H_{11}N_3OS$ = 233.0623; $[M+H]^+$ found = 234.0695.

5. (*E*)-2-(3-Ethoxy-4-(prop-2-yn-1-yloxy)benzylidene)hydrazine-1-carbothioamide (5)

Light yellow solid, Yield: 78%. M.P: 190-192°C. ^{1}H NMR (400 MHz, DMSO) δ 11.31 (s, 1H), 8.14 (s, 1H), 8.02 (s, 1H), 7.95 (s, 1H), 7.51 (s, 1H), 7.14 (s, 1H), 7.04 (s, 1H), 4.83 (s, 2H), 4.09 (q, *J* = 7.0 Hz, 2H), 3.56 (s, 1H), 1.34 (t, *J* = 7.0 Hz, 3H). ^{13}C NMR (101 MHz, DMSO) δ 177.65, 148.79, 148.35, 142.49, 127.93, 121.74, 113.58, 109.99, 79.22, 78.55, 64.00, 55.94, 14.77. FT-IR (KBr, cm-1): 3321.9 (N-H stretch), 3265.9 (alkyne C-H stretch), 2982.4 (aromatic C-H), 2124.8 (alkyne -C≡C- stretch), 1593.5 (aromatic C=C), 1532.2 (C=N stretch), 1365.6 (C-N stretch), 1323, 1265.6 (C-O stretch). ESI-HRMS: Calculated for $C_{13}H_{15}N_{3}O2S$ = 277.0885; $[M+H]^{+}$ found = 278.0958.

6. (*E*)-2-(5-Bromo-2-(prop-2-yn-1-yloxy)benzylidene)hydrazine-1-carbothioamide (6)

Light brown solid, Yield: 80%. M.P: 204-206°C. ^{1}H NMR (400 MHz, DMSO) δ 11.49 (s, 1H), 8.33 (d, *J* = 10.8 Hz, 2H), 8.20 (s, 2H), 7.50 (s, 1H), 7.09 (s, 1H), 4.88 (s, 2H), 3.61 (s, 1H). ^{13}C NMR (101 MHz, DMSO) δ 178.10, 154.86, 136.12, 133.25, 128.38, 125.25, 115.74, 113.91, 79.03, 78.73, 56.57. FT-IR (KBr, cm-1): 3435.5 (N-H stretch), 3278.2 (alkyne C-H stretch), 2971.5 (aromatic C-H), 2118.7 (alkyne -C≡C- stretch), 1595.2 (aromatic C=C), 1530.6 (C=N stretch), 1363.1 (C-N stretch), 1266.7 (C-O stretch), 650.2 (C-Br stretch). ESI-HRMS: Calculated for $C_{11}H_{10}BrN_{3}OS$ = 312.9707; $[M+H]^{+}$ found = 311.9798.

7. (*E*)-4-Phenyl-2-(2-(2-(prop-2-yn-1-yloxy)benzylidene)hydrazinyl)thiazole (7)

White solid, Yield: 70%. M.P: 183-185°C. ^{1}H NMR (400 MHz, DMSO) δ 12.15 (s, 1H), 8.36 (s, 1H), 7.86 – 7.80 (m, 3H), 7.40 (s, 3H), 7.30 (d, *J* = 7.2 Hz, 2H), 7.16 (s, 1H), 7.06 (s, 1H), 4.90 (s, 2H), 3.61 (s, 1H). ^{13}C NMR (101 MHz, DMSO) δ 168.42, 155.14, 150.57, 136.75, 134.73, 130.67, 128.81, 127.76, 125.71, 125.24, 123.24, 121.80, 113.56, 103.82, 79.21, 78.78, 56.21. FT-IR (KBr,

cm-1): 3448.5 (N-H stretch), 3276.7 (alkyne C-H stretch), 2915.4 (aromatic C-H), 2120.8 (alkyne -C≡C- stretch), 1603.8 (aromatic C=C), 1570.8 (C=N stretch), 1366.3 (C-N stretch), 1272.9 (C-O stretch). ESI-HRMS: Calculated for $C_{19}H_{15}N_3OS$ = 333.0936; $[M+H]^+$ found = 334.1004.

8. (*E*)-4-(4-Fluorophenyl)-2-(2-(2-(prop-2-yn-1-yloxy)benzylidene)hydrazinyl)thiazole (8*)*

Pale yellow solid, Yield: 70%. M.P: 180-182°C. ^{1}H NMR (400 MHz, DMSO) δ 11.89 (s, 1H), 8.36 (s, 1H), 7.87 – 7.80 (m, 3H), 7.36 (s, 1H), 7.29 – 7.18 (m, 3H), 7.14 (d, *J* = 8.1 Hz, 1H), 7.05 (s, 1H), 4.89 (s, 2H), 3.60 (s, 1H). ^{13}C NMR (101 MHz, DMSO) δ 168.56, 163.03, 160.60, 155.19, 149.40, 137.02, 131.28, 130.74, 127.78, 125.27, 123.21, 121.81, 115.74, 115.53, 113.57, 103.59, 79.20, 78.78, 56.24. FT-IR (KBr, cm-1): 3439.6 (N-H stretch), 3280.1 (alkyne C-H stretch), 3188.6 (aromatic C-H), 2100 (alkyne -C≡C- stretch), 1602.5 (aromatic C=C), 1569.5 (C=N stretch), 1434.4 (C-F stretch), 1362.4 (C-N stretch), 1273.7 (C-O stretch). ESI-HRMS: Calculated for $C_{19}H_{14}FN_3OS$ = 351.0842; $[M+H]^+$ found = 352.0912.

9. (*E*)-4-(4-Chlorophenyl)-2-(2-(2-(prop-2-yn-1-yloxy)benzylidene)hydrazinyl)thiazole (9)

White solid, Yield: 75%. M.P: 160-164°C. ^{1}H NMR (400 MHz, DMSO) δ 12.19 (s, 1H), 8.36 (s, 1H), 7.88 – 7.79 (m, 3H), 7.46 (d, *J* = 8.2 Hz, 2H), 7.37 (d, *J* = 7.6 Hz, 2H), 7.16 (s, 1H), 7.06 (s, 1H), 4.91 (s, 2H), 3.63 (s, 1H). ^{13}C NMR (101 MHz, DMSO) δ 168.39, 155.05, 149.23, 136.76, 133.50, 131.97, 130.54, 128.66, 127.28, 125.09, 123.07, 121.64, 113.45, 104.50, 79.07, 78.71, 56.12. FT-IR (KBr, cm-1): 3457.5 (N-H stretch), 3284.9 (alkyne C-H stretch), 2100 (alkyne -C≡C- stretch), 1627.5 (C=N stretch), 1602.6

(aromatic C=C), 1567.9 (C=N stretch), 1359 (C-N stretch), 1229.6 (C-O stretch), 828 (C-Cl stretch). ESI-HRMS: Calculated for $C_{19}H_{14}ClN_3OS$ = 367.0546; $[M+H]^+$ found = 368.0616.

10. (*E*)-4-(3,4-Dichlorophenyl)-2-(2-(2-(prop-2-yn-1-yloxy)benzylidene)hydrazinyl)thiazole (10)

White solid, Yield: 75%. M.P: 175-177^{0}C. ^{1}H NMR (400 MHz, DMSO) δ 12.19 (s, 1H), 8.36 (s, 1H), 8.06 (s, 1H), 7.80 (d, *J* = 7.9 Hz, 2H), 7.66 (s, 1H), 7.52 (s, 1H), 7.37 (s, 1H), 7.16 (s, 1H), 7.05 (s, 1H), 4.90 (s, 2H), 3.62 (s, 1H). ^{13}C NMR (101 MHz, DMSO) δ 168.53, 155.10, 147.97, 136.97, 135.24, 131.49, 130.91, 130.65, 129.76, 127.21, 125.64, 125.14, 123.05, 121.68, 113.5, 105.96, 79.10, 78.72, 56.16. FT-IR (KBr, cm-1): 3450.9 (N-H stretch), 3295.5 (alkyne C-H stretch), 2100 (alkyne -C≡C- stretch), 1602 (aromatic C=C), 1568.1 (C=N stretch), 1356 (C-N stretch), 1226.4 (C-O stretch), 747.9 (C-Cl stretch). ESI-HRMS: Calculated for $C_{19}H_{13}Cl_2N_3OS$ = 401.0156; $[M+H]^+$ found = 402.0230.

11. (*E*)-4-(4-Bromophenyl)-2-(2-(2-(prop-2-yn-1-yloxy)benzylidene)hydrazinyl)thiazole (11)

Light yellowish brown solid, Yield: 70%. M.P: 144-146°C. ^{1}H NMR (400 MHz, DMSO) δ 12.17 (s, 1H), 8.35 (s, 1H), 7.80 (d, *J* = 8.4 Hz, 3H), 7.59 (d, *J* = 8.4 Hz, 2H), 7.37 (s, 2H), 7.16 (s, 1H), 7.06 (s, 1H), 4.91 (s, 2H), 3.62 (s, 1H). ^{13}C NMR (101 MHz, DMSO) δ 168.44, 155.07, 149.43, 136.73, 133.93, 131.60, 130.58, 127.63, 125.13, 123.12, 121.69, 120.59, 113.48, 104.6, 79.11, 78.71. FT-IR (KBr, cm-1): 3461.1 (N-H stretch), 3272.1 (alkyne C-H stretch), 3072.3 (aromatic C-H), 2100 (alkyne -C≡C- stretch), 1604.8 (aromatic C=C), 1569.4 (C=N stretch), 1357.3 (C-N stretch), 1225.1 (C-O stretch), 692.1 (C-Br stretch). ESI-HRMS: Calculated for $C_{19}H_{14}BrN_3OS$ = 413.0022; $[M+H]^+$ found = 412.0111.

12. (*E*)-4-(4-Nitrophenyl)-2-(2-(2-(prop-2-yn-1-yloxy)benzylidene)hydrazinyl)thiazole (12)

Orange red solid, Yield: 75%. M.P: 185-187°C. ^{1}H NMR (400 MHz, DMSO) δ 12.28 (s, 1H), 8.37 (s, 1H), 8.26 (d, *J* = 8.5 Hz, 2H), 8.09 (d, *J* = 8.7 Hz, 2H), 7.80 (d, *J* = 7.7 Hz, 1H), 7.69 (s, 1H), 7.38 (t, *J* = 7.7 Hz, 1H), 7.15 (d, *J* = 8.4 Hz, 1H), 7.06 (t, *J* = 7.5 Hz, 1H), 4.91 (s, 2H), 3.63 (s, 1H). ^{13}C NMR (101 MHz, DMSO) δ 168.44, 155.07, 149.43, 136.73, 133.93, 131.60, 130.58, 127.63, 125.13, 123.12, 121.69, 120.59, 113.48, 104.6, 79.11, 78.71, 56.14. FT-IR (KBr, cm-1): 3310.6 (N-H stretch), 3267.7 (alkyne C-H stretch), 2110 (alkyne -C≡C- stretch), 1598.7 (aromatic C=C), 1564.9 (C=N stretch), 1500.1 (N-O stretch), 1329.7 (N-O stretch), 1280.8 (C-N stretch), 1239.7 (C-O stretch). ESI-HRMS: Calculated for $C_{19}H_{14}N_4O_3S$ = 378.0787; $[M+H]^+$ found = 379.0858.

13. (*E*)-4-(2-(2-(2-(Prop-2-yn-1-yloxy)benzylidene)hydrazinyl)thiazol-4-yl)benzonitrile (13)

Light green solid, Yield: 65%. M.P: 150-152°C. ^{1}H NMR (400 MHz, DMSO) δ 12.23 (s, 1H),8.36 (s, 1H), 8.01 (d, *J* = 8.1 Hz, 2H), 7.84 (d, *J* = 8.1 Hz, 2H), 7.79 (s, 1H), 7.58 (s, 1H), 7.37 (s, 1H), 7.16 (s, 1H), 7.05 (s, 1H), 4.90 (s, 2H), 3.60 (s, 1H). ^{13}C NMR (101 MHz, DMSO) δ 168.72, 155.19, 148.95, 138.85, 137.15, 136.92, 132.83, 130.76, 126.29, 125.24, 123.12, 121.79, 119.17, 109.71, 107.66, 79.18, 78.76, 56.24. FT-IR (KBr, cm-1): 3445.7 (N-H stretch), 3277.2 (alkyne C-H stretch), 2224.1 (C≡N stretch), 2110 (alkyne -C≡C- stretch), 1605.8 (aromatic C=C), 1566.8 (C=N stretch), 1361 (C-N stretch), 1228.1 (C-O stretch). ESI-HRMS: Calculated for $C_{20}H_{14}N_4OS$ = 358.0888; $[M+H]^+$ found = 359.0959.

14. (*E*)-2-(2-(2-(Prop-2-yn-1-yloxy)benzylidene)hydrazinyl)-4-(p-tolyl)thiazole (14)

White solid, Yield: 70%. M.P: 128-130°C. ^{1}H NMR (400 MHz, DMSO) δ 8.34 (s, 1H), 7.81 (s, 1H), 7.73 (d, *J* = 8.1 Hz, 2H), 7.37 (s, 1H), 7.20 (d, *J* = 5.8 Hz, 3H), 7.16 (s, 1H), 7.06 (s, 1H), 4.90 (s, 2H), 3.60 (s, 1H), 2.30 (s, 3H). ^{13}C NMR (101 MHz, DMSO) δ 168.3, 155.11, 150.64, 137.01, 136.62, 132.09, 130.62, 129.35, 125.64, 125.20, 123.26, 121.79, 113.56, 102.87, 79.20, 78.75, 56.20, 20.96. FT-IR (KBr, cm-1): 3287.7 (alkyne C-H stretch), 2921.3 (aromatic C-H), 2110 (alkyne -C≡C- stretch), 1602.9 (aromatic C=C), 1568.7 (C=N stretch), 1362.4 (C-N stretch), 1224.9 (C-O stretch). ESI-HRMS: Calculated for $C_{20}H_{17}N_3OS$= 347.1092; [M+H]$^+$ found = 348.1161.

15. (*E*)-4-(4-Methoxyphenyl)-2-(2-(2-(prop-2-yn-1-yloxy)benzylidene)hydrazinyl)thiazole (15)

White solid, Yield: 75%. M.P: 181-183°C. ^{1}H NMR (400 MHz, DMSO) δ 8.35 (s, 1H), 7.81 (dd, J = 7.8, 1.7 Hz, 1H), 7.77 (d, J = 8.9 Hz, 2H), 7.38 (s, 1H), 7.16 (d, J = 7.7 Hz, 2H), 7.06 (t, J = 7.5 Hz, 1H), 6.97 (d, J = 8.9 Hz, 2H), 4.91 (s, 2H), 3.78 (s, 3H), 3.63 (s, 1H). 13C NMR (101 MHz, DMSO) δ 168.34, 167.09, 159.17, 155.27, 148.15, 138.21, 130.94, 129.05, 127.22, 126.32, 125.30, 122.87, 121.71, 114.12, 109.07, 101.95, 79.09, 78.80, 56.21, 55.27, 39.52. FT-IR (KBr, cm-1): 3450.4 (N-H stretch), 3252.2 (alkyne C-H stretch), 3052.9 (aromatic C-H), 2115.9 (alkyne -C≡C- stretch), 1622.1 (aromatic C=C), 1567.6 (C=N stretch), 1329 (C-N stretch), 1256.6 (C-O stretch), 1184.2 (C-O stretch). ESI-HRMS: Calculated for C20H17N3O2S = 363.1041; [M+H] + found = 364.1112.

16. (*E*)-2-(2-(3-Ethoxy-4-(prop-2-yn-1-yloxy)benzylidene)hydrazinyl)-4-phenylthiazole (16)

White solid, Yield: 72%. M.P: 221-223°C. ^{1}H NMR (400 MHz, DMSO) δ 8.01 (s, 1H), 7.84 (d, J = 7.1 Hz, 2H), 7.41 (t, J = 7.6 Hz, 2H), 7.31 (t, J = 7.3 Hz, 3H), 7.19 (dd, J = 8.4, 1.8 Hz, 1H), 7.08 (d, J = 8.4 Hz, 1H), 4.85 (s, 2H), 4.07 (d, J = 7.0 Hz, 2H), 3.59 (s, 1H), 1.36 (t, J = 7.0 Hz, 3H). ^{13}C NMR (101 MHz, DMSO) δ 168.55, 167.18, 148.76, 148.15, 142.67, 133.69, 129.51, 128.84, 128.49, 128.01, 125.83, 120.24, 114.12, 110.08, 103.89, 90.74, 79.26, 78.67, 63.95, 56.09, 14.84. FT-IR (KBr, cm-1): 3428.5 (N-H stretch), 3201.8 (alkyne C-H stretch), 2934.3 (aromatic C-H), 2111.7 (alkyne -C≡C- stretch), 1624.2 (aromatic C=C), 1510.2 (C=N stretch), 1323.1 (C-N stretch), 1269.3 (C-O stretch), 1223.1 (C-O stretch). ESI-HRMS: Calculated for C21H19N3O2S = 377.1198; [M+H] + found = 378.1263.

17. (*E*)-2-(2-(3-Ethoxy-4-(prop-2-yn-1-yloxy)benzylidene)hydrazinyl)-4-(4-fluorophenyl)thiazole (17)

Pale yellow solid, Yield: 78%.M.P: 152-154°C. ^{1}H NMR (400 MHz, DMSO) δ 12.05 (s, 1H), 7.96 (s, 1H), 7.87 (d, *J* = 8.9 Hz, 2H), 7.28 (s, 1H), 7.26 (s, 1H), 7.21 (d, *J* = 8.9 Hz, 2H), 7.16 (s, 1H), 7.08 (s, 1H), 4.84 (s, 2H), 4.08 (s, 2H), 3.56 (s, 1H), 1.36 (s, 3H). ^{13}C NMR (101 MHz, DMSO) δ 168.52, 160.46, 149.55, 148.71, 147.87, 141.43, 131.40, 128.27, 127.52, 119.81, 115.53, 114.12, 109.90, 79.25, 78.56, 63.86, 56.03, 14.78. FT-IR (KBr, cm-1): 3449.7 (N-H stretch), 3278.5 (alkyne C-H stretch), 2983.8 (aromatic C-H), 2110 (alkyne -C≡C- stretch), 1570 (C=N stretch), 1511.1 (aromatic C=C), 1407.3 (C-F stretch), 1358 (C-N stretch), 1265.6 (C-O stretch), 1225.3 (C-O stretch). ESI-HRMS: Calculated for $C_{21}H_{18}FN_3O_2S$ = 395.1104; $[M+H]^+$ found = 396.1178.

18. (*E*)-4-(4-Chlorophenyl)-2-(2-(3-ethoxy-4-(prop-2-yn-1-yloxy)benzylidene)hydrazinyl)thiazole (18)

White solid, Yield: 65%. M.P: 185-186°C. ^{1}H NMR (400 MHz, DMSO) δ 12.06 (s, 1H), 7.96 (s, 1H), 7.86 (d, *J* = 8.6 Hz, 2H), 7.45 (d, *J* = 8.6 Hz, 2H), 7.34 (s, 1H), 7.27 (s, 1H), 7.16 (s, 1H), 7.08 (s, 1H), 4.84 (s, 2H), 4.08 (q, *J* = 7.0 Hz, 2H), 3.56 (s, 1H), 1.36 (t, *J* = 6.9 Hz, 3H). ^{13}C NMR (101 MHz, DMSO) δ 168.55, 149.35, 148.71, 147.89, 141.50, 133.63, 131.98, 128.69, 128.24, 127.28, 119.82, 114.13, 109.93, 104.32, 79.23, 78.54, 63.86, 56.03, 14.77. FT-IR (KBr, cm-1): 3437.8 (N-H stretch), 3310.5 (alkyne C-H stretch), 2875.9 (aromatic C-H), 2110 (alkyne -C≡C- stretch), 1572.4 (C=N stretch), 1508.5 (aromatic C=C), 1367.1 (C-N stretch), 1264.6 (C-O stretch), 1220.6 (C-O stretch), 826.4 (C-Cl stretch). ESI-HRMS: Calculated for $C_{21}H_{18}ClN_3O_2S$ = 411.0808; $[M+H]^+$ found = 412.0879.

19. (*E*)-4-(3,4-Dichlorophenyl)-2-(2-(3-ethoxy-4-(prop-2-yn-1-yloxy)benzylidene)hydrazinyl)thiazole (19)

White solid, Yield: 60%. M.P: 153-155°C. ^{1}H NMR (400 MHz, DMSO) δ 12.15 (s, 1H), 8.06 (s, 1H), 7.96 (s, 1H), 7.82 (d, *J* = 8.2 Hz, 1H), 7.64 (d, *J* = 8.4 Hz, 1H), 7.50 (s, 1H), 7.27 (s, 1H), 7.16 (d, *J* = 8.1 Hz, 1H), 7.07 (d, *J* = 8.3 Hz, 1H), 4.84 (s, 2H), 4.06 (q, *J* = 13.4, 6.6 Hz, 2H), 3.57 (s, 1H), 1.36 (s, 3H). ^{13}C NMR (101 MHz, DMSO) δ 168.64, 148.69, 147.93, 141.71, 135.29, 131.51, 130.92, 129.73, 128.15, 127.21, 125.60, 119.89, 114.08, 109.89, 105.79, 79.22, 78.56, 63.84, 56.02, 14.77. FT-IR (KBr, cm-1): 3527.6 (N-H stretch), 3276.9 (alkyne C-H stretch), 2982.2, 2935.6 (aromatic C-H), 2115 (alkyne -C≡C- stretch), 1573.9 (C=N stretch), 1510.9 (aromatic C=C), 1356.1 (C-N stretch), 1264.2 (C-O stretch), 1220.3 (C-O stretch), 862 (C-Cl stretch). ESI-HRMS: Calculated for $C_{21}H_{17}Cl_2N_3O_2S$ = 445.0419; $[M+H]^+$ found = 446.0492.

20. (*E*)-4-(4-Bromophenyl)-2-(2-(3-ethoxy-4-(prop-2-yn-1-yloxy)benzylidene)hydrazinyl)thiazole (20)

Light brown solid, Yield: 75%. M.P: 187-189°C. ^{1}H NMR (400 MHz, $CDCl_3$) δ 7.96 (s, 1H), 7.79 (d, *J* = 8.5 Hz, 2H), 7.58 (d, *J* = 8.5 Hz, 2H), 7.36 (s, 1H), 7.28 (s, 1H), 7.17 (d, *J* = 8.4 Hz, 1H), 7.07 (d, *J* = 8.4 Hz, 1H), 4.84 (s, 2H), 4.07 (q, *J* = 6.9 Hz, 2H), 3.57 (s, 1H), 1.36 (t, *J* = 6.9 Hz, 3H). ^{13}C NMR (101 MHz, DMSO) δ 168.55, 149.40, 148.70, 147.88, 141.51, 133.98, 131.61, 128.22, 127.60, 120.57, 119.84, 114.11, 109.89, 104.43, 79.23, 78.56, 63.85, 56.02, 14.78. FT-IR (KBr, cm-1): 3449 (N-H stretch), 3308 (alkyne C-H stretch), 2115 (alkyne -C≡C- stretch), 1571.8 (C=N stretch), 1508.3 (aromatic C=C), 1366.9 (C-N stretch), 1264.1 (C-O stretch), 1220.7 (C-O stretch), 621.7 (C-Br stretch). ESI-HRMS: Calculated for $C_{21}H_{18}BrN_3O_2S$ = 457.0284; $[M+H]^+$ found = 456.0363.

21. (*E*)-2-(2-(3-Ethoxy-4-(prop-2-yn-1-yloxy)benzylidene)hydrazinyl)-4-(4-nitrophenyl)thiazole (21)

Orange red solid, Yield: 78%. M.P:191-193°C. ^{1}H NMR (400 MHz, DMSO) δ 12.15 (s, 1H), 8.25 (d, *J* = 9.0 Hz, 2H), 8.08 (d, *J* = 9.0 Hz, 2H), 7.97 (s, 1H), 7.66 (s, 1H), 7.27 (s, 1H), 7.17 (d, *J* = 8.4 Hz, 1H), 7.07 (d, *J* = 8.4 Hz, 1H), 4.84 (s, 2H), 4.08 (q, *J* = 7.0 Hz, 2H), 3.56 (s, 1H), 1.36 (t, *J* = 6.9 Hz, 3H). ^{13}C NMR (101 MHz, DMSO) δ 168.83, 148.71, 147.96, 146.24, 143.59, 140.78, 128.13, 126.38, 125.45, 124.18, 119.93, 114.07, 109.90, 108.45, 79.23, 78.57, 63.86, 56.03, 14.78. FT-IR (KBr, cm-1): 3450 (N-H stretch), 3279.1 (alkyne C-H stretch), 2936.2 (aromatic C-H), 2110 (alkyne -C≡C- stretch), 1597.2 (aromatic C=C), 1575.5 (C=N stretch), 1510.3 (N-O stretch), 1344.3 (N-O stretch), 1266.1 (C-N stretch), 1225.1 (C-O stretch). ESI-HRMS: Calculated for $C_{21}H_{18}N_4O_4S$ = 422.1049; $[M+H]^+$ found = 423.1118.

22. (*E*)-4-(2-(2-(3-Ethoxy-4-(prop-2-yn-1-yloxy)benzylidene)hydrazinyl)thiazol-4-yl)benzonitrile (22)

Light grey solid, Yield: 70%. M.P: 178-180°C. ^{1}H NMR (400 MHz, DMSO) δ 12.10 (s, 1H), 8.04 – 7.96 (m, 3H), 7.87 (d, *J* = 17.9 Hz, 2H), 7.58 (s, 1H), 7.27 (s, 1H), 7.16 (s, 1H), 7.08 (s, 1H), 4.84 (s, 2H), 4.09 – 4.04 (q, 2H), 3.56 (s, 1H), 1.36 (t, *J* = 6.9 Hz, 3H). ^{13}C NMR (101 MHz, DMSO) δ 168.75, 148.77, 147.96, 141.84, 138.84, 132.78, 128.15, 126.21, 119.93, 119.12, 114.10, 109.93, 109.64, 107.45, 79.24, 78.59, 63.87, 56.04, 14.79. FT-IR (KBr, cm-1): 3528.6 (N-H stretch), 3290.1 (alkyne C-H stretch), 2879.6 (aromatic C-H), 2223.5 (C≡N stretch), 2115 (alkyne -C≡C- stretch), 1604.2 (aromatic C=C), 1572 (C=N stretch), 1356.3 (C-N stretch), 1266.2 (C-O stretch), 1221.9 (C-O stretch). ESI-HRMS: Calculated for $C_{22}H_{18}N_4O_2S$ = 402.1150; $[M+H]^+$ found = 403.1222.

23. (*E*)-2-(2-(3-Ethoxy-4-(prop-2-yn-1-yloxy)benzylidene)hydrazinyl)-4-(p-tolyl)thiazole (23)

White solid, Yield: 76%. M.P: 166-168°C. ^{1}H NMR (400 MHz, DMSO) δ 7.98 (s, 1H), 7.72 (d, *J* = 8.1 Hz, 2H), 7.28 (s, 1H), 7.20 (d, *J* = 6.2 Hz, 3H), 7.16 (s, 1H), 7.08 (s, 1H), 4.84 (d, *J* = 2.3 Hz, 2H), 4.07 (d, *J* = 7.0 Hz, 2H), 3.57 (s, 1H), 2.31 (s, 3H), 1.36 (t, *J* = 6.9 Hz, 3H). ^{13}C NMR (101 MHz, DMSO) δ 168.36, 150.09, 148.72, 147.92, 141.72, 137.03, 131.82, 129.30, 128.23, 125.60, 119.90, 114.12, 109.94, 102.69, 79.25, 78.58, 63.87, 56.04, 20.91, 14.79. FT-IR (KBr, cm-1): 3450.4 (N-H stretch), 3312.3 (alkyne C-H stretch), 2872.3 (aromatic C-H), 2115 (alkyne -C≡C- stretch), 1573.5 (C=N stretch), 1508.9 (aromatic C=C), 1368.8 (C-N stretch), 1264.8 (C-O stretch), 1220.1 (C-O stretch). ESI-HRMS: Calculated for $C_{22}H_{21}N_3O_2S$ = 391.1354; $[M+H]^+$ found = 392.1418.

24. (*E*)-2-(2-(3-Ethoxy-4-(prop-2-yn-1-yloxy)benzylidene)hydrazinyl)-4-(4-methoxyphenyl)thiazole (24)

White solid, Yield: 80%. M.P: 183-185°C. ^{1}H NMR (400 MHz, DMSO) δ 12.02 (s, 1H), 7.95 (s, 1H), 7.77 (d, *J* = 8.8 Hz, 2H), 7.27 (s, 1H), 7.15 (s, 1H), 7.09 (d, *J* = 8.6 Hz, 2H), 6.96 (d, *J* = 8.9 Hz, 2H), 4.84 (s, 2H), 4.07 (q, *J* = 6.9 Hz, 2H), 3.77 (s, 3H), 3.57

(s, 1H), 1.36 (t, *J* = 6.9 Hz, 3H). ^{13}C NMR (101 MHz, DMSO) δ 168.29, 158.84, 151.40, 148.70, 147.82, 141.21, 128.33, 127.65, 126.92, 119.76, 114.08, 109.88, 101.36, 79.25, 78.55, 63.86, 56.03, 55.19, 14.79. FT-IR (KBr, cm-1): 3446.9 (N-H stretch), 3287.1 (alkyne C-H stretch), 2838.4 (aromatic C-H), 2118.9 (alkyne -C≡C- stretch), 1574.9 (C=N stretch), 1507.5 (aromatic C=C), 1368.9 (C-N stretch), 1264.3 (C-O stretch), 1220.3 (C-O stretch), 1177.3 (C-O stretch). ESI-HRMS: Calculated for $C_{22}H_{21}N_3O_3S$ = 407.1304; $[M+H]^+$ found = 408.1374.

25. (*E*)-2-(2-(5-Bromo-2-(prop-2-yn-1-yloxy)benzylidene)hydrazinyl)-4-phenylthiazole (25)

White solid, Yield: 70%. M.P: 195-197°C. ^{1}H NMR (400 MHz, DMSO) δ 8.27 (s, 1H), 7.90 – 7.80 (m, 3H), 7.52 (d, *J* = 2.6 Hz, 1H), 7.49 – 7.38 (m, 2H), 7.38 – 7.16 (m, 2H), 7.13 (d, *J* = 8.9 Hz, 1H), 4.91 (s, 2H), 3.65 (s, 1H). ^{13}C NMR (101 MHz, DMSO) δ 168.13, 154.21, 150.24, 135.20, 134.40, 132.73, 128.62, 127.92, 127.06, 125.71, 125.46, 116.07, 113.62, 104.11, 79.12, 78.75, 56.59. FT-IR (KBr, cm-1): 3339.9 (N-H stretch), 3296.3 (alkyne C-H stretch), 2867.8 (aromatic C-H), 2120 (alkyne -C≡C- stretch), 1620.2 (aromatic C=C), 1565.2 (C=N stretch), 1362.8 (C-N stretch), 1227.6 (C-O stretch), 628.2 (C-Br stretch). ESI-HRMS: Calculated for $C_{19}H_{14}BrN_3OS$ = 413.0022; $[M+H]^+$ found = 412.0115.

26. (*E*)-2-(2-(5-Bromo-2-(prop-2-yn-1-yloxy)benzylidene)hydrazinyl)-4-(4-fluorophenyl)thiazole (26)

Pale yellow solid, Yield: 80%. M.P: 207-209°C. ^{1}H NMR (400 MHz, DMSO) δ 8.27 (s, 1H), 7.88 (dd, J = 8.7, 5.6 Hz, 2H), 7.85 (s, 1H), 7.54 (dd, J = 8.8, 2.6 Hz, 1H), 7.32 (s, 1H), 7.23 (t, J = 8.9 Hz, 2H), 7.14 (d, J = 8.9 Hz, 1H), 4.92 (s, 2H), 3.67 (s, 1H). ^{13}C NMR (101 MHz, DMSO) δ 168.23, 166.85, 163.07, 160.62, 154.29, 146.94, 136.03, 132.96, 130.11, 127.74, 127.13, 125.11,

115.44 (s), 113.65, 103.91, 91.18, 79.14, 78.68, 56.61, 39.52. FT-IR (KBr, cm-1): 3424.8 (N-H stretch), 3213.1 (alkyne C-H stretch), 2932.4 (aromatic C-H), 2115.4 (alkyne -C≡C- stretch), 1622.2 (aromatic C=C), 1507.5 (C=N stretch), 1477.7, 1414.1 (C-F stretch), 1367 (C-N stretch), 1233.3 (C-O stretch), 626.6 (C-Br stretch). ESI-HRMS: Calculated for C19H13BrFN3OS = 430.9927; [M+H] + found = 430.0019.

27. (*E*)-2-(2-(5-Bromo-2-(prop-2-yn-1-yloxy)benzylidene)hydrazinyl)-4-(4-chlorophenyl)thiazole (27)

White solid, Yield: 75%. M.P: 209-211°C. 1H NMR (400 MHz, DMSO) δ 8.27 (s, 1H), 7.85 (t, J = 5.4 Hz, 3H), 7.54 (dd, J = 8.8, 2.6 Hz, 1H), 7.46 (d, J = 8.6 Hz, 2H), 7.40 (s, 1H), 7.14 (d, J = 8.9 Hz, 1H), 4.92 (s, 2H), 3.67 (s, 1H). 13C NMR (101 MHz, DMSO) δ 168.23, 166.90, 154.28, 146.63, 136.04, 132.89, 132.40, 129.62, 128.72, 128.45, 127.39, 127.12, 125.36, 125.06, 115.92, 104.85, 91.91, 79.13, 78.66, 56.58, 48.71, 39.52. FT-IR (KBr, cm-1): 3425 (N-H stretch), 3211.9 (alkyne C-H stretch), 2877 (aromatic C-H), 2116.9 (alkyne -C≡C- stretch), 1627.3 (aromatic C=C), 1522.6 (C=N stretch), 1364.1 (C-N stretch), 1225 (C-O stretch), 822.5 (C-Cl stretch), 626.1 (C-Br stretch). ESI-HRMS: Calculated for C19H13BrClN3OS = 446.9629; [M+H] + found = 445.9723.

28. (*E*)-2-(2-(5-Bromo-2-(prop-2-yn-1-yloxy)benzylidene)hydrazinyl)-4-(3,4-dichlorophenyl)thiazole (28)

White solid, Yield: 68%. M.P: 213-215°C. ^{1}H NMR (400 MHz, DMSO) δ 12.46 (s, 1H), 8.25 (s, 1H), 8.04 (s, 1H), 7.82 (d, *J* = 18.2 Hz, 2H), 7.64 (s, 1H), 7.53 (d, *J* = 5.2 Hz, 2H), 7.13 (s, 1H), 4.91 (s, 2H), 3.65 (s, 1H). ^{13}C NMR (101 MHz, DMSO) δ 168.26, 166.93, 154.28, 147.96, 145.04, 134.02, 131.16, 130.73, 129.40, 127.69, 125.36, 124.98, 115.87, 113.59, 106.21, 93.07, 79.12, 78.66, 56.57, 39.57. FT-IR (KBr, cm-1): 3436.8 (N-H stretch), 3295.9 (alkyne C-H stretch), 2927.9 (aromatic C-H), 2118.1 (alkyne -C≡C- stretch), 1621.2 (aromatic C=C),

1370.8 (C-N stretch), 1224.7 (C-O stretch), 810.5 (C-Cl stretch), 770.9 (C-Cl stretch), 626.9 (C-Br stretch). ESI-HRMS: Calculated for $C_{19}H_{12}BrCl_2N_3OS$ = 480.9238; $[M+H]^+$ found = 479.9334.

29. (*E*)-2-(2-(5-Bromo-2-(prop-2-yn-1-yloxy)benzylidene)hydrazinyl)-4-(4-bromophenyl)thiazole (29)

Light reddish brown solid, Yield: 65%. M.P: 208-210°C. 1H NMR (400 MHz, DMSO) δ 12.38 (s, 1H), 8.26 (s, 1H), 7.85 (d, J = 2.6 Hz, 1H), 7.81 (dd, J = 8.7, 4.4 Hz, 2H), 7.67 (d, J = 8.7 Hz, 1H), 7.59 (d, J = 8.6 Hz, 1H), 7.56 (s, 1H), 7.42 (s, 1H), 7.14 (d, J = 8.9 Hz, 1H), 4.93 (s, 2H), 3.67 (s, 1H). 13C NMR (101 MHz, CDCl3) δ 168.22, 154.19, 149.22, 146.77, 133.70, 132.77, 131.66, 129.94, 127.69, 125.39, 121.60, 120.75, 116.03, 113.58, 104.97, 91.97, 79.14, 78.73, 56.15, 39.52, 18.65. FT-IR (KBr, cm-1): 3412.9 (N-H stretch), 3209.7 (alkyne C-H stretch), 2874.5 (aromatic C-H), 2116.7 (alkyne -C≡C- stretch), 1625.6 (aromatic C=C), 1364.5 (C-N stretch), 1225.0 (C-O stretch), 626.4 (C-Br stretch). Calculated for C19H13Br2N3OS = 490.9126; [M+H] + found = 489.9218.

30. (*E*)-4-(2-(2-(5-Bromo-2-(prop-2-yn-1-yloxy)benzylidene)hydrazinyl)thiazol-4-yl)benzonitrile (30)

Light grey solid, Yield: 65%. M.P: 199-201°C. ^{1}H NMR (400 MHz, DMSO) δ 12.37 (s, 1H), 8.26 (s, 1H), 8.01 (d, *J* = 8.6 Hz, 2H), 7.87 (d, *J* = 15.8 Hz, 3H), 7.61 (s, 1H), 7.53 (d, 1H), 7.12 (d, *J* = 9.0 Hz, 1H), 4.91 (s, 2H), 3.64 (s, 1H). ^{13}C NMR (101 MHz, DMSO) δ 168.41, 154.21, 148.95, 138.75, 135.27, 132.80, 128.53, 127.05, 126.26, 125.38, 119.13, 116.04, 113.61, 109.72, 107.91, 79.12, 78.75, 56.59. FT-IR (KBr, cm-1): 3406.3 (N-H stretch), 3265.2 (alkyne C-H stretch), 3112.8 (aromatic C-H), 2226.2 (C≡N stretch), 2115 (alkyne -C≡C- stretch), 1624.9 (aromatic C=C), 1564.3 (C=N stretch), 1270.1 (C-N stretch), 1226.5 (C-O stretch), 631.6 (C-Br stretch). Calculated for $C_{20}H_{13}BrN_4OS$ = 437.9974; $[M+H]^+$ found = 437.0069.

31. (*E*)-2-(2-(5-Bromo-2-(prop-2-yn-1-yloxy)benzylidene)hydrazinyl)-4-(p-tolyl)thiazole (31)

White solid, Yield: 70%. M.P: 191-193°C. ^{1}H NMR (400 MHz, DMSO) δ 8.29 (s, 1H), 7.86 (s, 1H), 7.72 (d, J = 6.9 Hz, 2H), 7.54 (d, J = 8.9 Hz, 1H), 7.26 (s, 1H), 7.21 (d, J = 8.1 Hz, 2H), 7.14 (d, J = 8.9 Hz, 1H), 4.92 (s, 2H), 3.67 (s, 1H), 2.31 (s, 3H). ^{13}C NMR (101 MHz, DMSO) δ 168.12, 166.71, 154.27, 147.87, 137.79, 135.90, 132.91, 130.80, 128.95, 127.88, 127.11, 125.15, 115.97, 113.63, 103.35, 90.63, 79.14, 78.67, 56.60, 48.71, 39.52, 21.00. FT-IR (KBr, cm-1): 3407.2 (N-H stretch), 3236 (alkyne C-H stretch), 2917 (aromatic C-H), 2117.6 (alkyne -C≡C- stretch), 1621.5 (aromatic C=C), 1507.4 (C=N stretch), 1270.4 (C-N stretch), 1228 (C-O stretch), 628.2 (C-Br stretch). Calculated for C20H16BrN3OS = 427.0178; [M+H] + found = 426.0272.

32. (*E*)-2-(2-(5-Bromo-2-(prop-2-yn-1-yloxy)benzylidene)hydrazinyl)-4-(4-methoxyphenyl)thiazole (32)

Pale yellow solid, Yield: 60%. M.P: 184-187°C. ^{1}H NMR (400 MHz, DMSO) δ 12.00 (s, 1H), 8.27 (s, 1H), 7.90 (d, J = 7.6 Hz, 3H), 7.60 (d, J = 9.0 Hz, 1H), 7.13 (s, 1H), 7.12 (d, J = 2.6 Hz, 2H), 6.89 (d, J = 9.0 Hz, 1H), 4.92 (s, 2H), 3.87 (s, 1H), 3.71 (s, 3H). ^{13}C NMR (101 MHz, DMSO) δ 168.10, 166.65, 159.17, 154.32, 147.72, 135.96, 132.94, 129.42, 127.24, 125.33, 116.10, 114.18, 113.64, 109.33, 102.18, 89.70, 79.18, 78.75, 56.64, 55.32, 39.52. FT-IR (KBr, cm-1): 3379.4 (N-H stretch), 3285.4 (alkyne C-H stretch), 2832.8 (aromatic C-H), 2120.8 (alkyne -C≡C- stretch), 1623.6 (aromatic C=C), 1511.1 (C=N stretch), 1359.9 (C-N stretch), 1228.3 (C-O stretch), 1186.6 (C-O stretch), 634.3 (C-Br stretch). Calculated for C20H16BrN3O2S = 443.0128; [M+H] + found = 442.0218.

C. Spectra (Mass, ¹H and ¹³C NMR, and FT-IR) of compounds from 1 to 32

Mass spectrum of 2-(Prop-2-yn-1-yloxy)benzaldehyde (1)

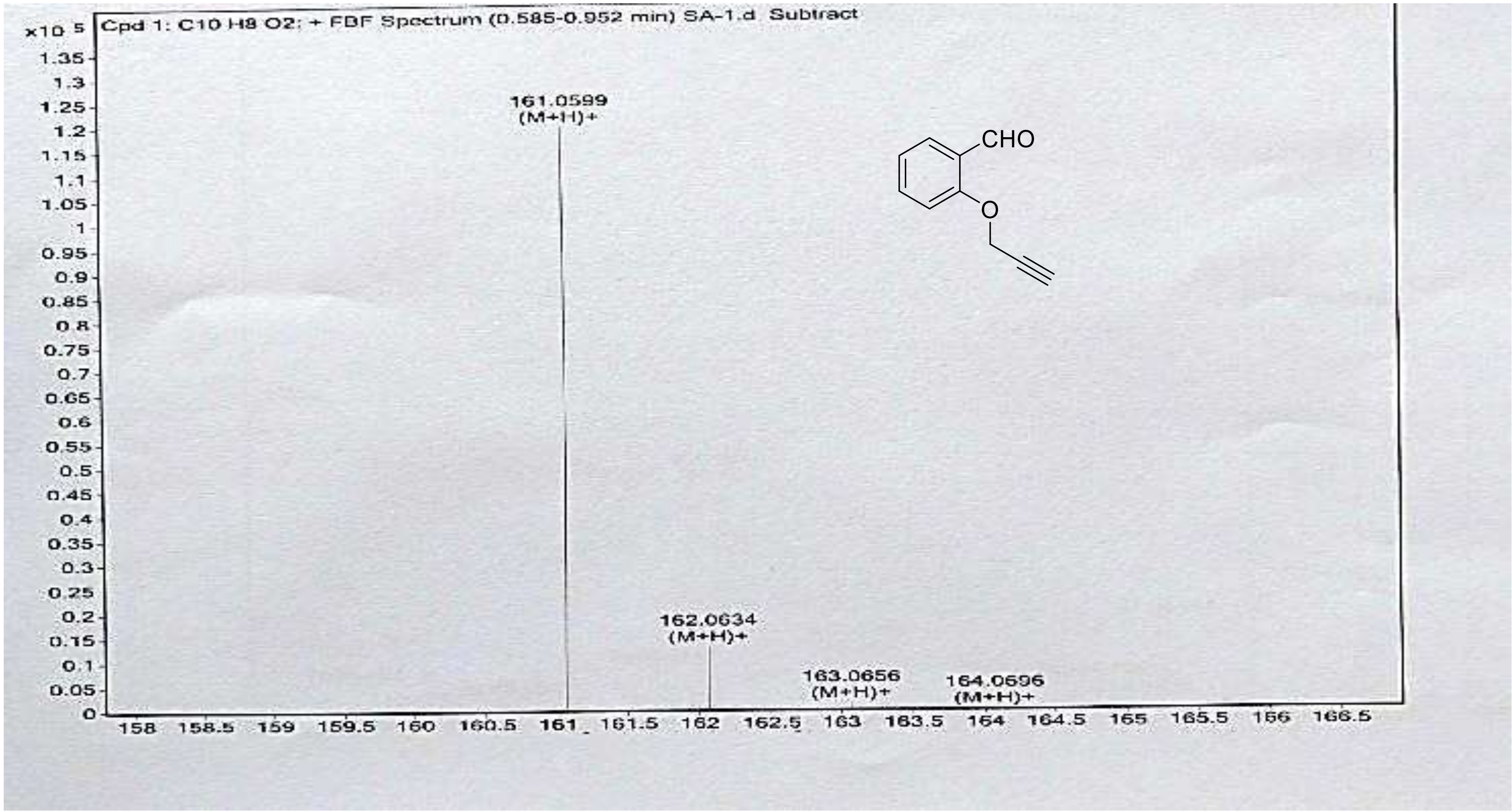

^{1}H NMR spectrum of 2-(Prop-2-yn-1-yloxy)benzaldehyde (1)

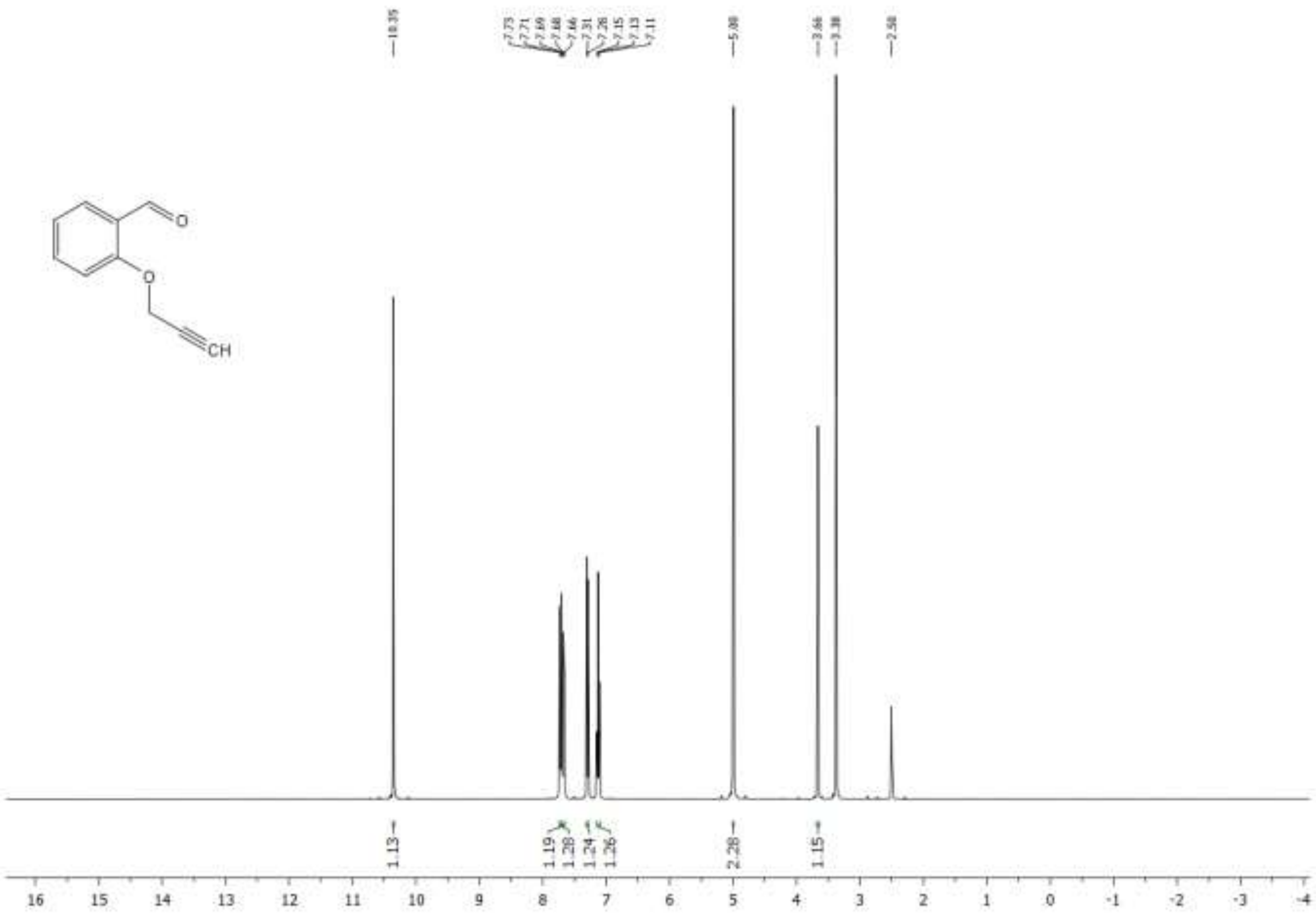

^{13}C NMR spectrum of 2-(Prop-2-yn-1-yloxy)benzaldehyde (1)

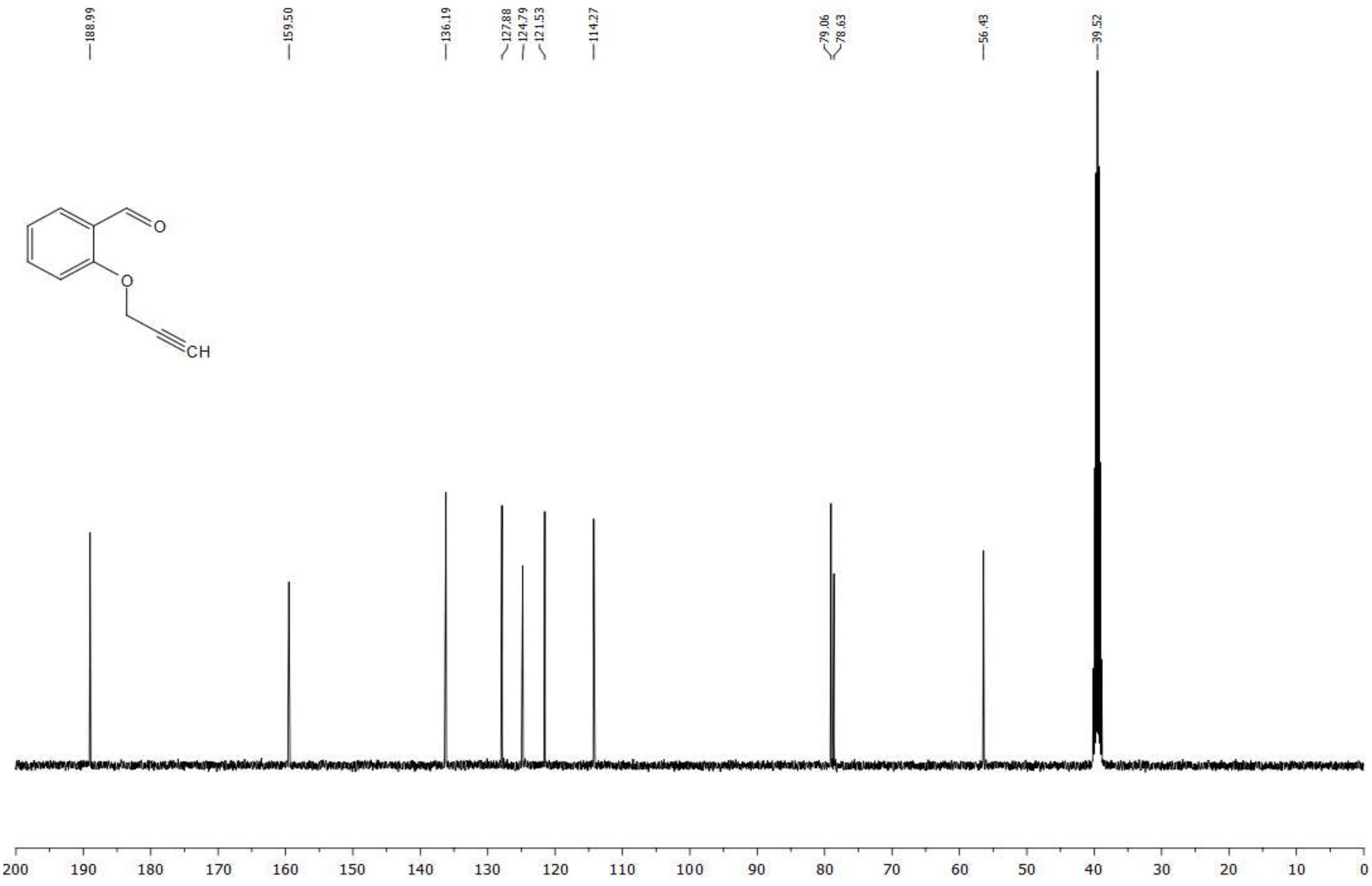

FT-IR spectrum of 2-(Prop-2-yn-1-yloxy)benzaldehyde (1)

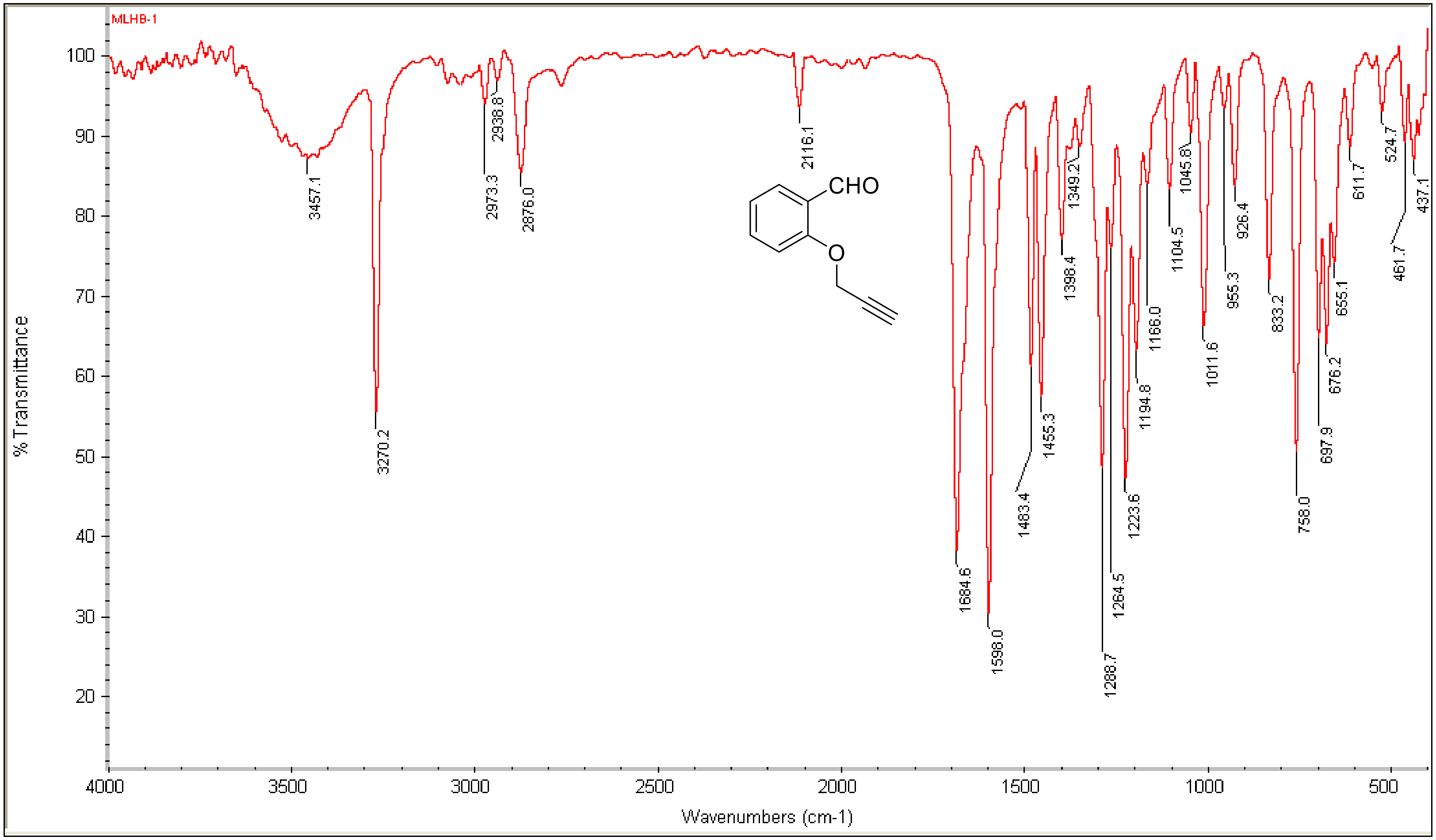

Mass spectrum of 3-Ethoxy-4-(prop-2-yn-1-yloxy)benzaldehyde (2)

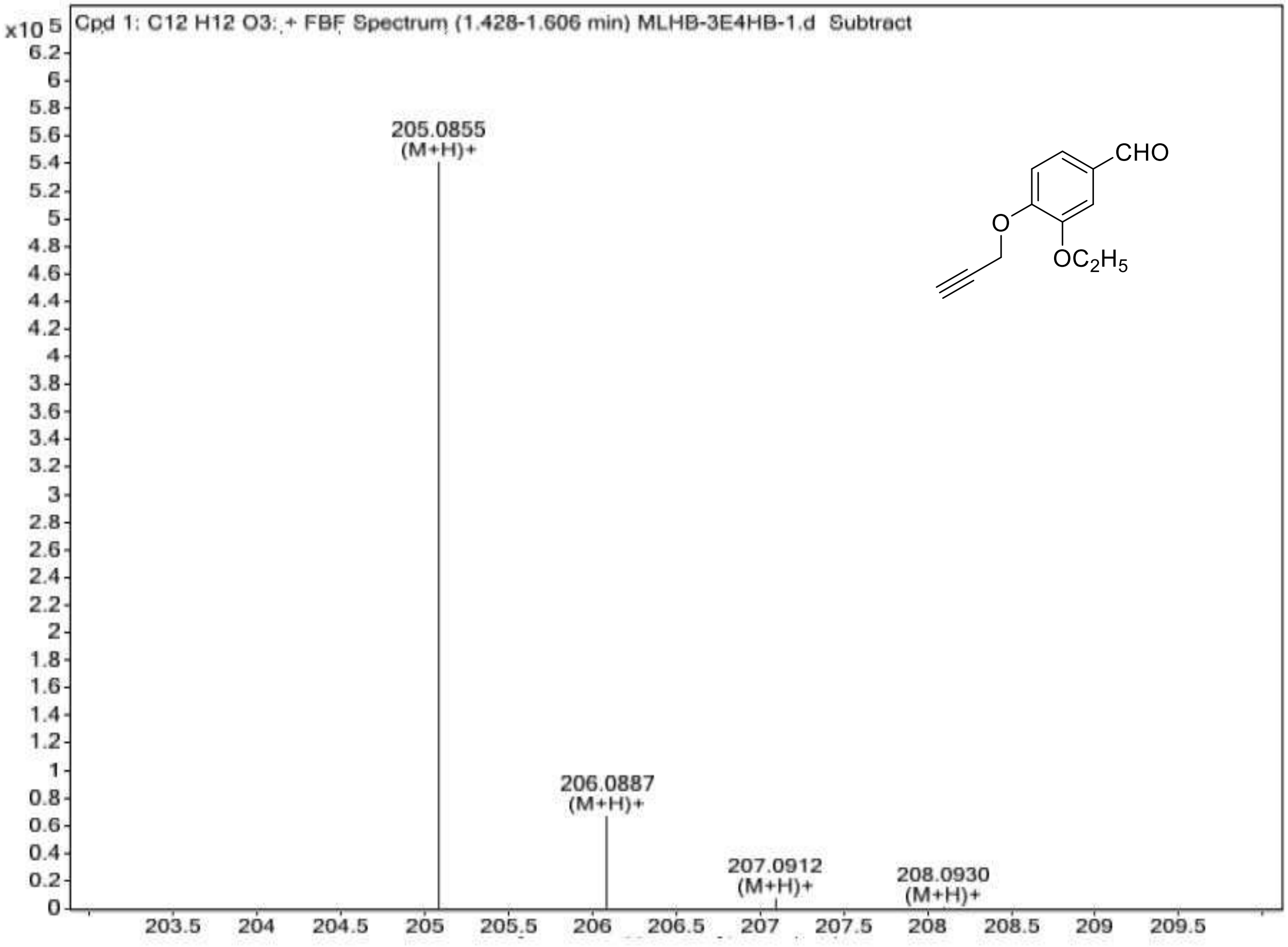

^{1}H NMR spectrum of 3-Ethoxy-4-(prop-2-yn-1-yloxy)benzaldehyde (2)

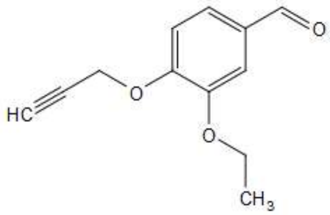

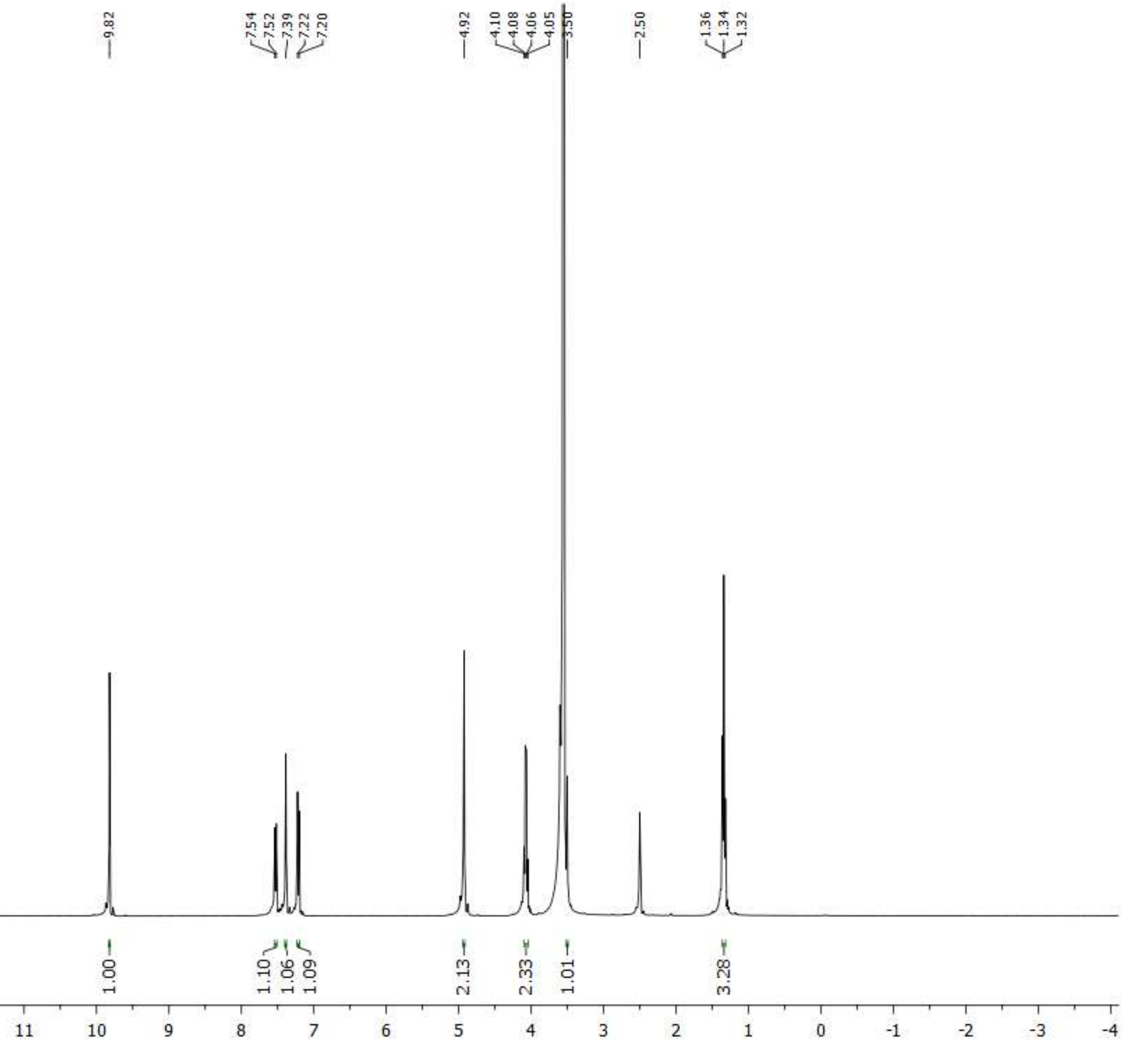

^{13}C NMR spectrum of 3-Ethoxy-4-(prop-2-yn-1-yloxy)benzaldehyde (2)

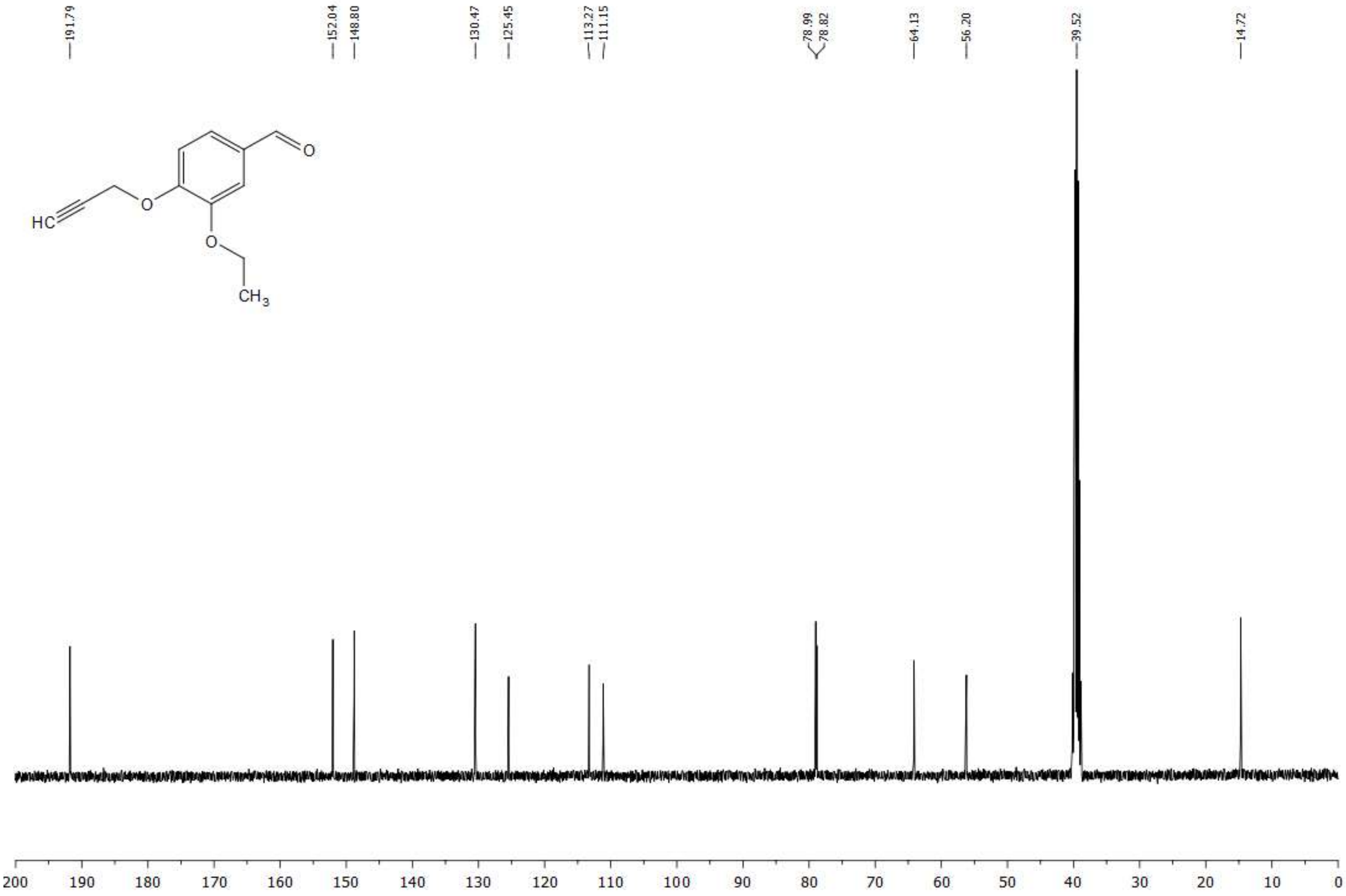

FT-IR spectrum of 3-Ethoxy-4-(prop-2-yn-1-yloxy)benzaldehyde (2)

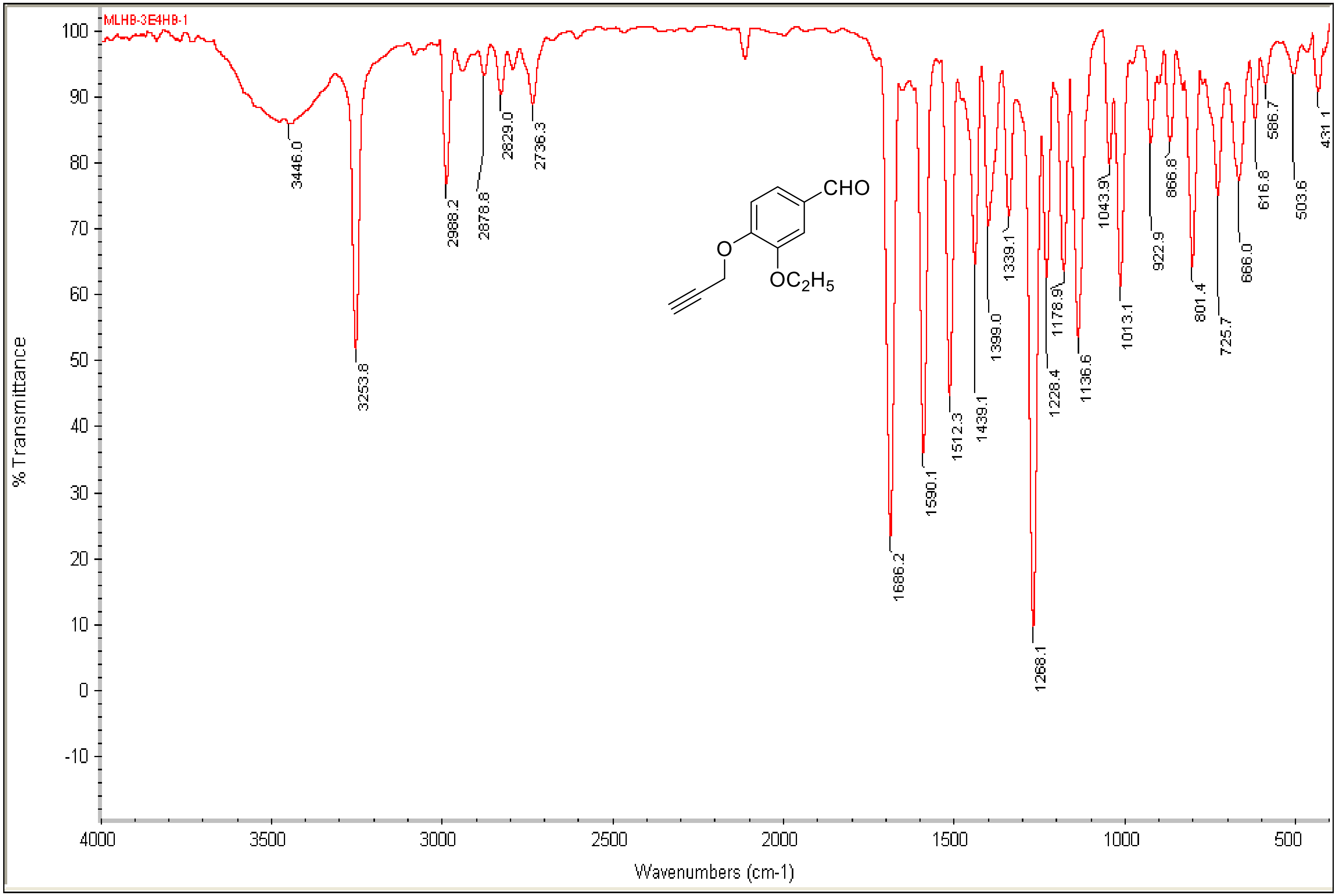

Mass spectrum of 5-Bromo-2-(prop-2-yn-1-yloxy)benzaldehyde (3)

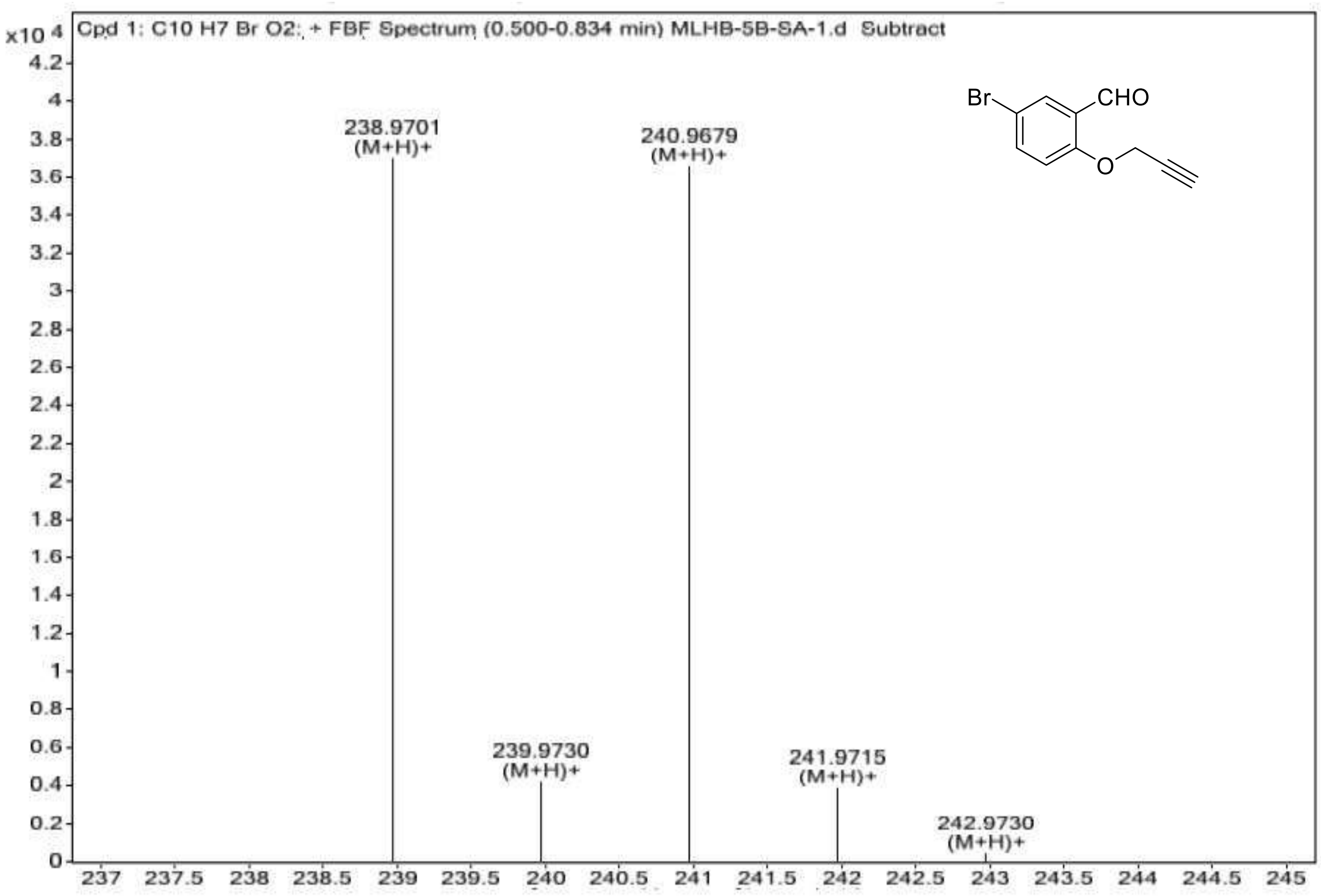

^{1}H NMR spectrum of 5-Bromo-2-(prop-2-yn-1-yloxy)benzaldehyde (3)

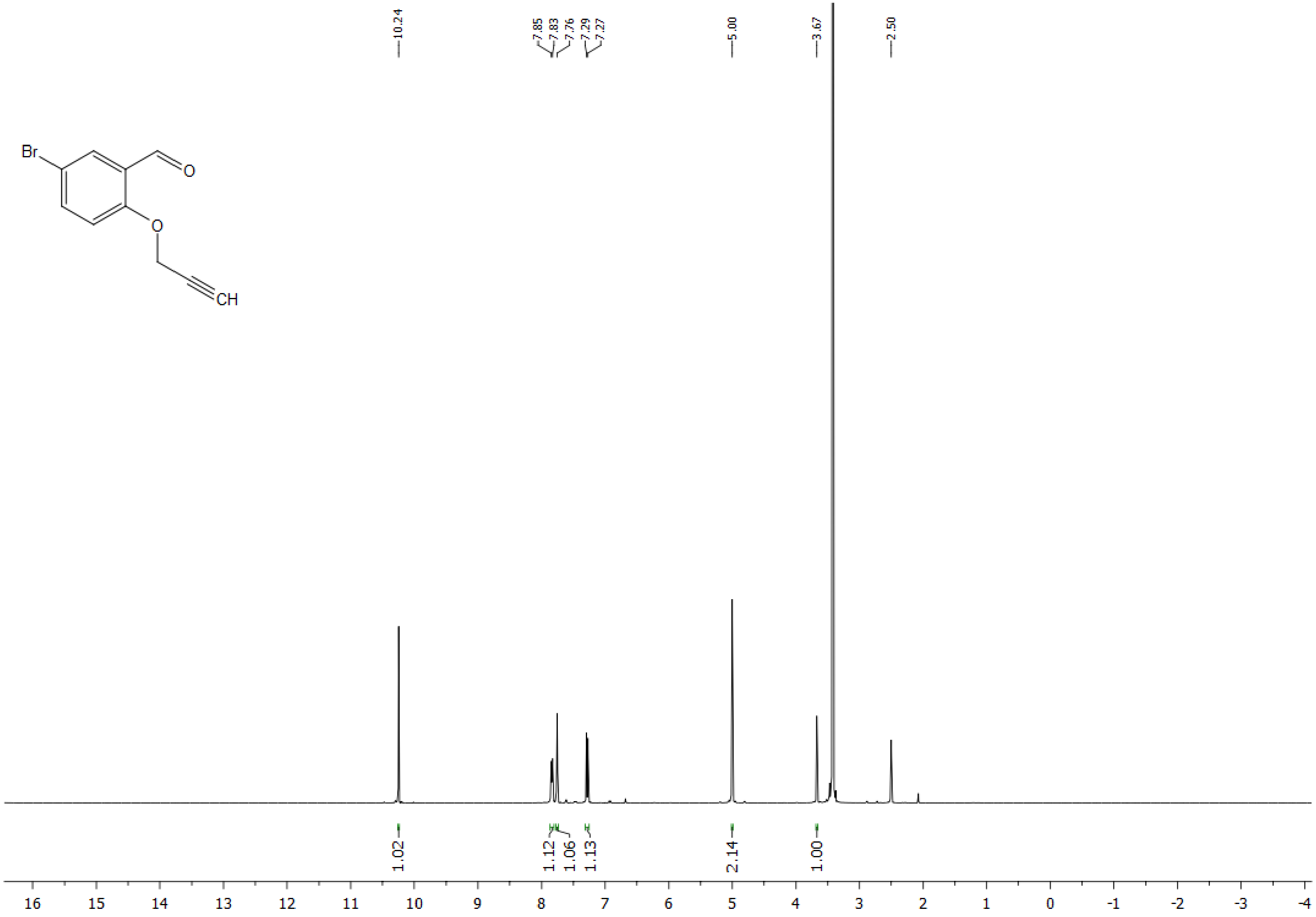

^{13}C NMR spectrum of 5-Bromo-2-(prop-2-yn-1-yloxy)benzaldehyde (3)

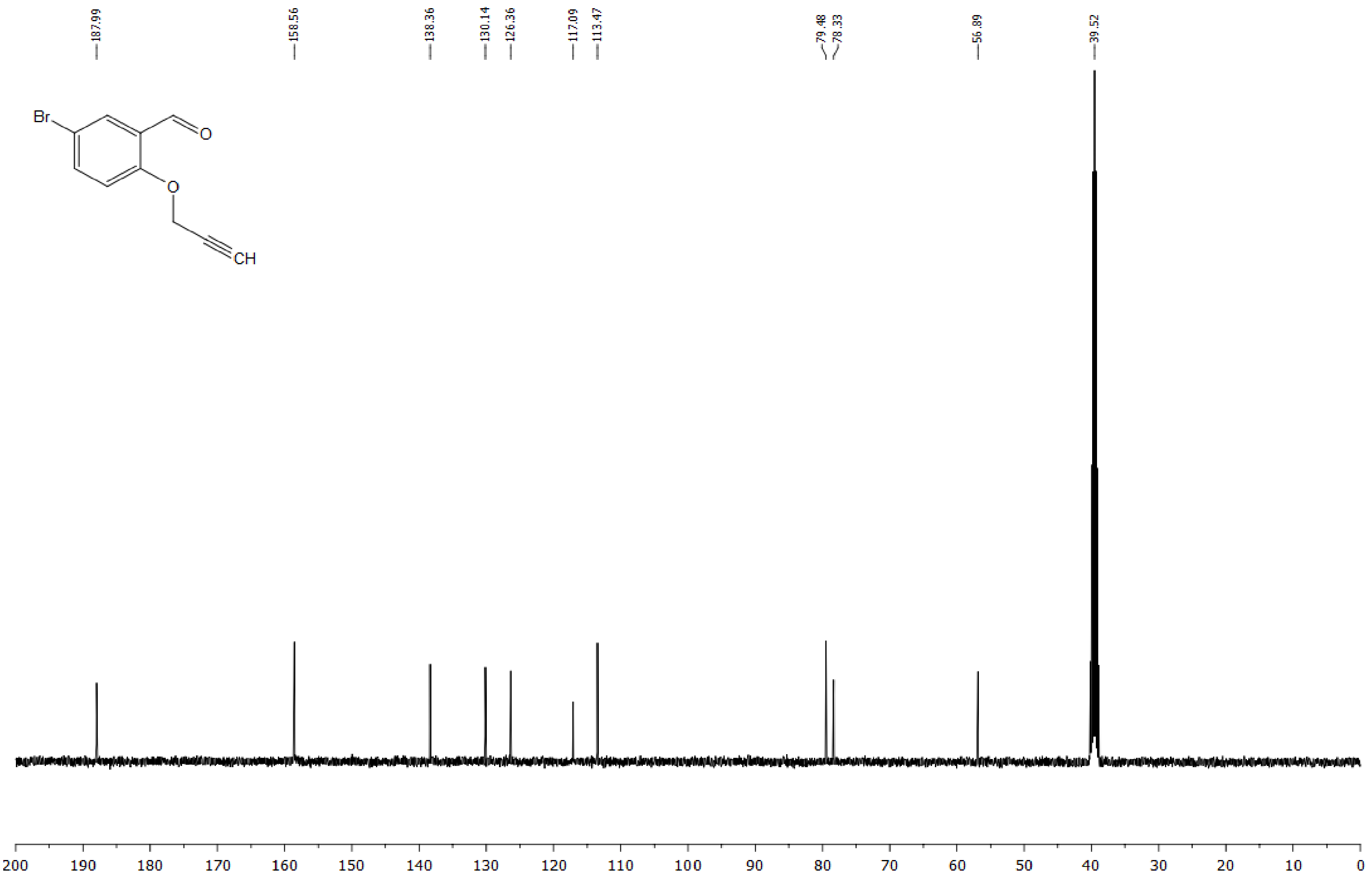

FT-IR spectrum of 5-Bromo-2-(prop-2-yn-1-yloxy)benzaldehyde (3)

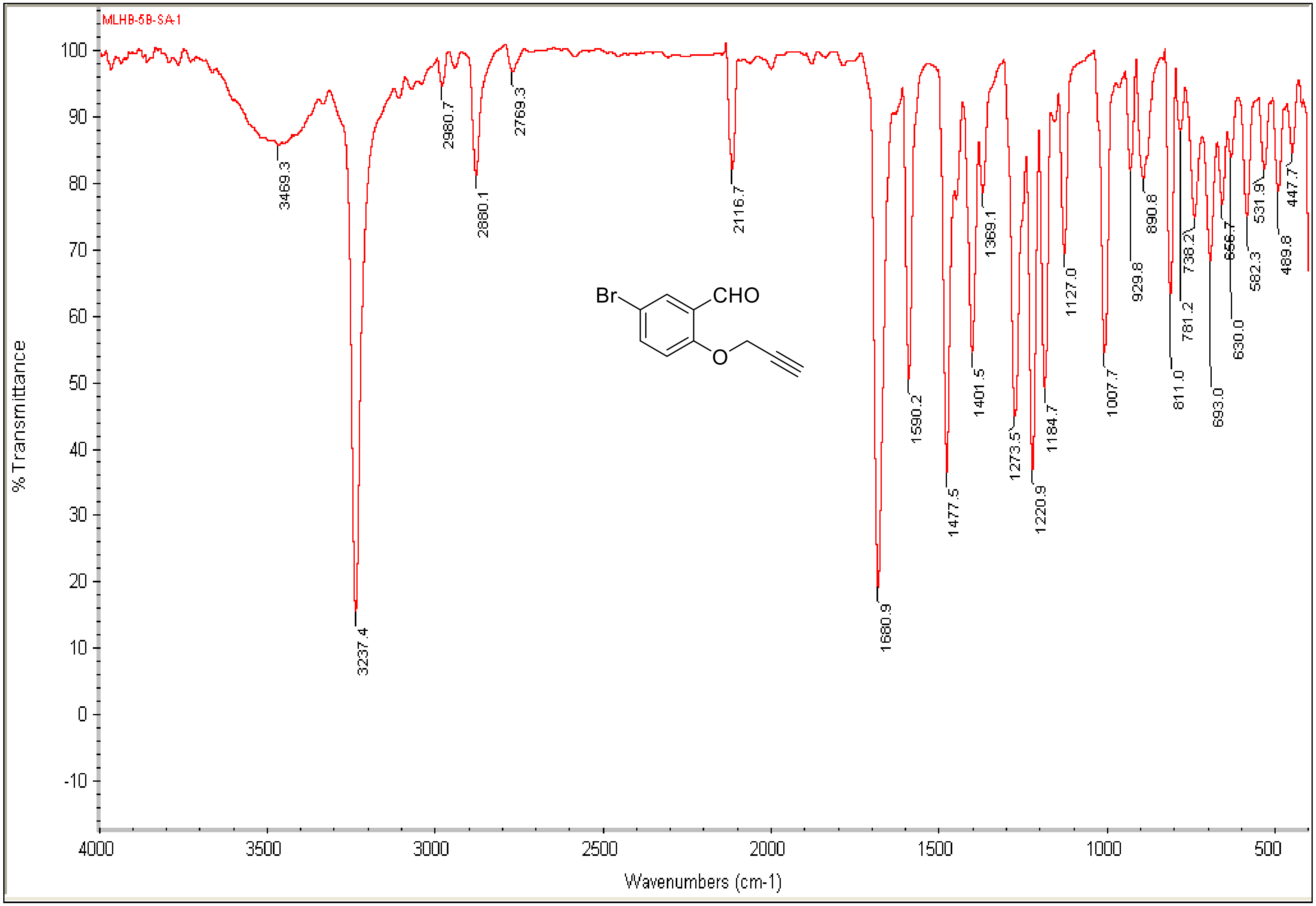

Mass spectrum of (*E*)-2-(2-(Prop-2-yn-1-yloxy)benzylidene)hydrazine-1-carbothioamide (4)

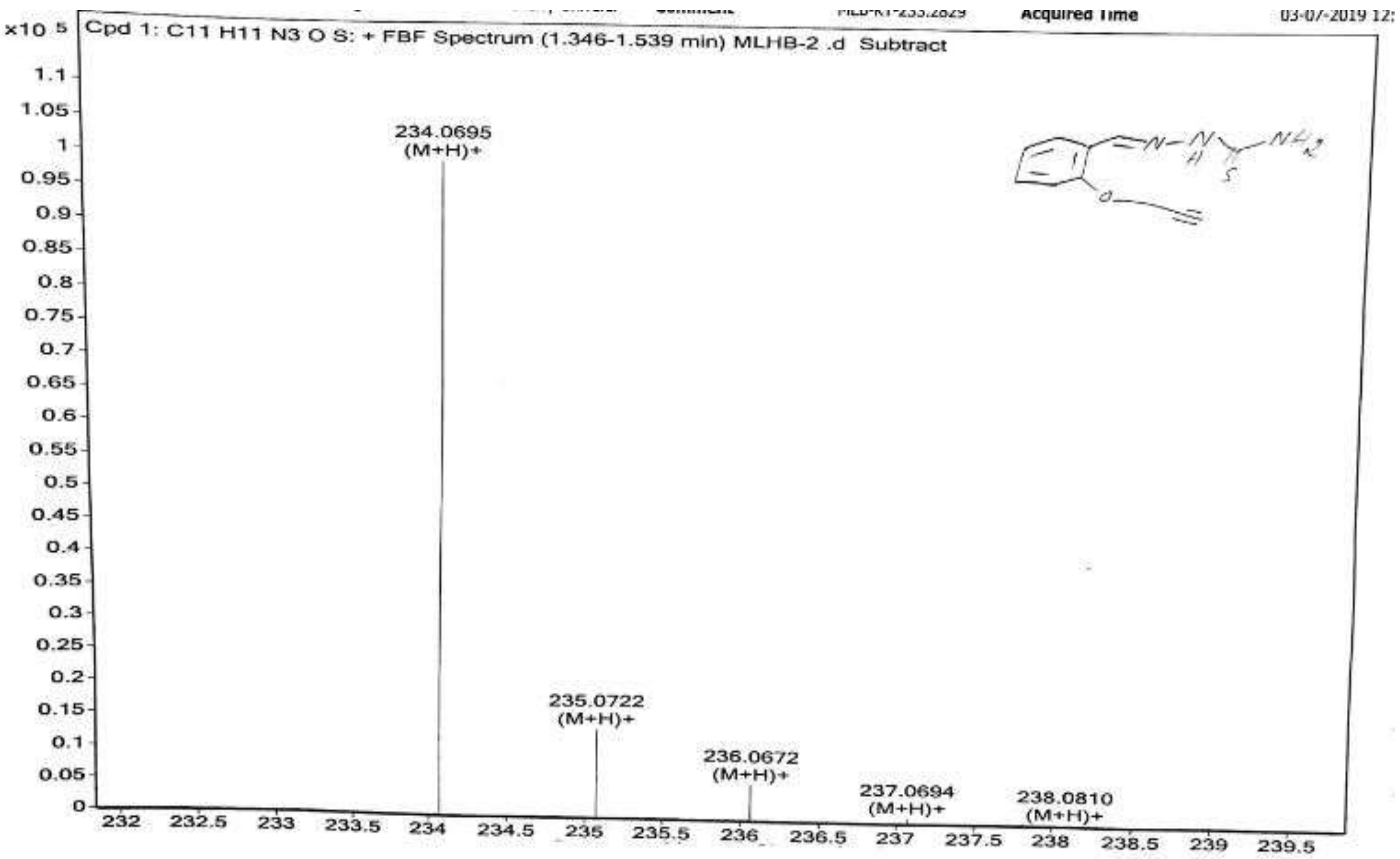

^{1}H NMR spectrum of (*E*)-2-(2-(Prop-2-yn-1-yloxy)benzylidene)hydrazine-1-carbothioamide (4)

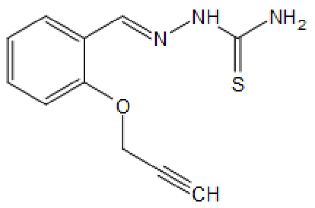

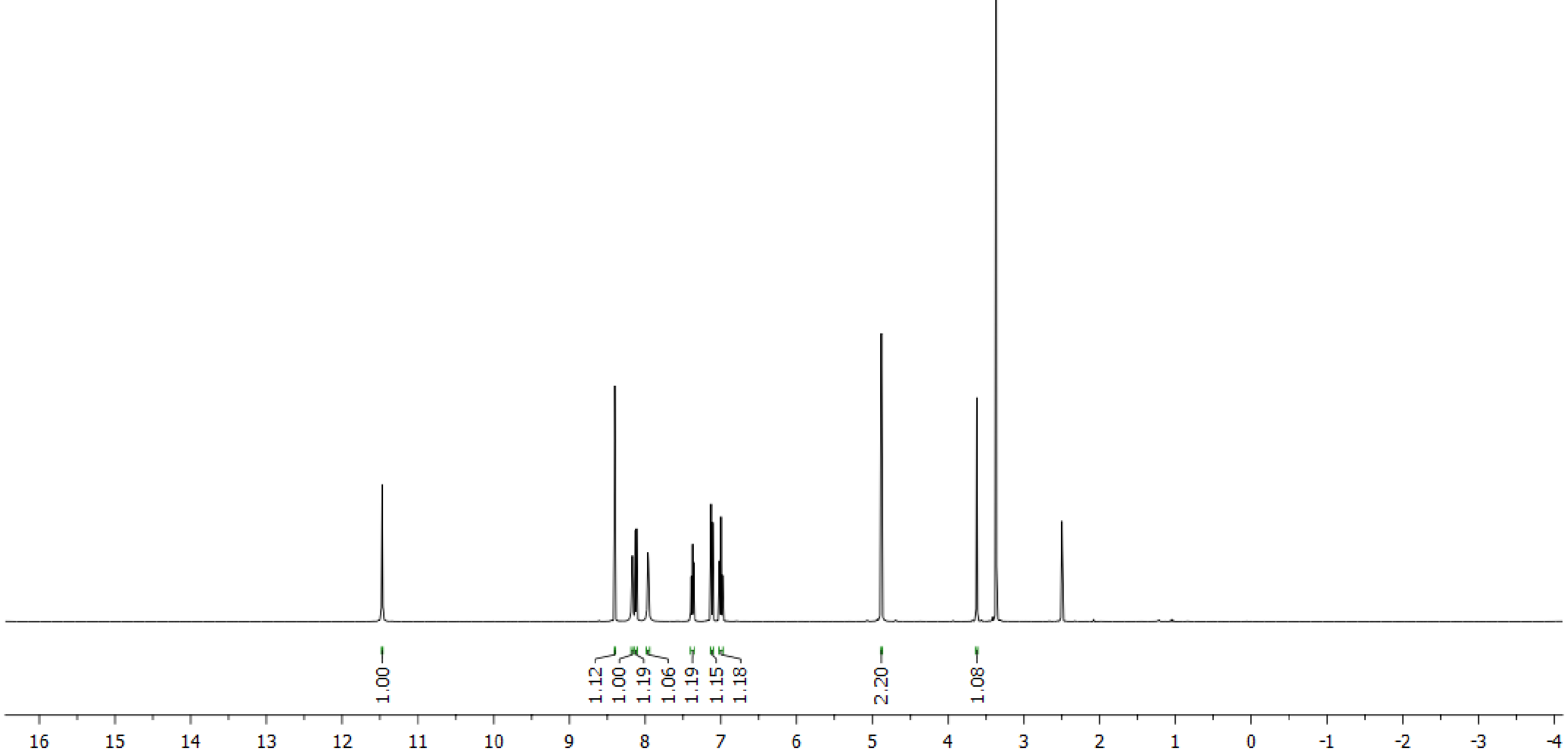

^{13}C NMR of (*E*)-2-(2-(Prop-2-yn-1-yloxy)benzylidene)hydrazine-1-carbothioamide (4)

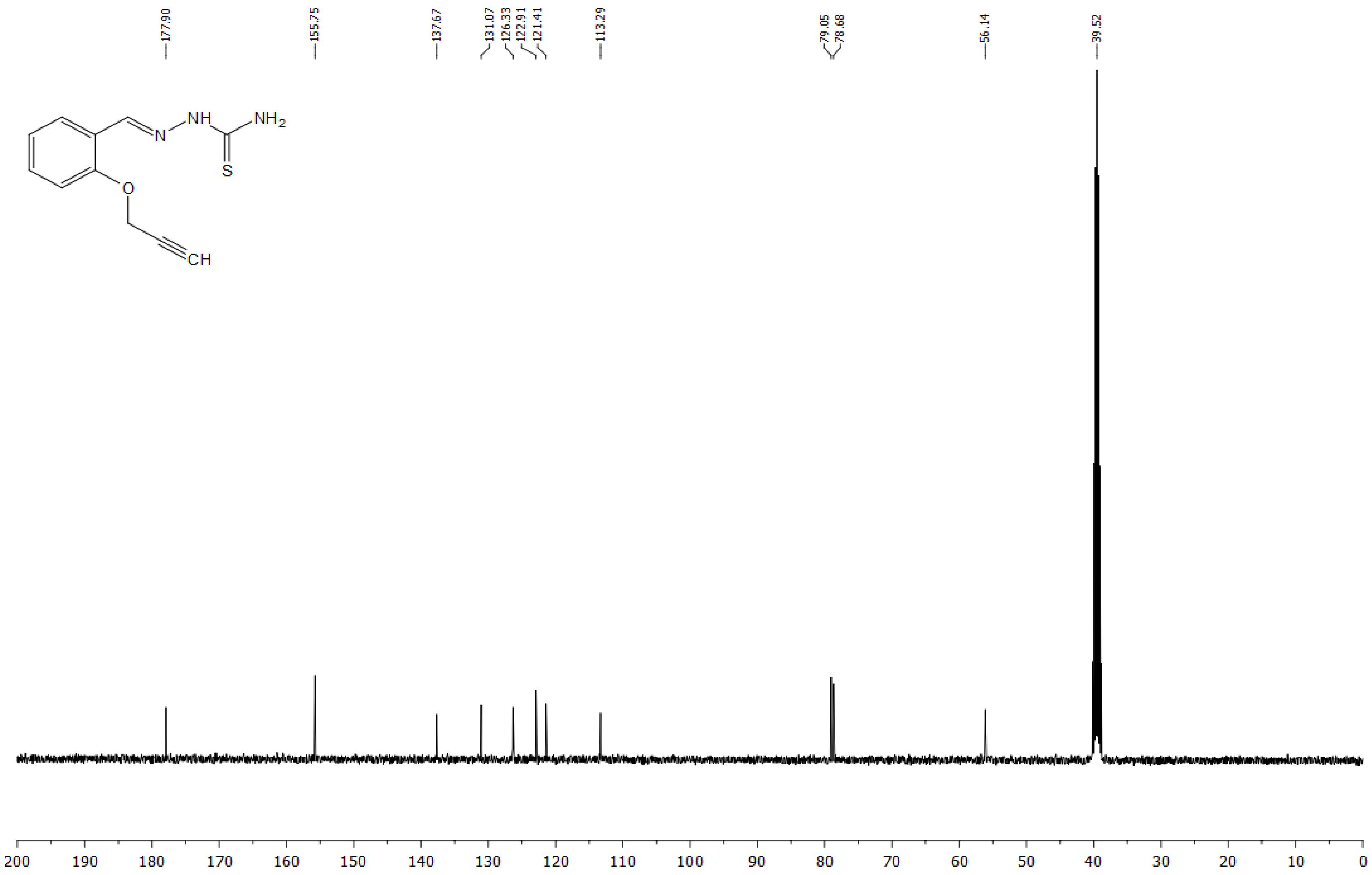

FT-IR spectrum of (*E*)-2-(2-(Prop-2-yn-1-yloxy)benzylidene)hydrazine-1-carbothioamide (4)

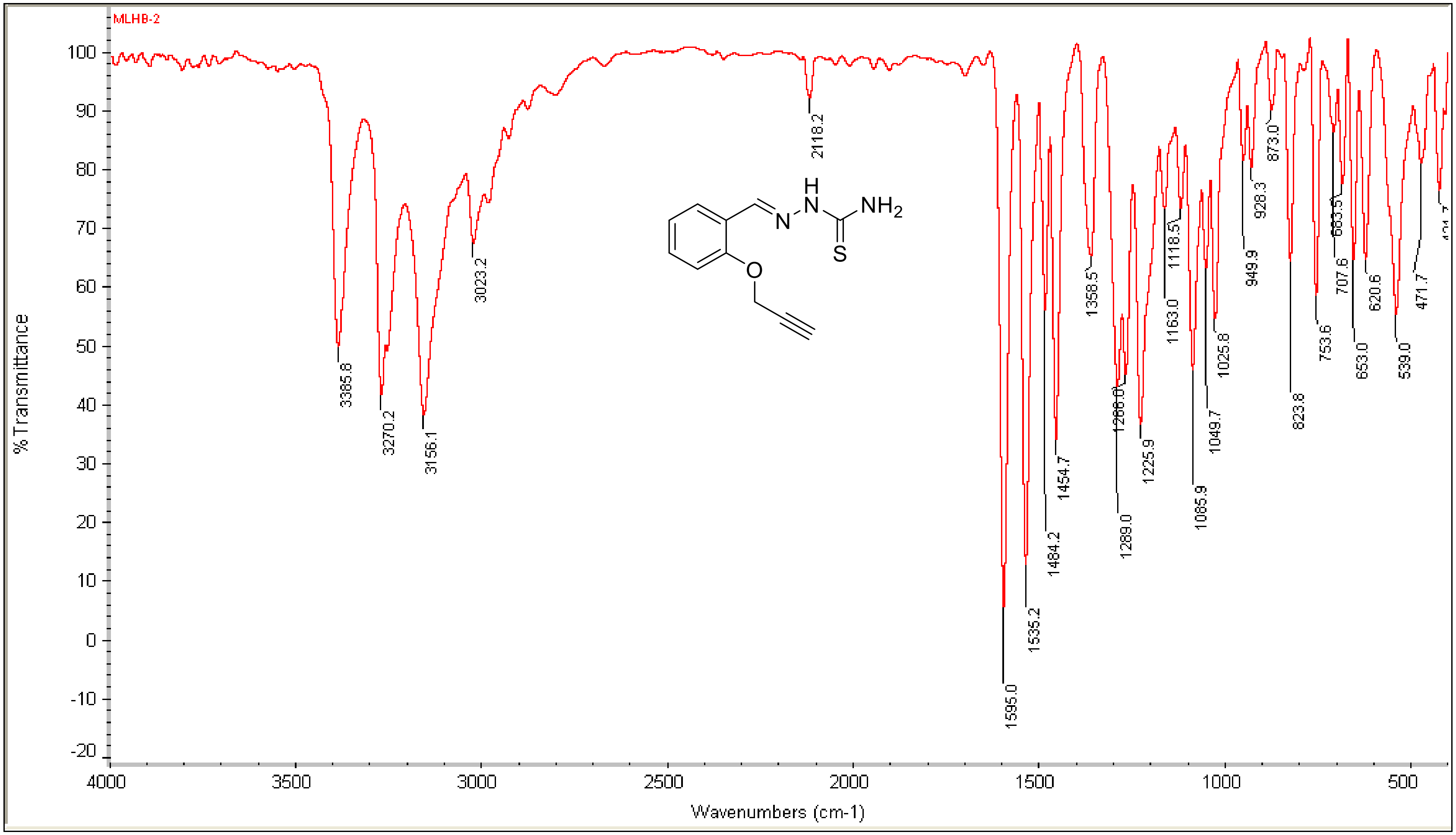

Mass spectrum of (*E*)-2-(3-Ethoxy-4-(prop-2-yn-1-yloxy)benzylidene)hydrazine-1-carbothioamide (5)

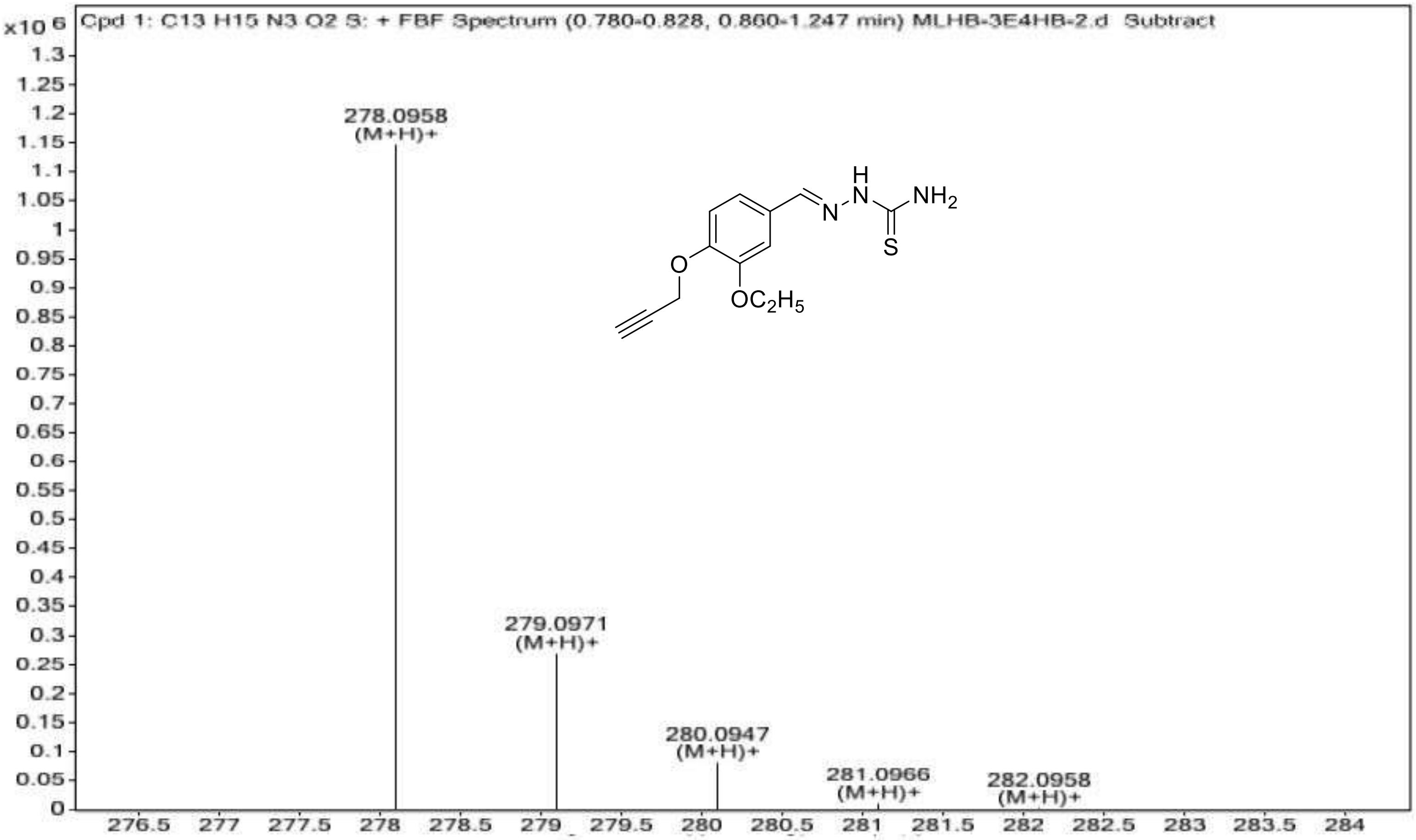

^{1}H NMR spectrum of (*E*)-2-(3-Ethoxy-4-(prop-2-yn-1-yloxy)benzylidene)hydrazine-1-carbothioamide (5)

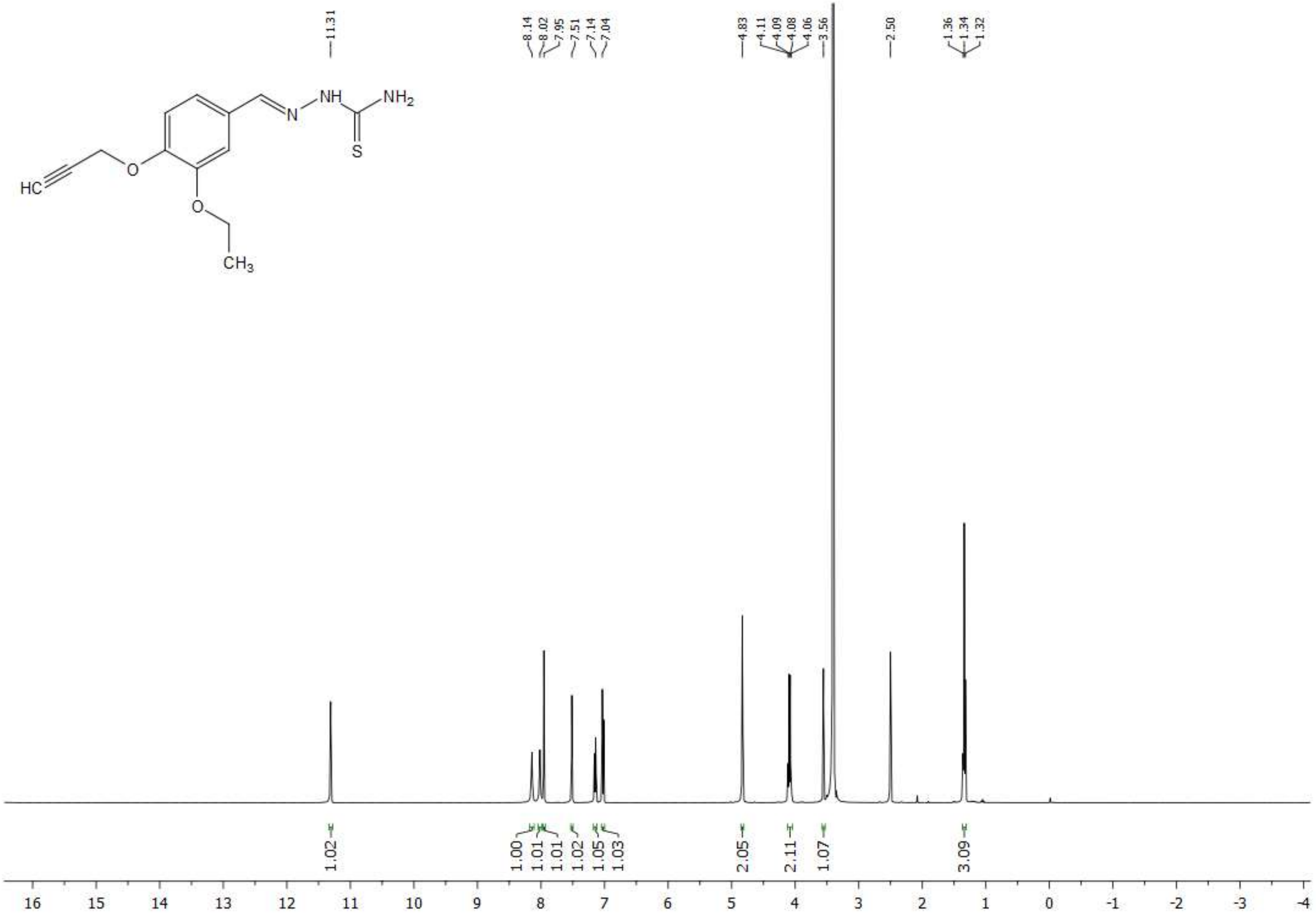

^{13}C NMR spectrum of (*E*)-2-(3-Ethoxy-4-(prop-2-yn-1-yloxy)benzylidene)hydrazine-1-carbothioamide (5)

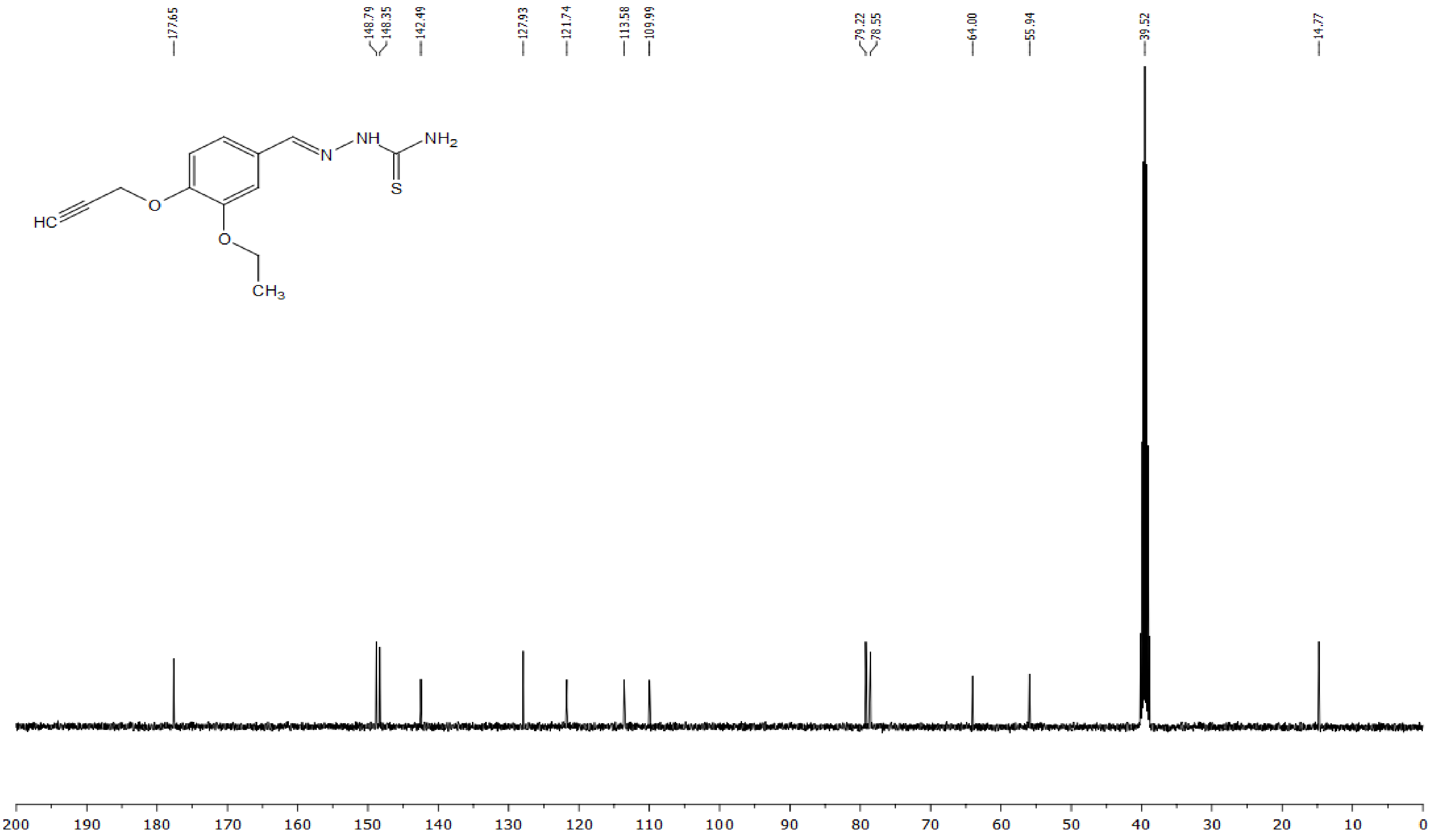

FT-IR spectrum of (*E*)-2-(3-Ethoxy-4-(prop-2-yn-1-yloxy)benzylidene)hydrazine-1-carbothioamide (5)

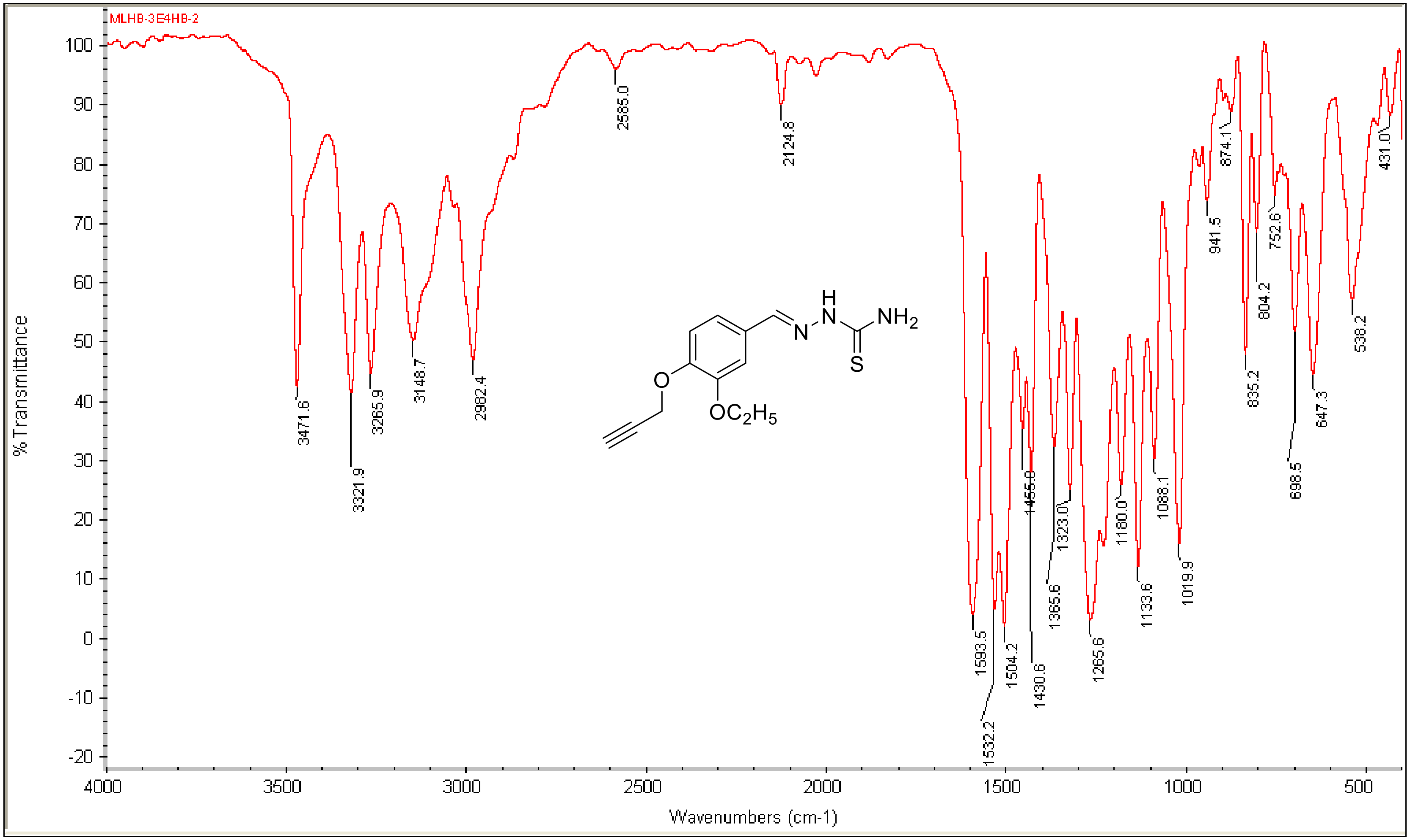

Mass spectrum of (*E*)-2-(5-Bromo-2-(prop-2-yn-1-yloxy)benzylidene)hydrazine-1-carbothioamide (6)

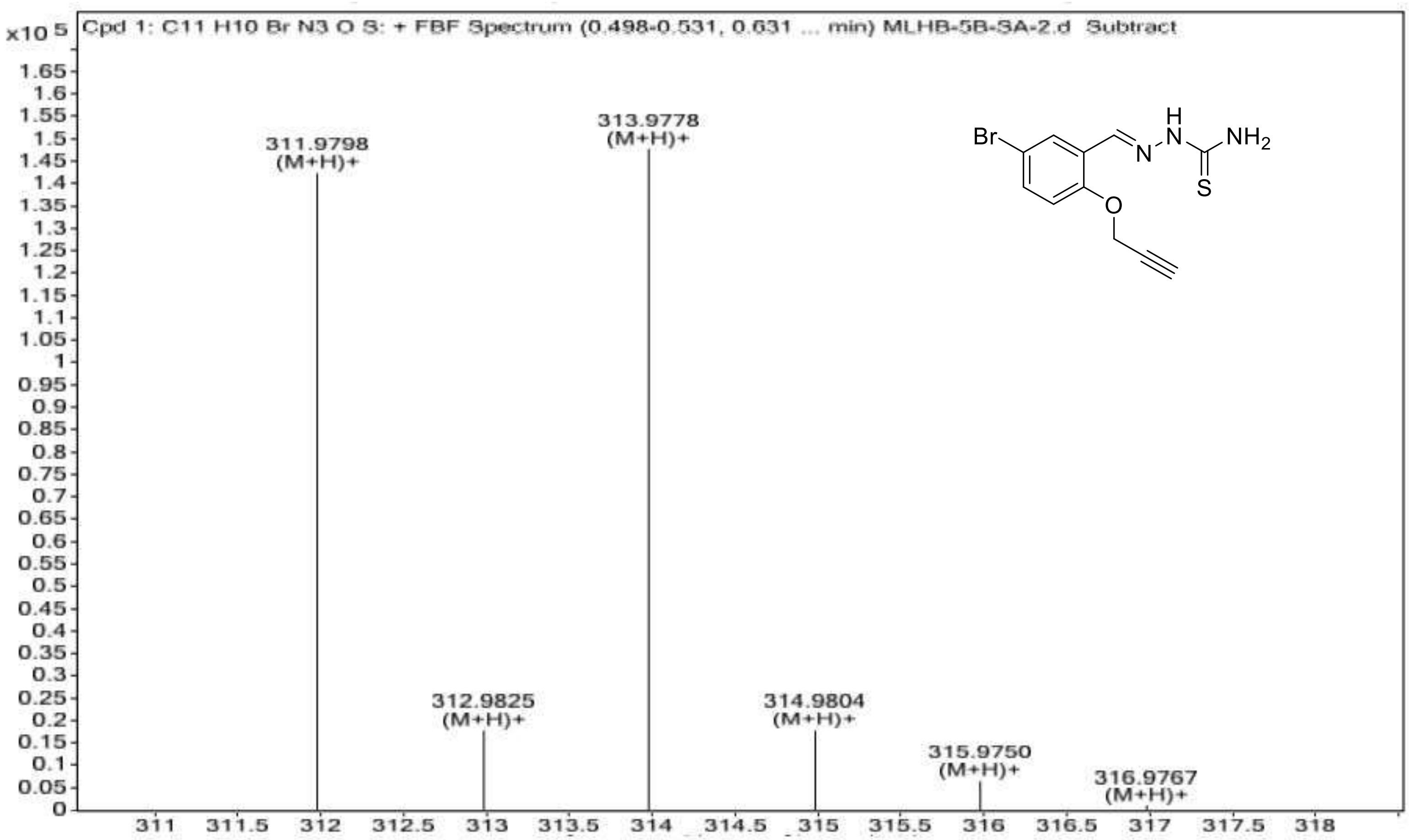

^{1}H NMR spectrum of (*E*)-2-(5-Bromo-2-(prop-2-yn-1-yloxy)benzylidene)hydrazine-1-carbothioamide (6)

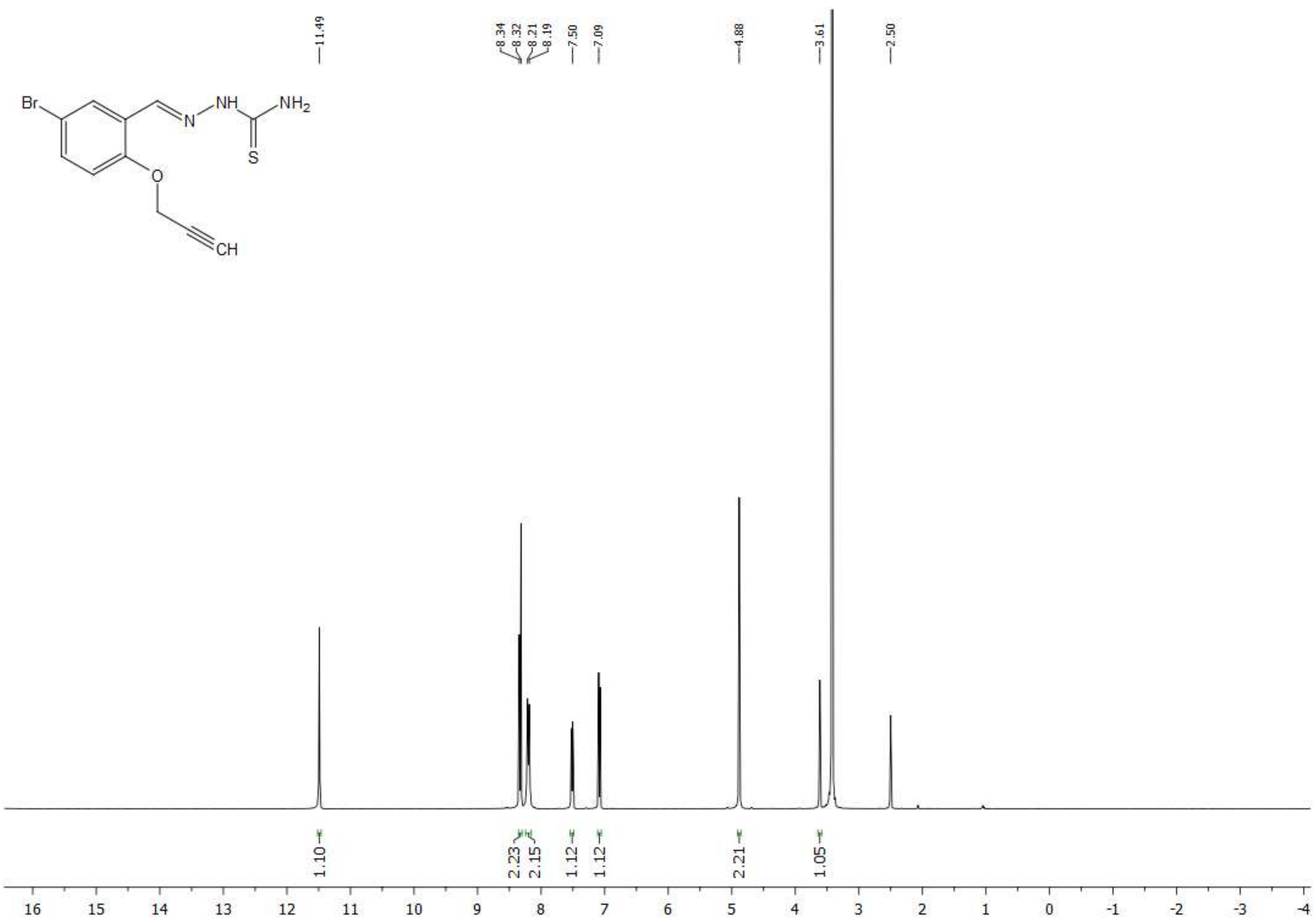

^{13}C NMR spectrum of (*E*)-2-(5-Bromo-2-(prop-2-yn-1-yloxy)benzylidene)hydrazine-1-carbothioamide (6)

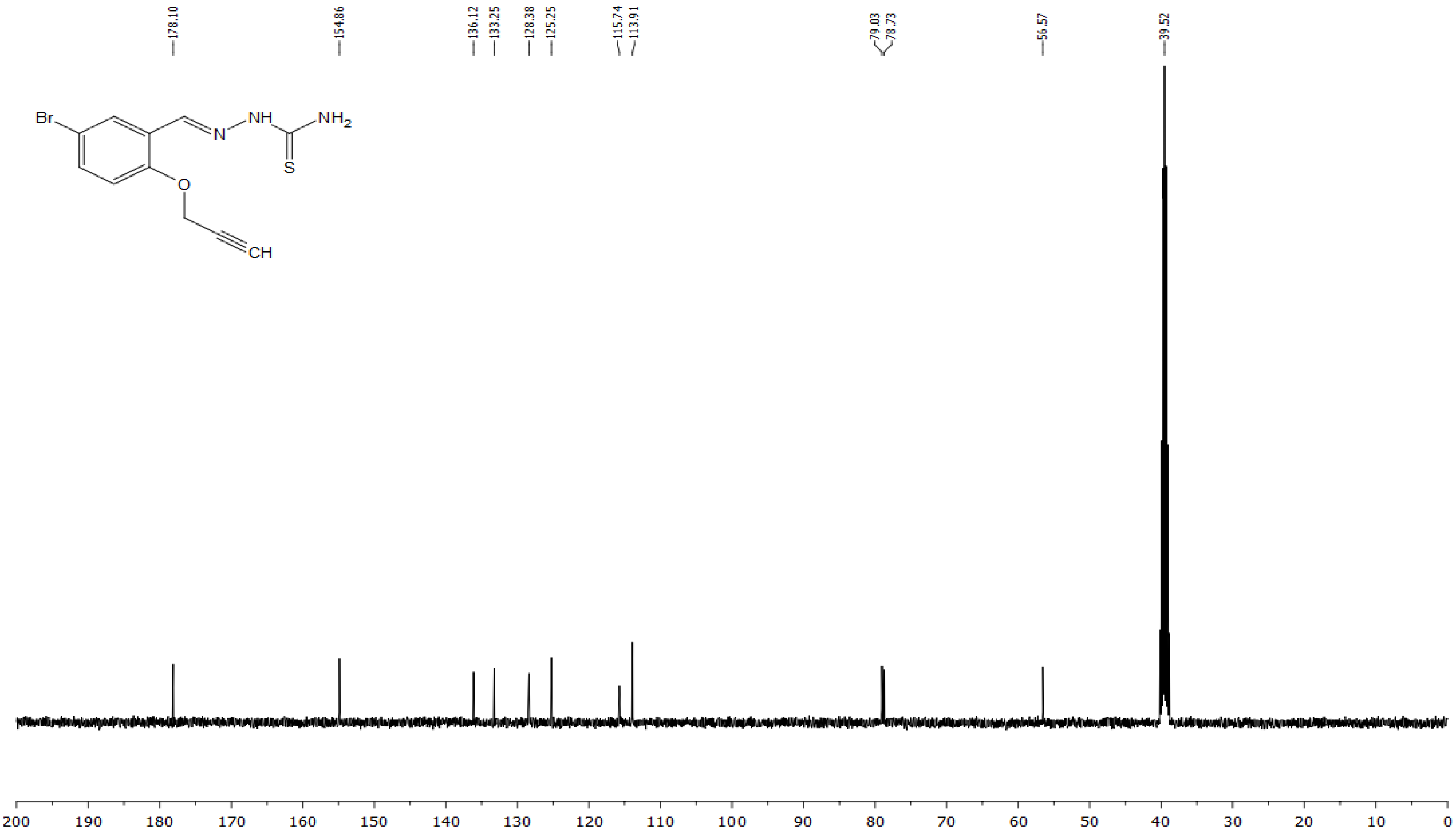

FT-IR spectrum of (*E*)-2-(5-Bromo-2-(prop-2-yn-1-yloxy)benzylidene)hydrazine-1-carbothioamide (6)

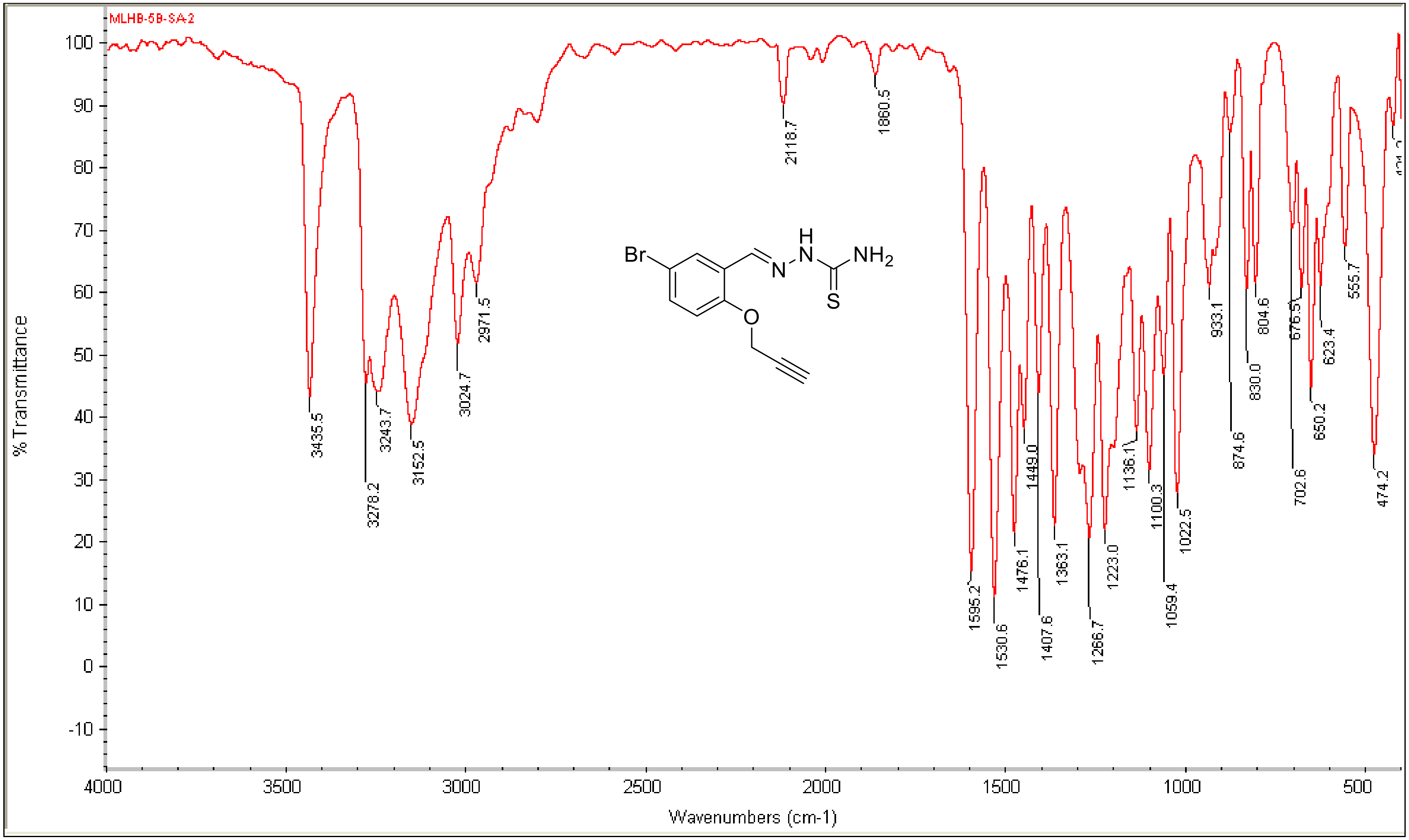

Mass spectrum of (*E*)-4-Phenyl-2-(2-(2-(prop-2-yn-1-yloxy)benzylidene)hydrazinyl)thiazole (7)

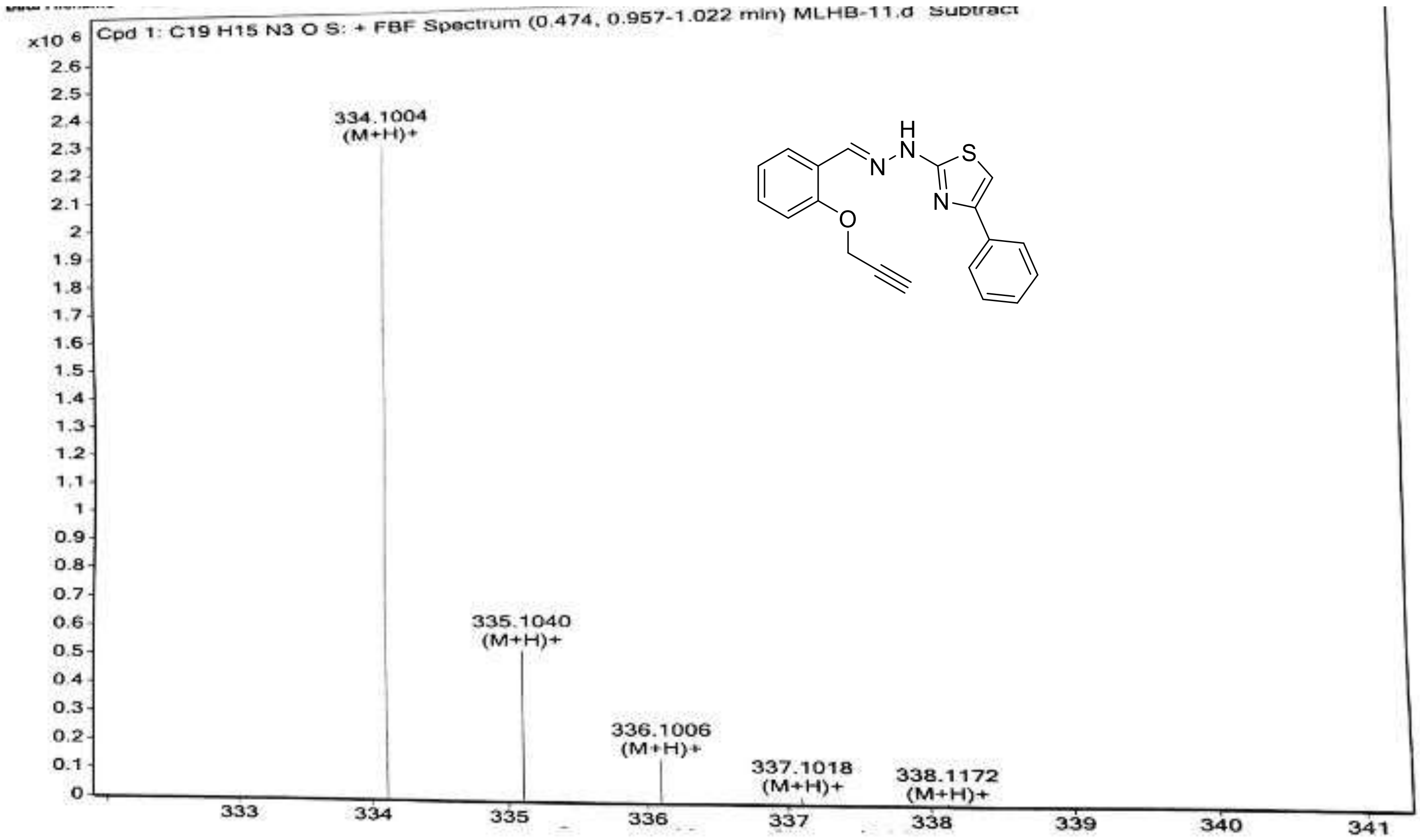

^{1}H NMR spectrum of (*E*)-4-Phenyl-2-(2-(2-(prop-2-yn-1-yloxy)benzylidene)hydrazinyl)thiazole (7)

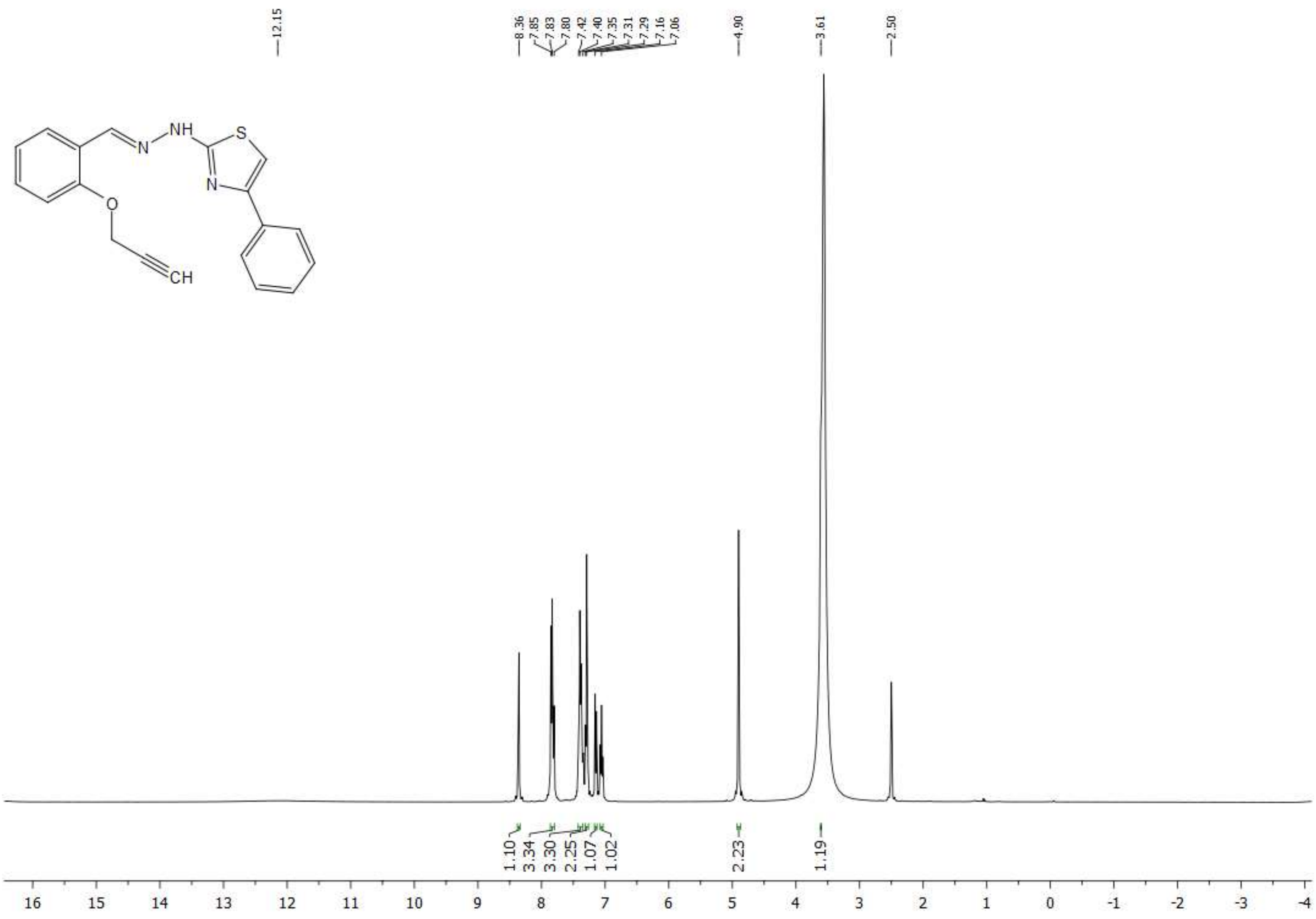

^{13}C NMR spectrum of (*E*)-4-Phenyl-2-(2-(2-(prop-2-yn-1-yloxy)benzylidene)hydrazinyl)thiazole (7)

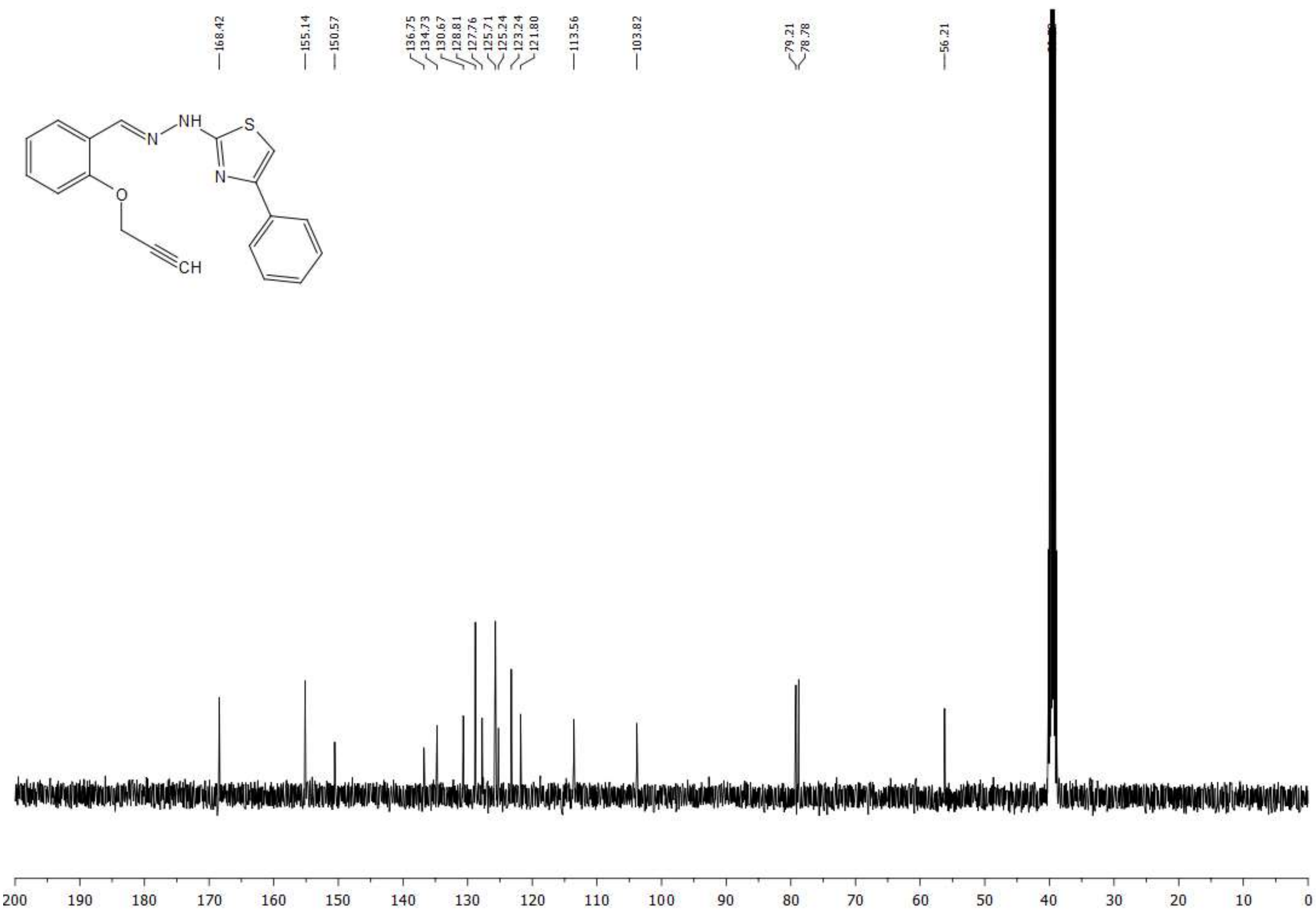

FT-IR spectrum of (*E*)-4-Phenyl-2-(2-(2-(prop-2-yn-1-yloxy)benzylidene)hydrazinyl)thiazole (7)

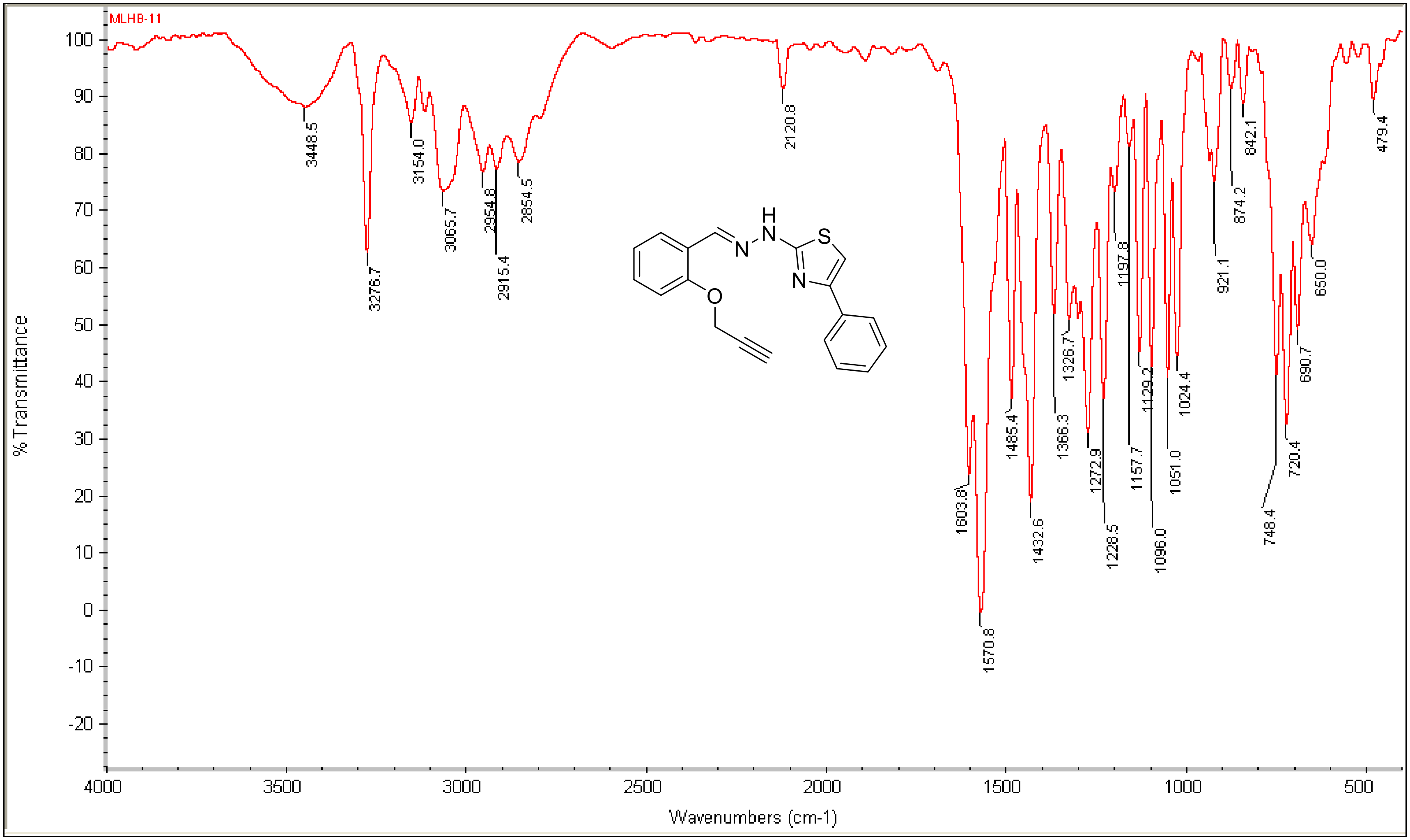

Mass spectrum of (*E*)-4-(4-Fluorophenyl)-2-(2-(2-(prop-2-yn-1-yloxy)benzylidene)hydrazinyl)thiazole (8)

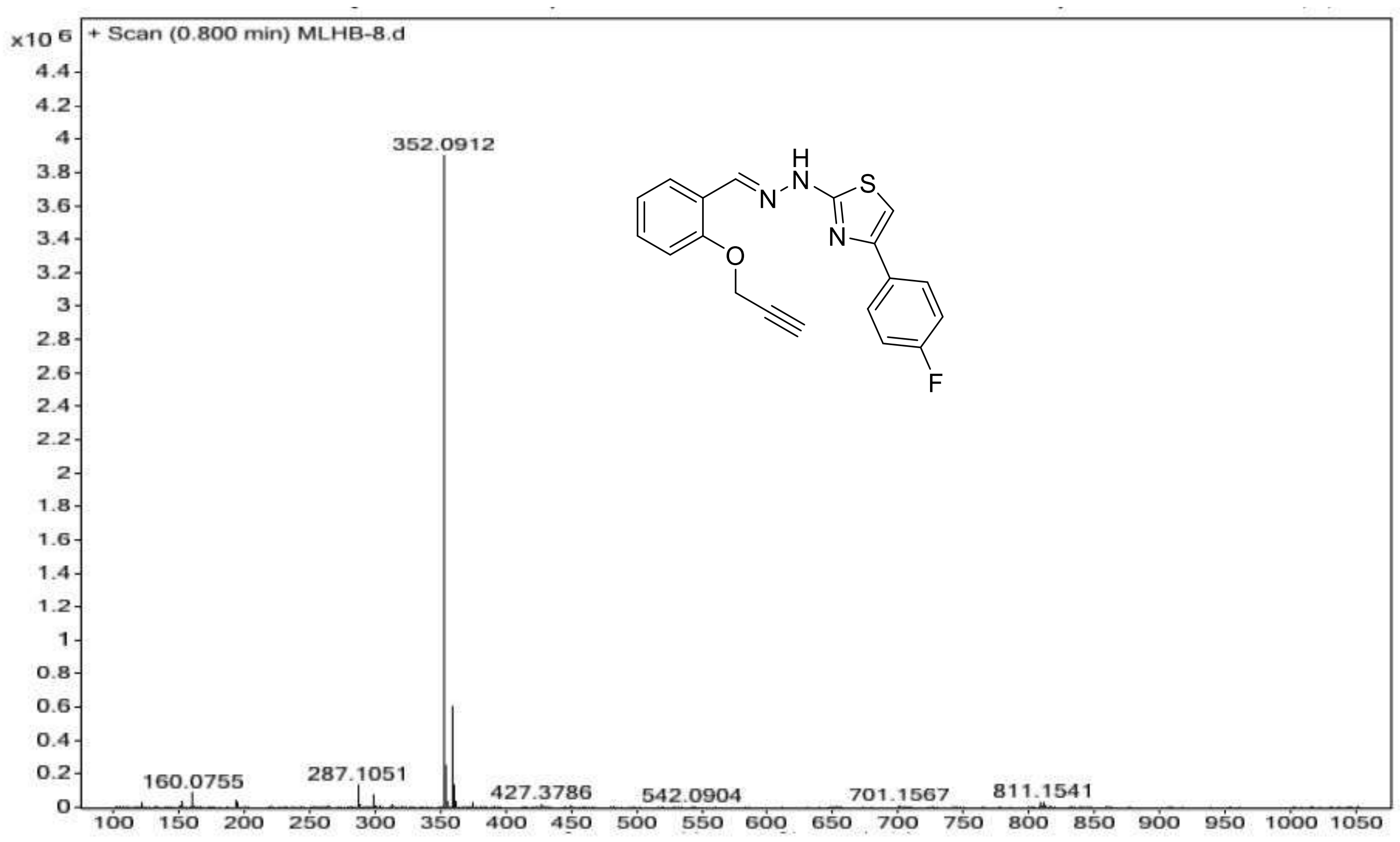

^{1}H NMR spectrum of (*E*)-4-(4-Fluorophenyl)-2-(2-(2-(prop-2-yn-1-yloxy)benzylidene)hydrazinyl)thiazole (8)

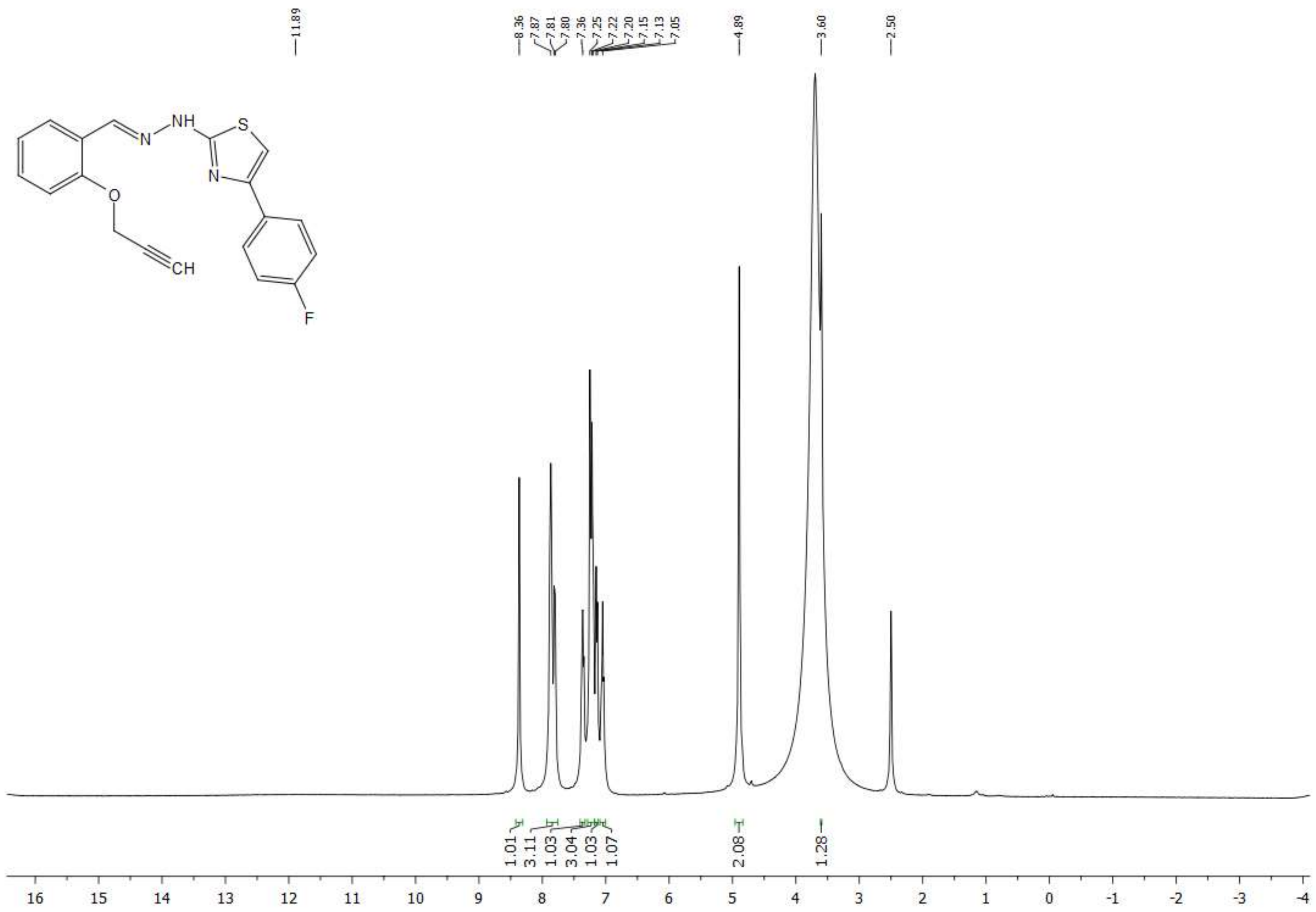

^{13}C NMR spectrum of (*E*)-4-(4-Fluorophenyl)-2-(2-(2-(prop-2-yn-1-yloxy)benzylidene)hydrazinyl)thiazole (8)

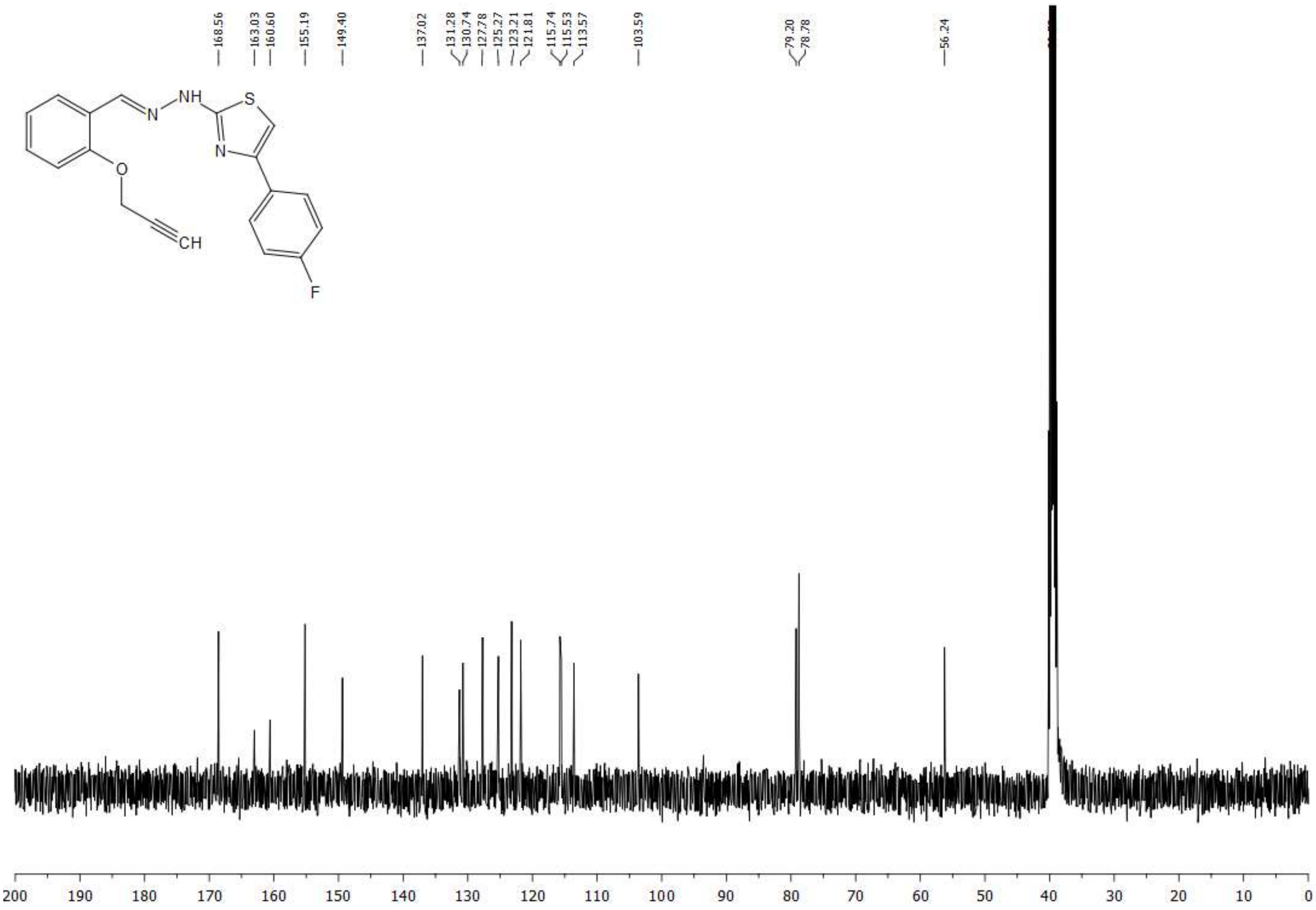

FT-IR spectrum of (*E*)-4-(4-Fluorophenyl)-2-(2-(2-(prop-2-yn-1-yloxy)benzylidene)hydrazinyl)thiazole (8)

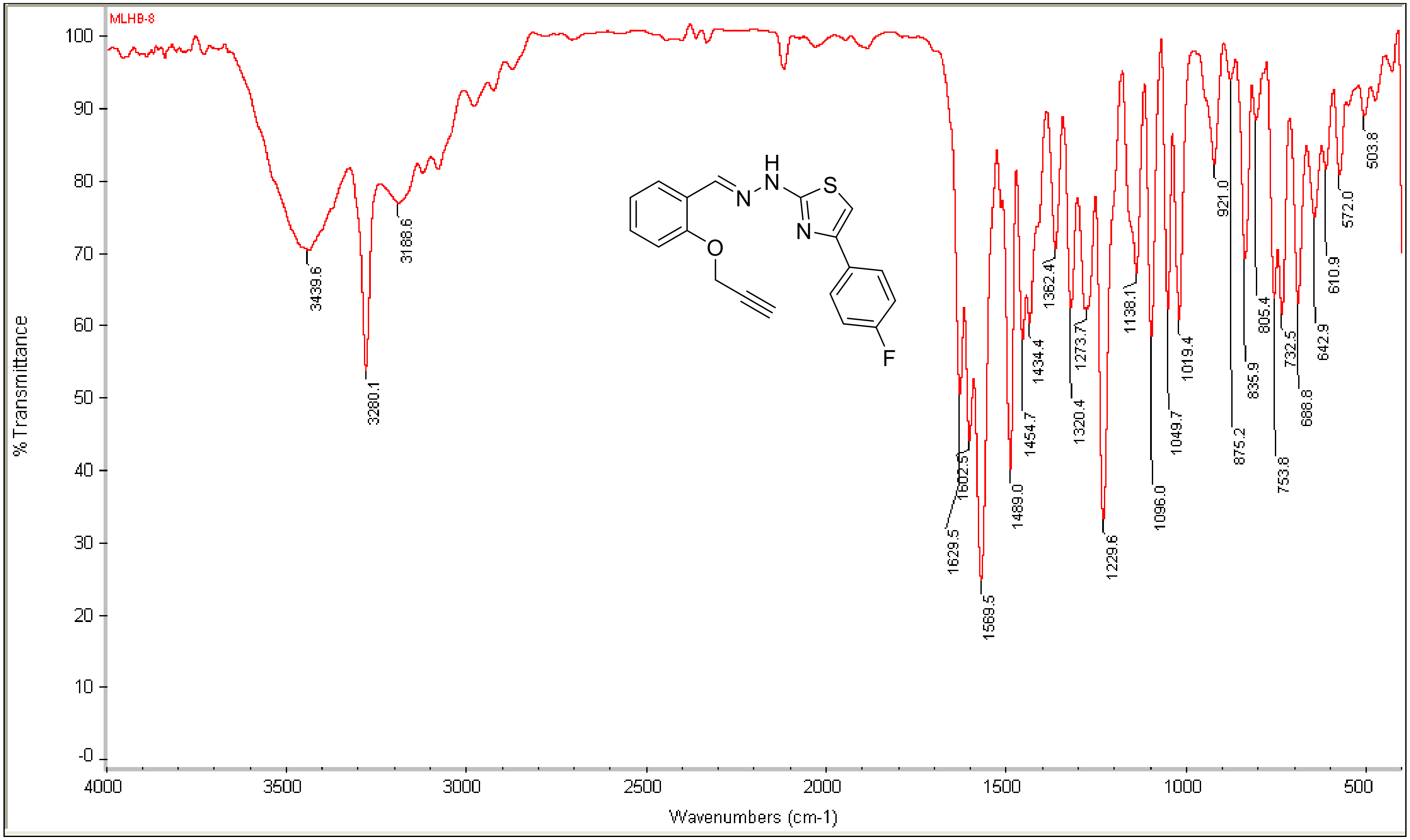

Mass spectrum of (*E*)-4-(4-Chlorophenyl)-2-(2-(2-(prop-2-yn-1-yloxy)benzylidene)hydrazinyl)thiazole (9)

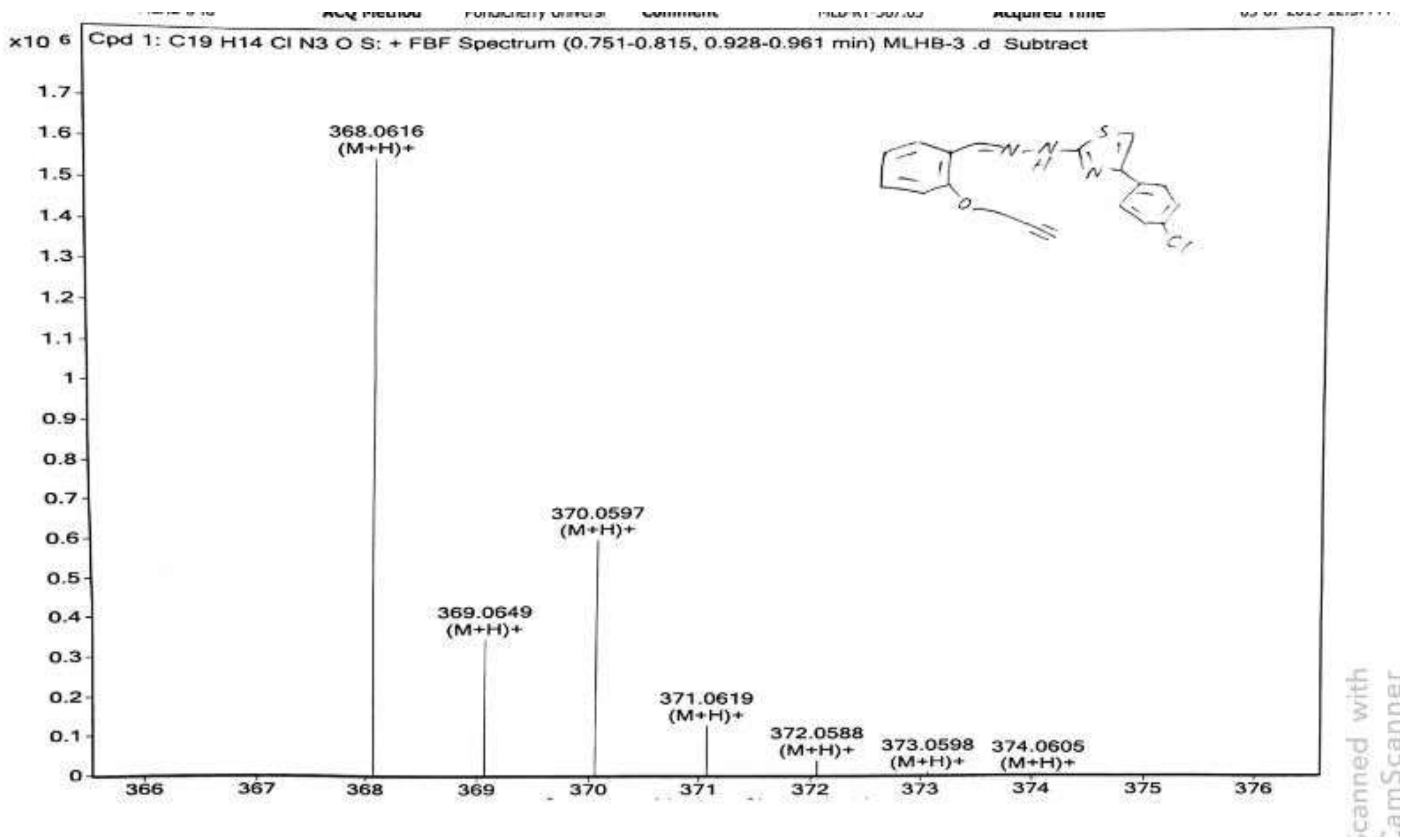

¹H NMR spectrum of (*E*)-4-(4-Chlorophenyl)-2-(2-(2-(prop-2-yn-1-yloxy)benzylidene)hydrazinyl)thiazole (9)

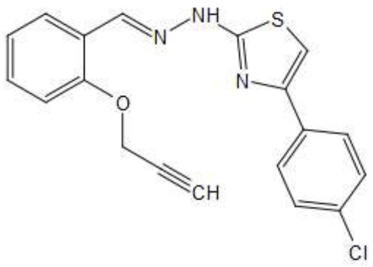

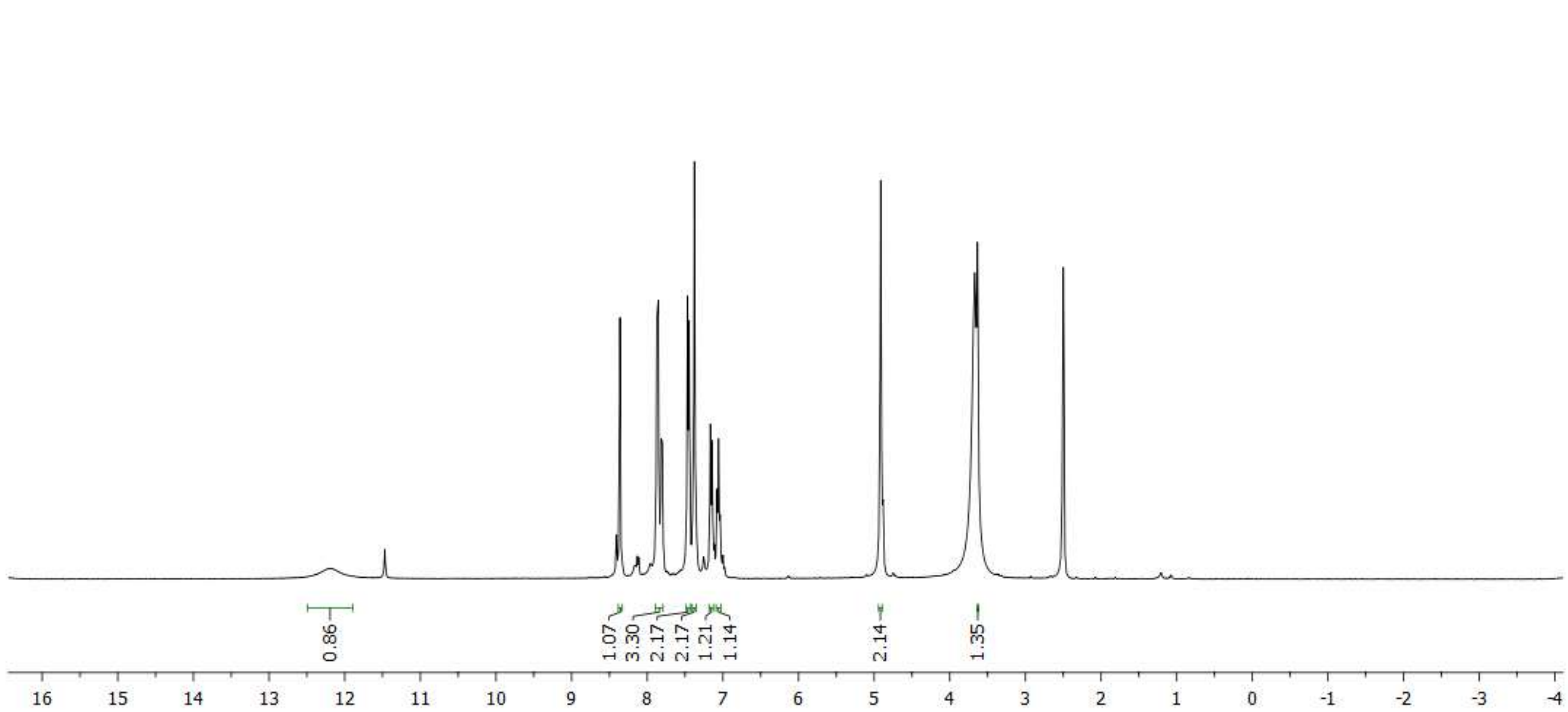

^{13}C NMR spectrum of (*E*)-4-(4-Chlorophenyl)-2-(2-(2-(prop-2-yn-1-yloxy)benzylidene)hydrazinyl)thiazole (9)

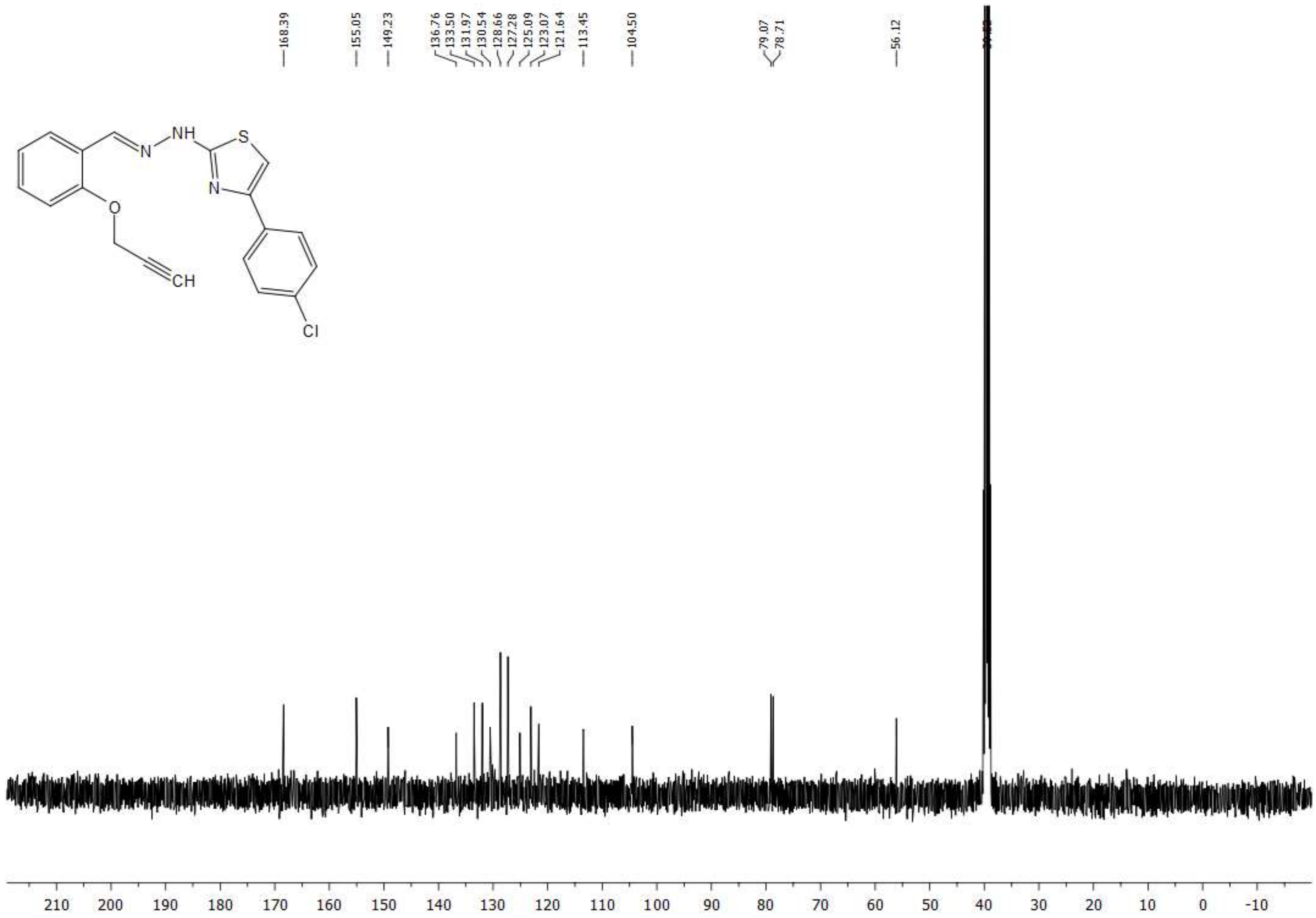

FT-IR spectrum of (*E*)-4-(4-Chlorophenyl)-2-(2-(2-(prop-2-yn-1-yloxy)benzylidene)hydrazinyl)thiazole (9)

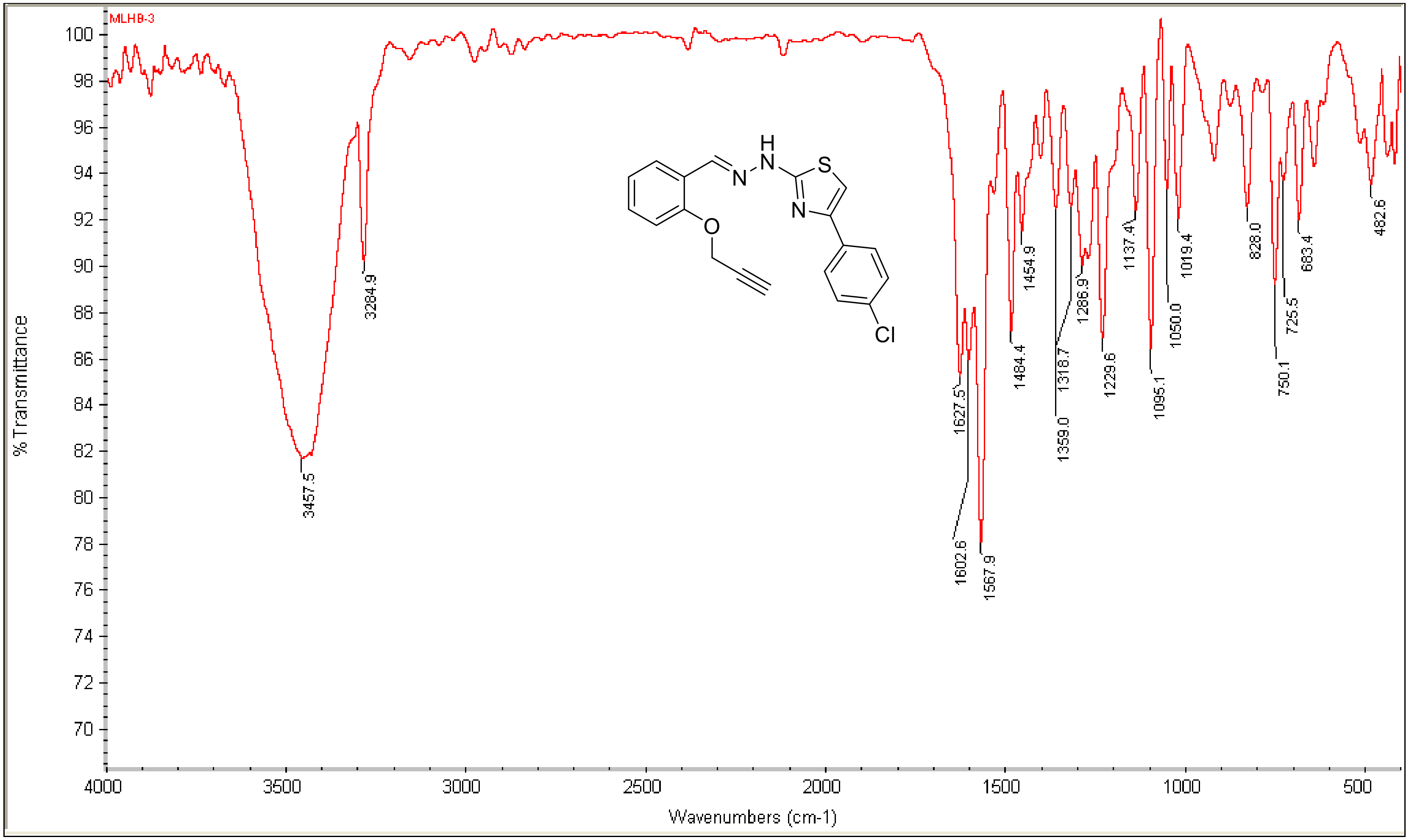

Mass spectrum of (*E*)-4-(3,4-Dichlorophenyl)-2-(2-(2-(prop-2-yn-1-yloxy)benzylidene)hydrazinyl)thiazole (10)

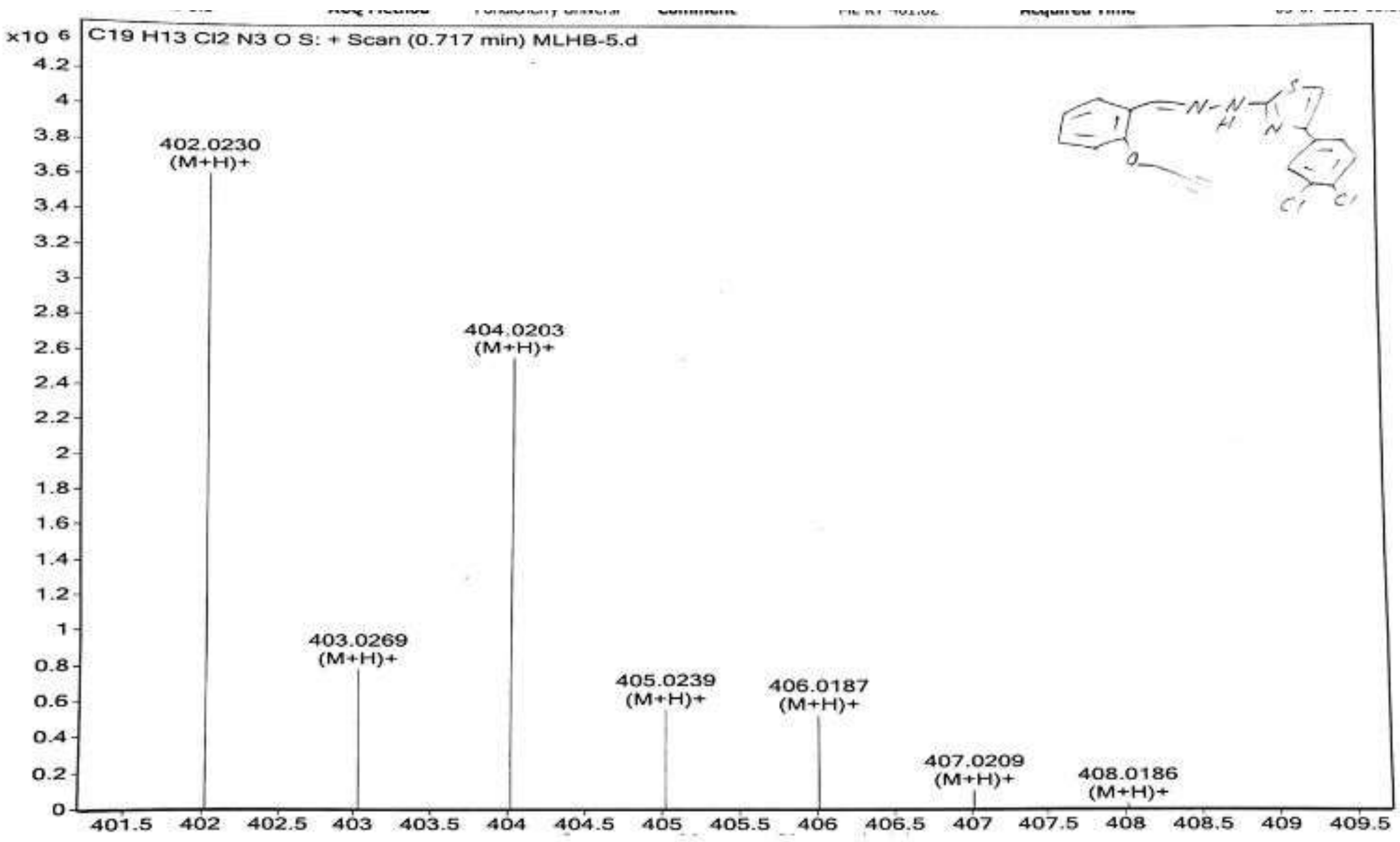

Scanned with CamScanner

^{1}H NMR spectrum of (*E*)-4-(3,4-Dichlorophenyl)-2-(2-(2-(prop-2-yn-1-yloxy)benzylidene)hydrazinyl)thiazole (10)

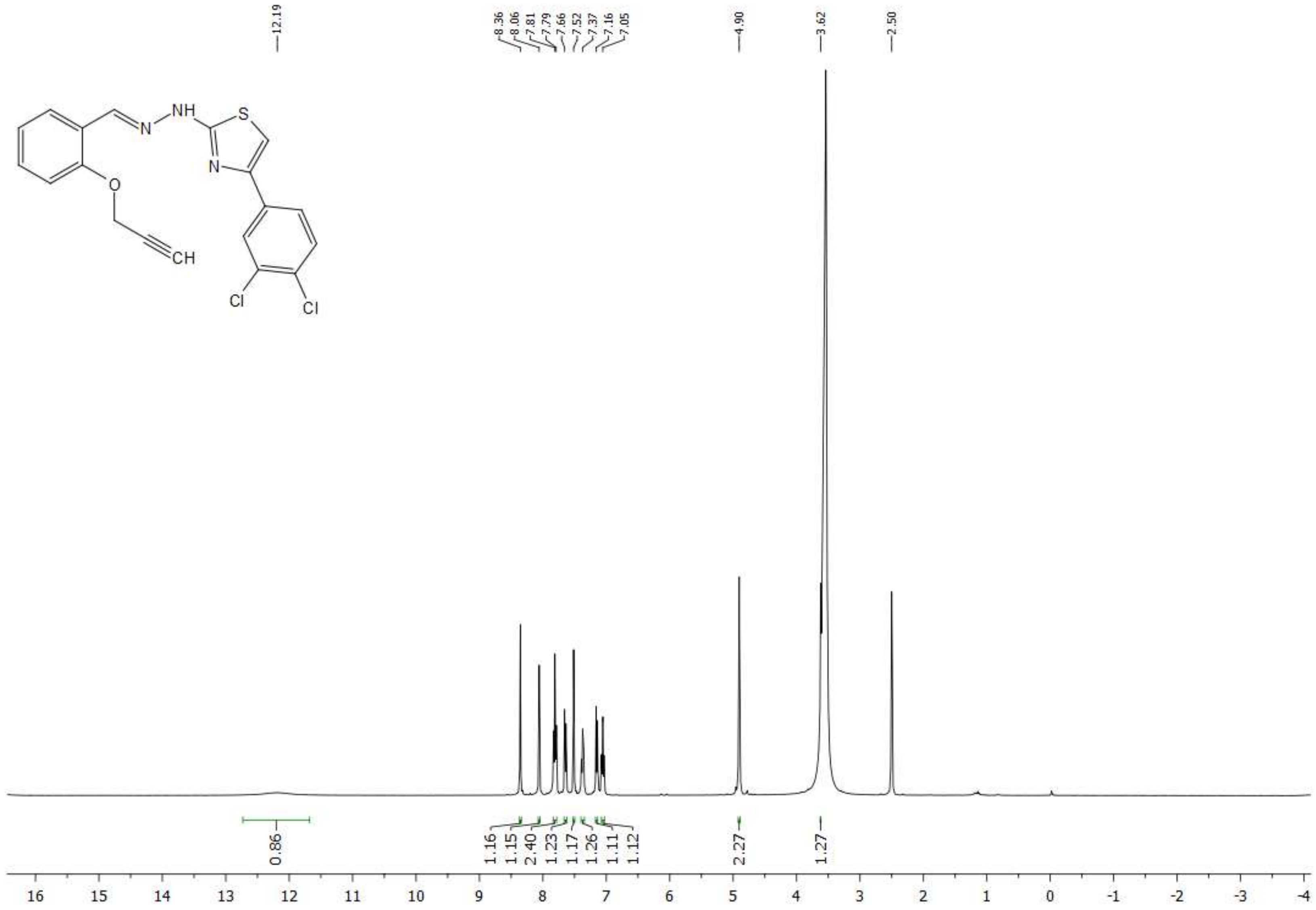

^{13}C NMR spectrum of (*E*)-4-(3,4-Dichlorophenyl)-2-(2-(2-(prop-2-yn-1-yloxy)benzylidene)hydrazinyl)thiazole (10)

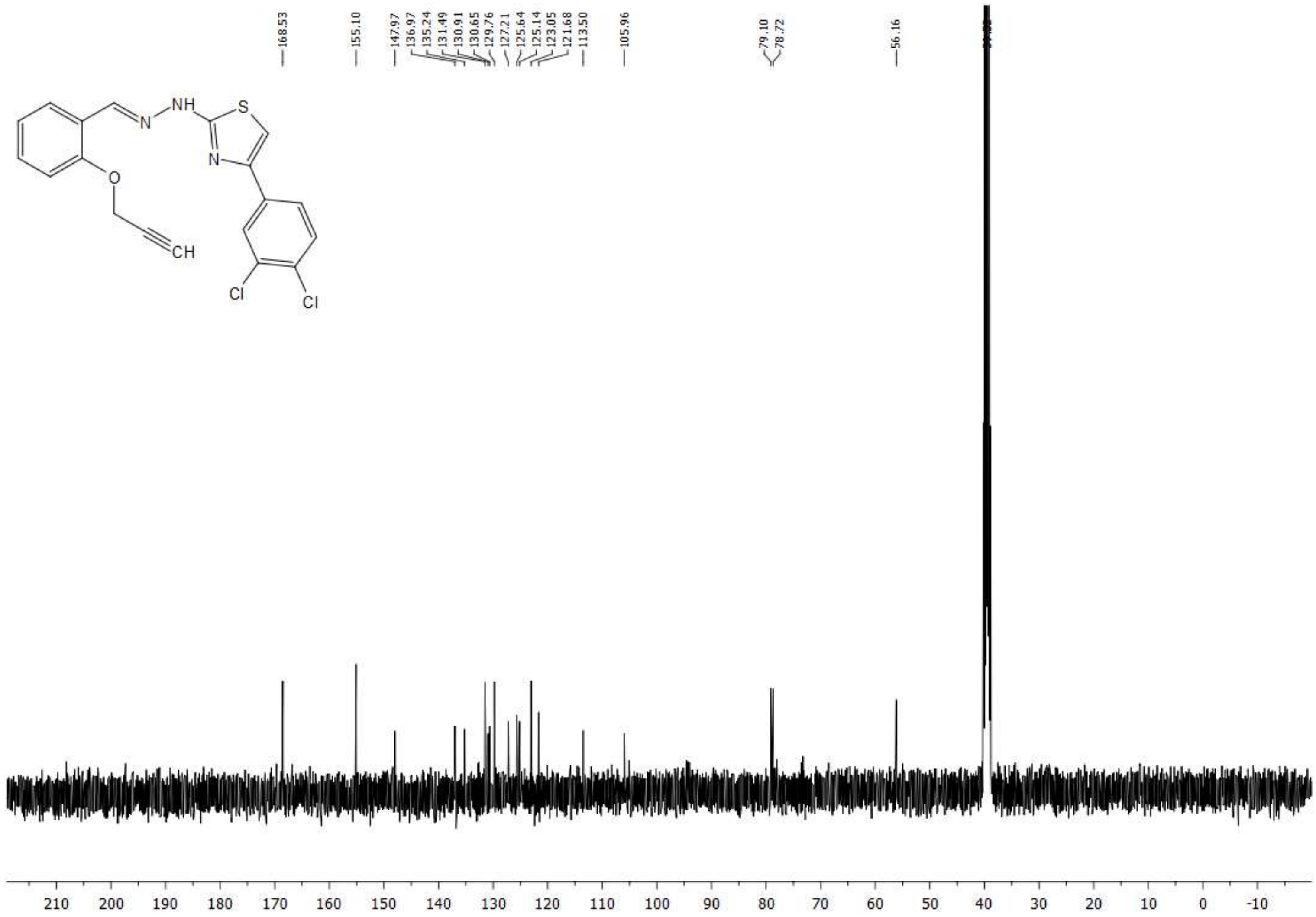

FT-IR spectrum of (*E*)-4-(3,4-Dichlorophenyl)-2-(2-(2-(prop-2-yn-1-yloxy)benzylidene)hydrazinyl)thiazole (10)

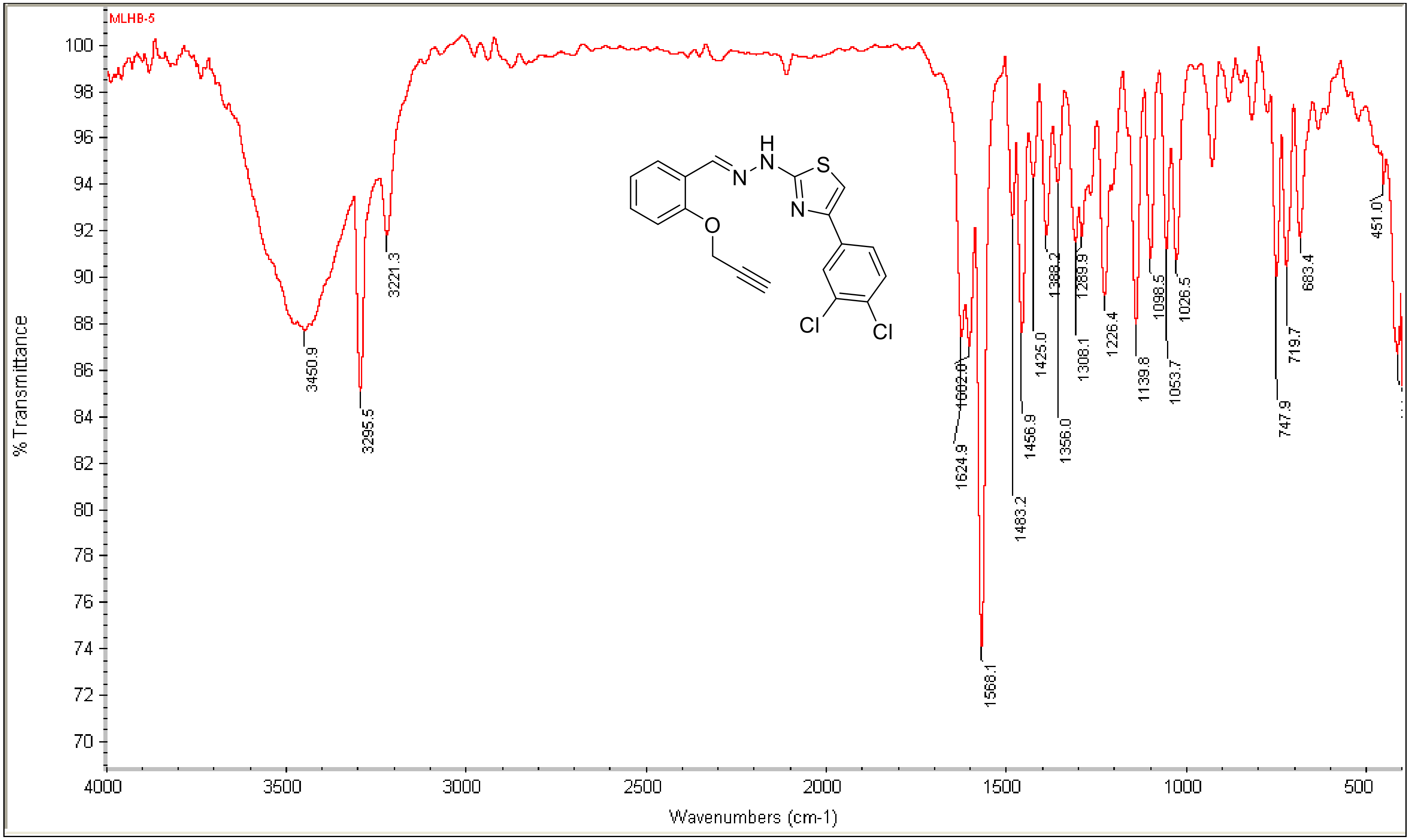

Mass spectrum of (*E*)-4-(4-Bromophenyl)-2-(2-(2-(prop-2-yn-1-yloxy)benzylidene)hydrazinyl)thiazole (11)

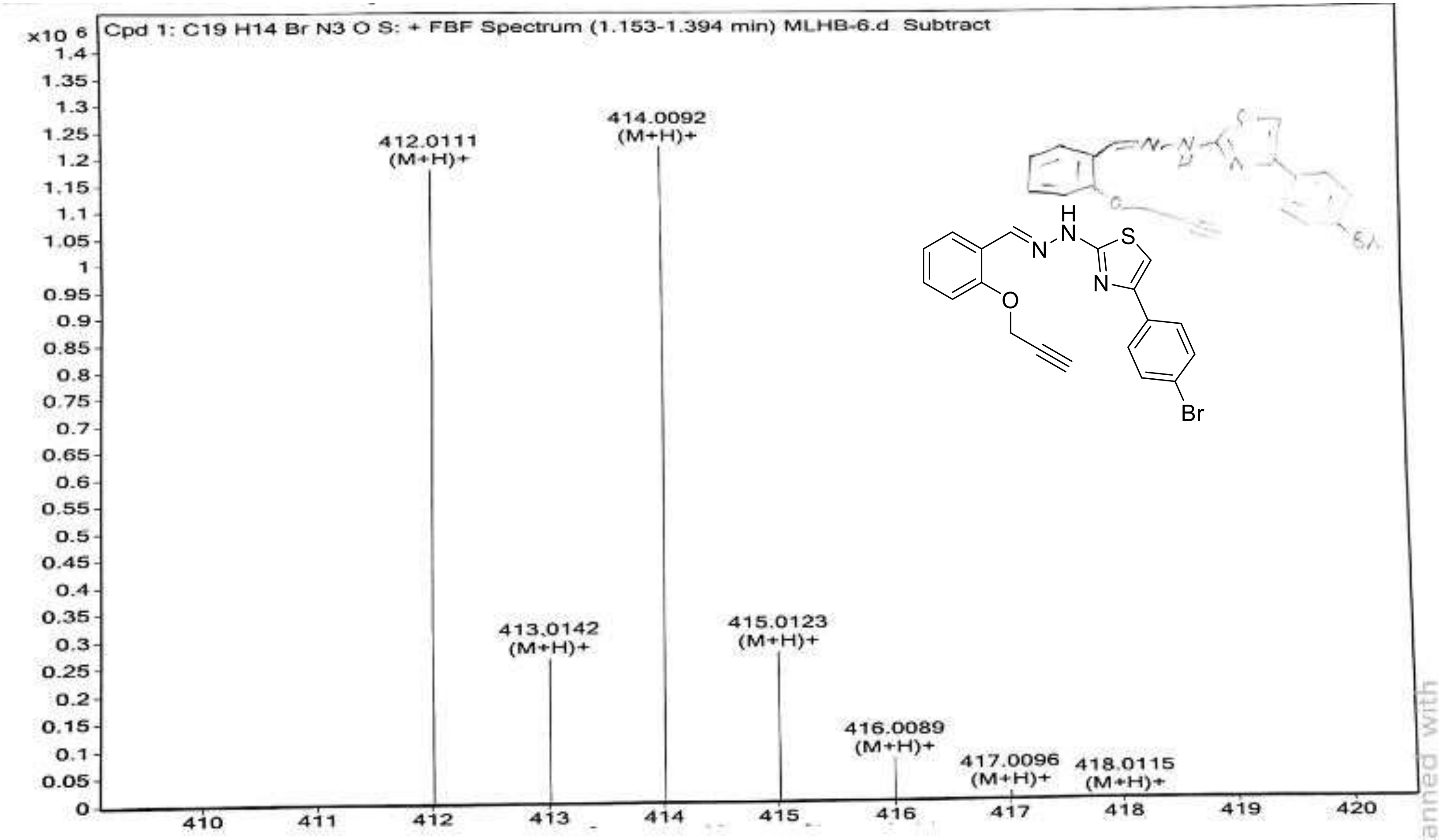

Scanned with CamScanner

^{1}H NMR spectrum of (*E*)-4-(4-Bromophenyl)-2-(2-(2-(prop-2-yn-1-yloxy)benzylidene)hydrazinyl)thiazole (11)

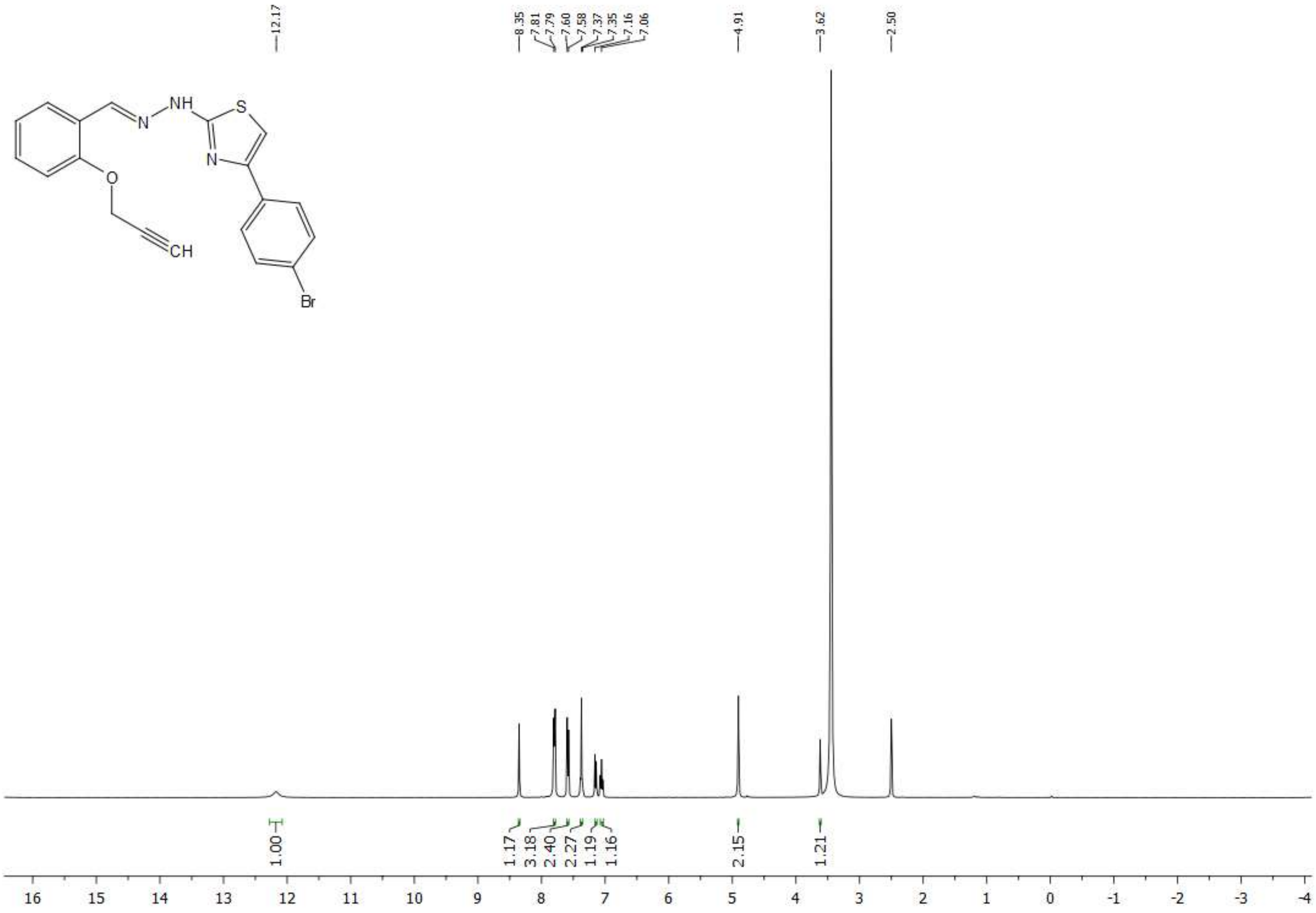

^{13}C NMR spectrum of (*E*)-4-(4-Bromophenyl)-2-(2-(2-(prop-2-yn-1-yloxy)benzylidene)hydrazinyl)thiazole (11)

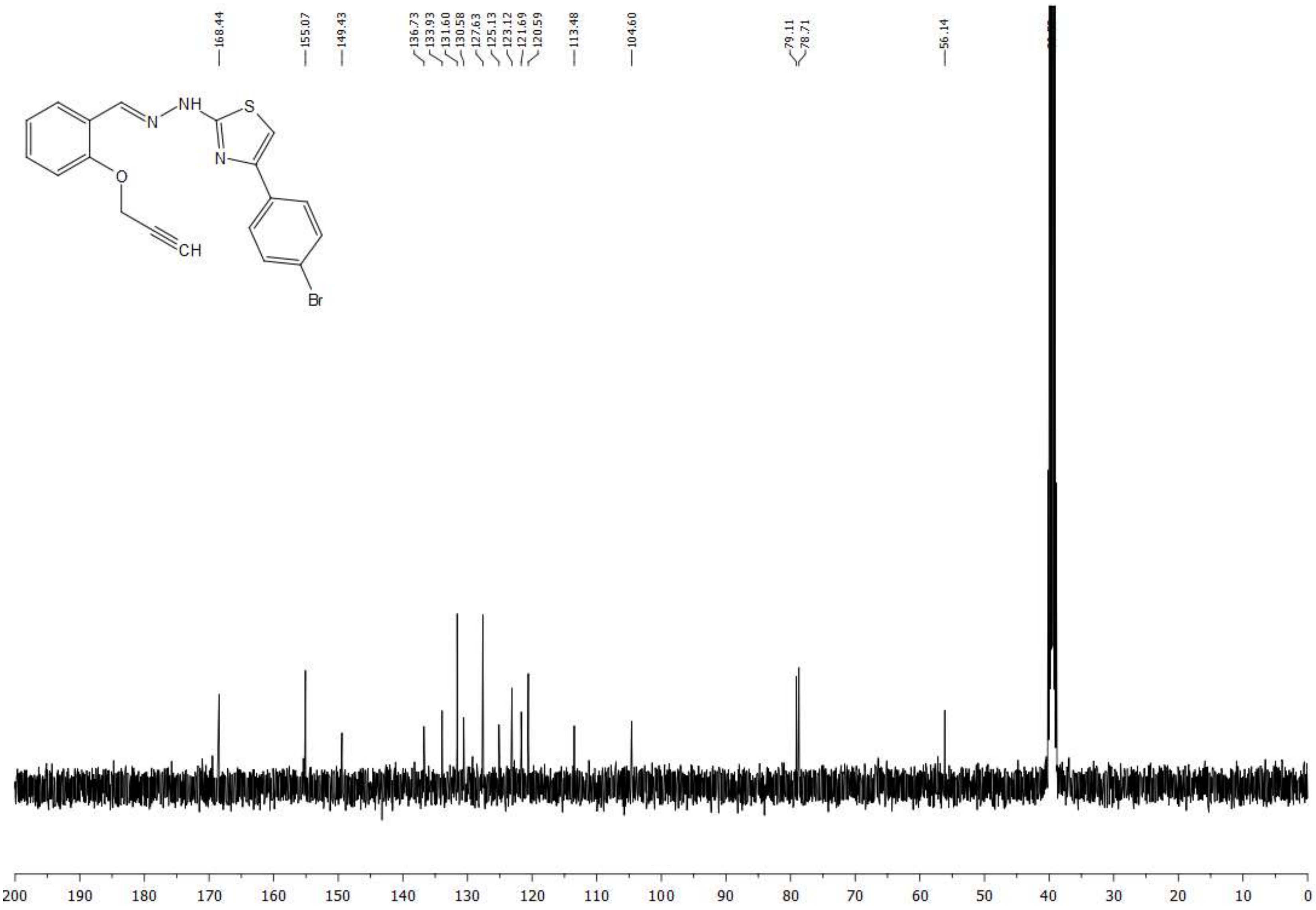

FT-IR spectrum of (*E*)-4-(4-Bromophenyl)-2-(2-(2-(prop-2-yn-1-yloxy)benzylidene)hydrazinyl)thiazole (11)

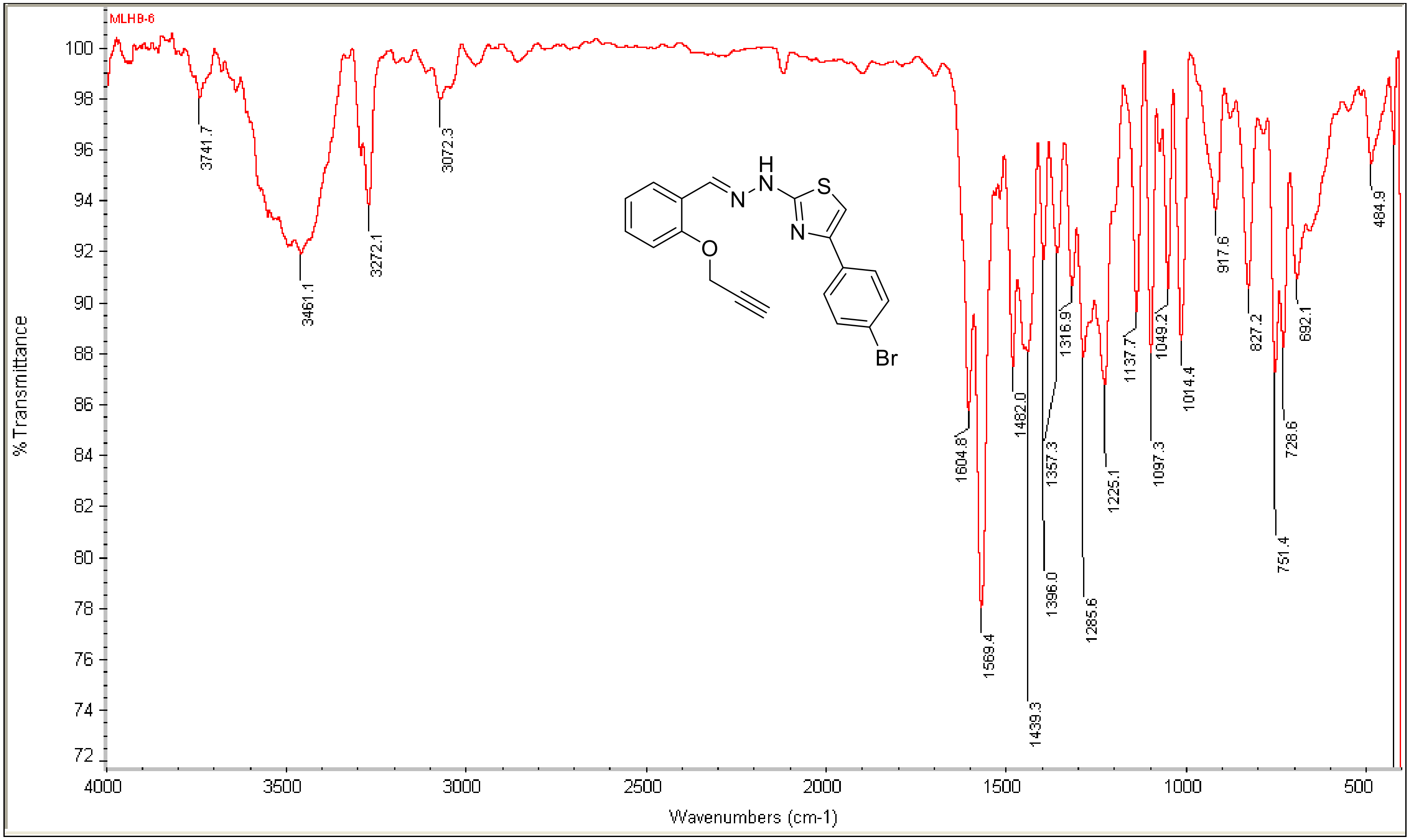

Mass spectrum of (*E*)-4-(4-Nitrophenyl)-2-(2-(2-(prop-2-yn-1-yloxy)benzylidene)hydrazinyl)thiazole (12)

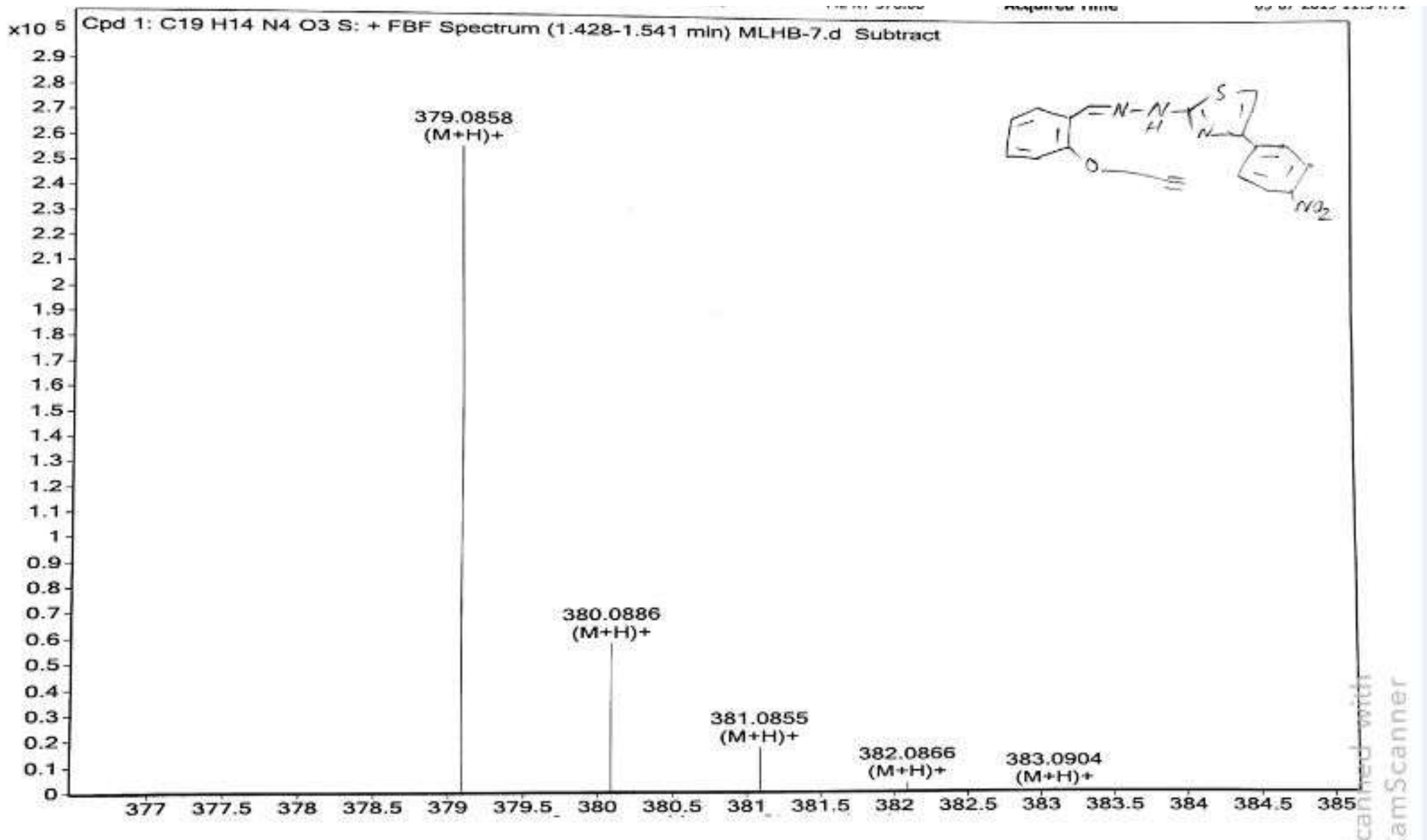

Scanned with CamScanner

^{1}H NMR spectrum of (*E*)-4-(4-Nitrophenyl)-2-(2-(2-(prop-2-yn-1-yloxy)benzylidene)hydrazinyl)thiazole (12)

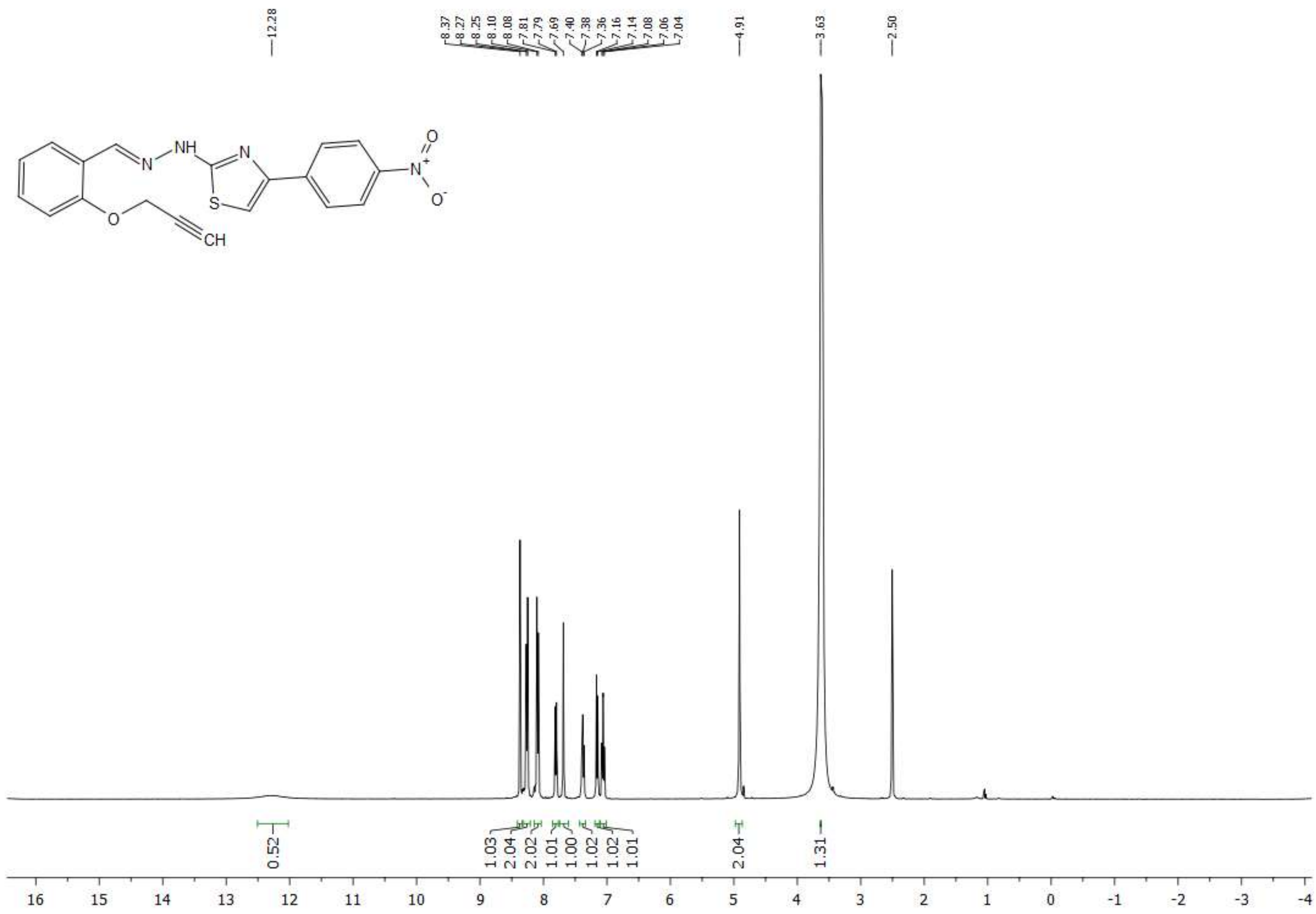

^{13}C NMR spectrum of (*E*)-4-(4-Nitrophenyl)-2-(2-(2-(prop-2-yn-1-yloxy)benzylidene)hydrazinyl)thiazole (12)

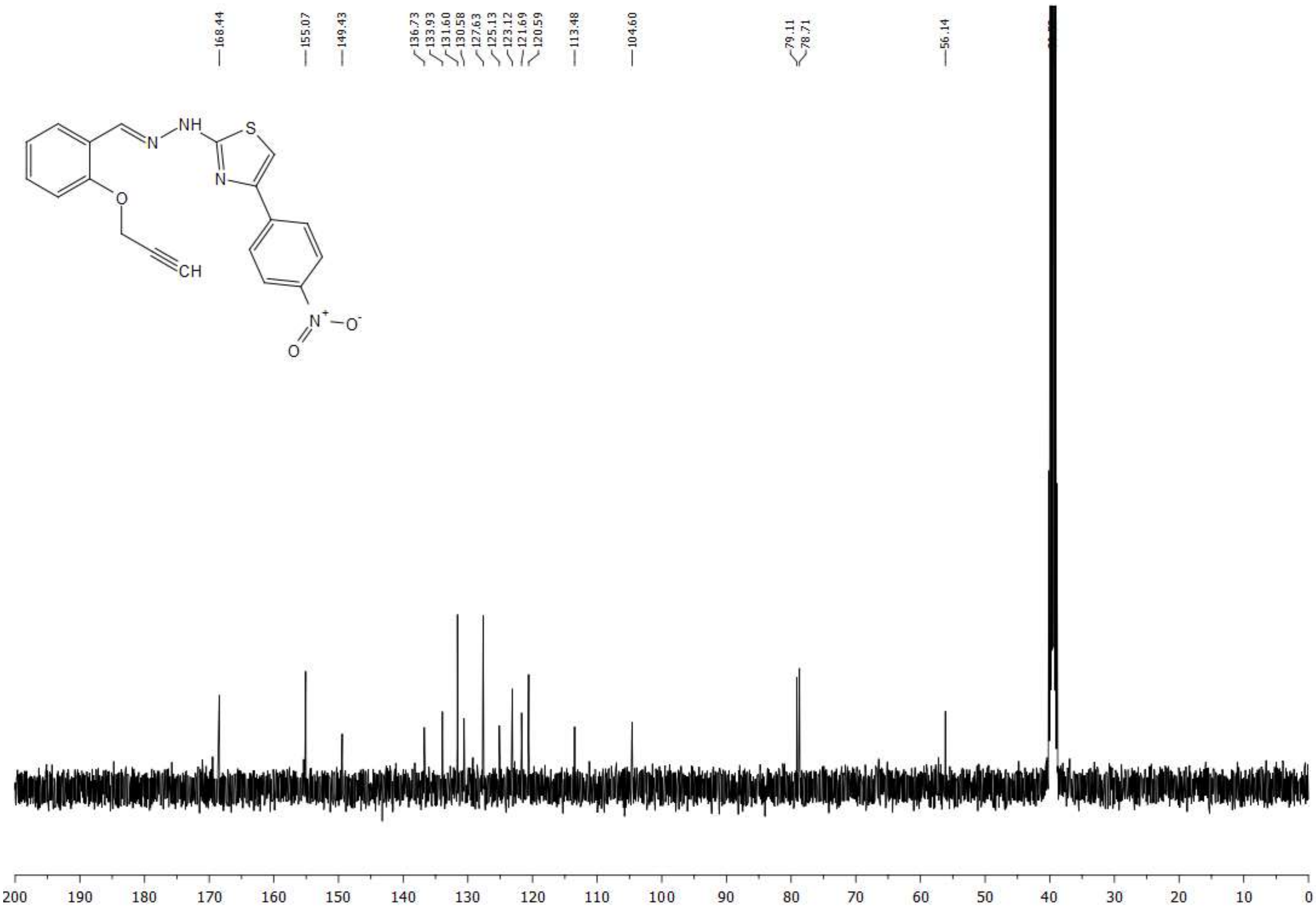

FT-IR spectrum of (*E*)-4-(4-Nitrophenyl)-2-(2-(2-(prop-2-yn-1-yloxy)benzylidene)hydrazinyl)thiazole (12)

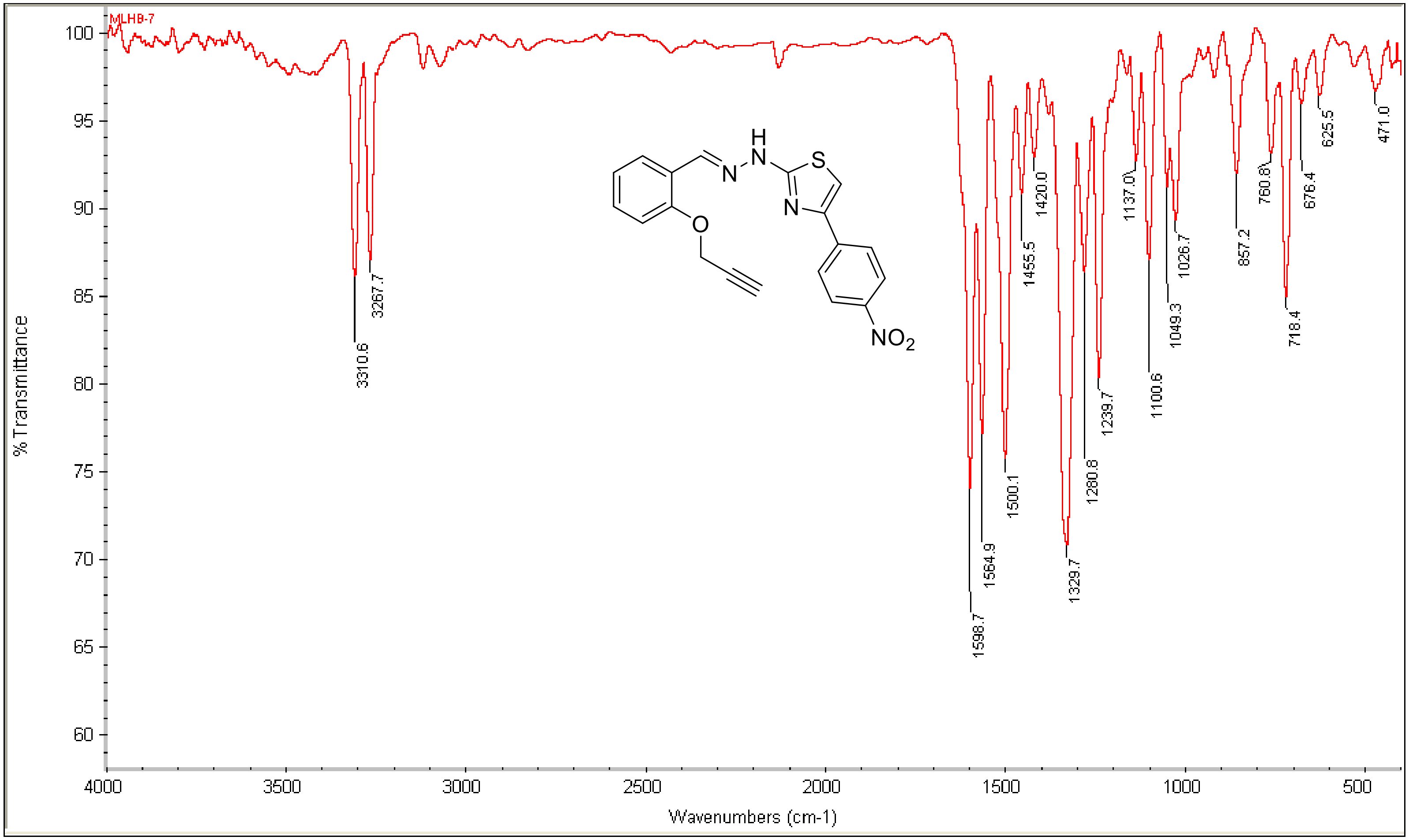

Mass spectrum of (*E*)-4-(2-(2-(2-(Prop-2-yn-1-yloxy)benzylidene)hydrazinyl)thiazol-4-yl)benzonitrile (13)

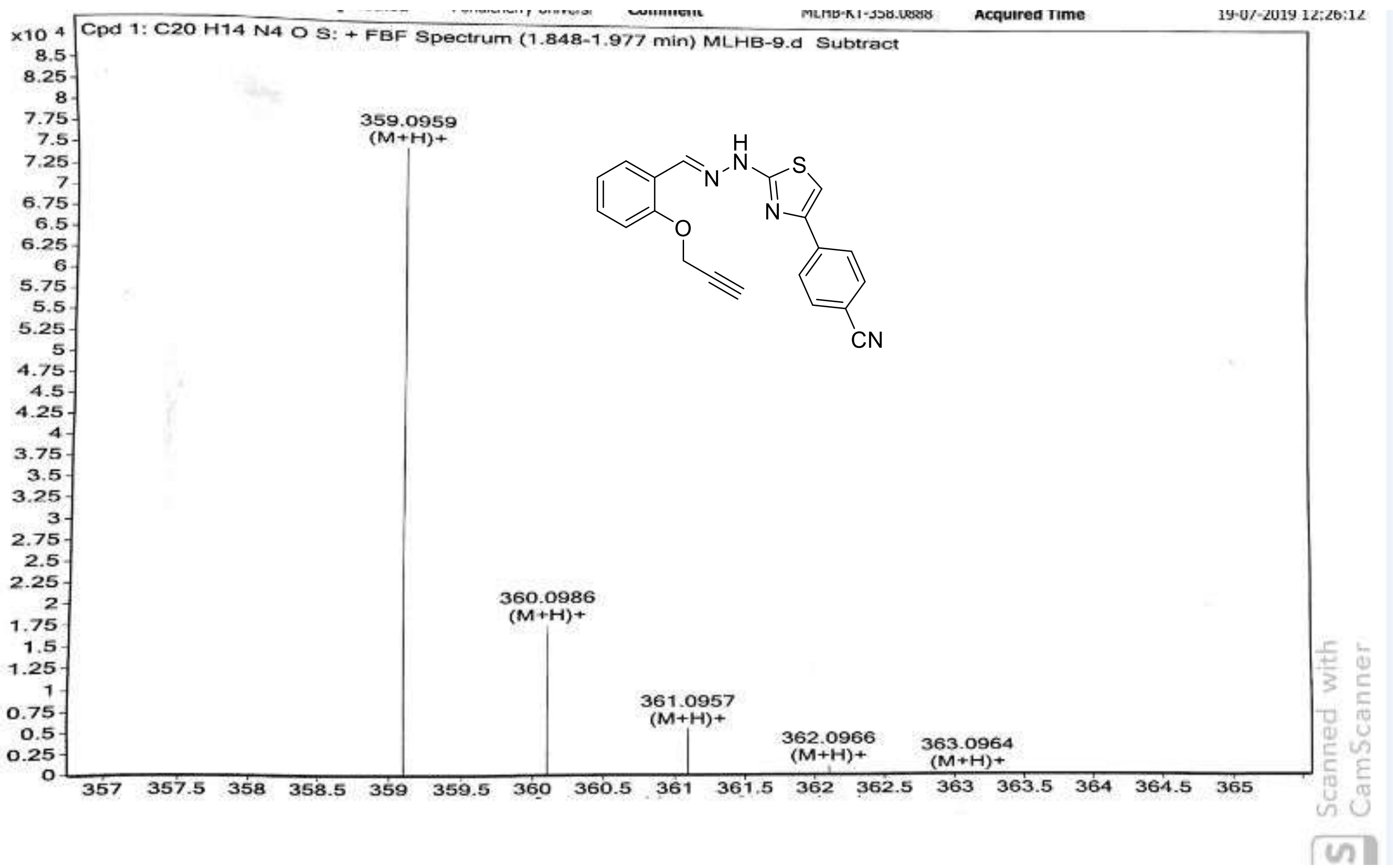

^{1}H NMR spectrum of (*E*)-4-(2-(2-(2-(Prop-2-yn-1-yloxy)benzylidene)hydrazinyl)thiazol-4-yl)benzonitrile (13)

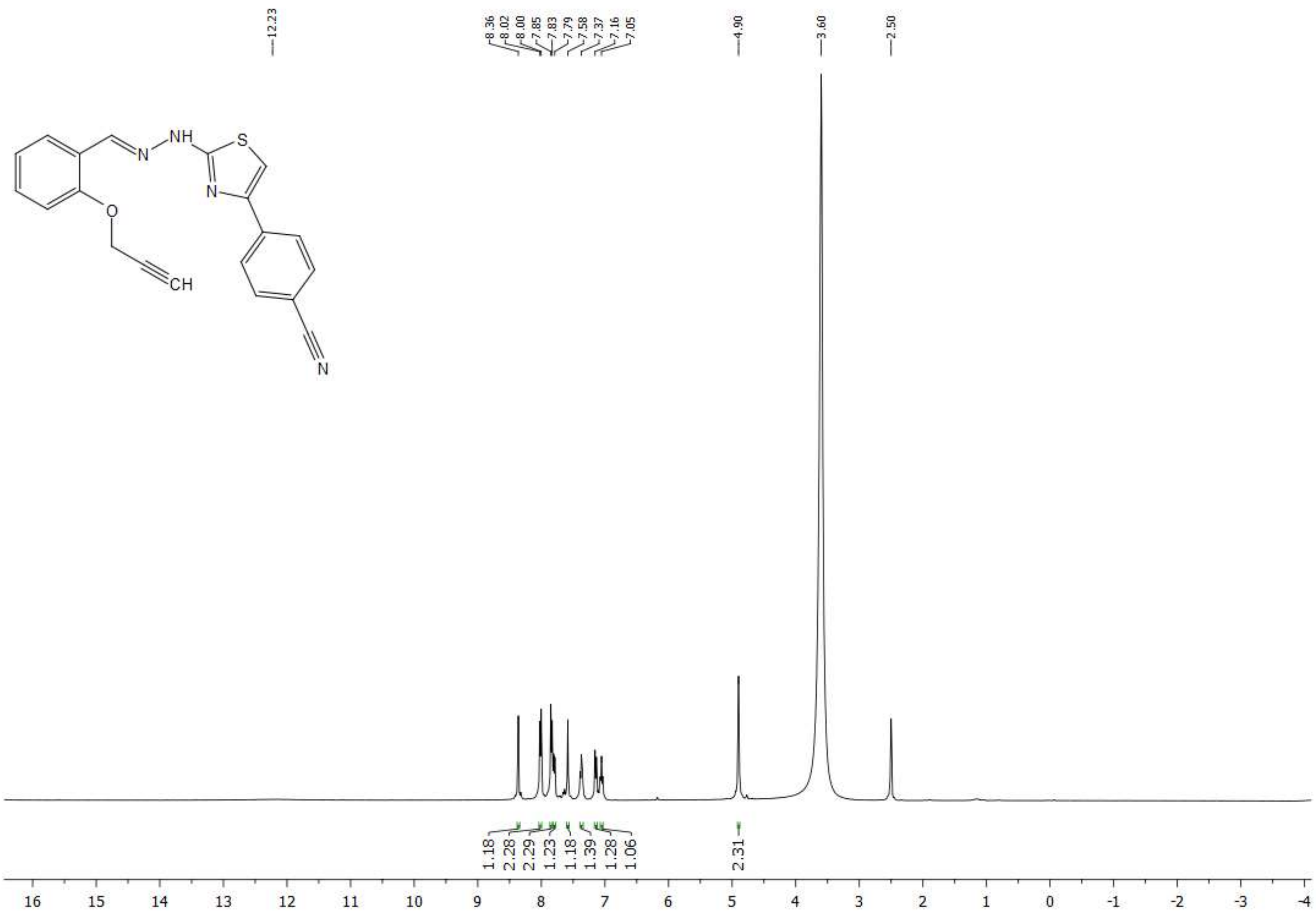

^{13}C NMR spectrum of (*E*)-4-(2-(2-(2-(Prop-2-yn-1-yloxy)benzylidene)hydrazinyl)thiazol-4-yl)benzonitrile (13)

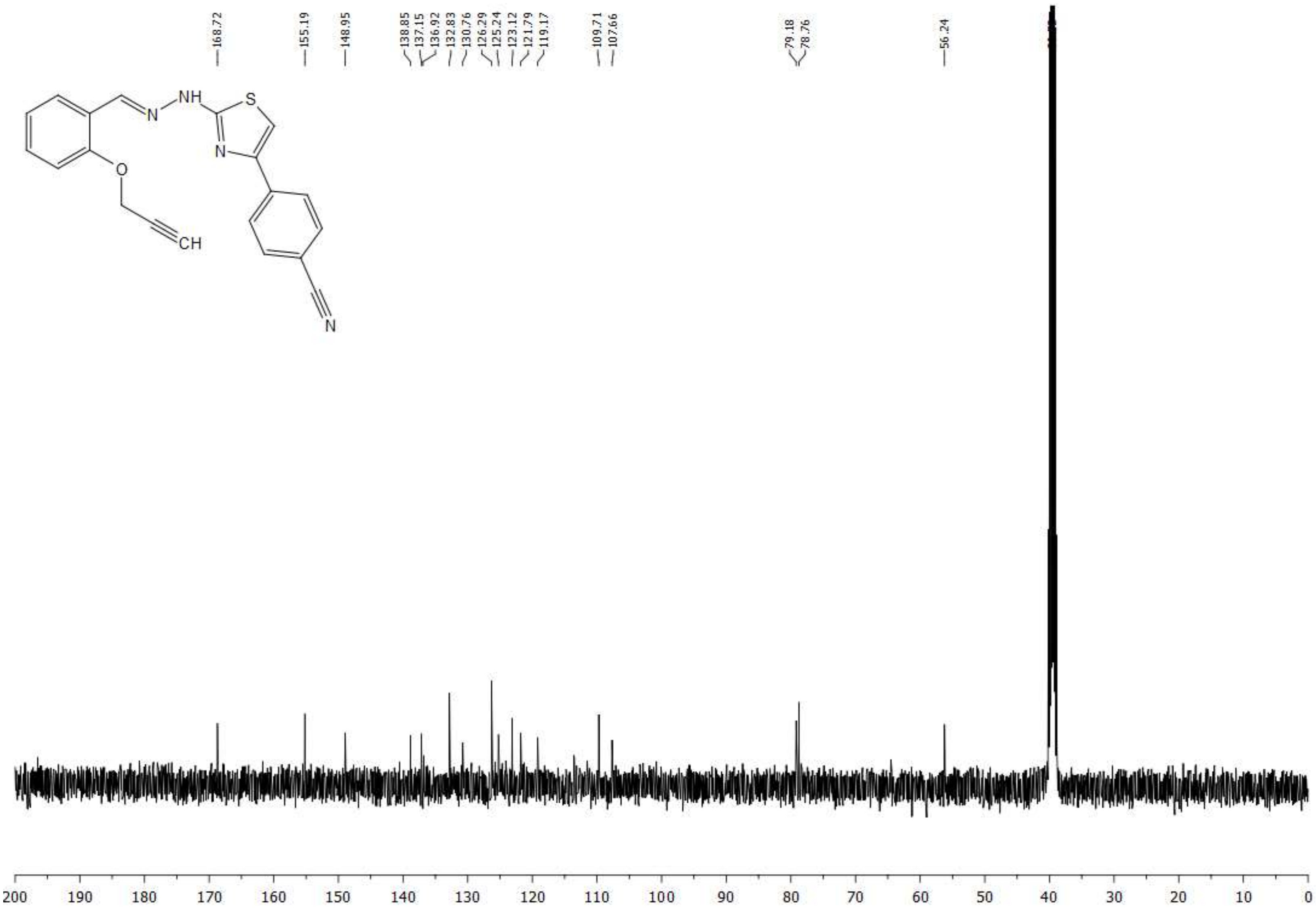

FT-IR spectrum of (*E*)-4-(2-(2-(2-(Prop-2-yn-1-yloxy)benzylidene)hydrazinyl)thiazol-4-yl)benzonitrile (13)

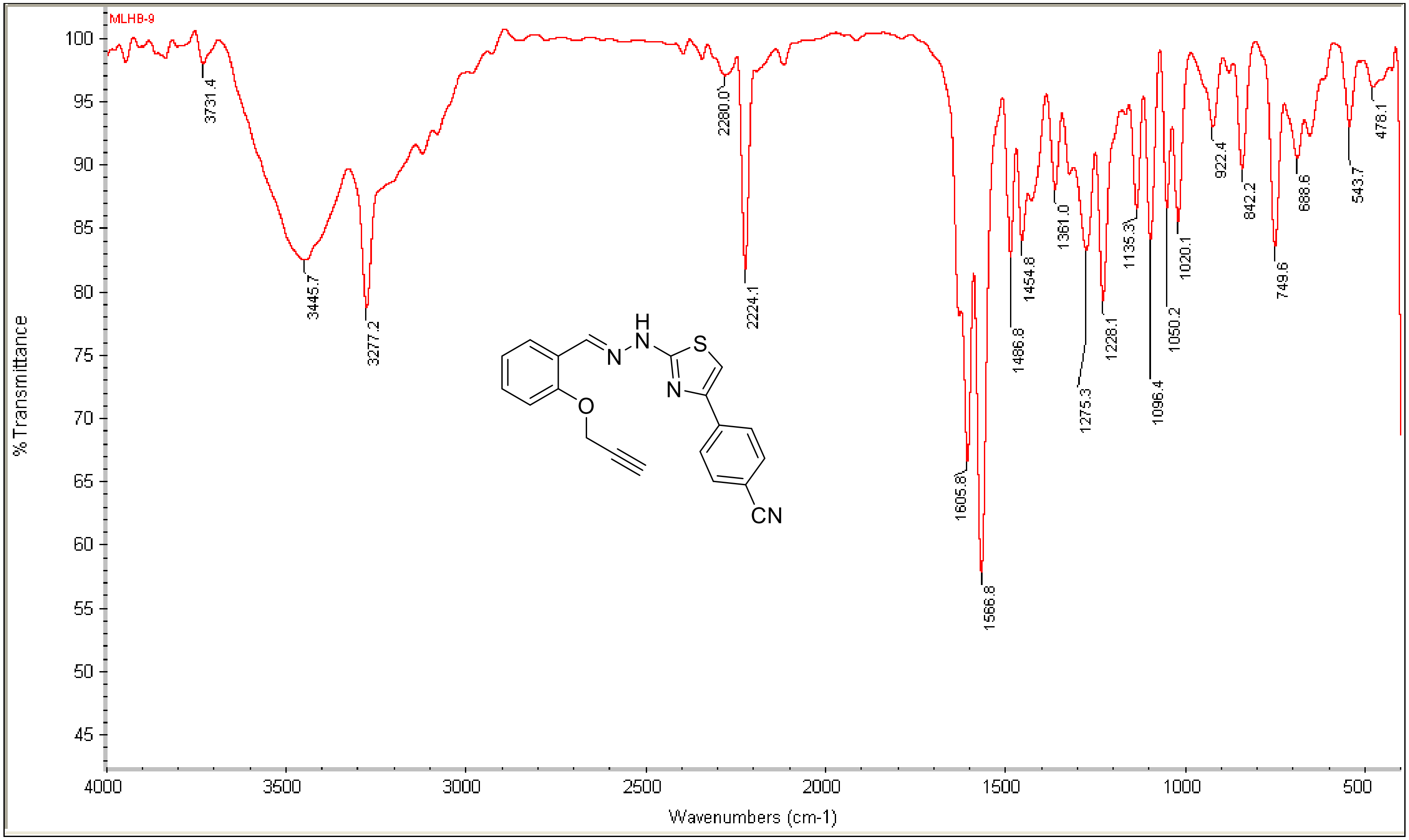

Mass spectrum of (*E*)-2-(2-(2-(Prop-2-yn-1-yloxy)benzylidene)hydrazinyl)-4-(p-tolyl)thiazole (14)

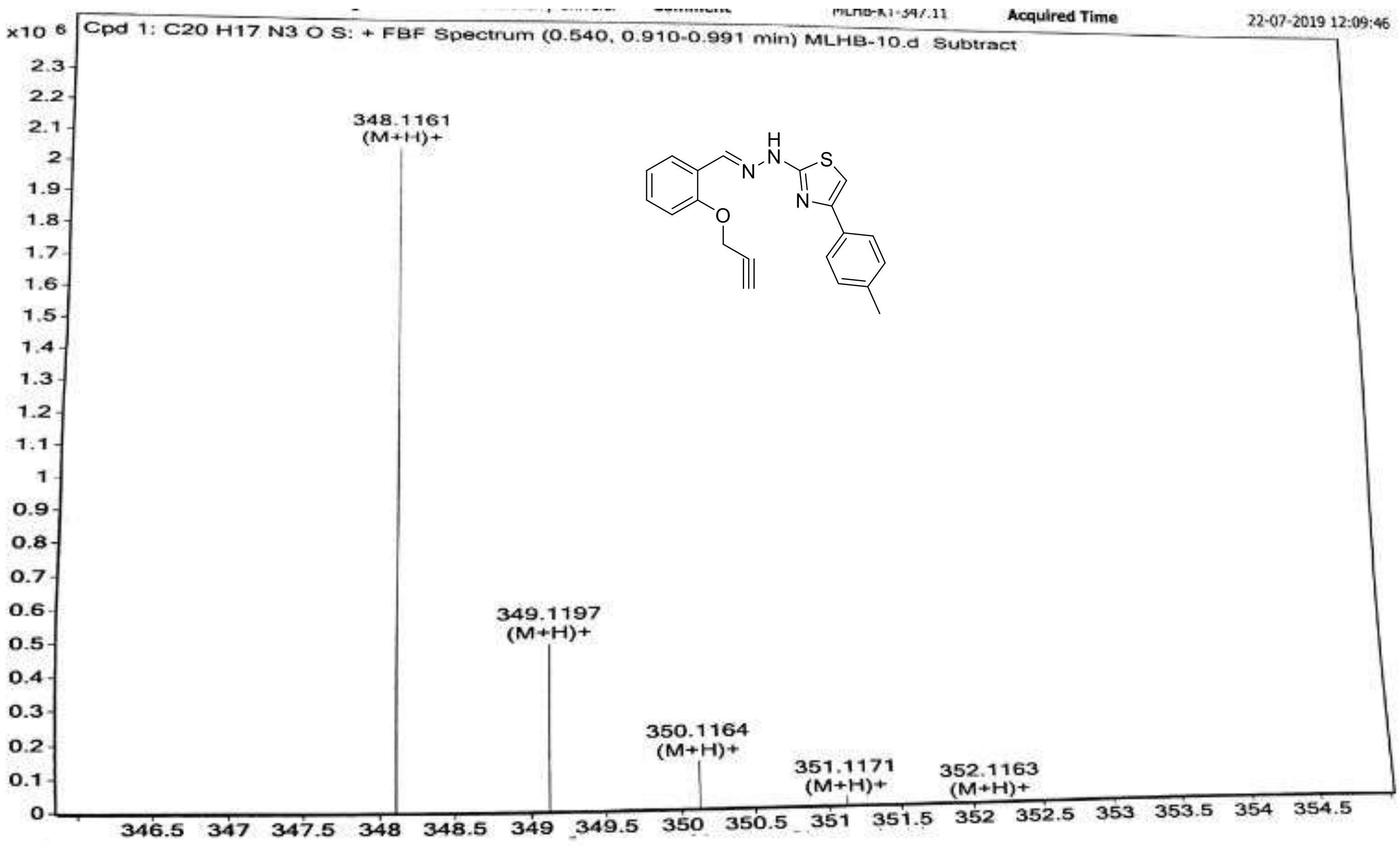

^{1}H NMR spectrum of (*E*)-2-(2-(2-(Prop-2-yn-1-yloxy)benzylidene)hydrazinyl)-4-(p-tolyl)thiazole (14)

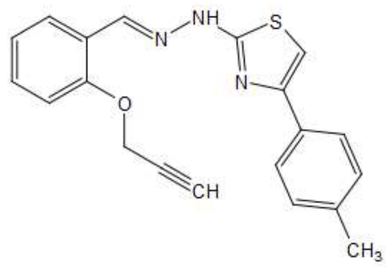

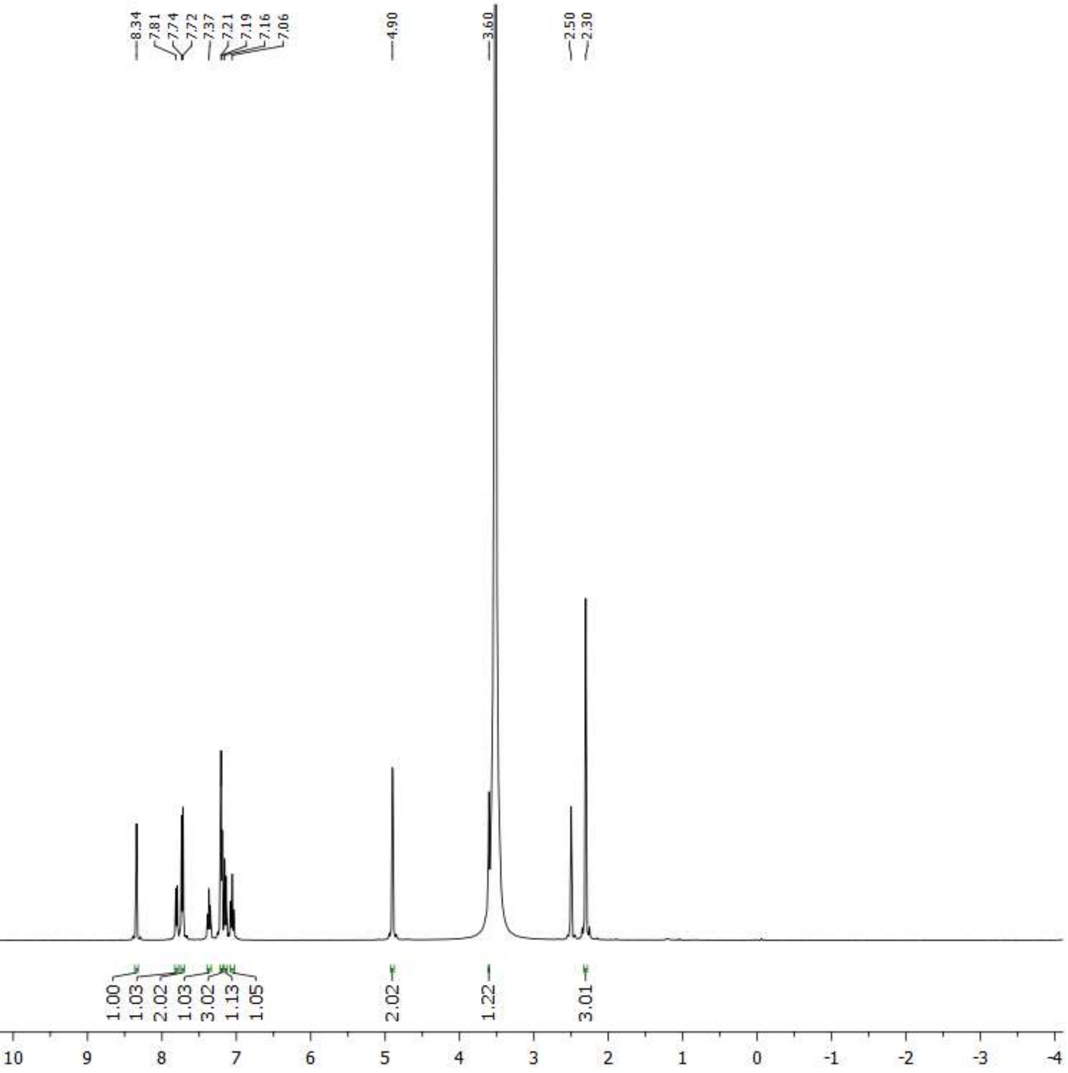

[13]NMR spectrum of (*E*)-2-(2-(2-(Prop-2-yn-1-yloxy)benzylidene)hydrazinyl)-4-(p-tolyl)thiazole (14)

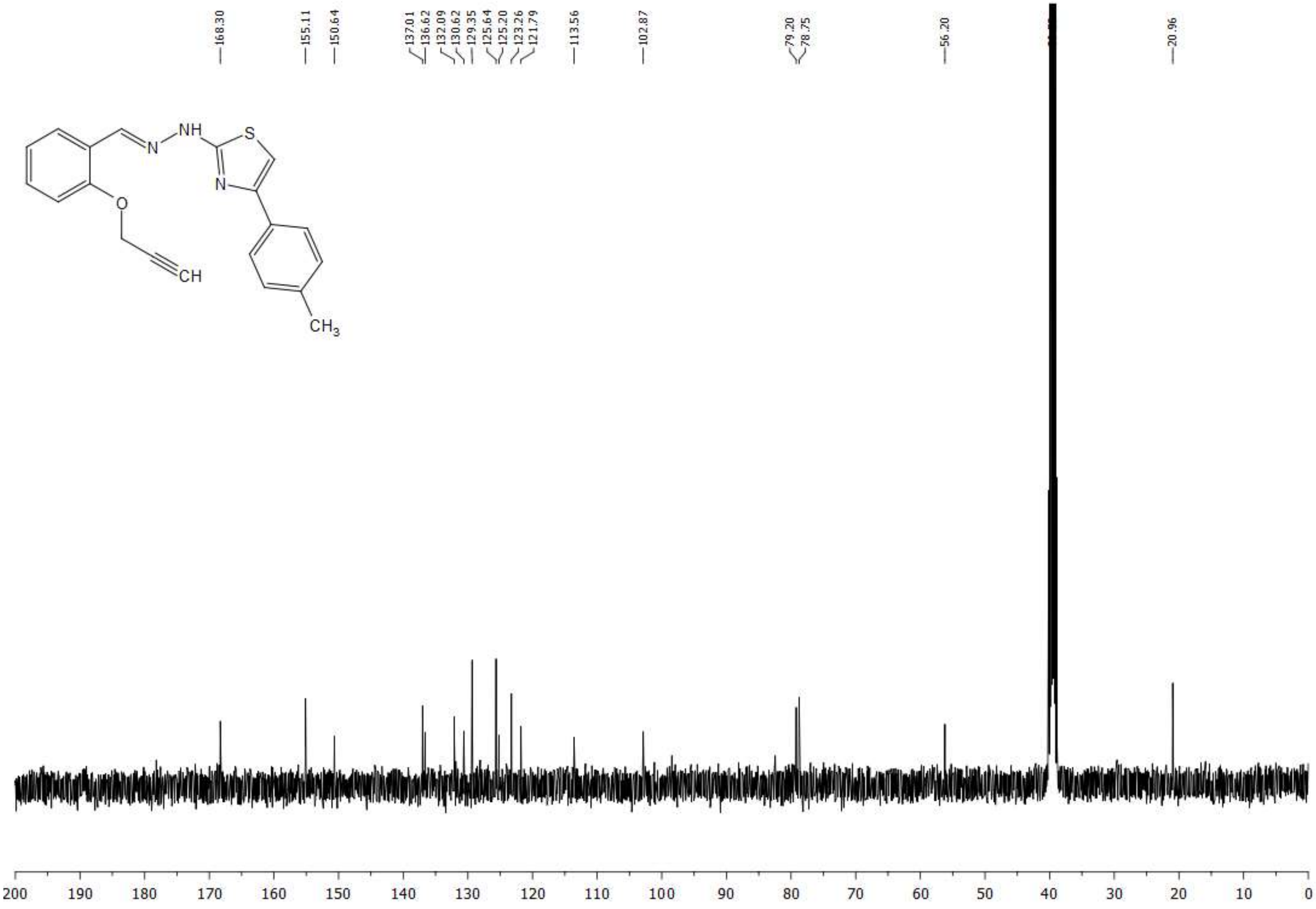

FT-IR spectrum of (*E*)-2-(2-(2-(Prop-2-yn-1-yloxy)benzylidene)hydrazinyl)-4-(p-tolyl)thiazole (14)

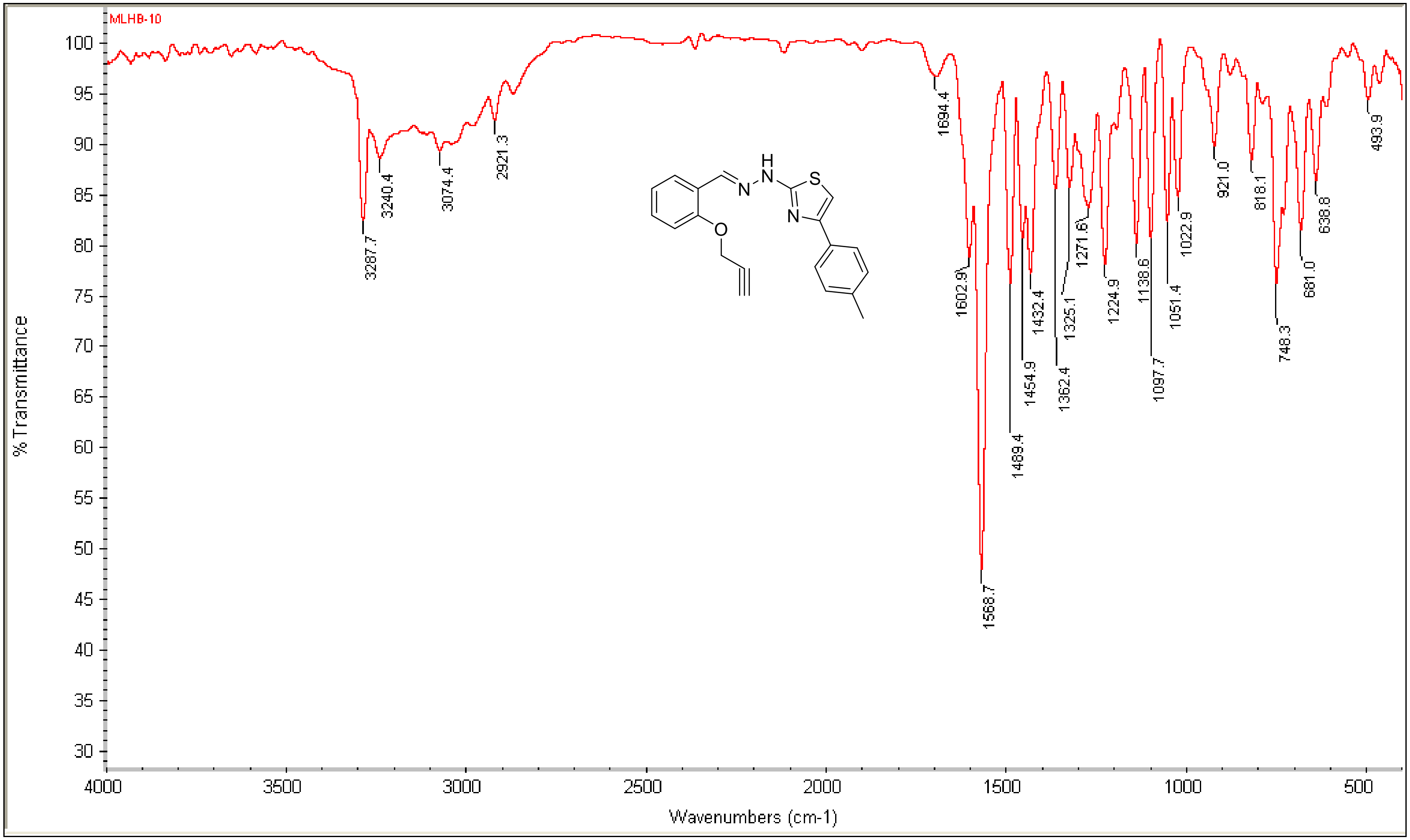

Mass spectrum of (*E*)-4-(4-Methoxyphenyl)-2-(2-(2-(prop-2-yn-1-yloxy)benzylidene)hydrazinyl)thiazole (15)

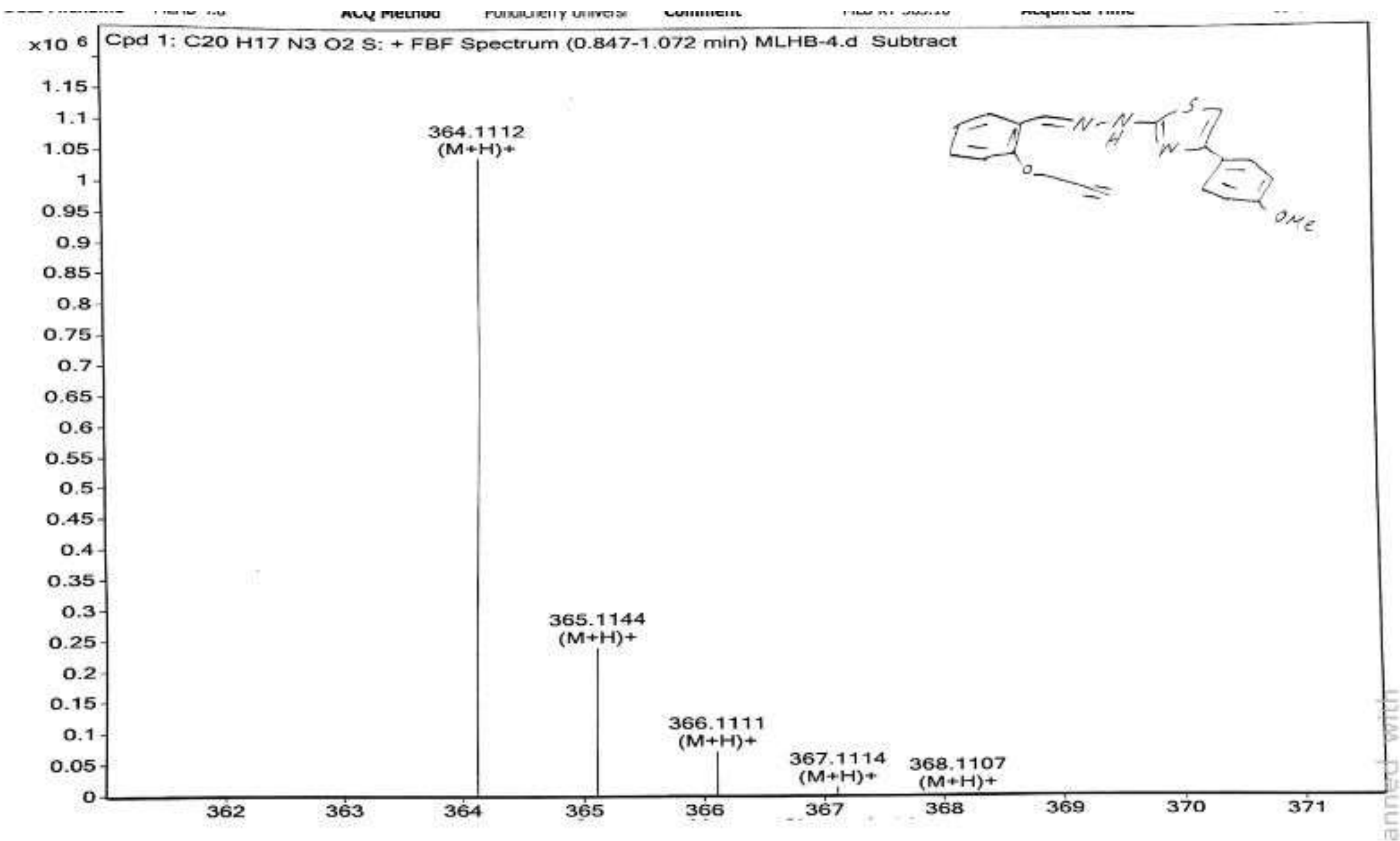

Scanned with CamScanner

^{1}H NMR spectrum of (*E*)-4-(4-Methoxyphenyl)-2-(2-(2-(prop-2-yn-1-yloxy)benzylidene)hydrazinyl)thiazole (15)

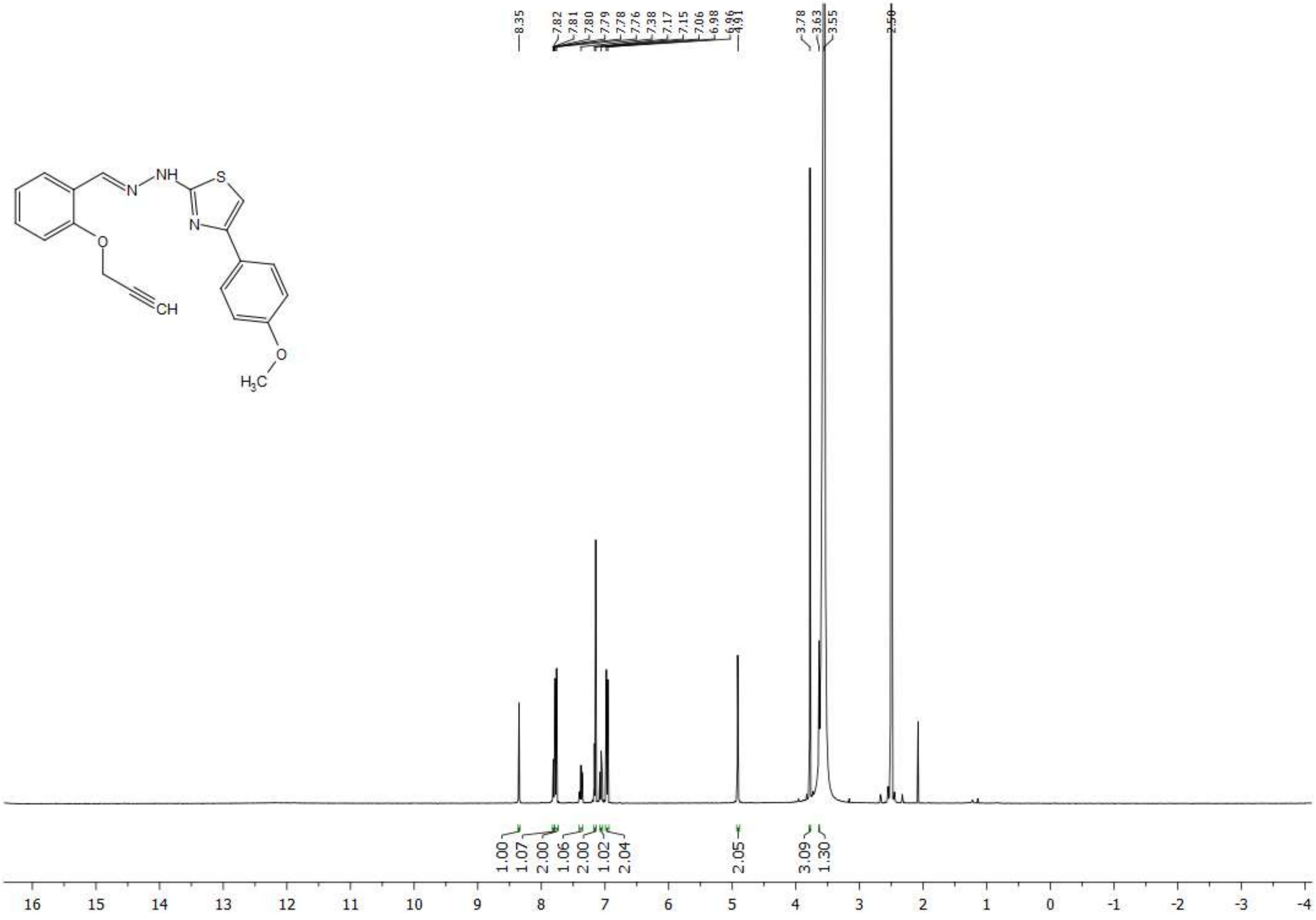

^{13}C NMR spectrum of (*E*)-4-(4-Methoxyphenyl)-2-(2-(2-(prop-2-yn-1-yloxy)benzylidene)hydrazinyl)thiazole (15)

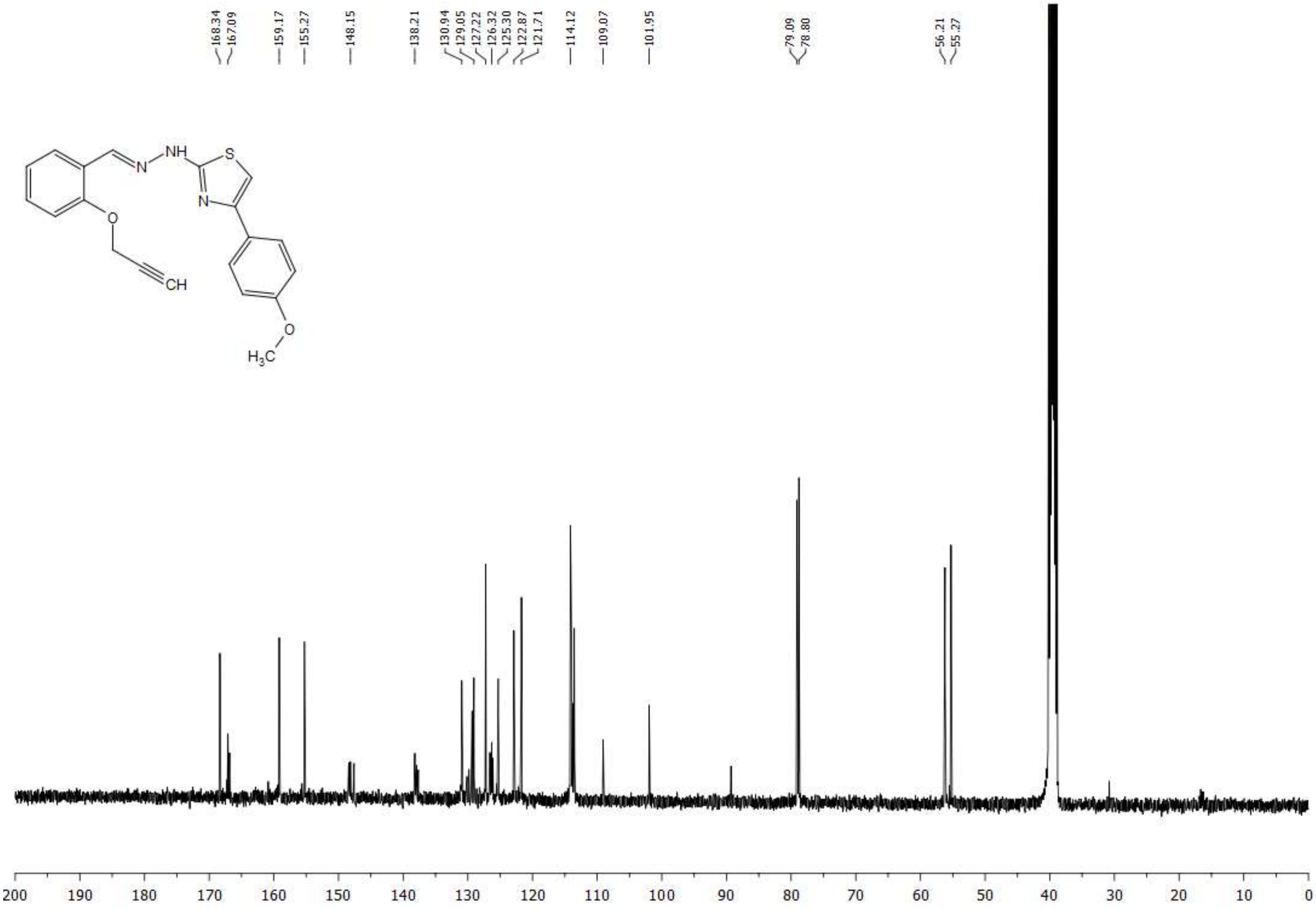

FT-IR spectrum of (*E*)-4-(4-Methoxyphenyl)-2-(2-(2-(prop-2-yn-1-yloxy)benzylidene)hydrazinyl)thiazole (15)

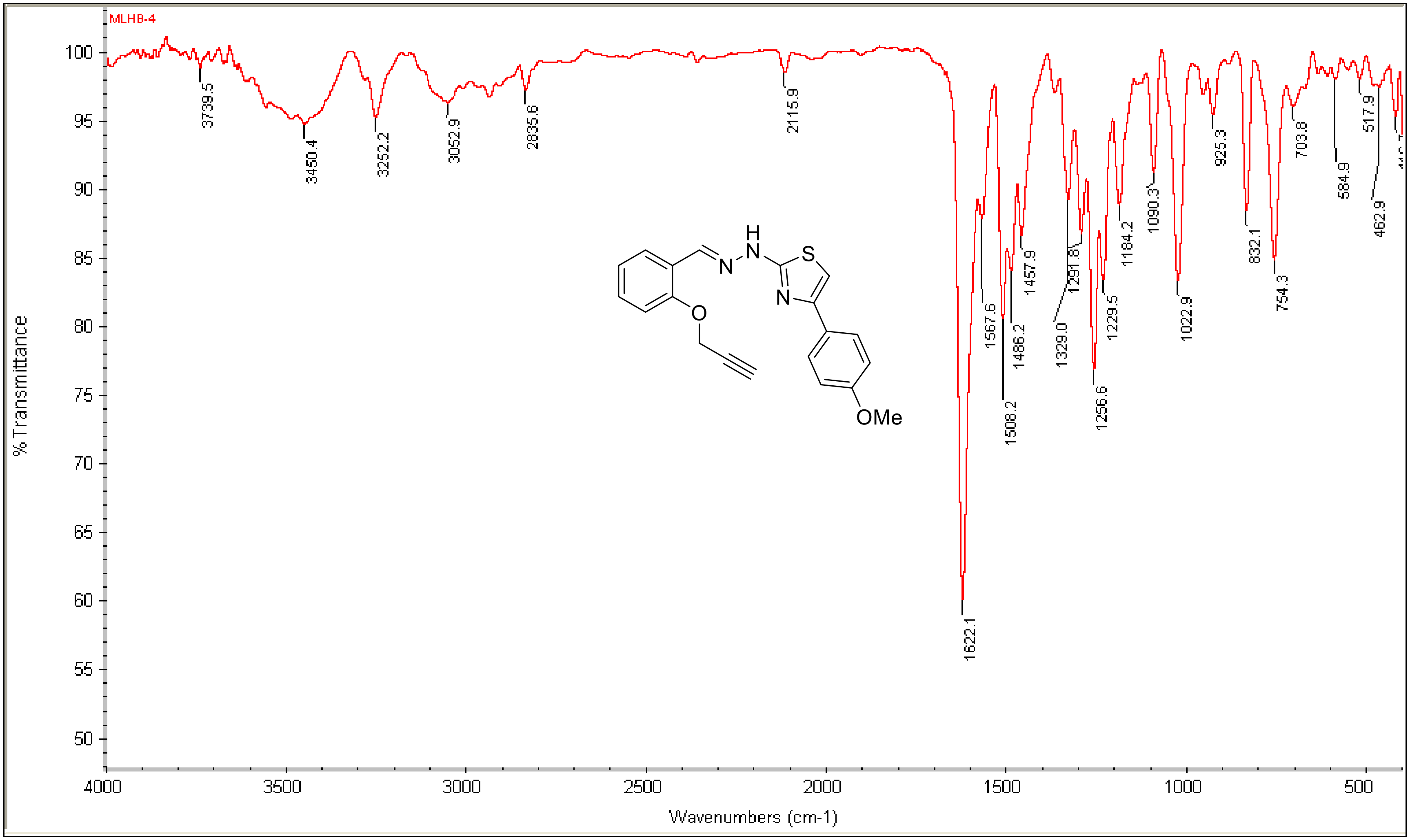

Mass spectrum of (*E*)-2-(2-(3-Ethoxy-4-(prop-2-yn-1-yloxy)benzylidene)hydrazinyl)-4-phenylthiazole (16)

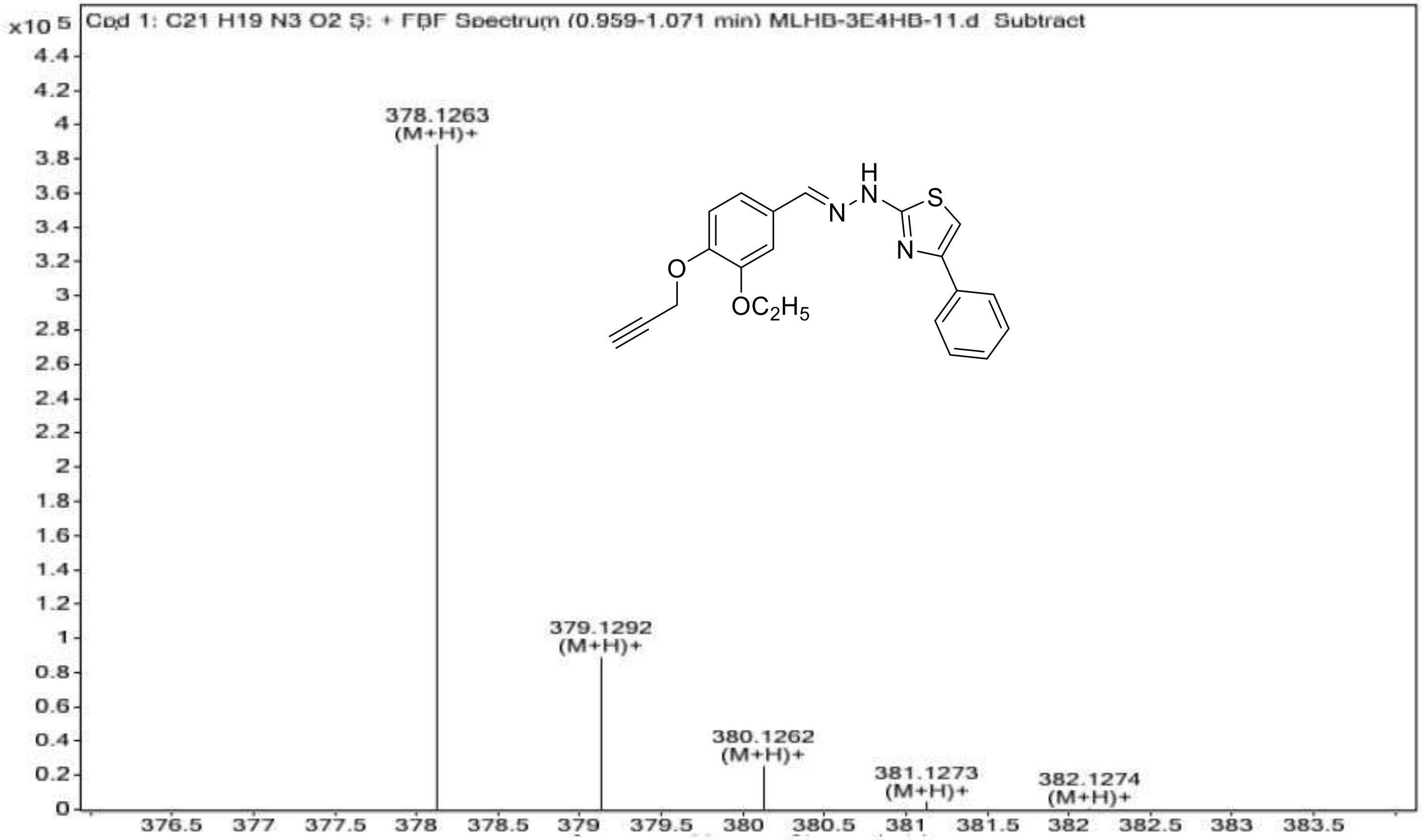

^{1}H NMR spectrum of (*E*)-2-(2-(3-Ethoxy-4-(prop-2-yn-1-yloxy)benzylidene)hydrazinyl)-4-phenylthiazole (16)

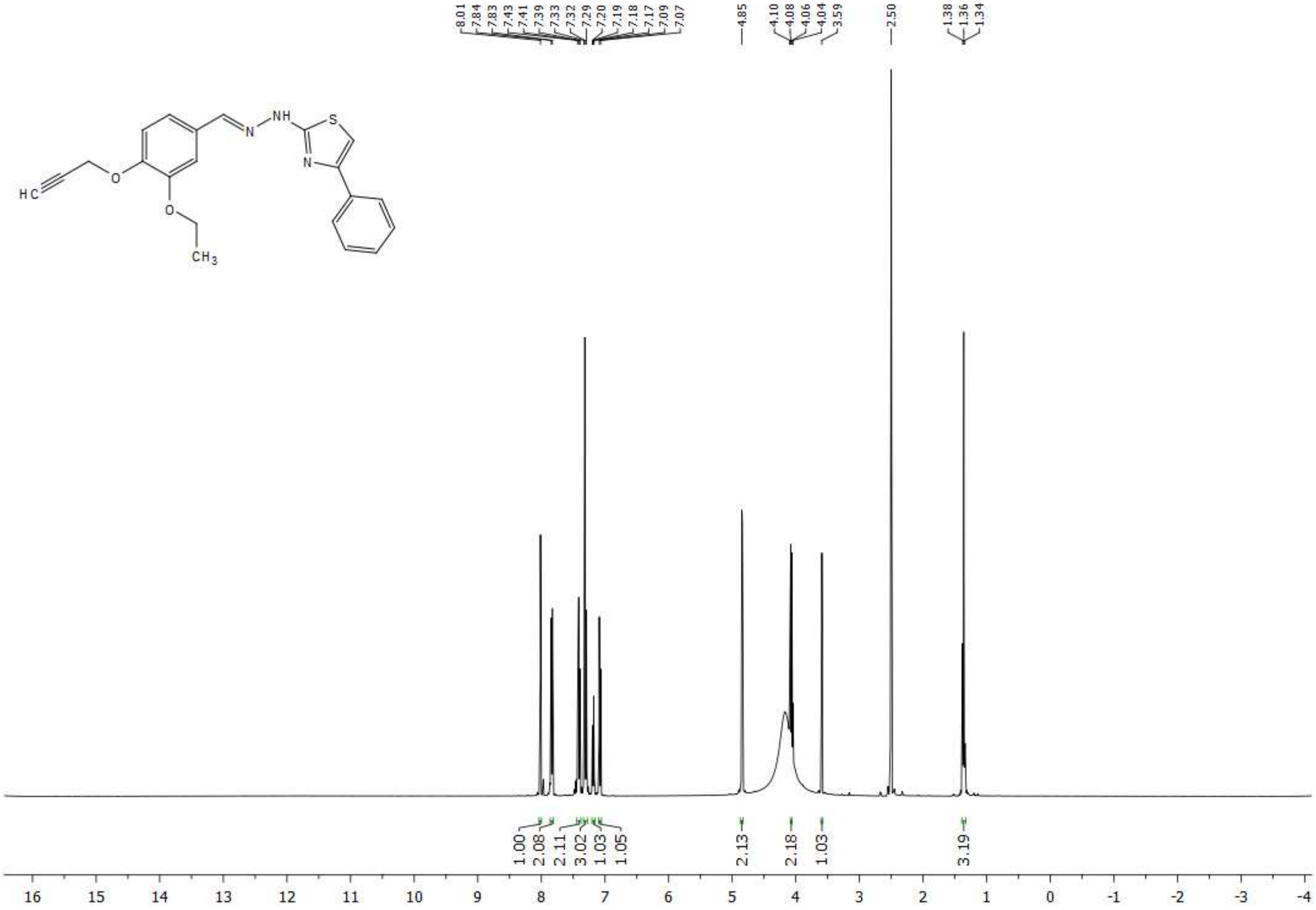

[13]NMR spectrum of (*E*)-2-(2-(3-Ethoxy-4-(prop-2-yn-1-yloxy)benzylidene)hydrazinyl)-4-phenylthiazole (16)

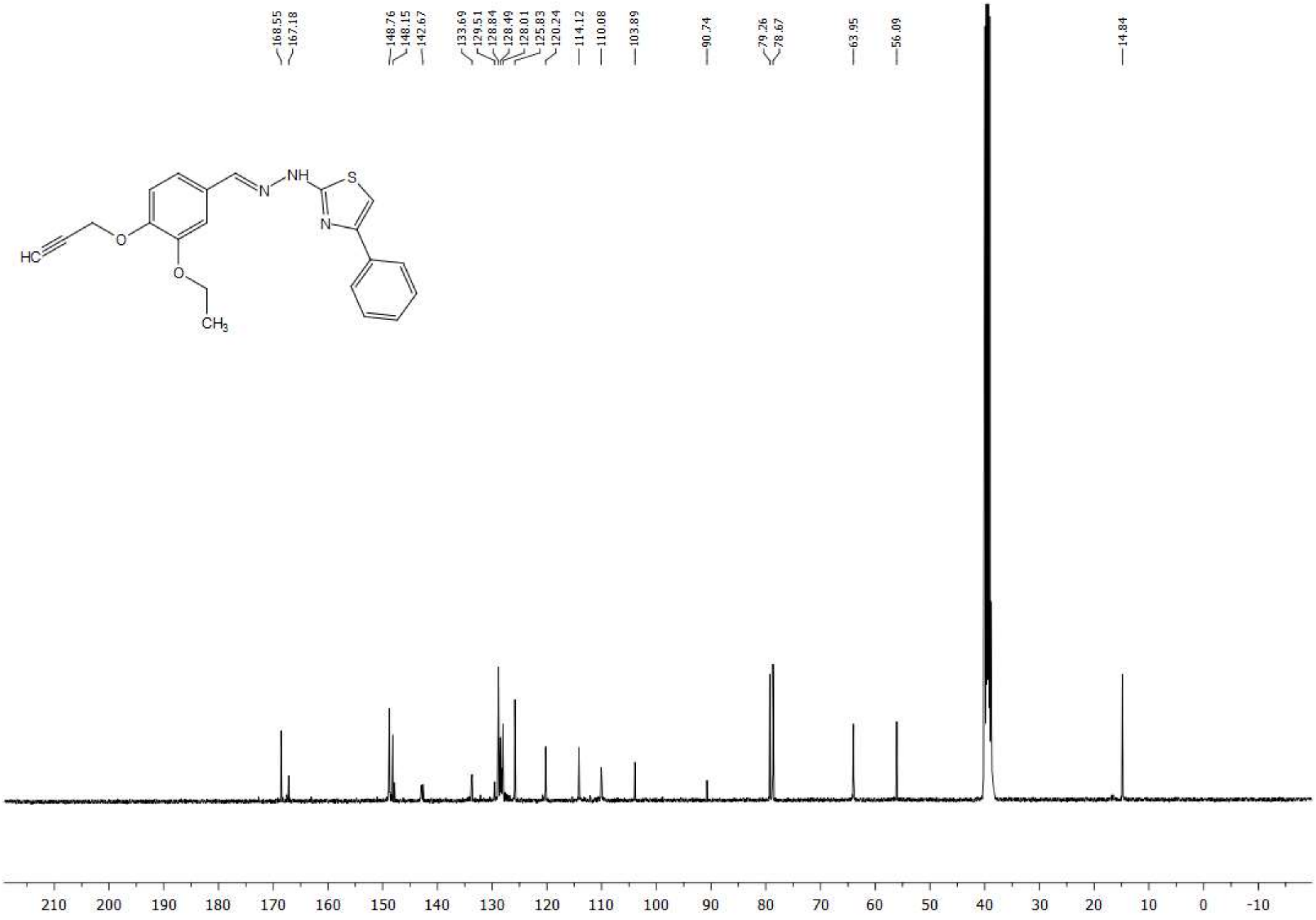

FT-IR spectrum of (*E*)-2-(2-(3-Ethoxy-4-(prop-2-yn-1-yloxy)benzylidene)hydrazinyl)-4-phenylthiazole (16)

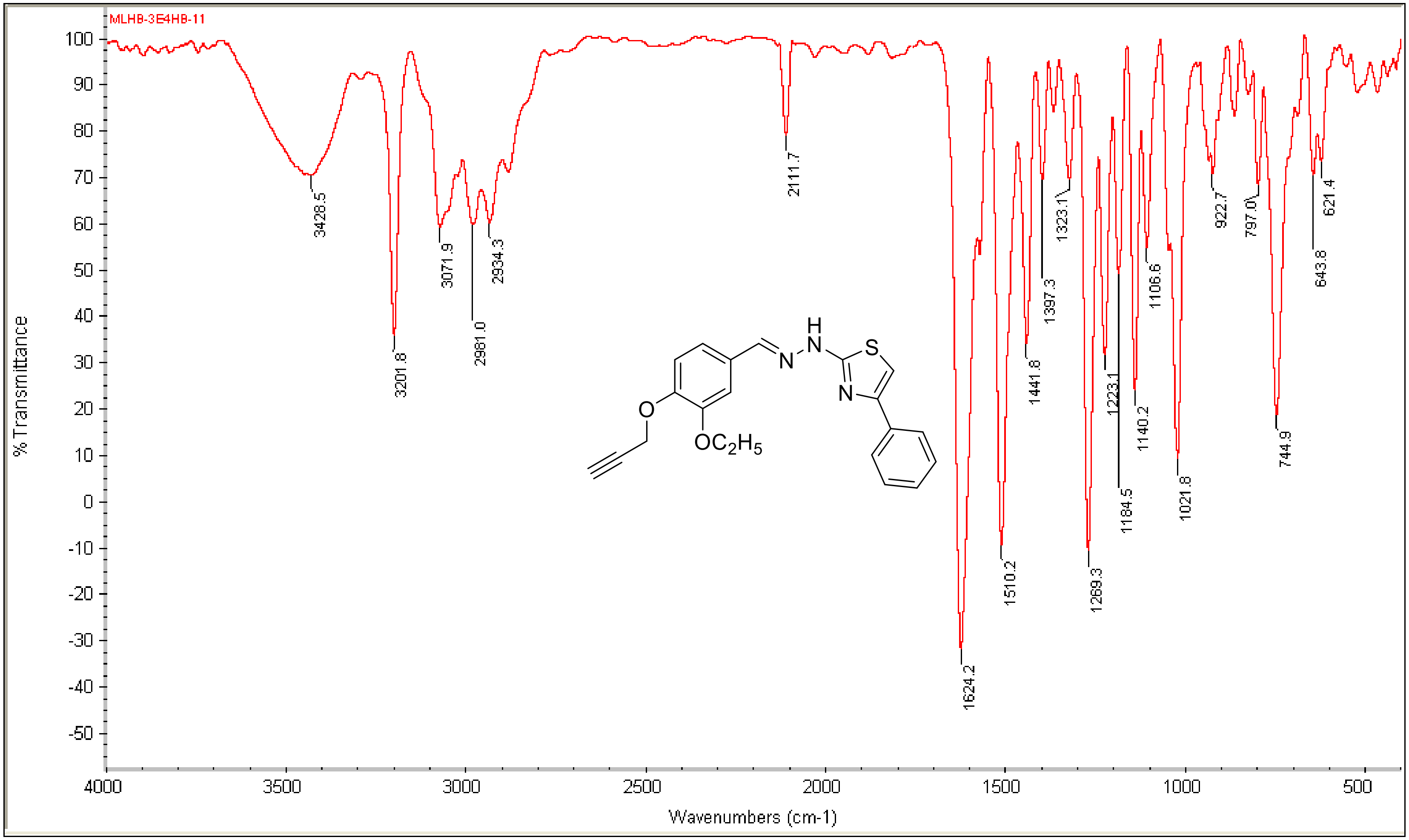

Mass spectrum of (*E*)-2-(2-(3-Ethoxy-4-(prop-2-yn-1-yloxy)benzylidene)hydrazinyl)-4-(4-fluorophenyl)thiazole (17)

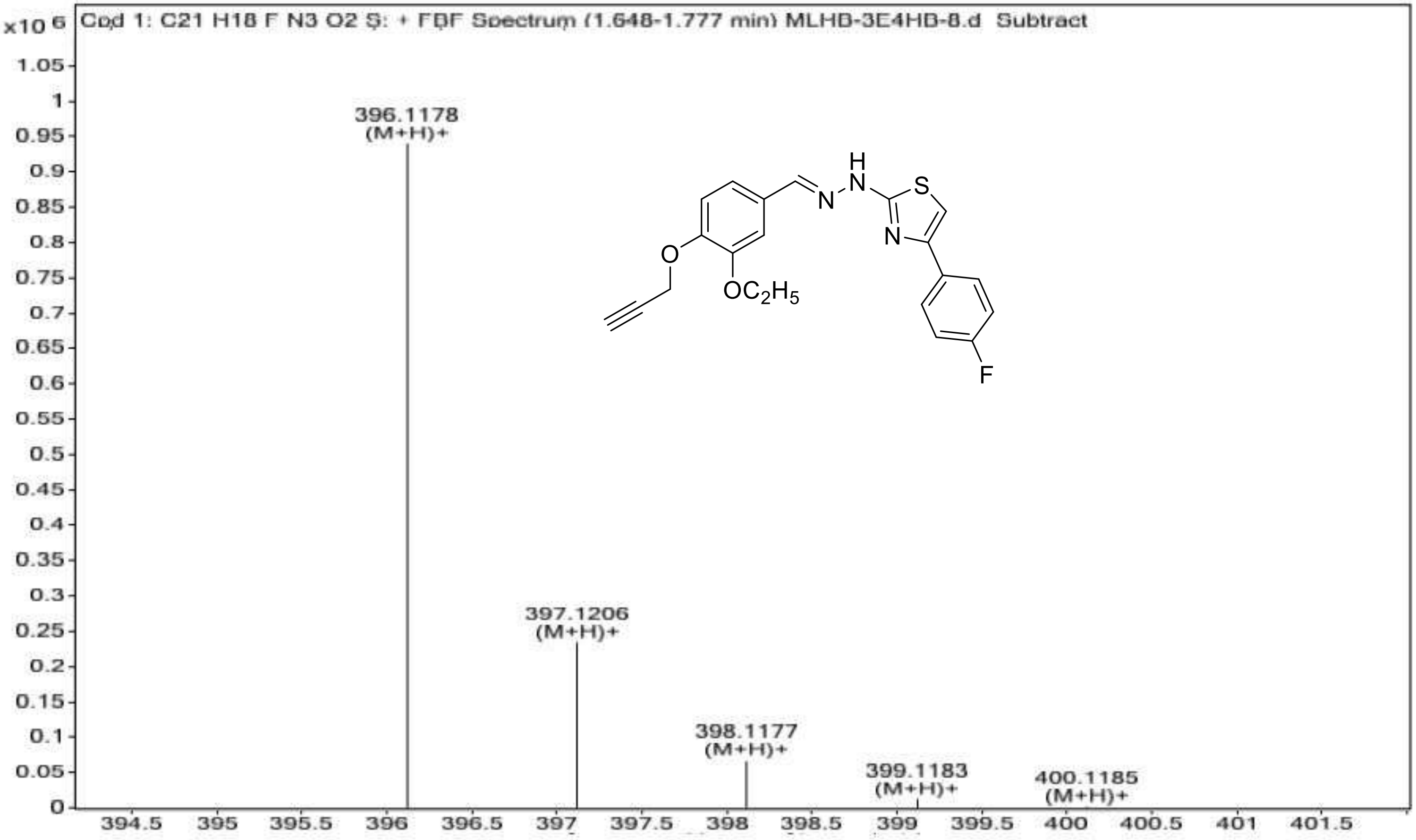

^{1}H NMR spectrum of (*E*)-2-(2-(3-Ethoxy-4-(prop-2-yn-1-yloxy)benzylidene)hydrazinyl)-4-(4-fluorophenyl)thiazole (17)

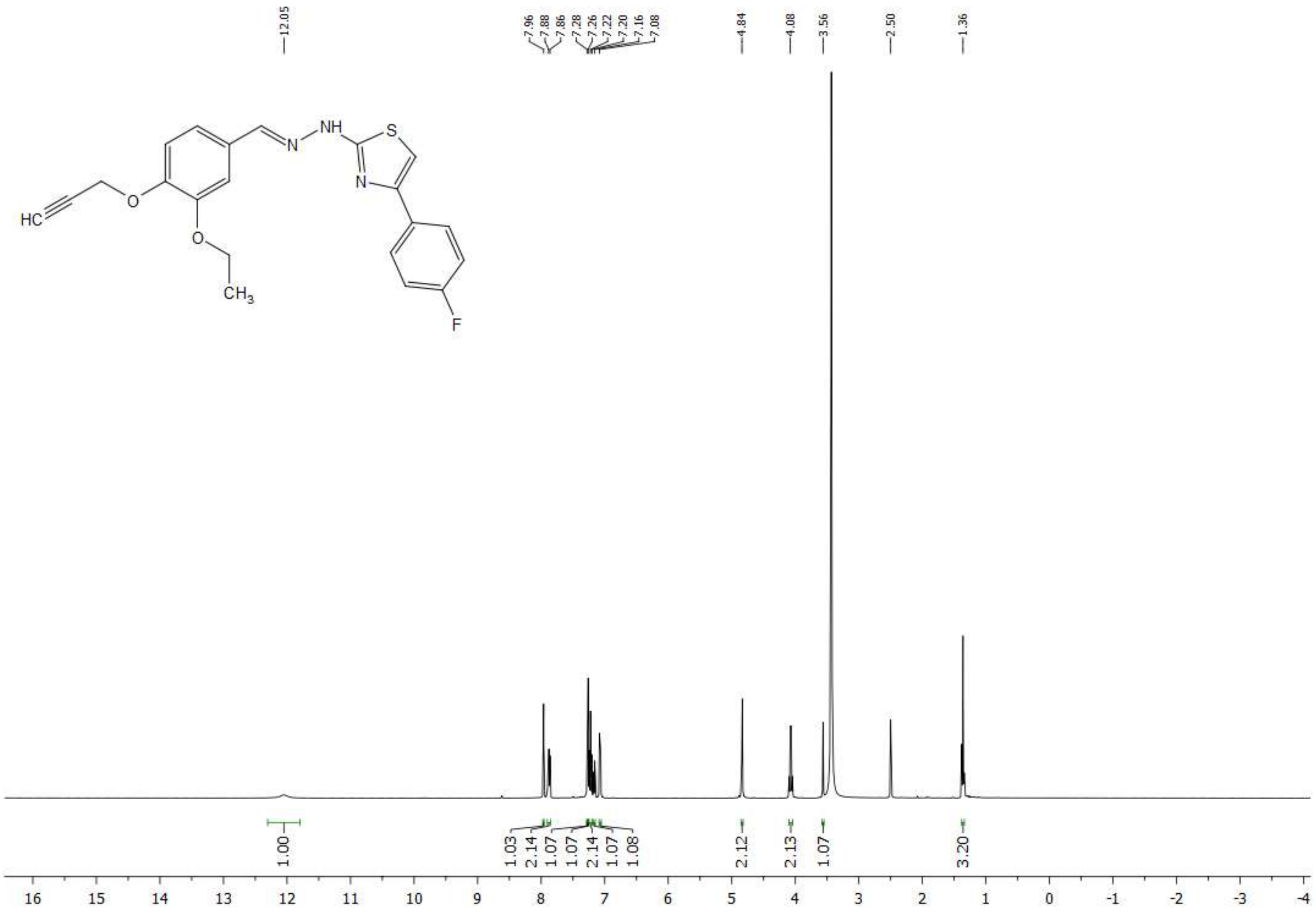

^{13}C NMR spectrum of (*E*)-2-(2-(3-Ethoxy-4-(prop-2-yn-1-yloxy)benzylidene)hydrazinyl)-4-(4-fluorophenyl)thiazole (17)

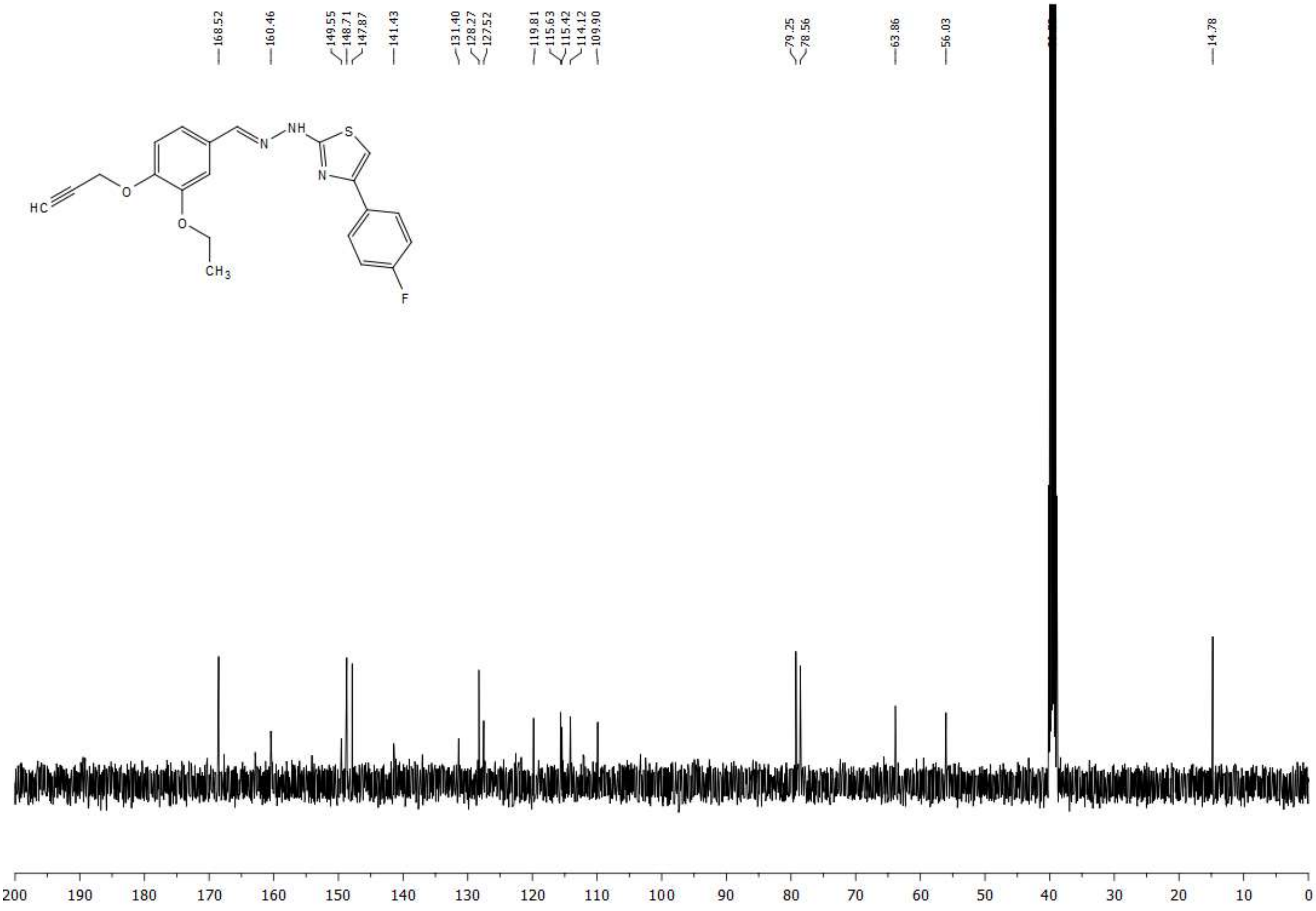

FT-IR spectrum of (*E*)-2-(2-(3-Ethoxy-4-(prop-2-yn-1-yloxy)benzylidene)hydrazinyl)-4-(4-fluorophenyl)thiazole (17)

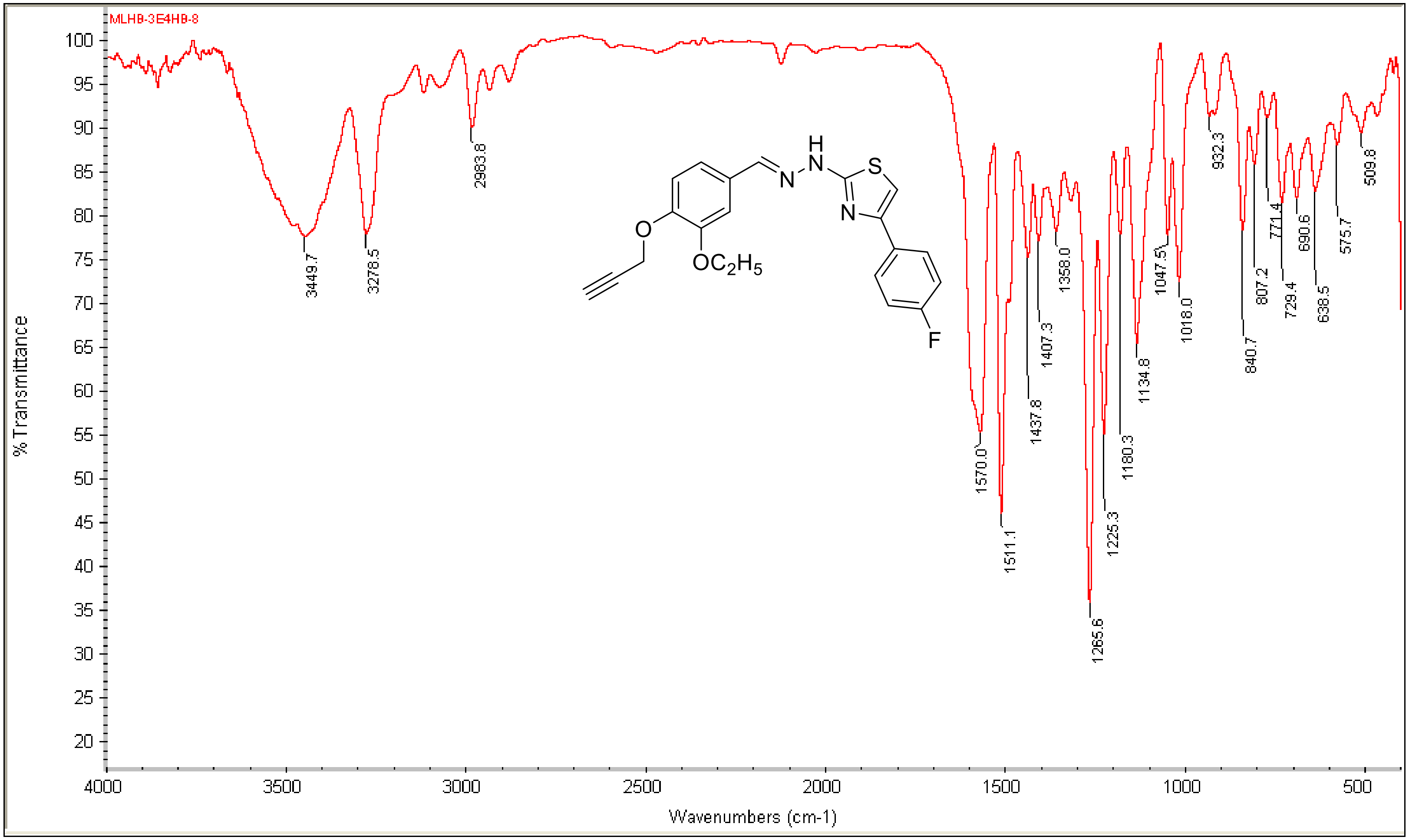

Mass spectrum of (*E*)-4-(4-Chlorophenyl)-2-(2-(3-ethoxy-4-(prop-2-yn-1-yloxy)benzylidene)hydrazinyl)thiazole (18)

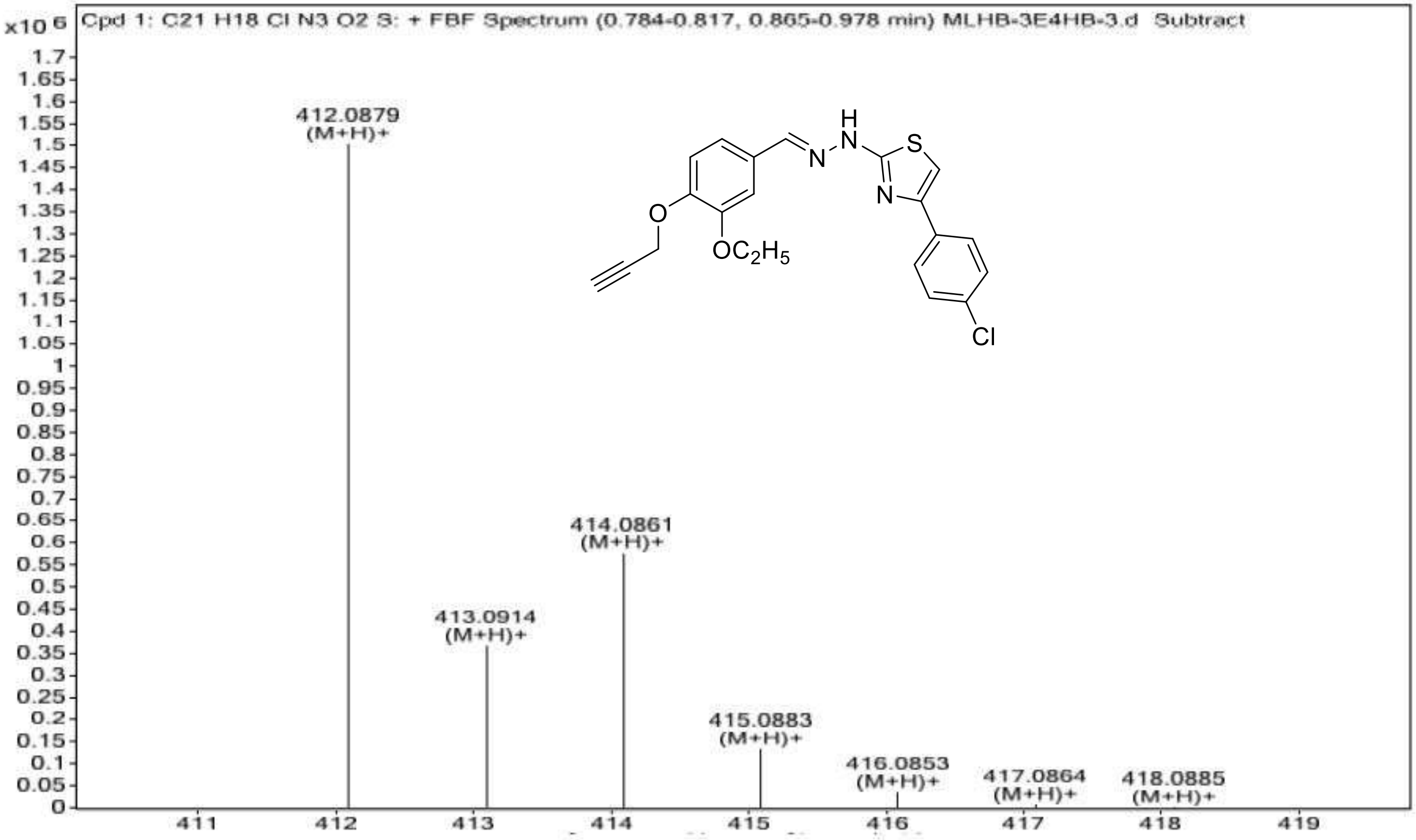

¹H NMR spectrum of (*E*)-4-(4-Chlorophenyl)-2-(2-(3-ethoxy-4-(prop-2-yn-1-yloxy)benzylidene)hydrazinyl)thiazole (18)

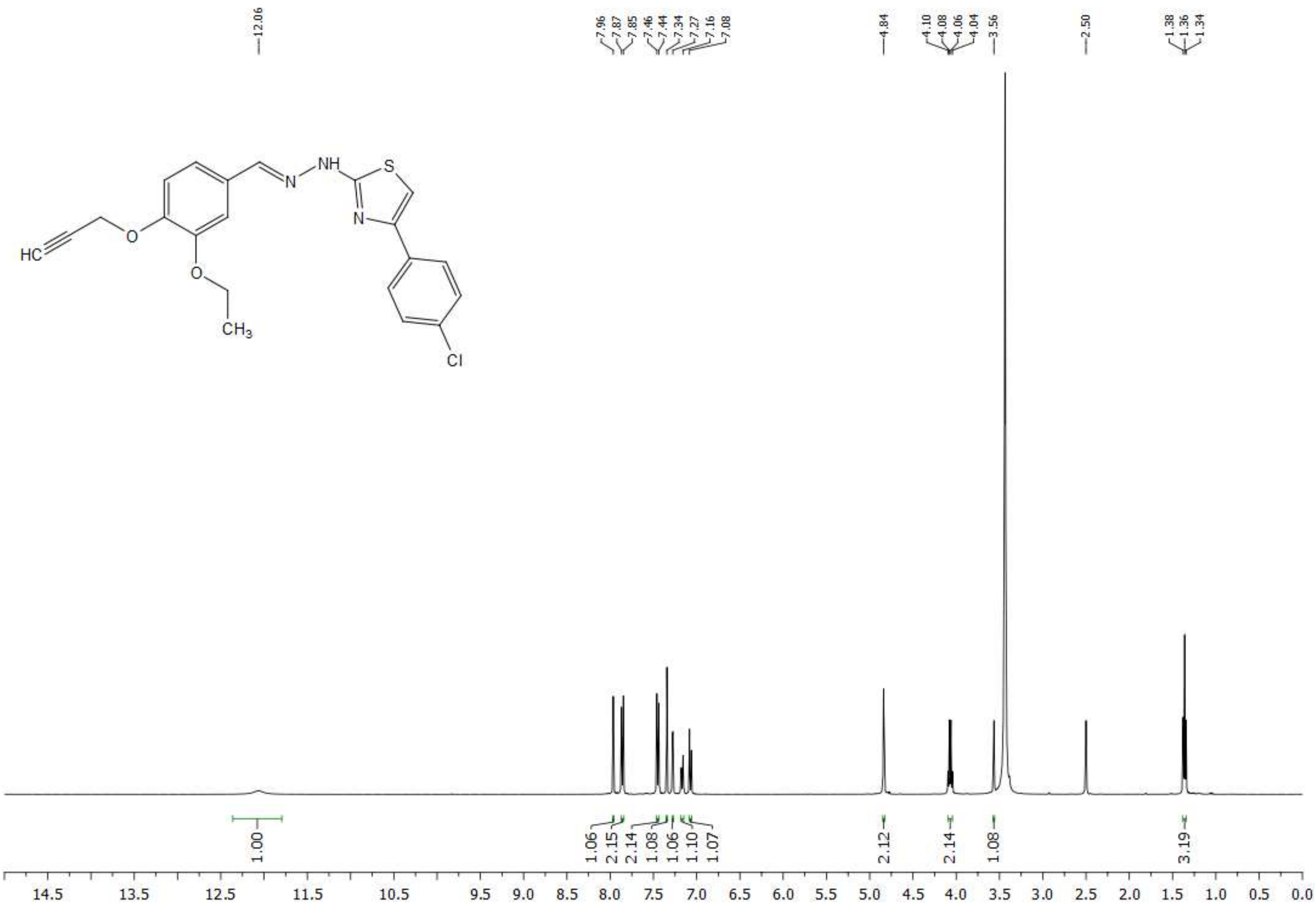

^{13}C NMR spectrum of (*E*)-4-(4-Chlorophenyl)-2-(2-(3-ethoxy-4-(prop-2-yn-1-yloxy)benzylidene)hydrazinyl)thiazole (18)

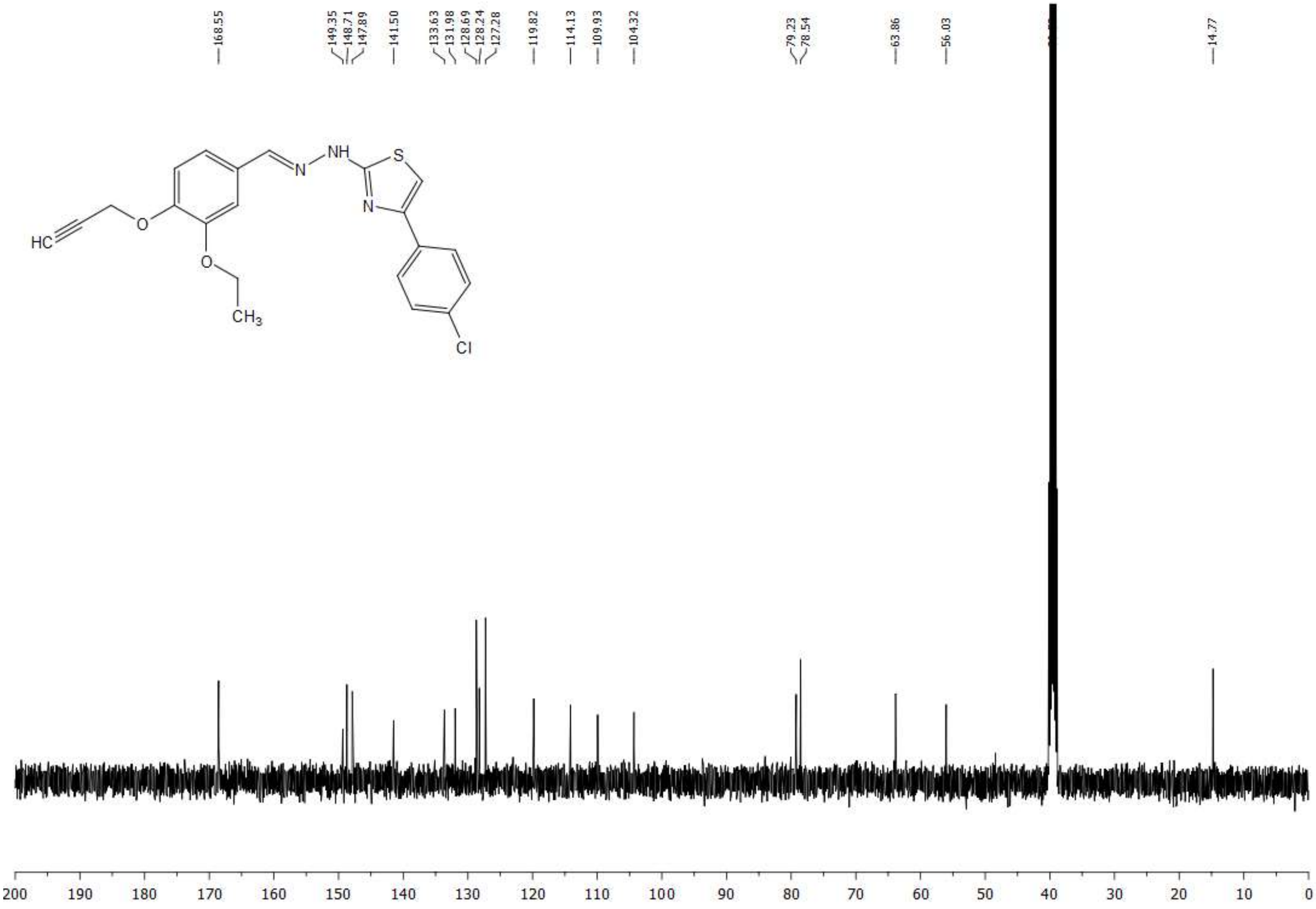

FT-IR spectrum of (*E*)-4-(4-Chlorophenyl)-2-(2-(3-ethoxy-4-(prop-2-yn-1-yloxy)benzylidene)hydrazinyl)thiazole (18)

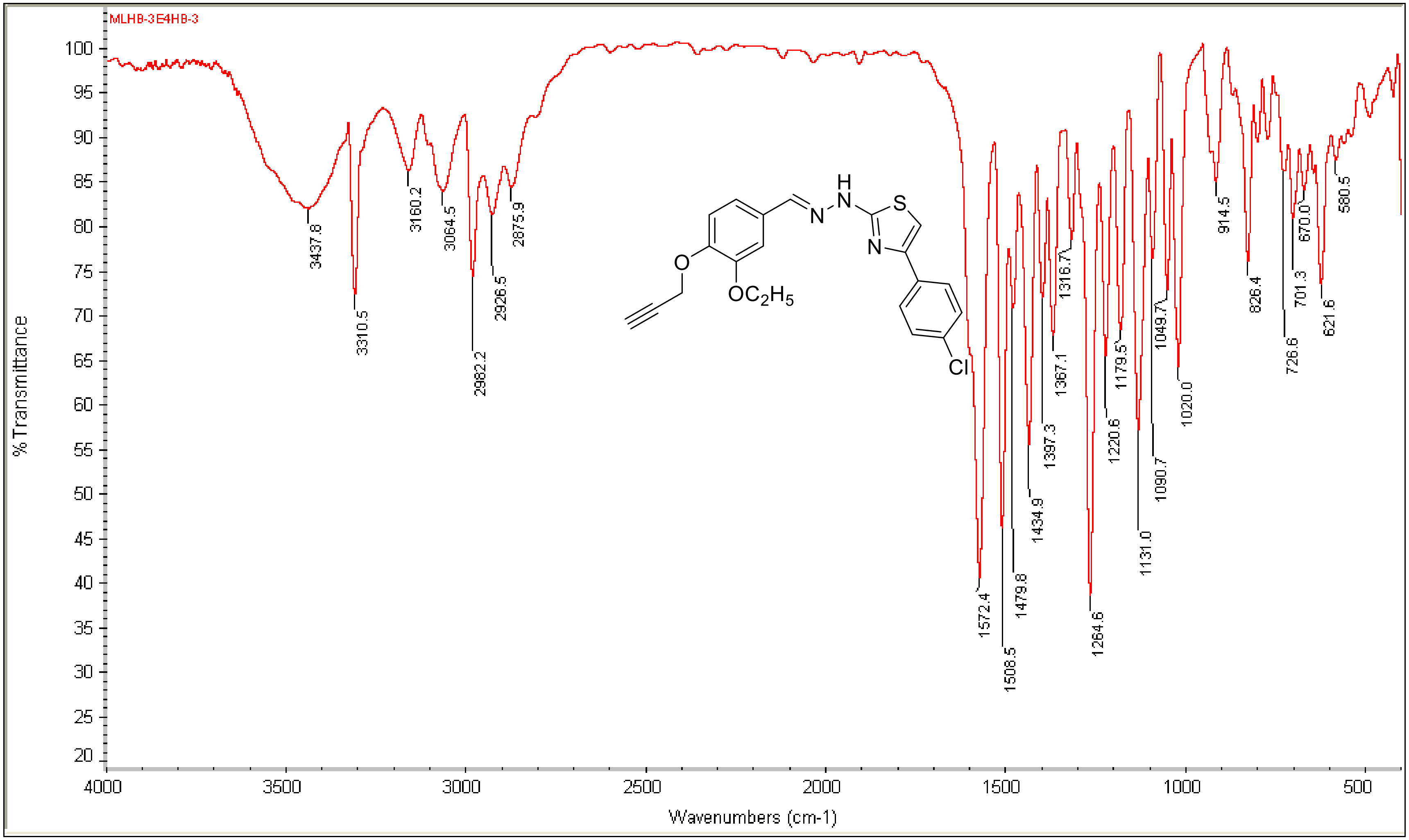

Mass spectrum of (*E*)-4-(3,4-Dichlorophenyl)-2-(2-(3-ethoxy-4-(prop-2-yn-1-yloxy)benzylidene)hydrazinyl)thiazole (19)

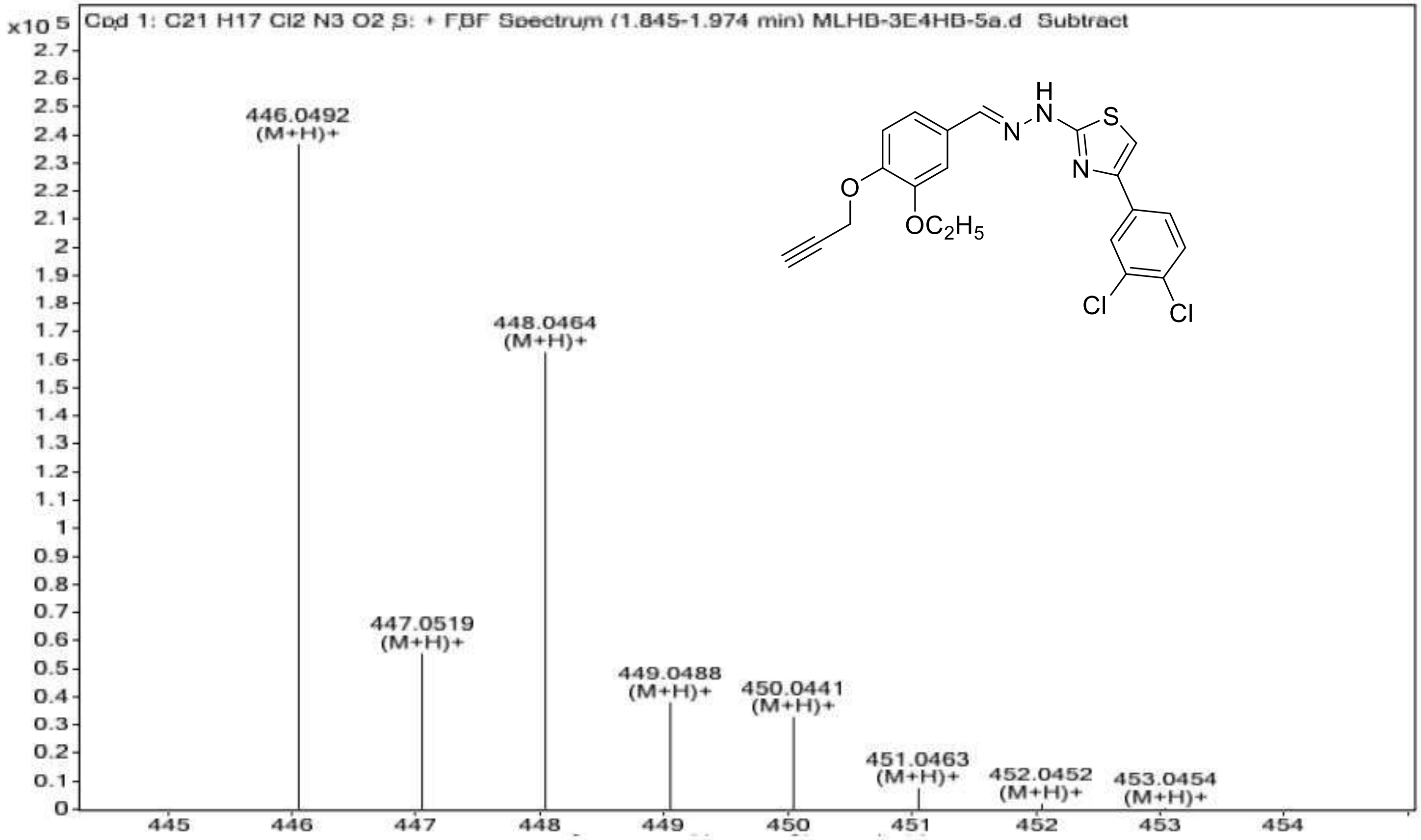

^{1}H NMR spectrum of (*E*)-4-(3,4-Dichlorophenyl)-2-(2-(3-ethoxy-4-(prop-2-yn-1-yloxy)benzylidene)hydrazinyl)thiazole (19)

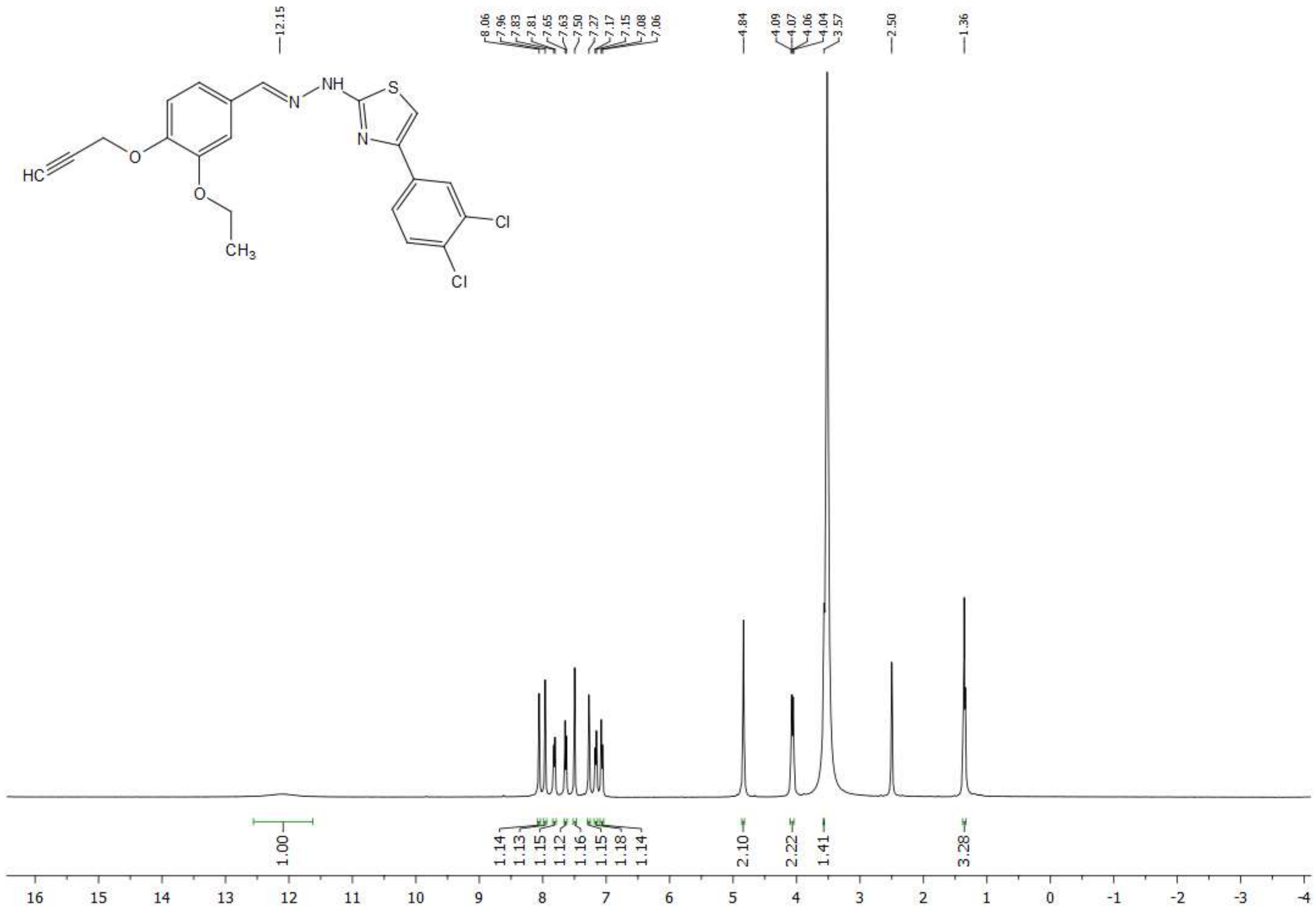

^{13}C NMR spectrum of (*E*)-4-(3,4-Dichlorophenyl)-2-(2-(3-ethoxy-4-(prop-2-yn-1-yloxy)benzylidene)hydrazinyl)thiazole (19)

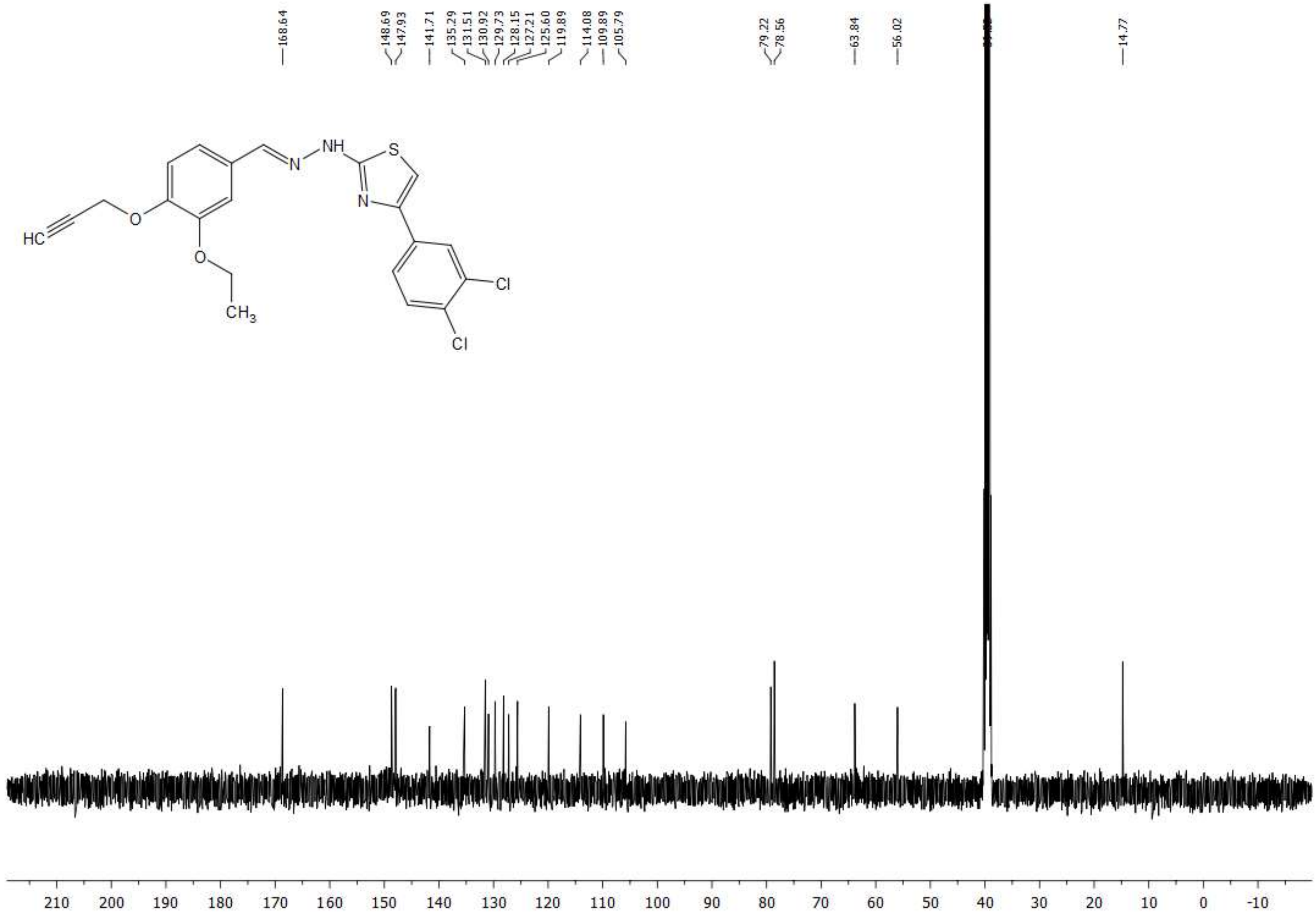

FT-IR spectrum of (*E*)-4-(3,4-Dichlorophenyl)-2-(2-(3-ethoxy-4-(prop-2-yn-1-yloxy)benzylidene)hydrazinyl)thiazole (19)

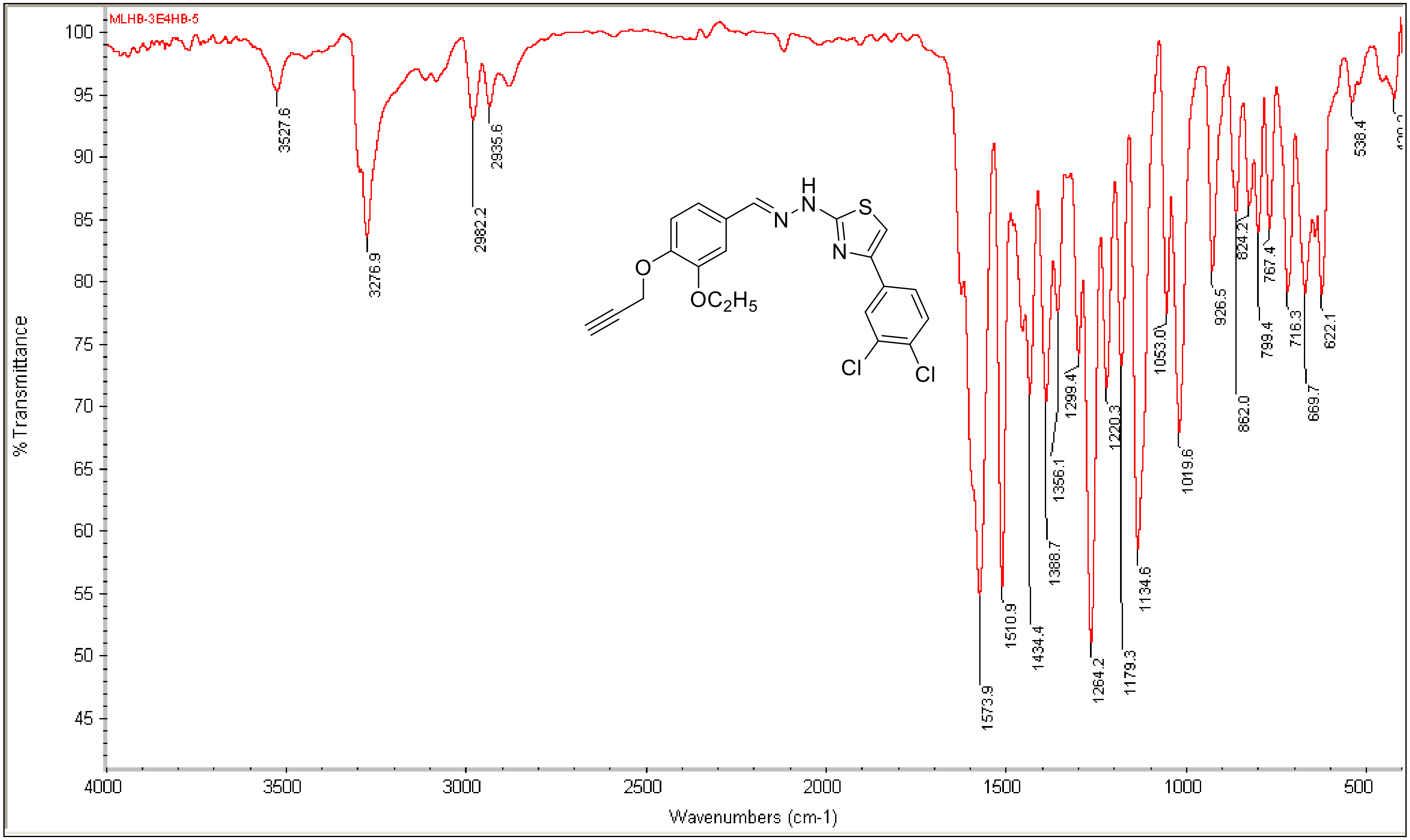

Mass spectrum of (*E*)-4-(4-Bromophenyl)-2-(2-(3-ethoxy-4-(prop-2-yn-1-yloxy)benzylidene)hydrazinyl)thiazole (20)

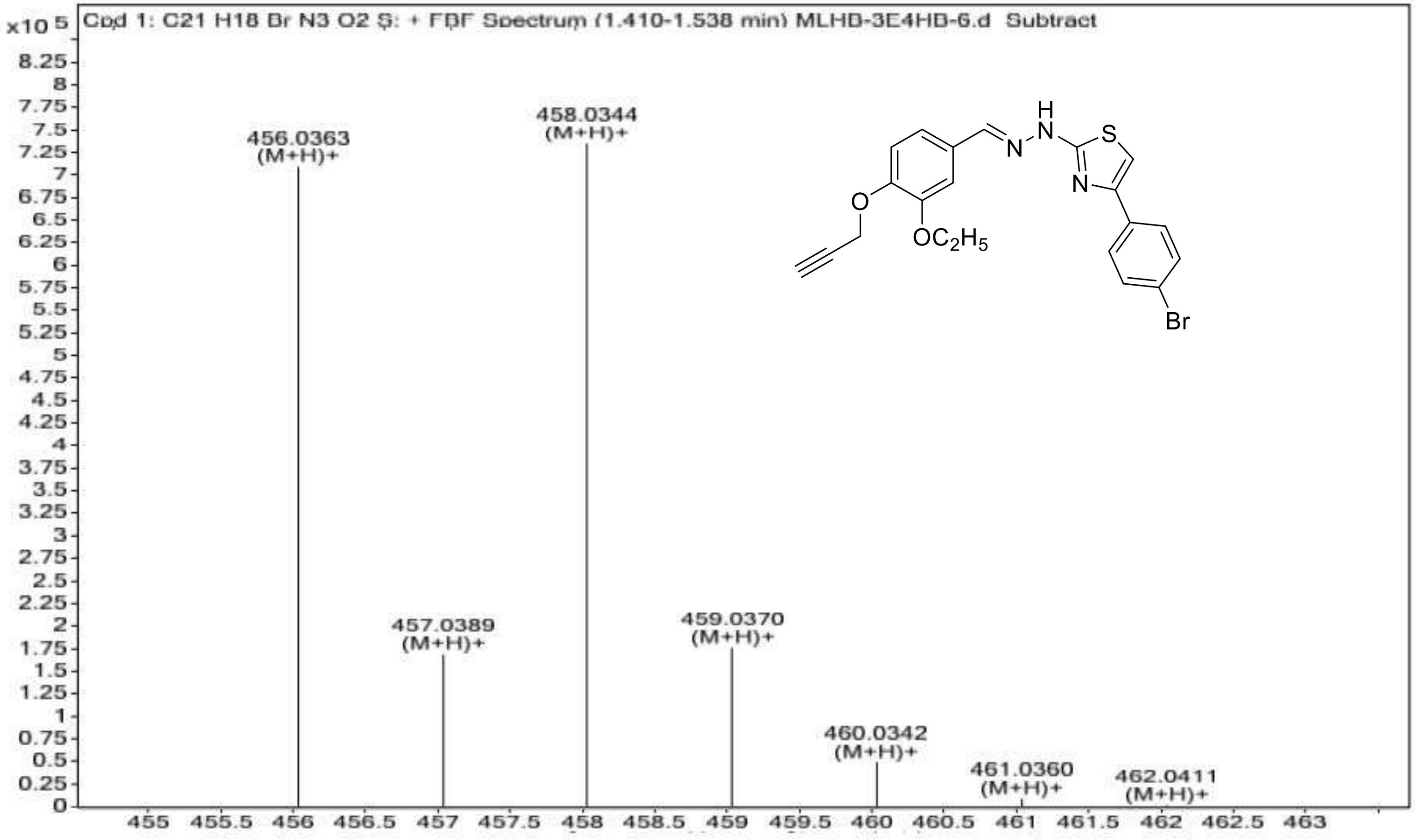

^{1}H NMR spectrum of (*E*)-4-(4-Bromophenyl)-2-(2-(3-ethoxy-4-(prop-2-yn-1-yloxy)benzylidene)hydrazinyl)thiazole (20)

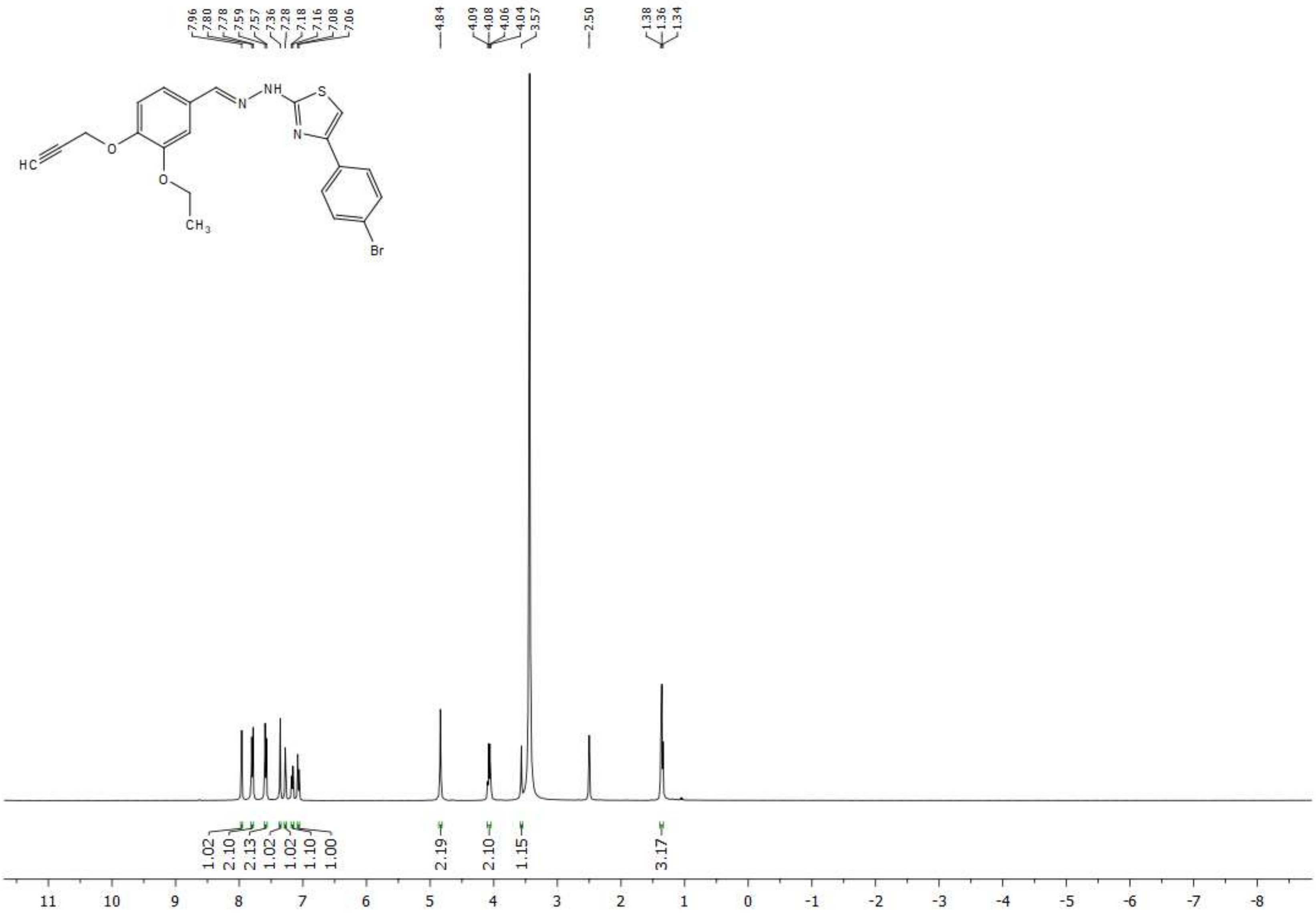

^{13}C NMR spectrum of (*E*)-4-(4-Bromophenyl)-2-(2-(3-ethoxy-4-(prop-2-yn-1-yloxy)benzylidene)hydrazinyl)thiazole (20)

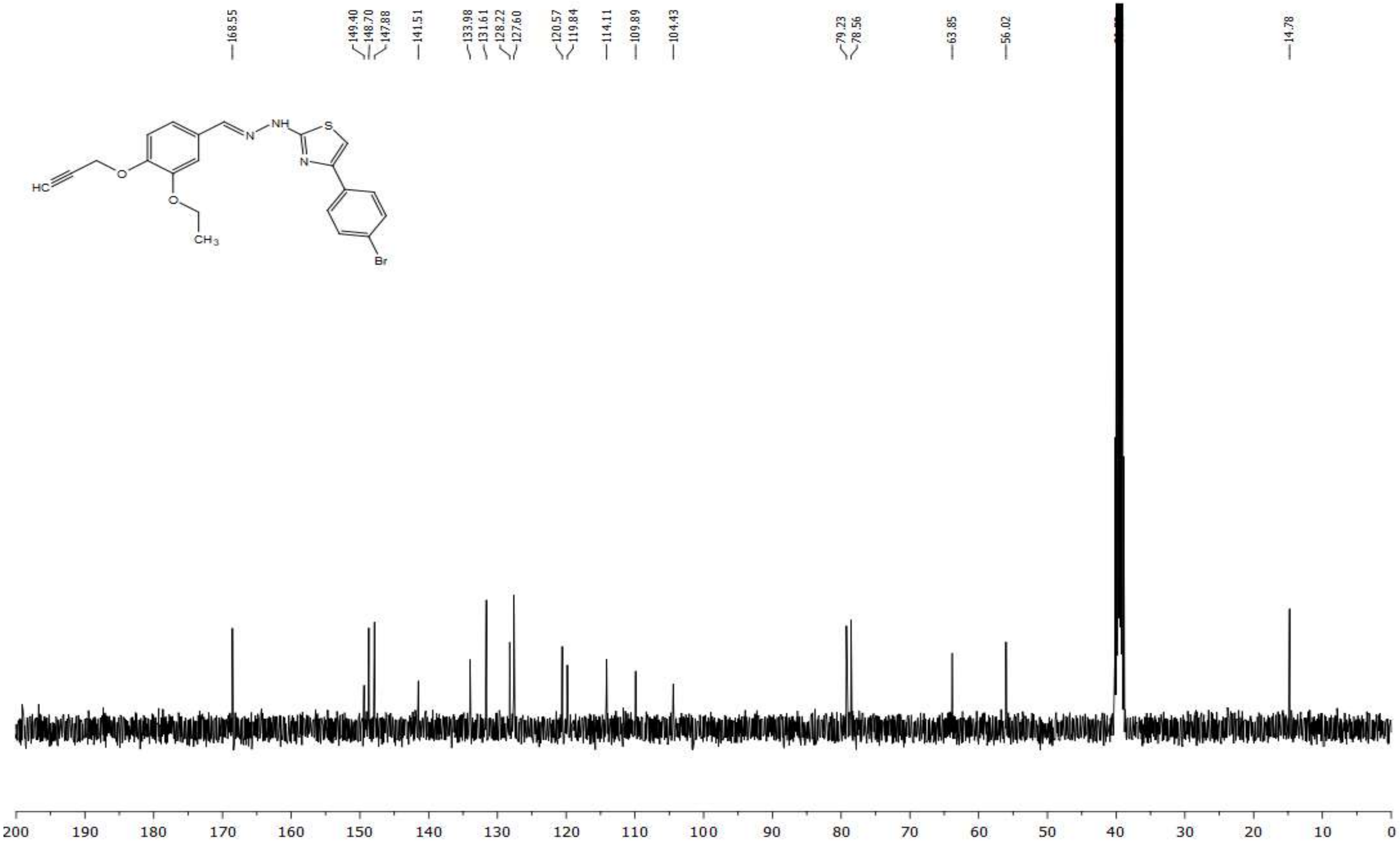

FT-IR spectrum of (*E*)-4-(4-Bromophenyl)-2-(2-(3-ethoxy-4-(prop-2-yn-1-yloxy)benzylidene)hydrazinyl)thiazole (20)

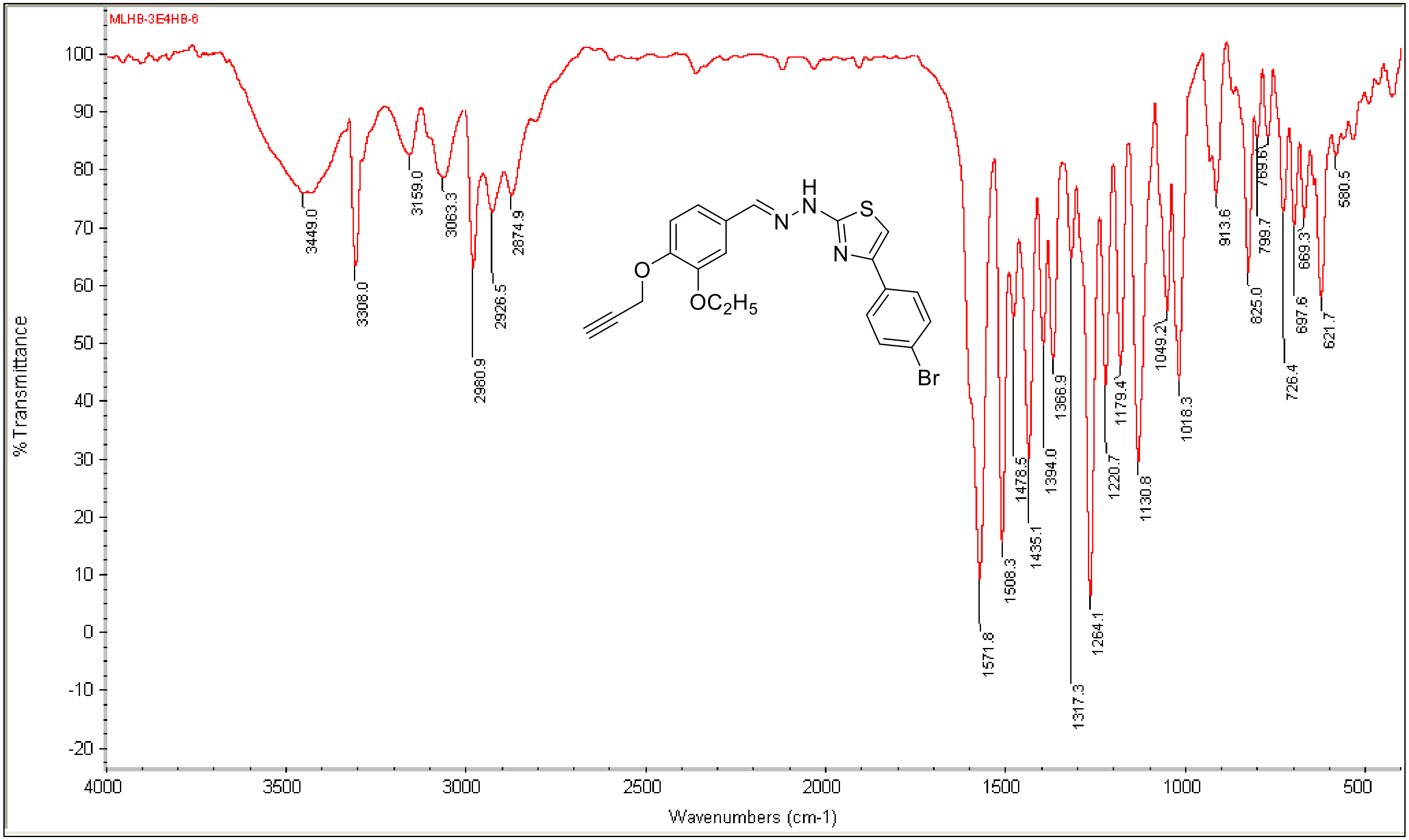

Mass spectrum of (*E*)-2-(2-(3-Ethoxy-4-(prop-2-yn-1-yloxy)benzylidene)hydrazinyl)-4-(4-nitrophenyl)thiazole (21)

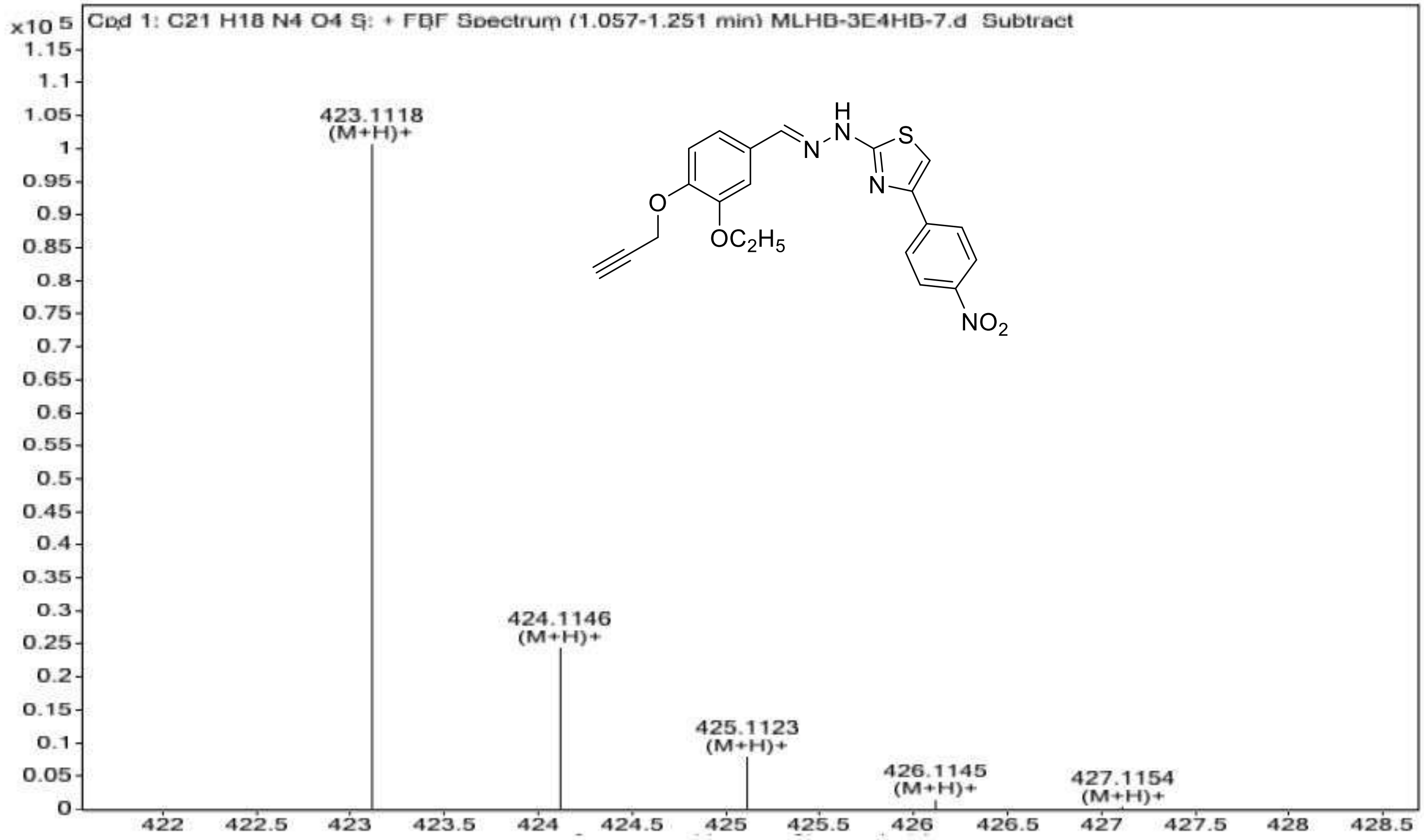

^{1}H NMR spectrum of (*E*)-2-(2-(3-Ethoxy-4-(prop-2-yn-1-yloxy)benzylidene)hydrazinyl)-4-(4-nitrophenyl)thiazole (21)

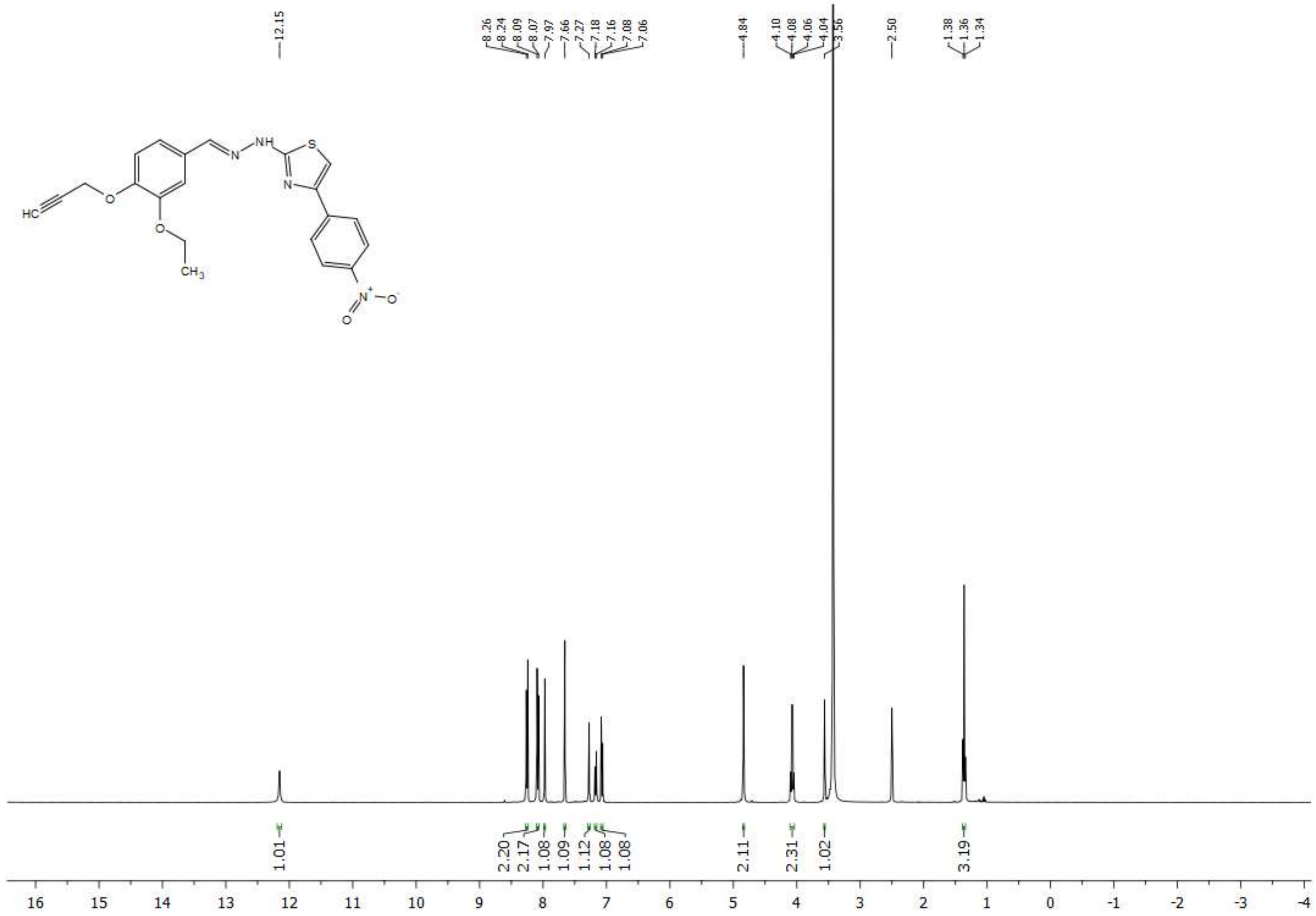

^{13}C NMR spectrum of (*E*)-2-(2-(3-Ethoxy-4-(prop-2-yn-1-yloxy)benzylidene)hydrazinyl)-4-(4-nitrophenyl)thiazole (21)

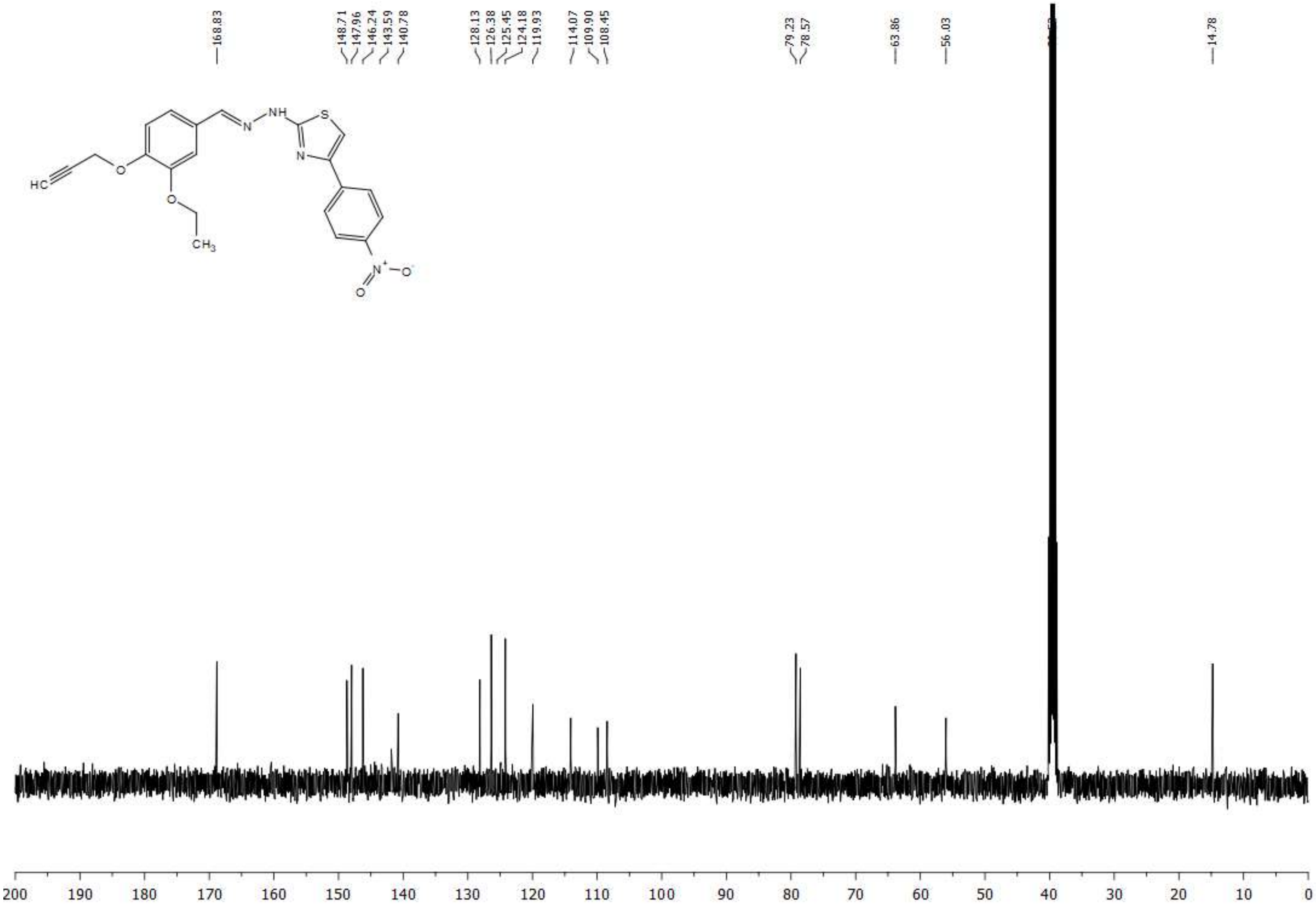

FT-IR spectrum of (*E*)-2-(2-(3-Ethoxy-4-(prop-2-yn-1-yloxy)benzylidene)hydrazinyl)-4-(4-nitrophenyl)thiazole (21)

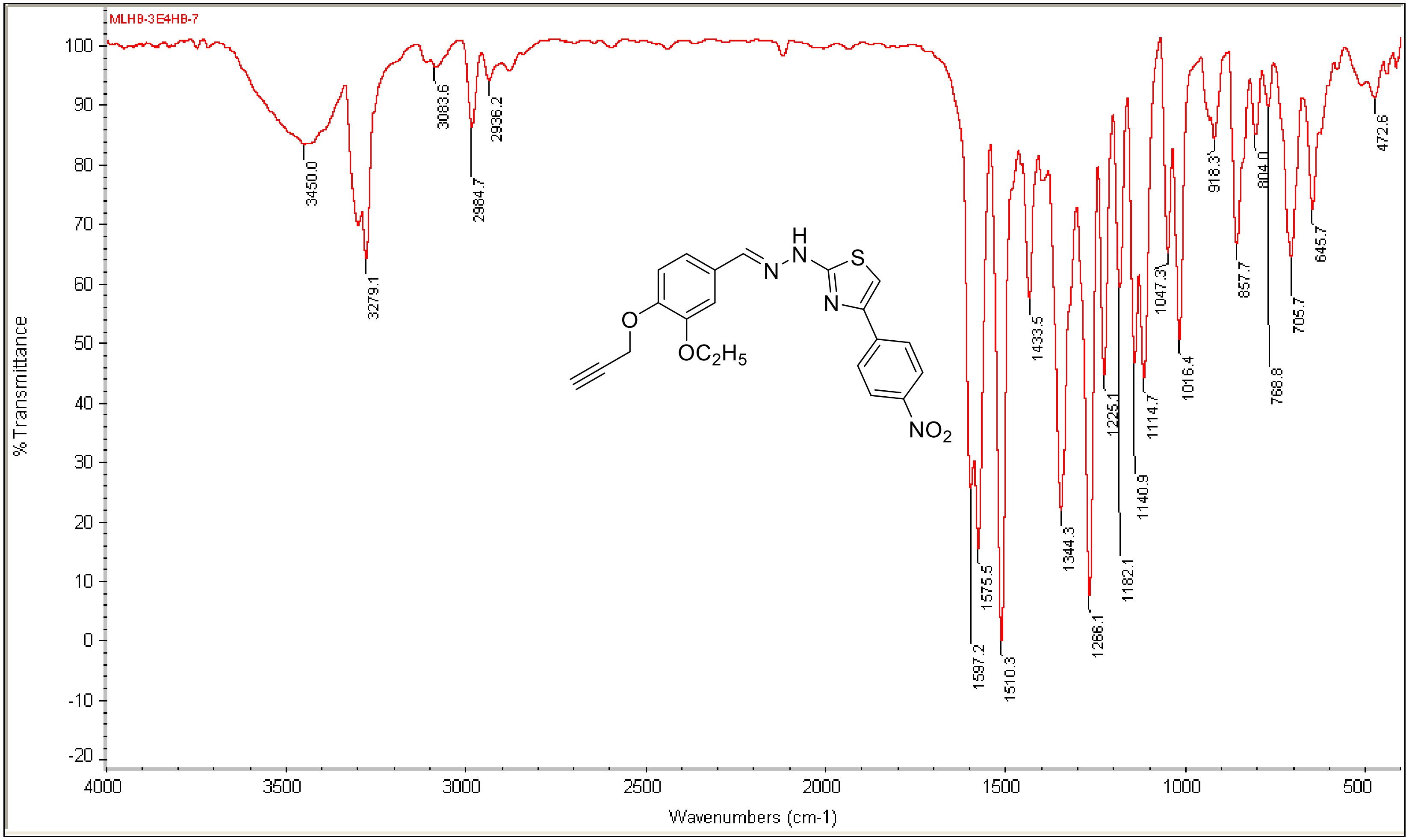

Mass spectrum of (*E*)-4-(2-(2-(3-Ethoxy-4-(prop-2-yn-1-yloxy)benzylidene)hydrazinyl)thiazol-4-yl)benzonitrile (22)

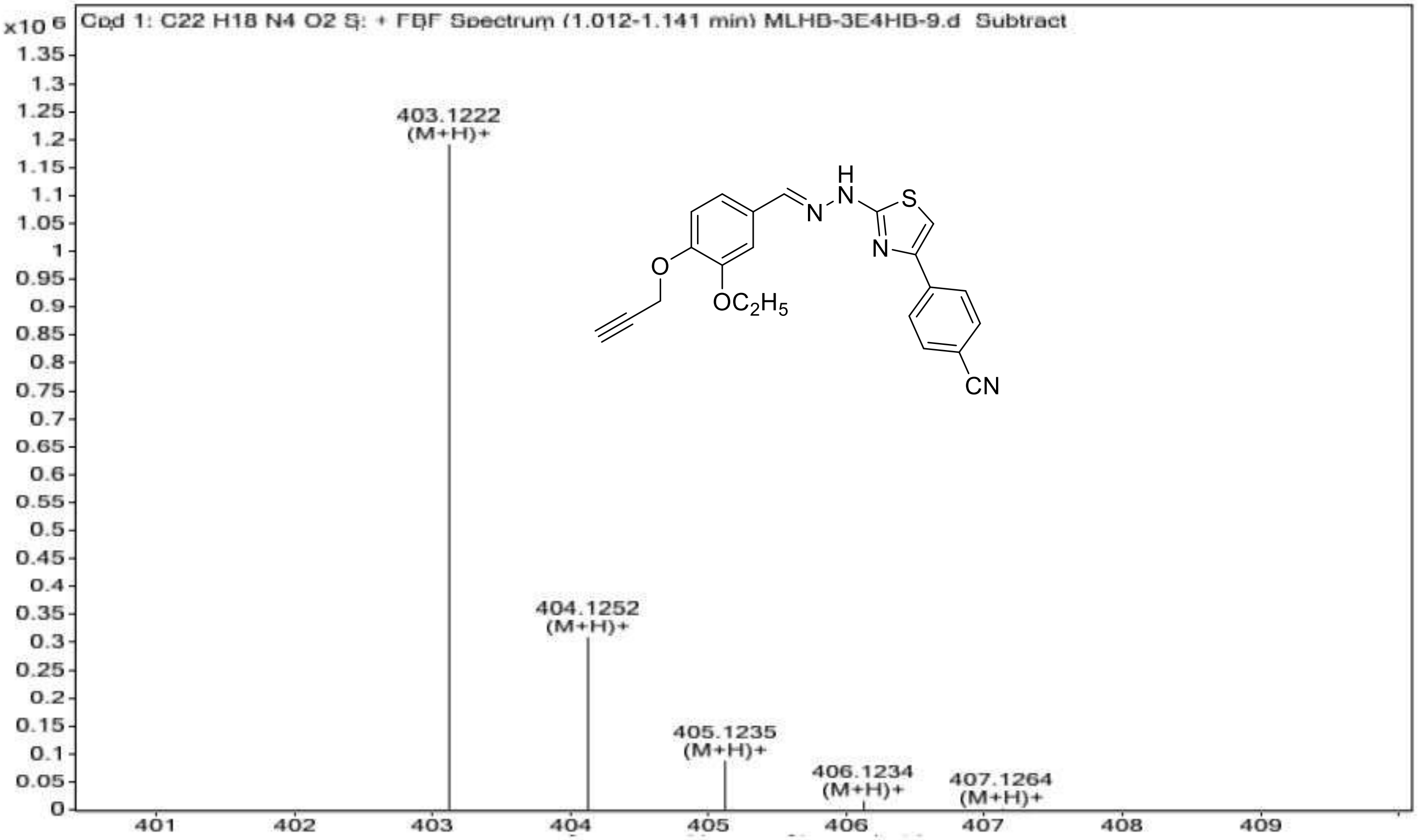

^{1}H NMR spectrum of (*E*)-4-(2-(2-(3-Ethoxy-4-(prop-2-yn-1-yloxy)benzylidene)hydrazinyl)thiazol-4-yl)benzonitrile (22)

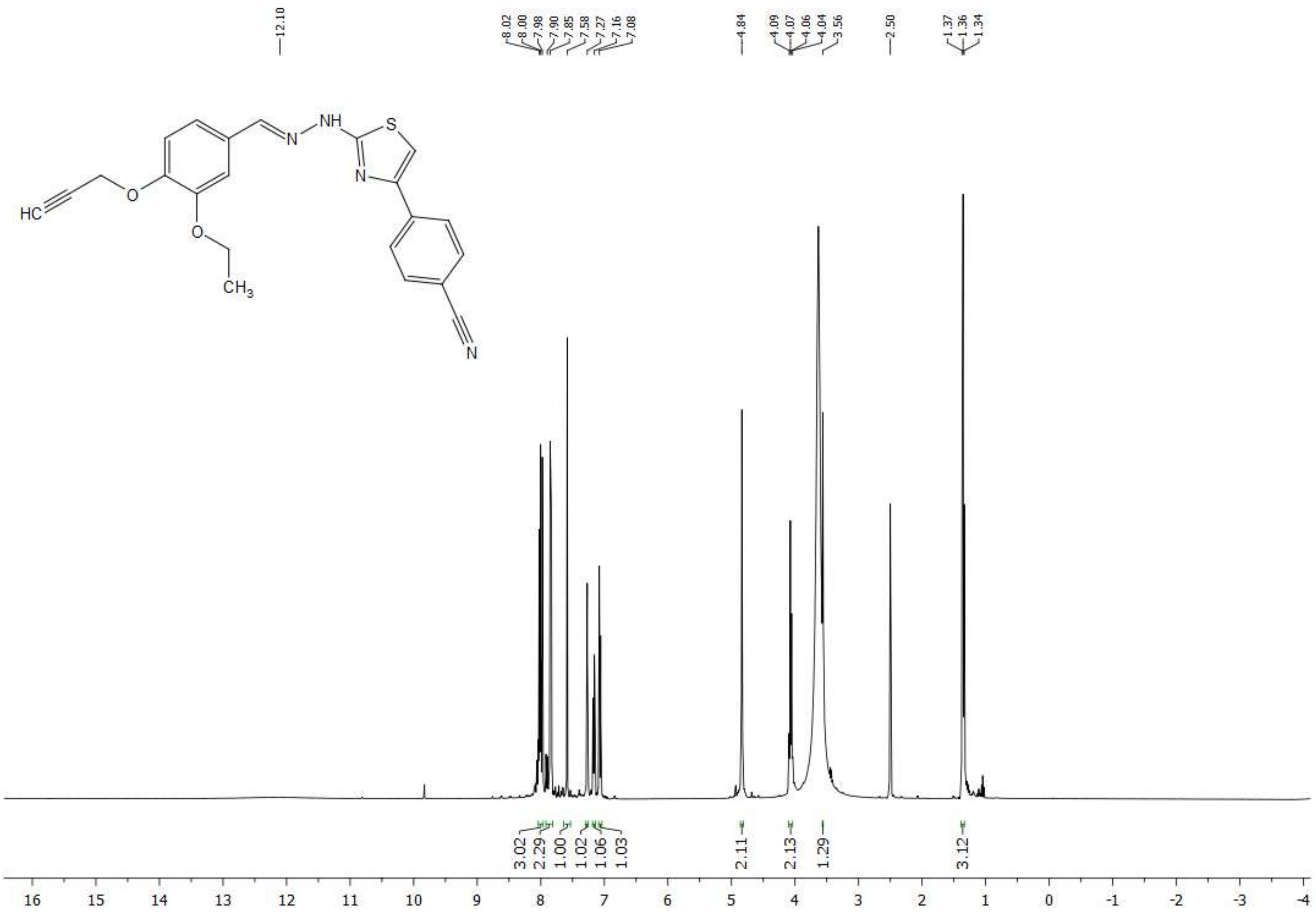

^{13}C NMR spectrum of (*E*)-4-(2-(2-(3-Ethoxy-4-(prop-2-yn-1-yloxy)benzylidene)hydrazinyl)thiazol-4-yl)benzonitrile (22)

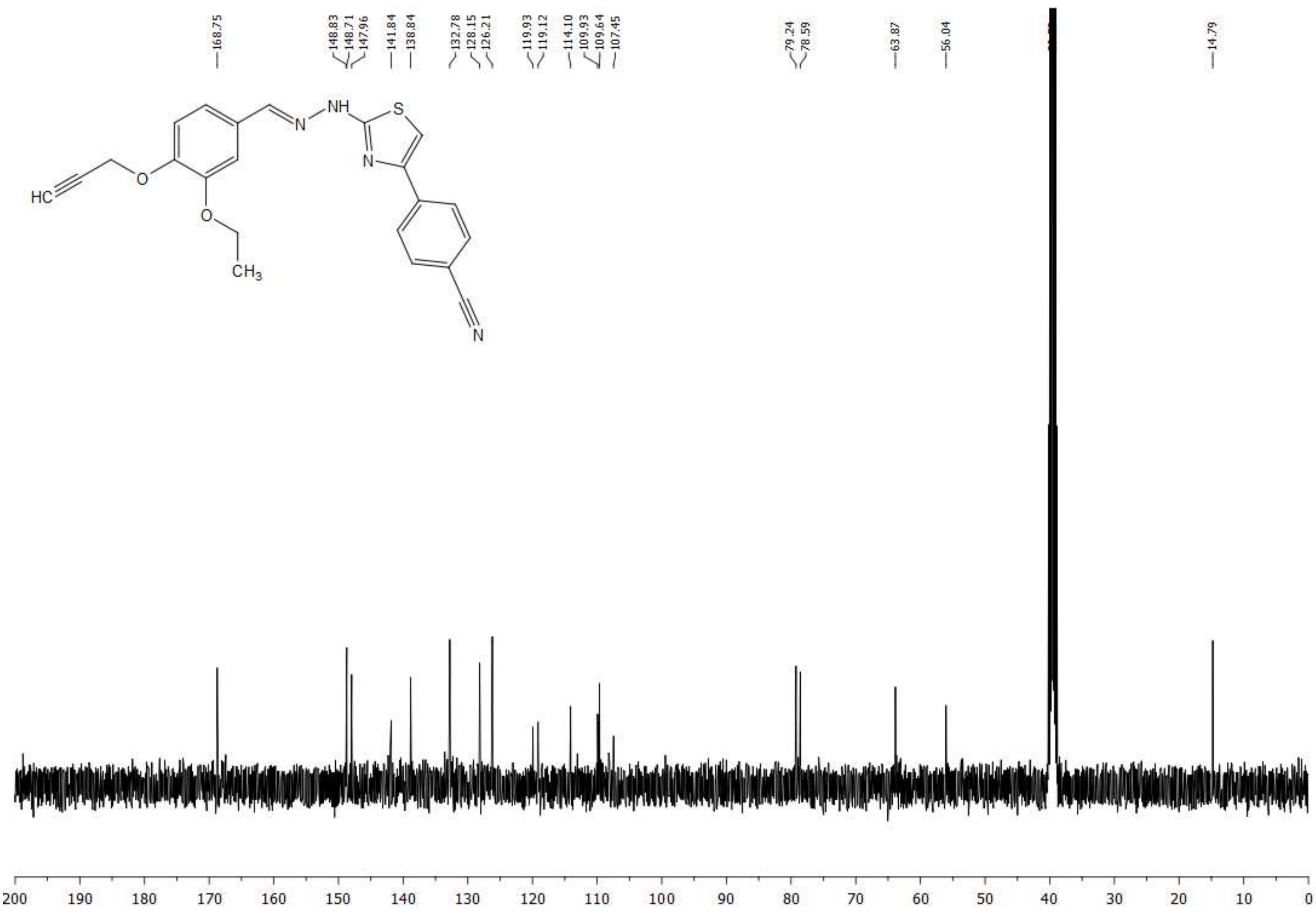

FT-IR spectrum of (*E*)-4-(2-(2-(3-Ethoxy-4-(prop-2-yn-1-yloxy)benzylidene)hydrazinyl)thiazol-4-yl)benzonitrile (22)

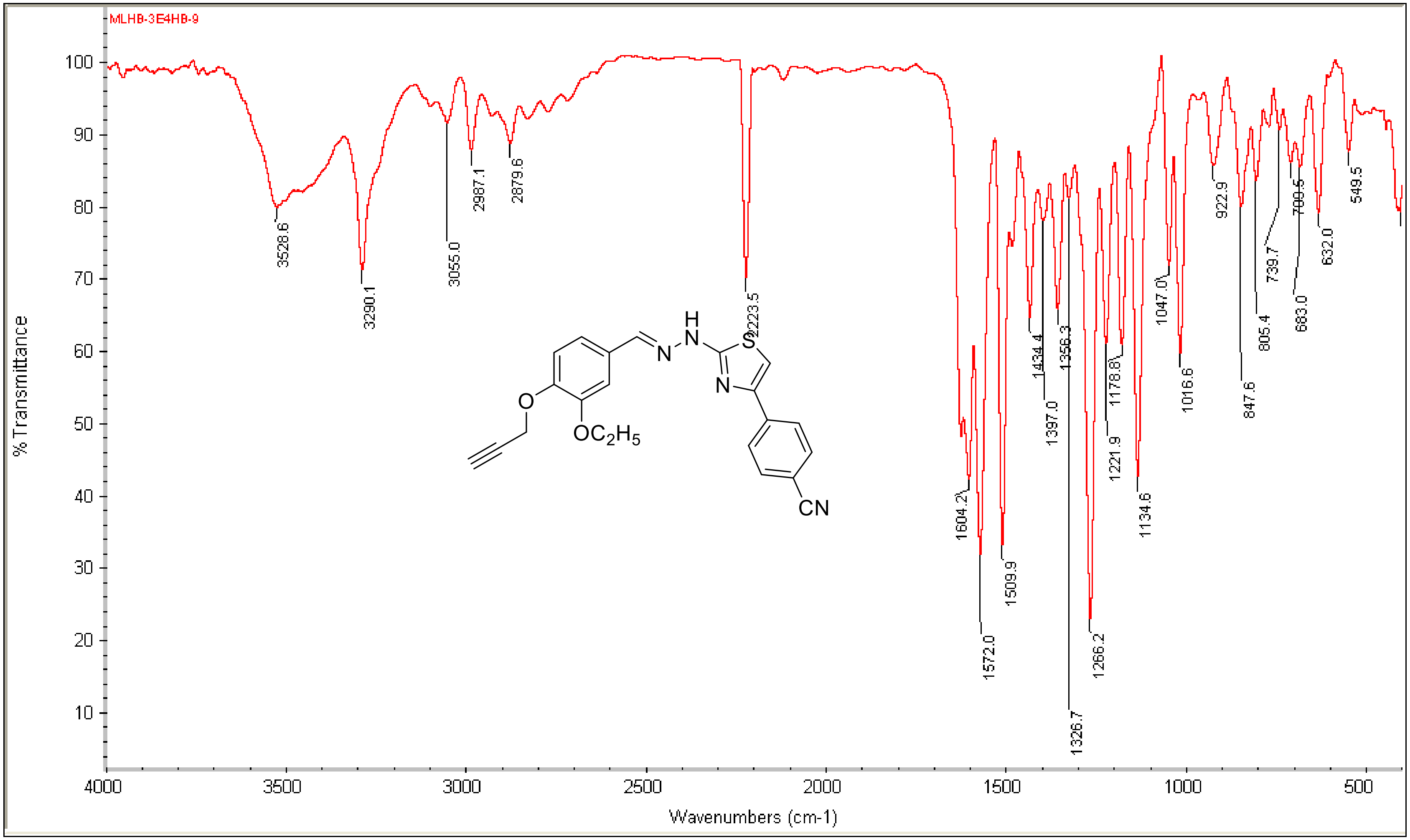

Mass spectrum of (*E*)-2-(2-(3-Ethoxy-4-(prop-2-yn-1-yloxy)benzylidene)hydrazinyl)-4-(p-tolyl)thiazole (23)

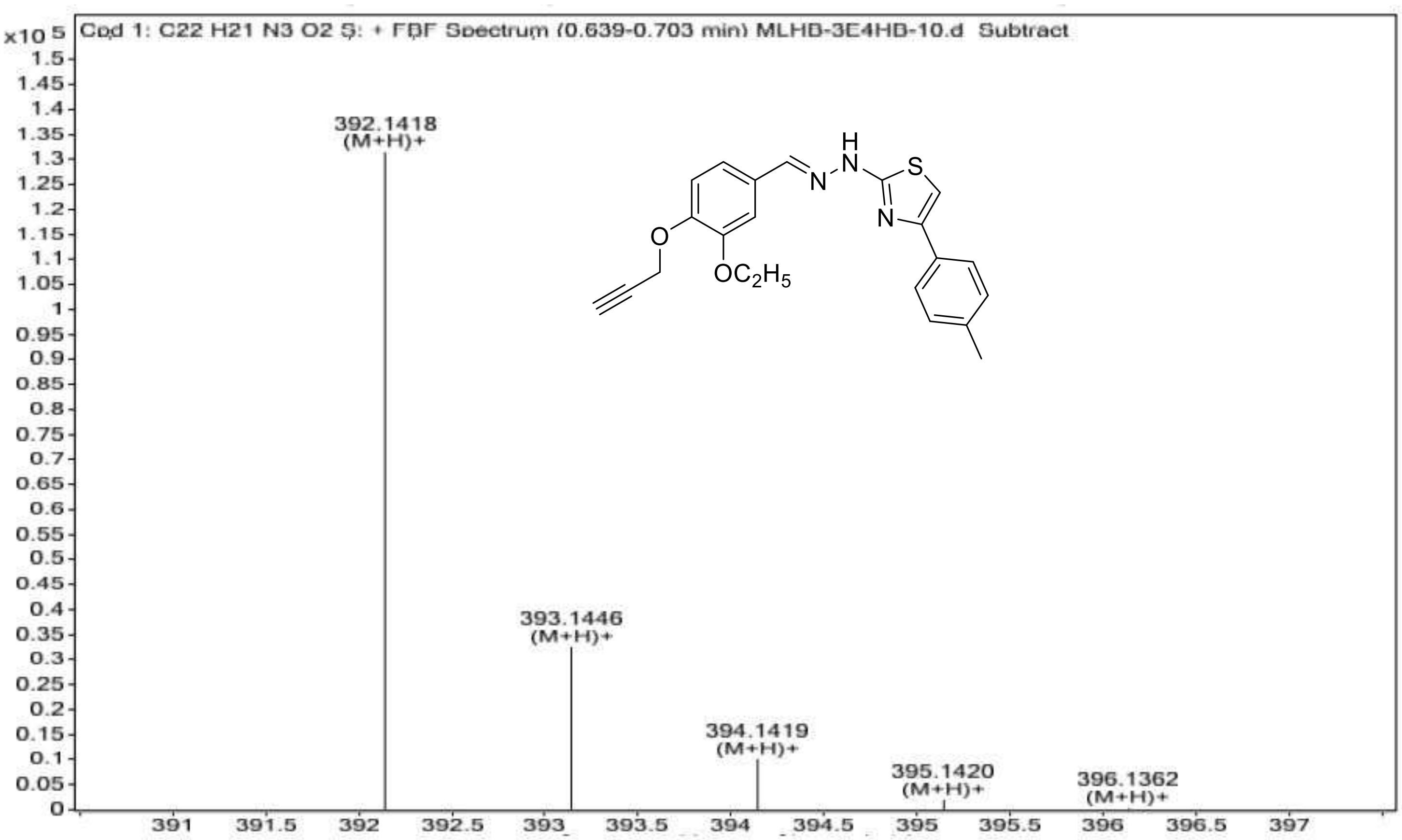

^{1}H NMR spectrum of (*E*)-2-(2-(3-Ethoxy-4-(prop-2-yn-1-yloxy)benzylidene)hydrazinyl)-4-(p-tolyl)thiazole (23)

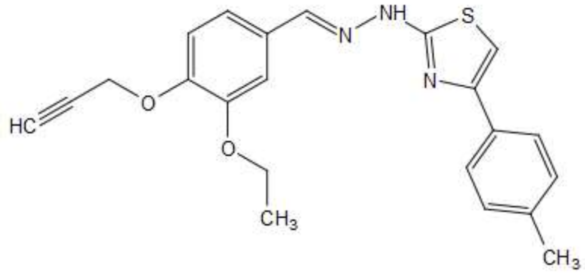

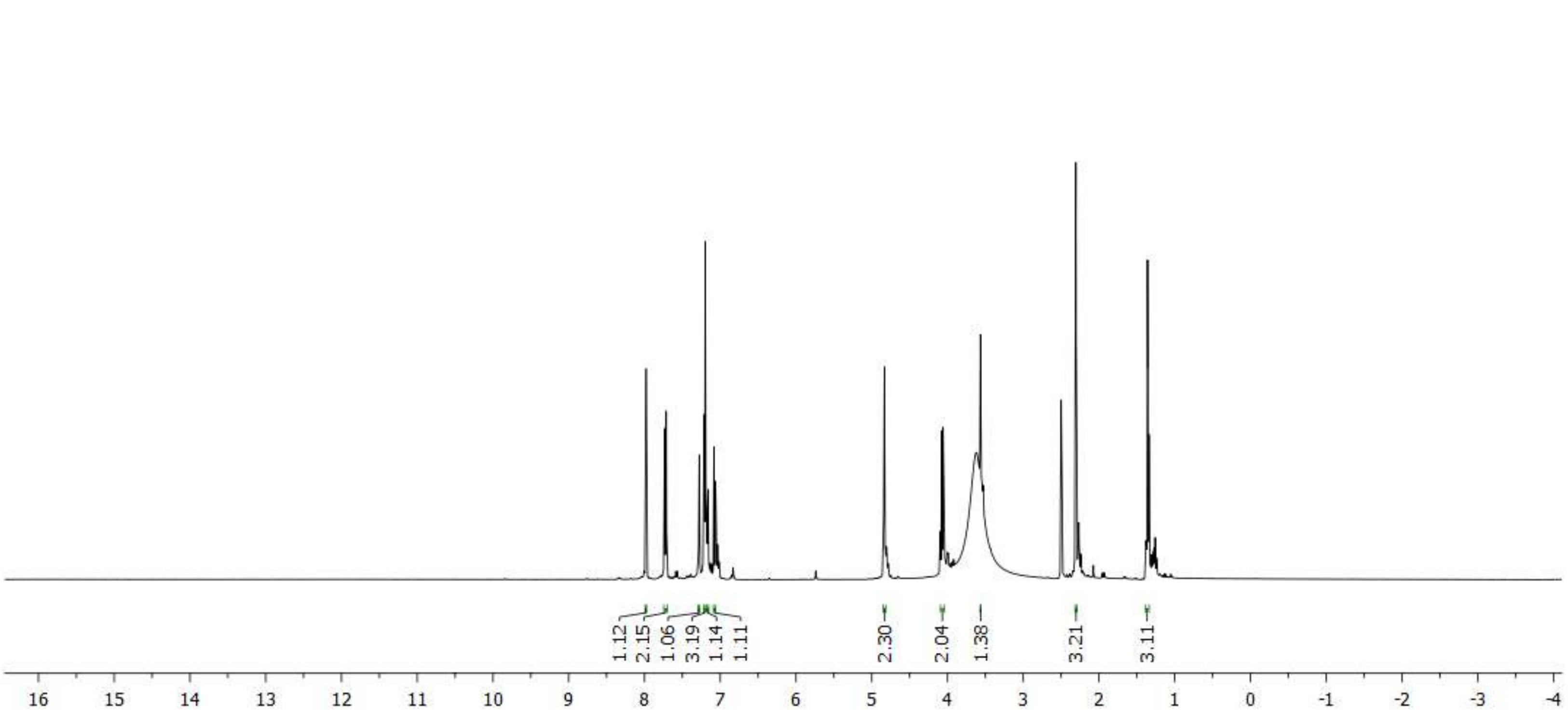

^{13}C NMR spectrum of (*E*)-2-(2-(3-Ethoxy-4-(prop-2-yn-1-yloxy)benzylidene)hydrazinyl)-4-(p-tolyl)thiazole (23)

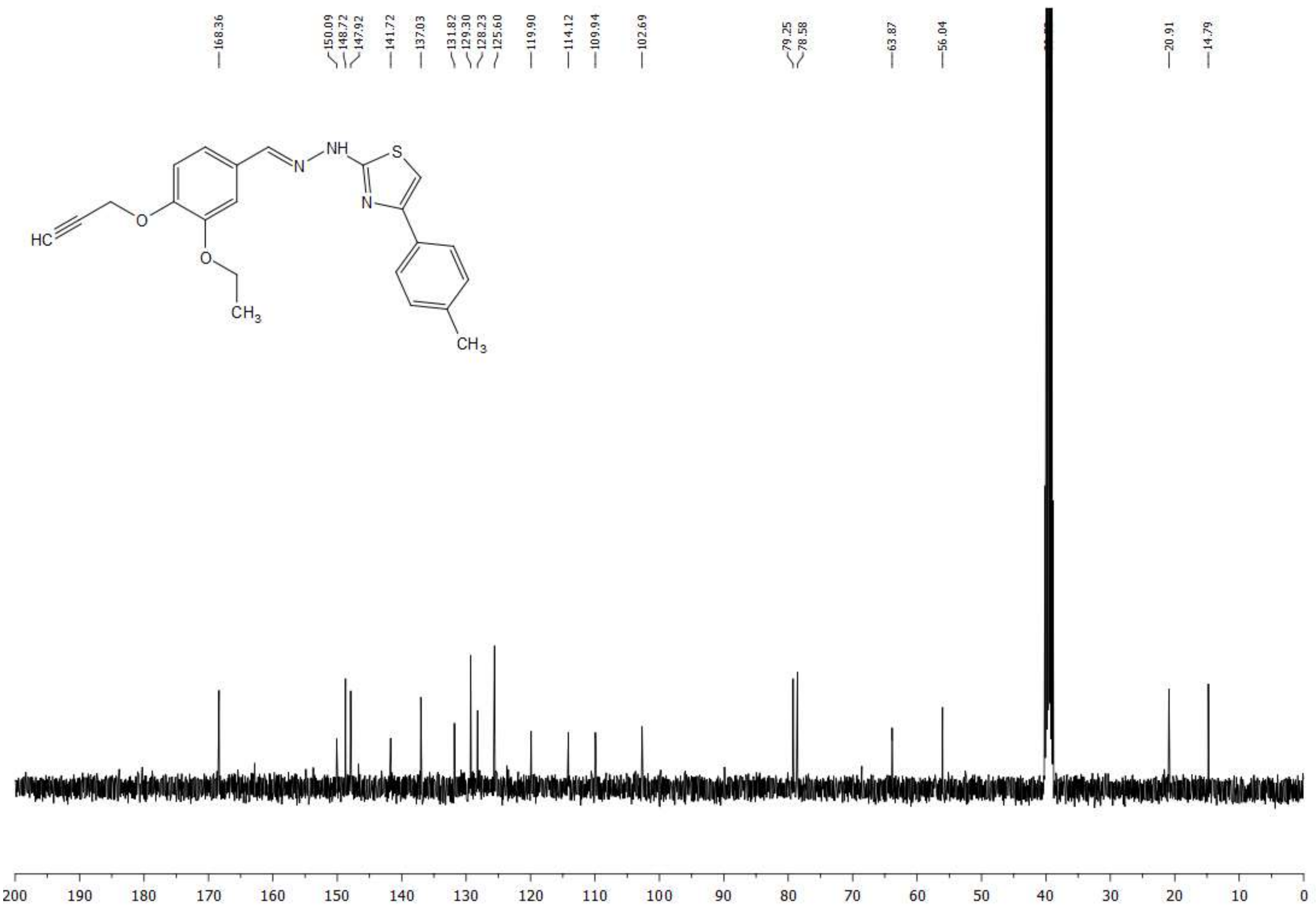

FT-IR spectrum of (*E*)-2-(2-(3-Ethoxy-4-(prop-2-yn-1-yloxy)benzylidene)hydrazinyl)-4-(p-tolyl)thiazole (23)

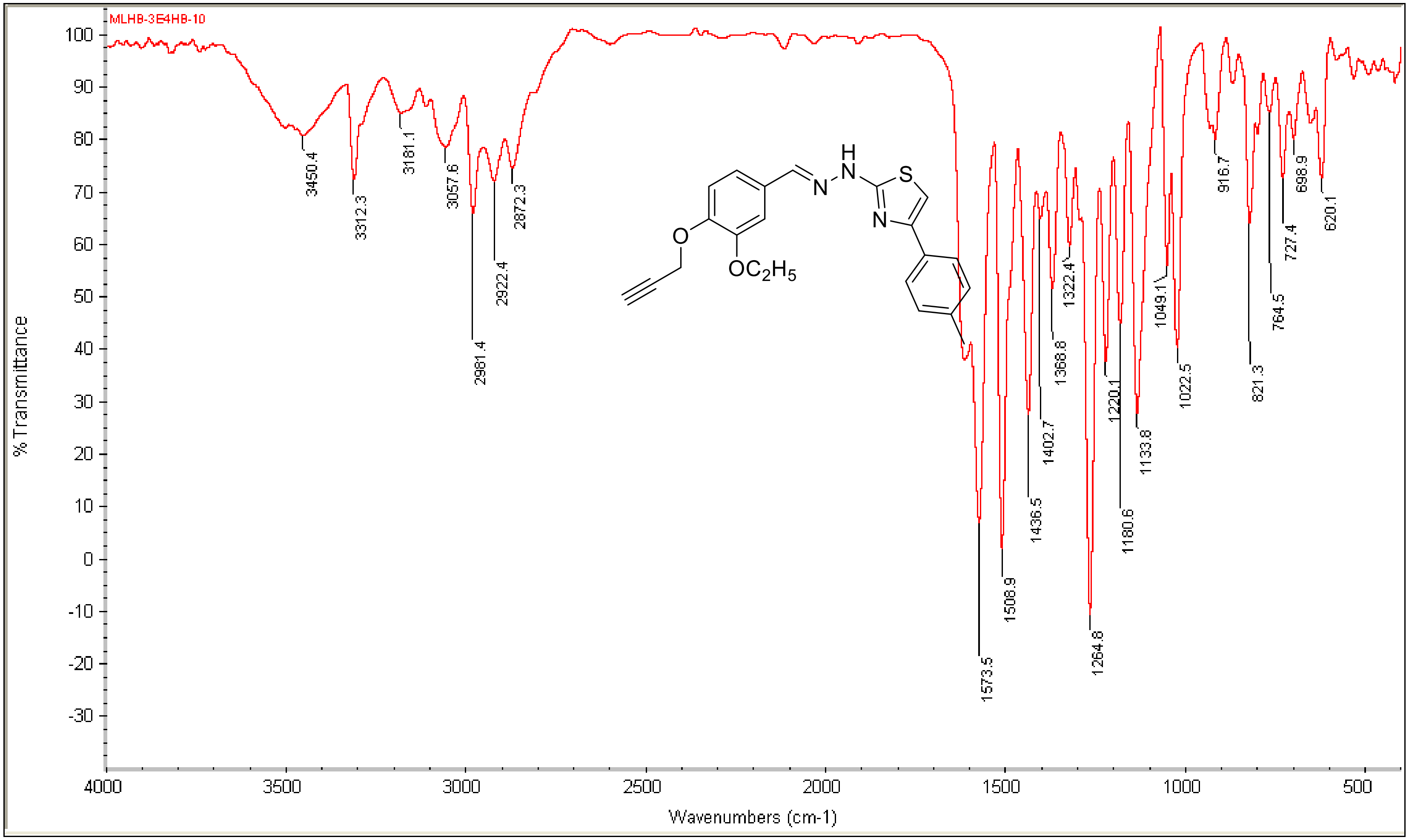

Mass spectrum of (*E*)-2-(2-(3-Ethoxy-4-(prop-2-yn-1-yloxy)benzylidene)hydrazinyl)-4-(4-methoxyphenyl)thiazole (24)

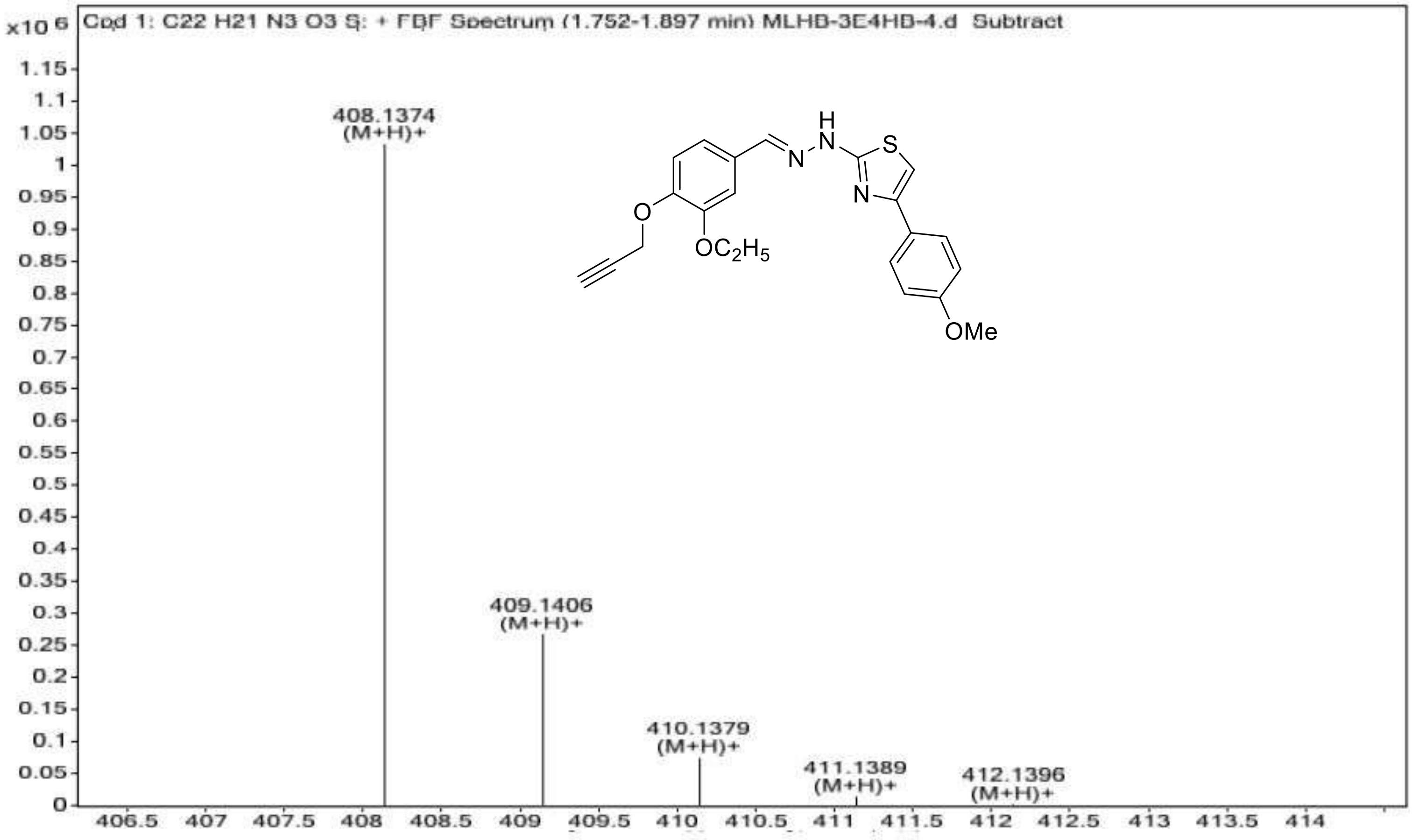

^{1}H NMR spectrum of (*E*)-2-(2-(3-Ethoxy-4-(prop-2-yn-1-yloxy)benzylidene)hydrazinyl)-4-(4-methoxyphenyl)thiazole (24)

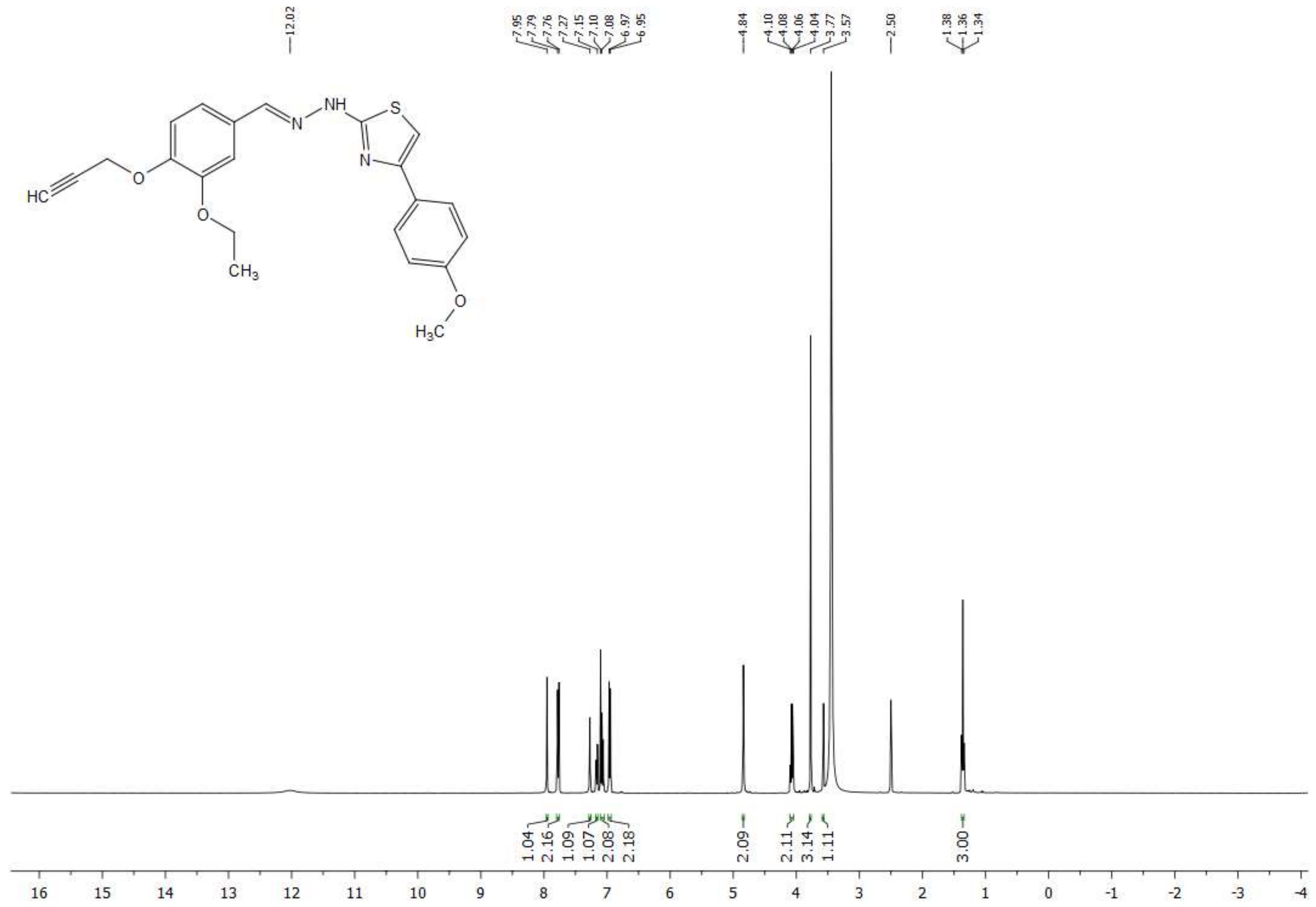

^{13}C NMR spectrum of (*E*)-2-(2-(3-Ethoxy-4-(prop-2-yn-1-yloxy)benzylidene)hydrazinyl)-4-(4-methoxyphenyl)thiazole (24)

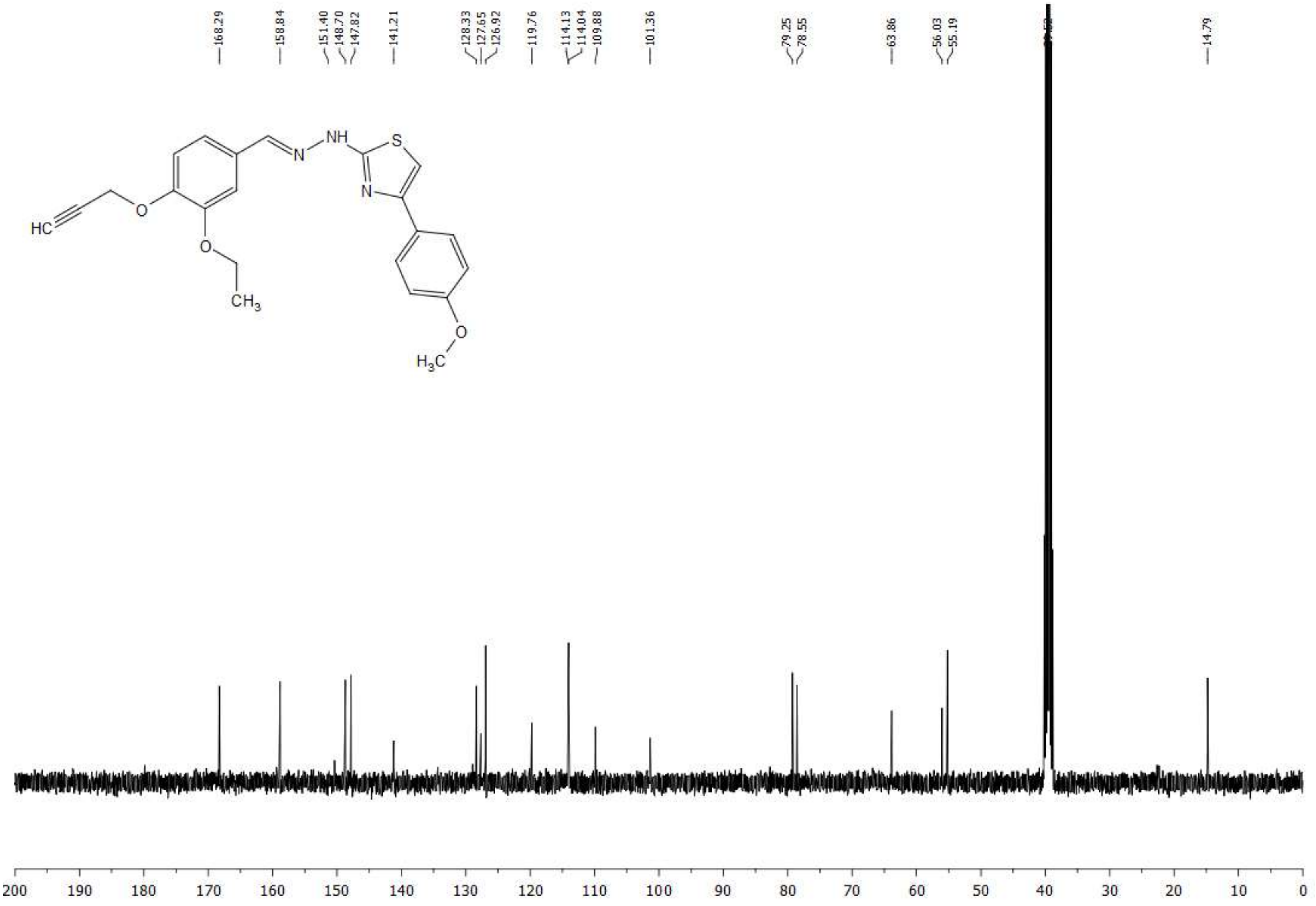

FT-IR spectrum of (*E*)-2-(2-(3-Ethoxy-4-(prop-2-yn-1-yloxy)benzylidene)hydrazinyl)-4-(4-methoxyphenyl)thiazole (24)

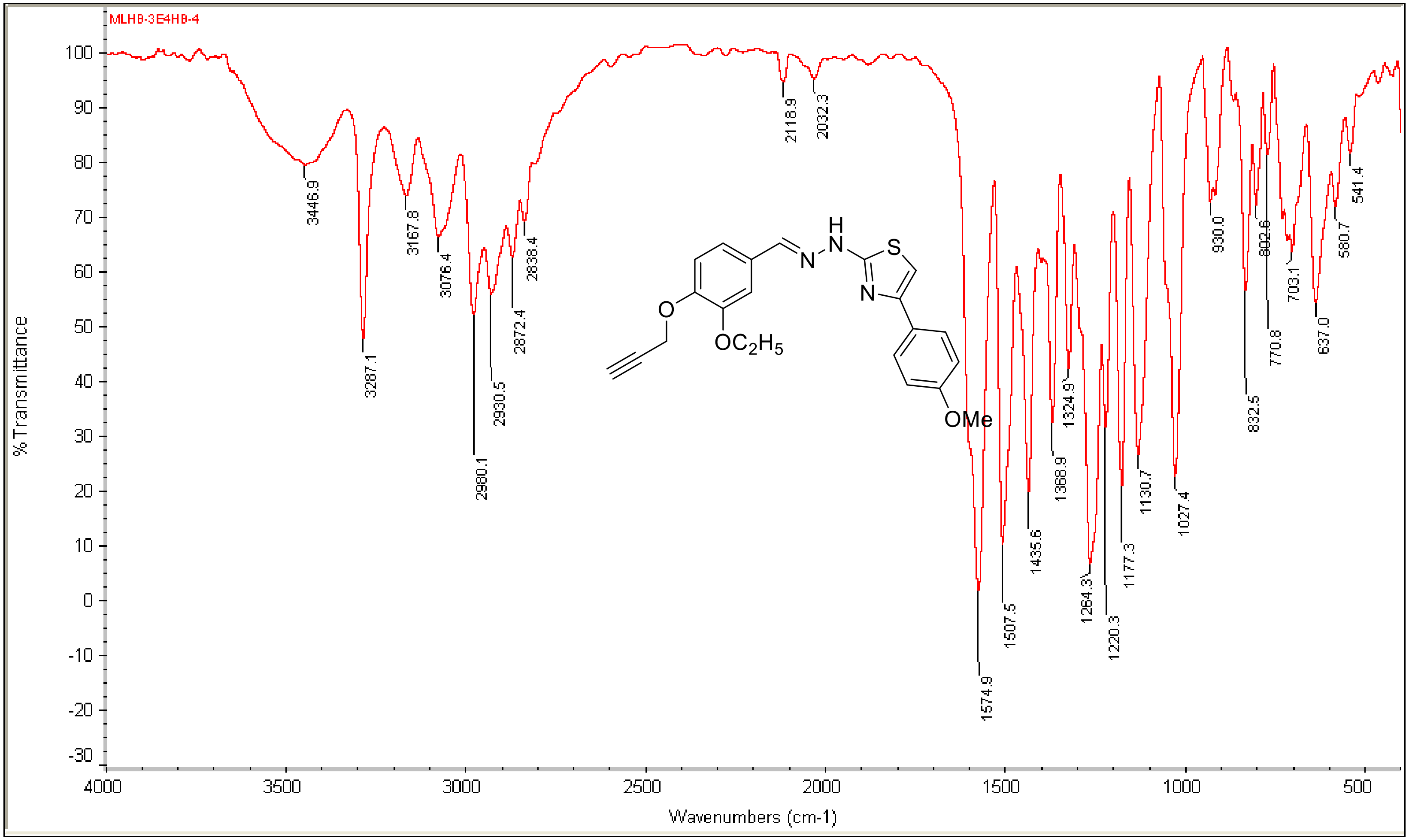

Mass spectrum of (*E*)-2-(2-(5-Bromo-2-(prop-2-yn-1-yloxy)benzylidene)hydrazinyl)-4-phenylthiazole (25)

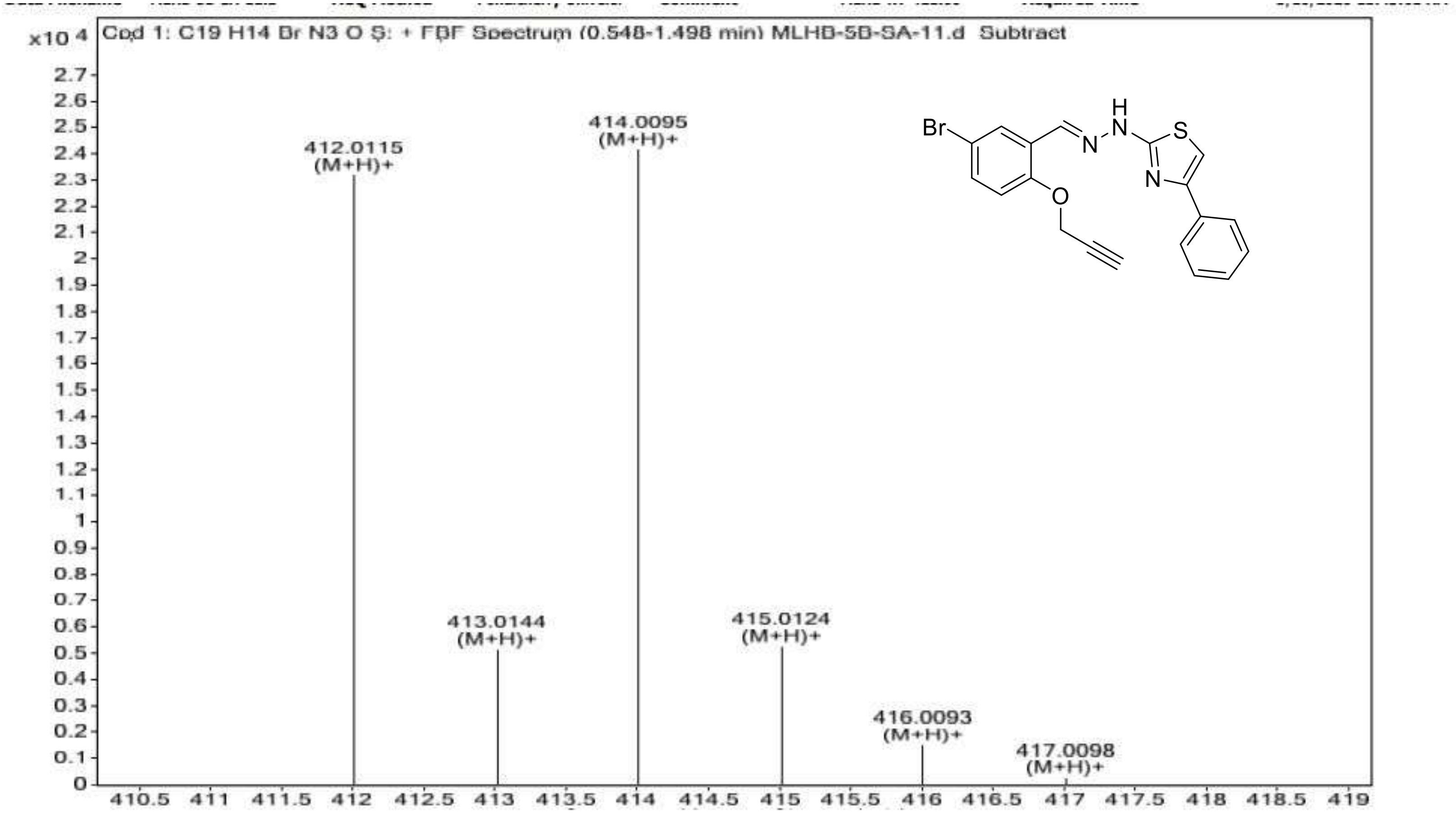

^{1}H NMR spectrum of (*E*)-2-(2-(5-Bromo-2-(prop-2-yn-1-yloxy)benzylidene)hydrazinyl)-4-phenylthiazole (25)

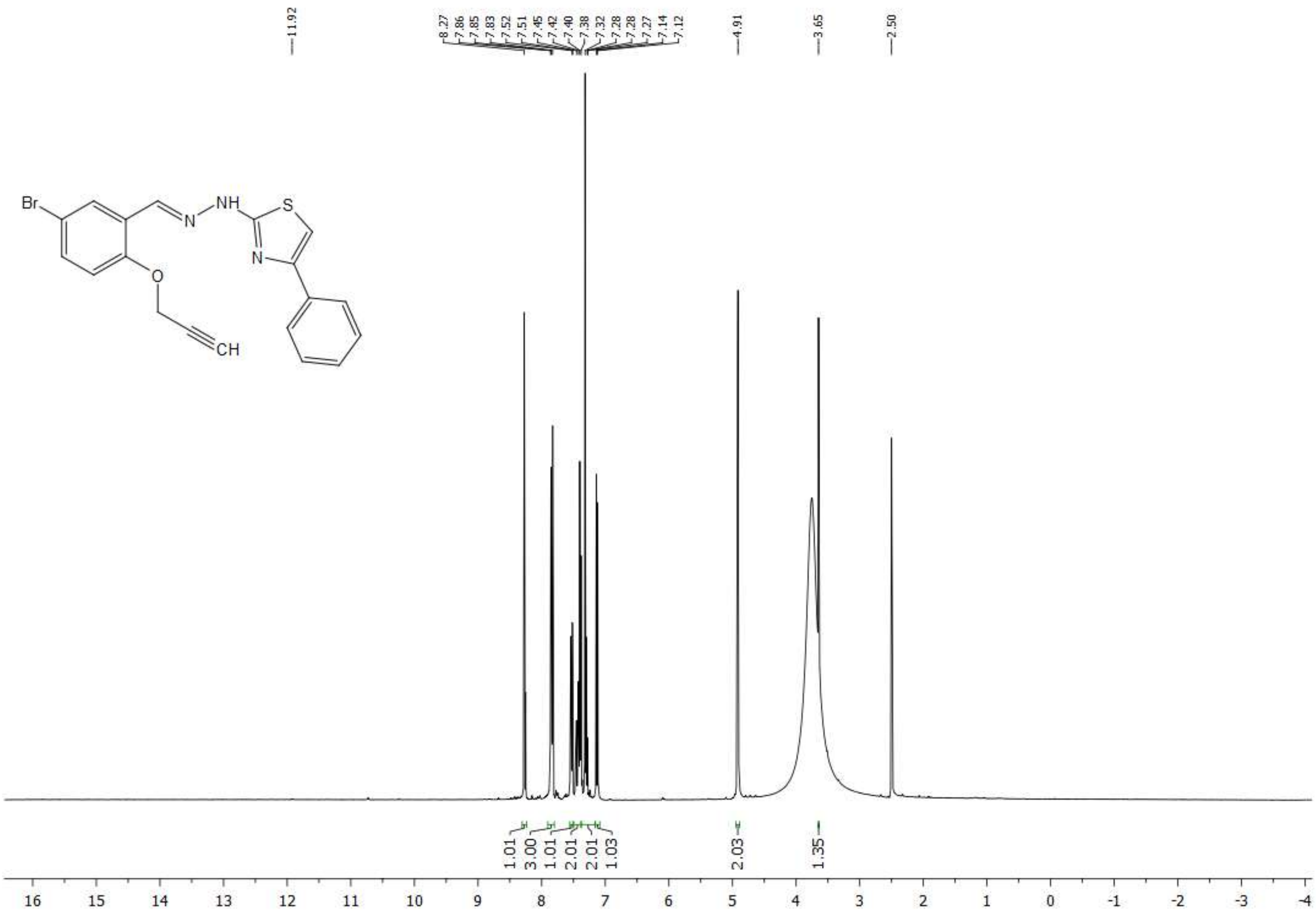

^{13}C NMR spectrum of (*E*)-2-(2-(5-Bromo-2-(prop-2-yn-1-yloxy)benzylidene)hydrazinyl)-4-phenylthiazole (25)

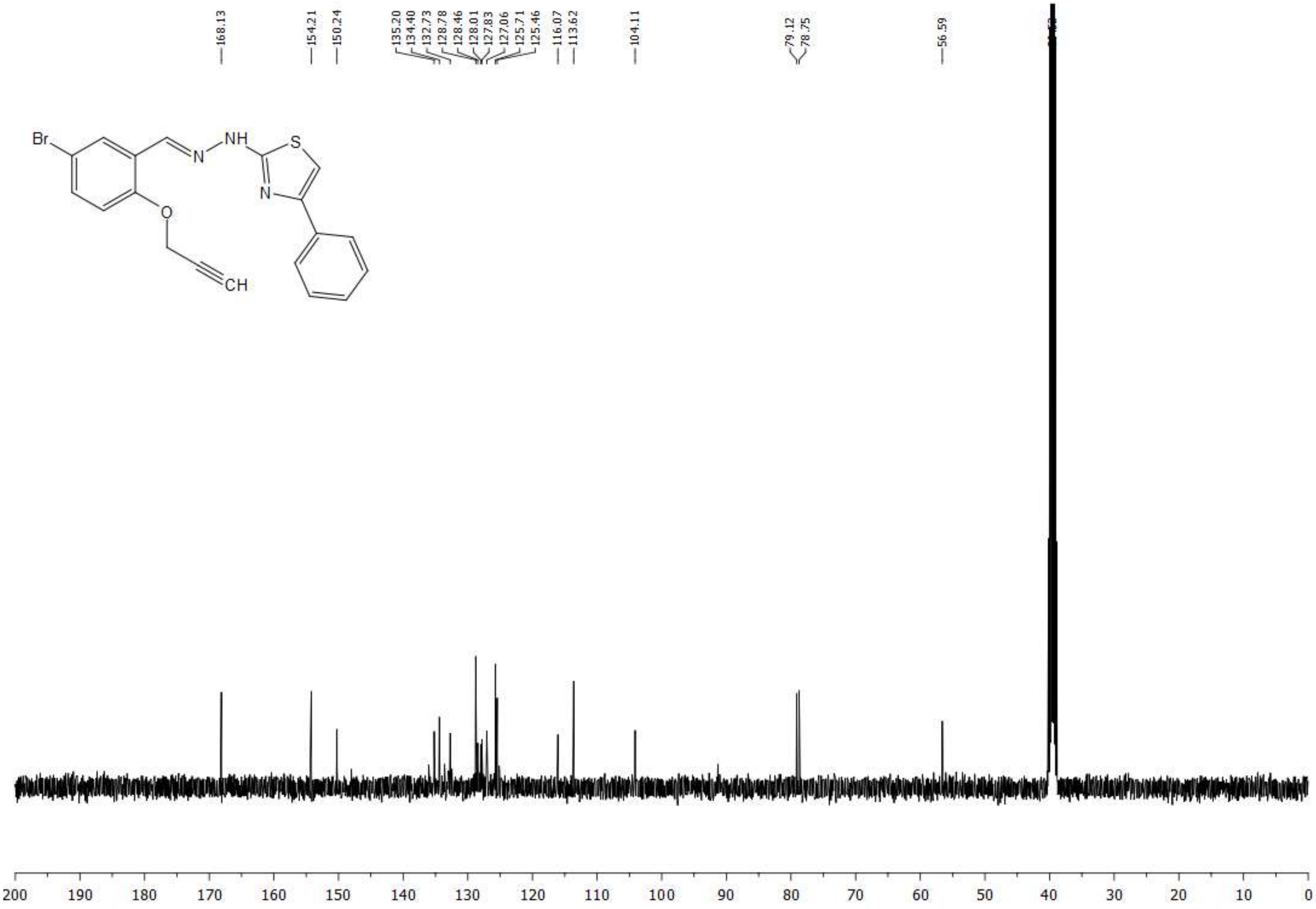

FT-IR spectrum of (*E*)-2-(2-(5-Bromo-2-(prop-2-yn-1-yloxy)benzylidene)hydrazinyl)-4-phenylthiazole (25)

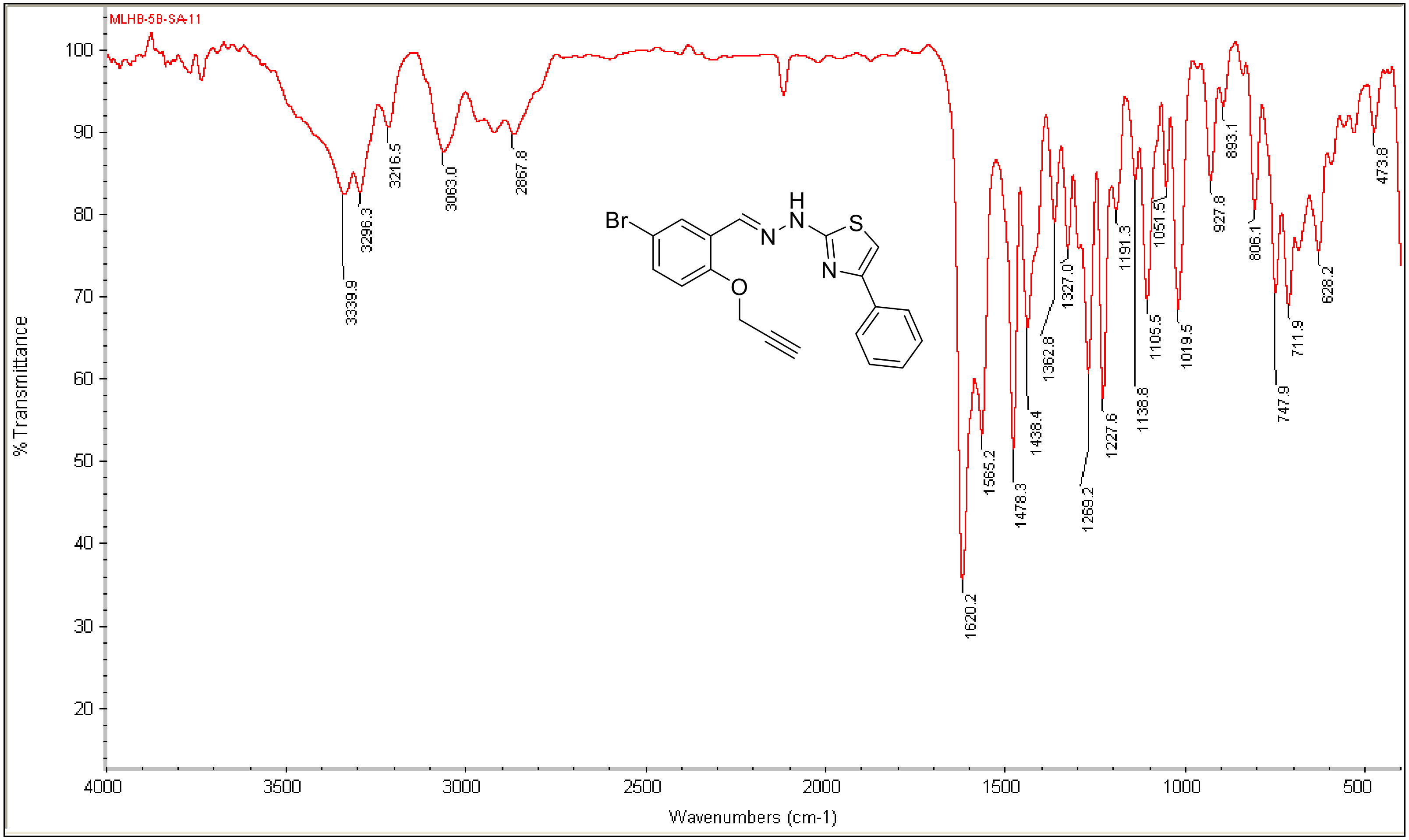

Mass spectrum of (*E*)-2-(2-(5-Bromo-2-(prop-2-yn-1-yloxy)benzylidene)hydrazinyl)-4-(4-fluorophenyl)thiazole (26)

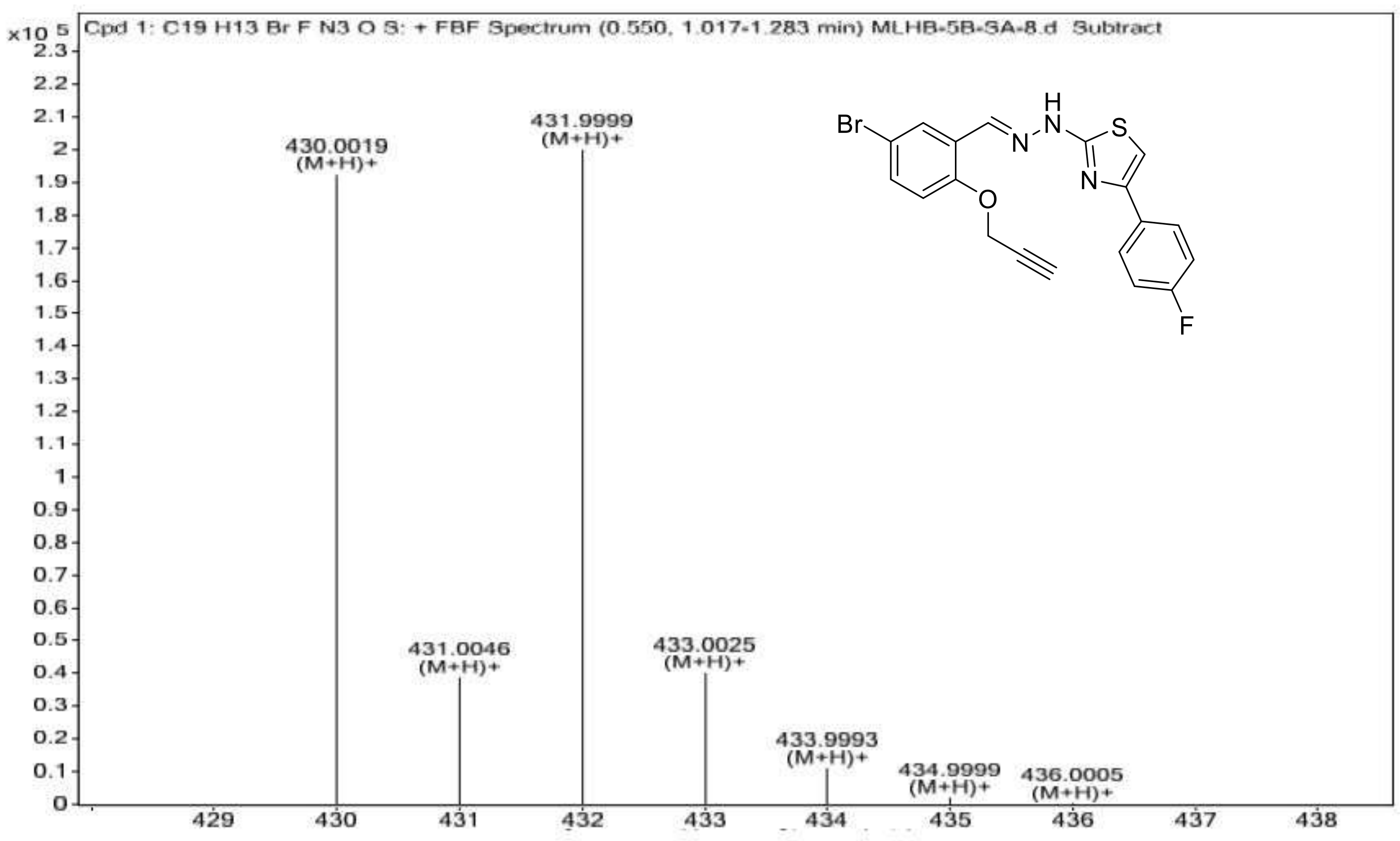

^{1}H NMR spectrum of (*E*)-2-(2-(5-Bromo-2-(prop-2-yn-1-yloxy)benzylidene)hydrazinyl)-4-(4-fluorophenyl)thiazole (26)

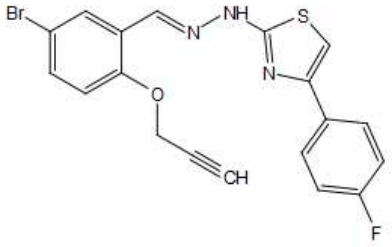

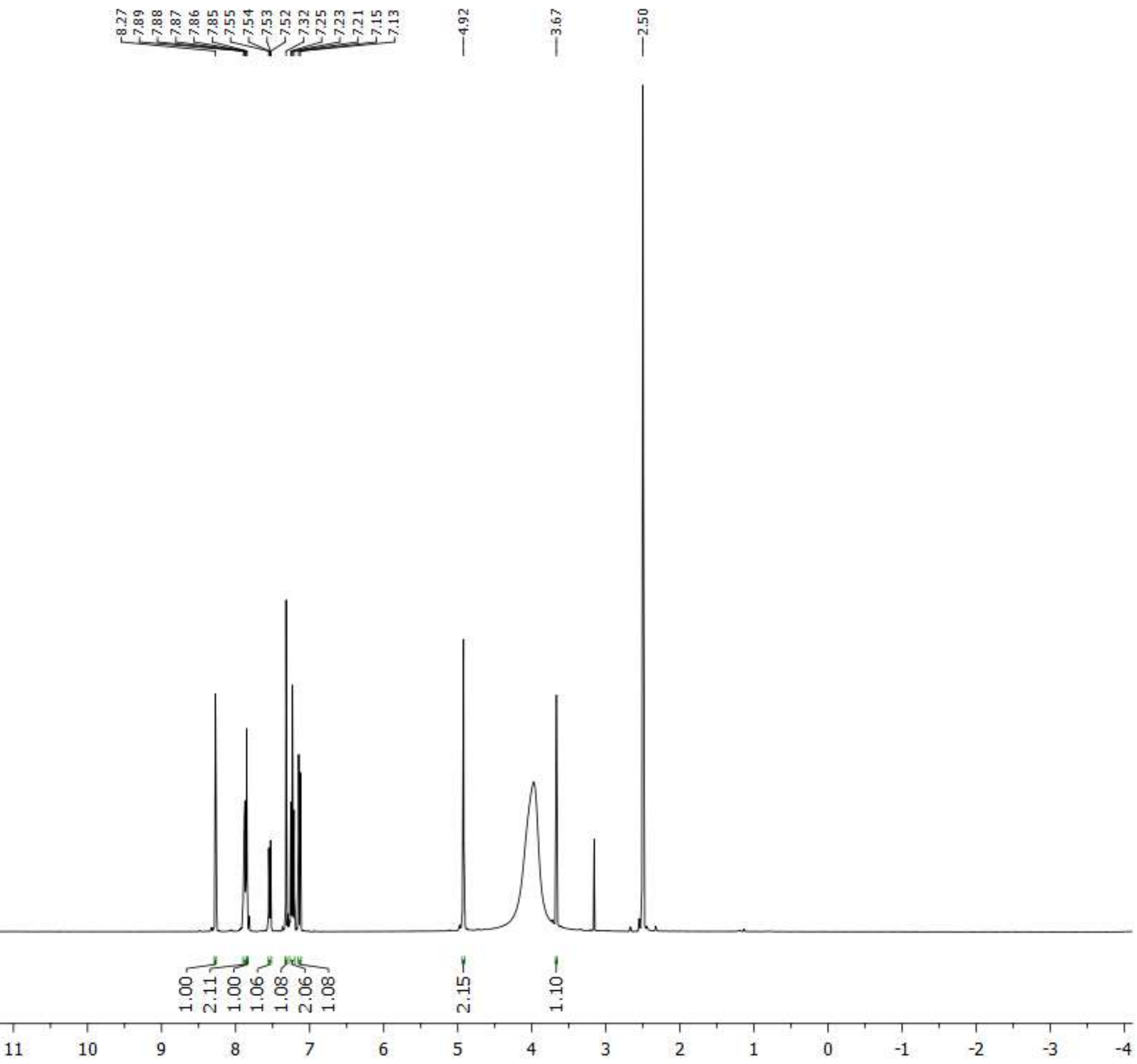

^{13}C NMR spectrum of (*E*)-2-(2-(5-Bromo-2-(prop-2-yn-1-yloxy)benzylidene)hydrazinyl)-4-(4-fluorophenyl)thiazole (26)

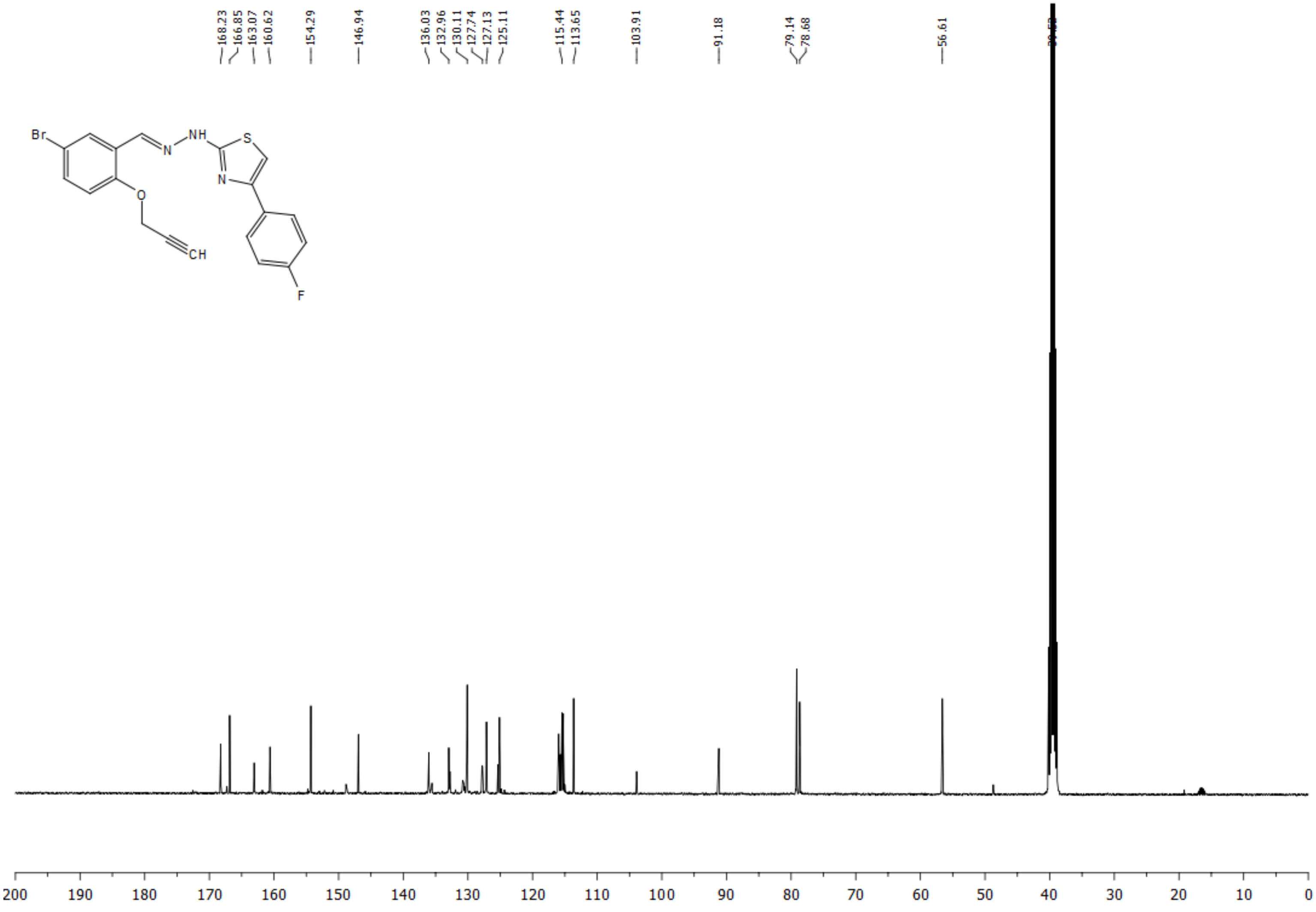

FT-IR spectrum of (*E*)-2-(2-(5-Bromo-2-(prop-2-yn-1-yloxy)benzylidene)hydrazinyl)-4-(4-fluorophenyl)thiazole (26)

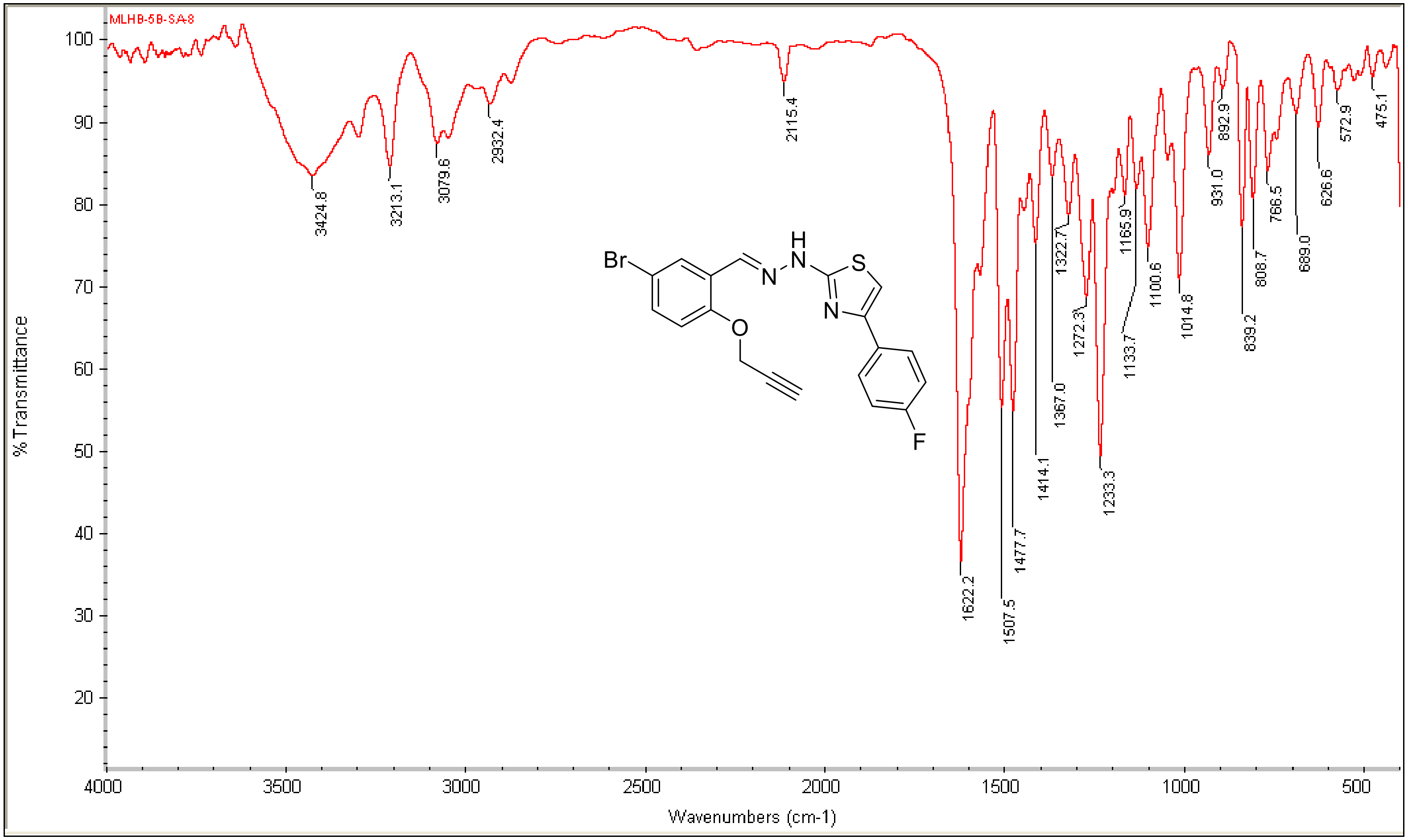

Mass spectrum of (*E*)-2-(2-(5-Bromo-2-(prop-2-yn-1-yloxy)benzylidene)hydrazinyl)-4-(4-chlorophenyl)thiazole (27)

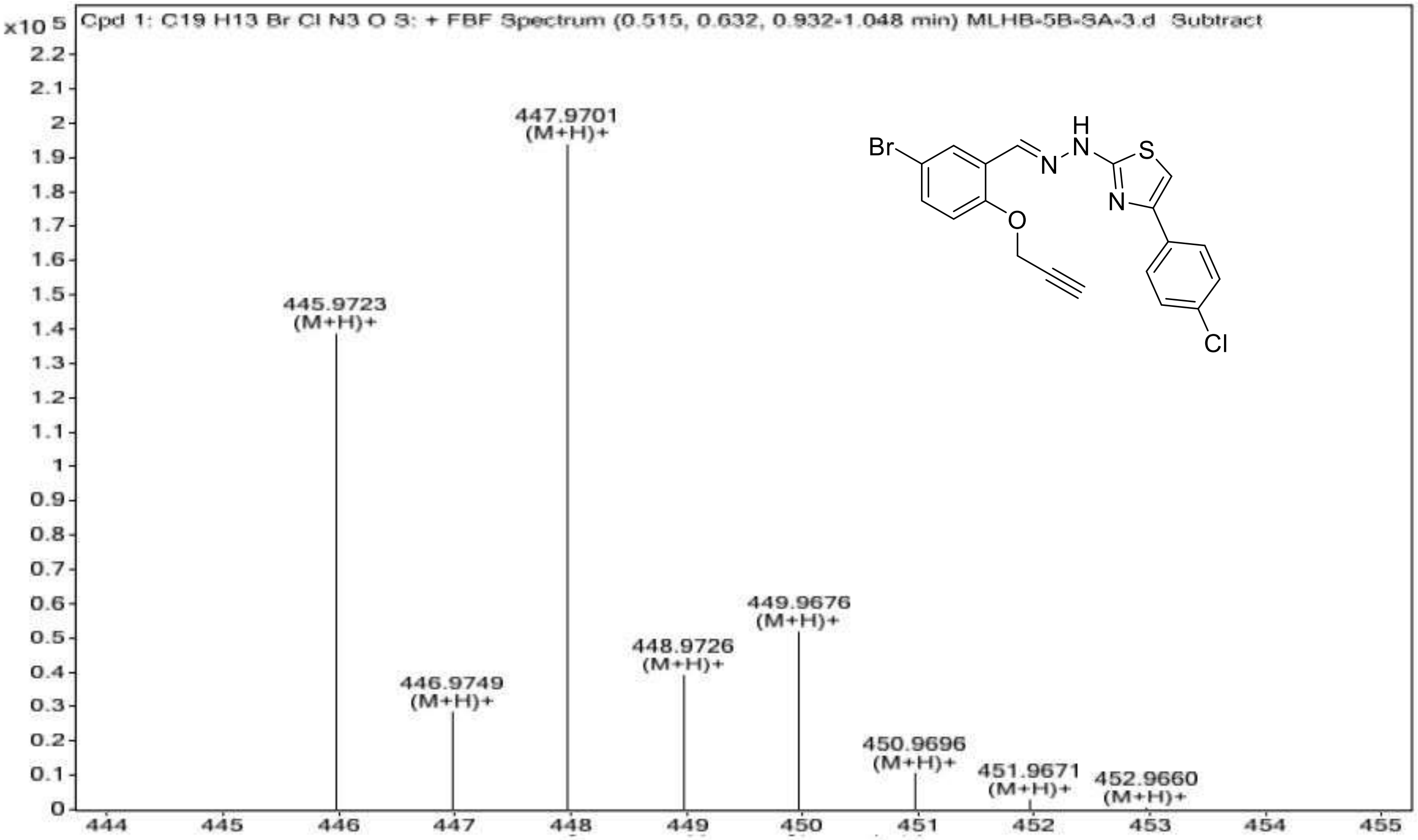

^{1}H NMR spectrum of (*E*)-2-(2-(5-Bromo-2-(prop-2-yn-1-yloxy)benzylidene)hydrazinyl)-4-(4-chlorophenyl)thiazole (27)

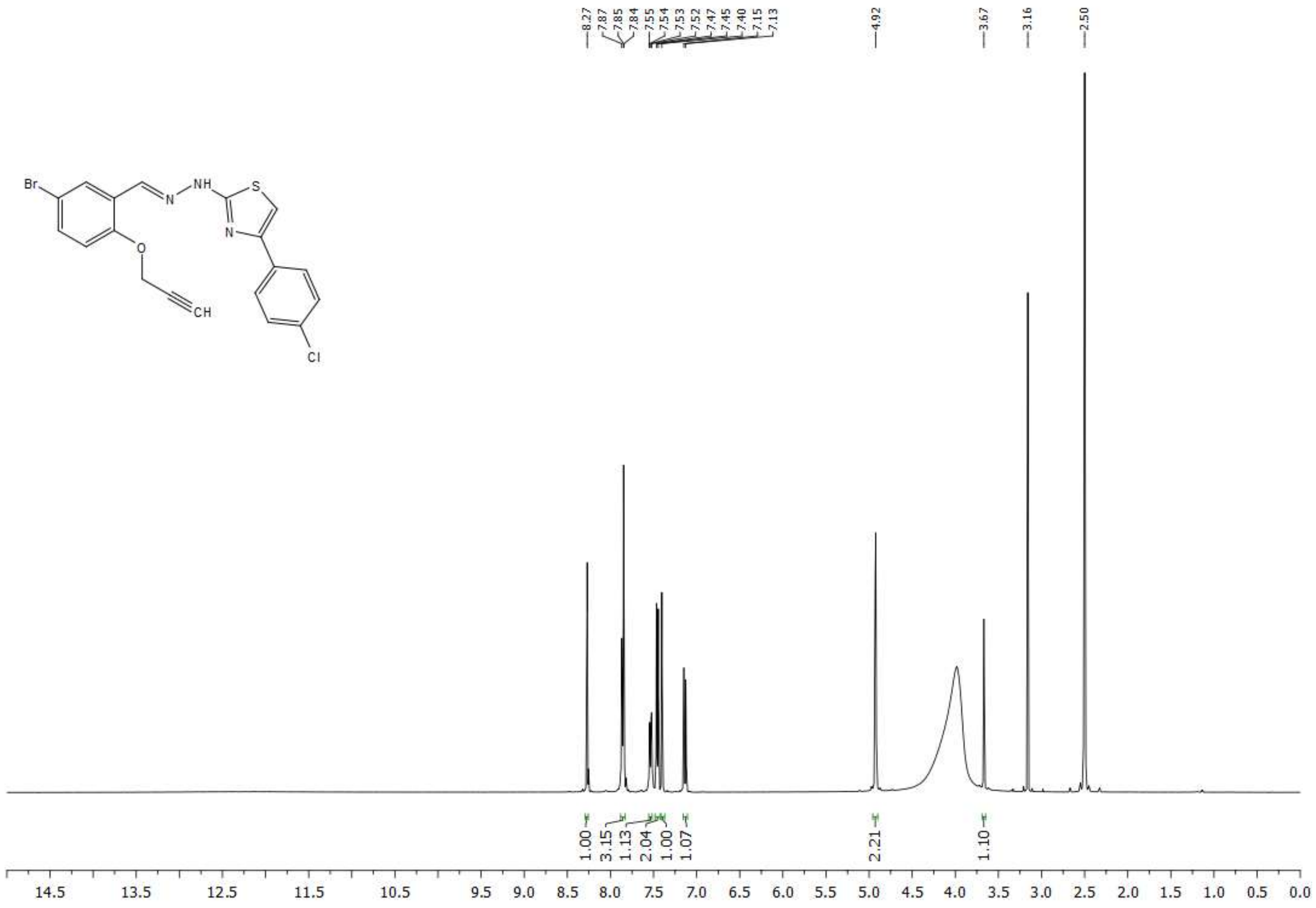

^{13}C NMR spectrum of (*E*)-2-(2-(5-Bromo-2-(prop-2-yn-1-yloxy)benzylidene)hydrazinyl)-4-(4-chlorophenyl)thiazole (27)

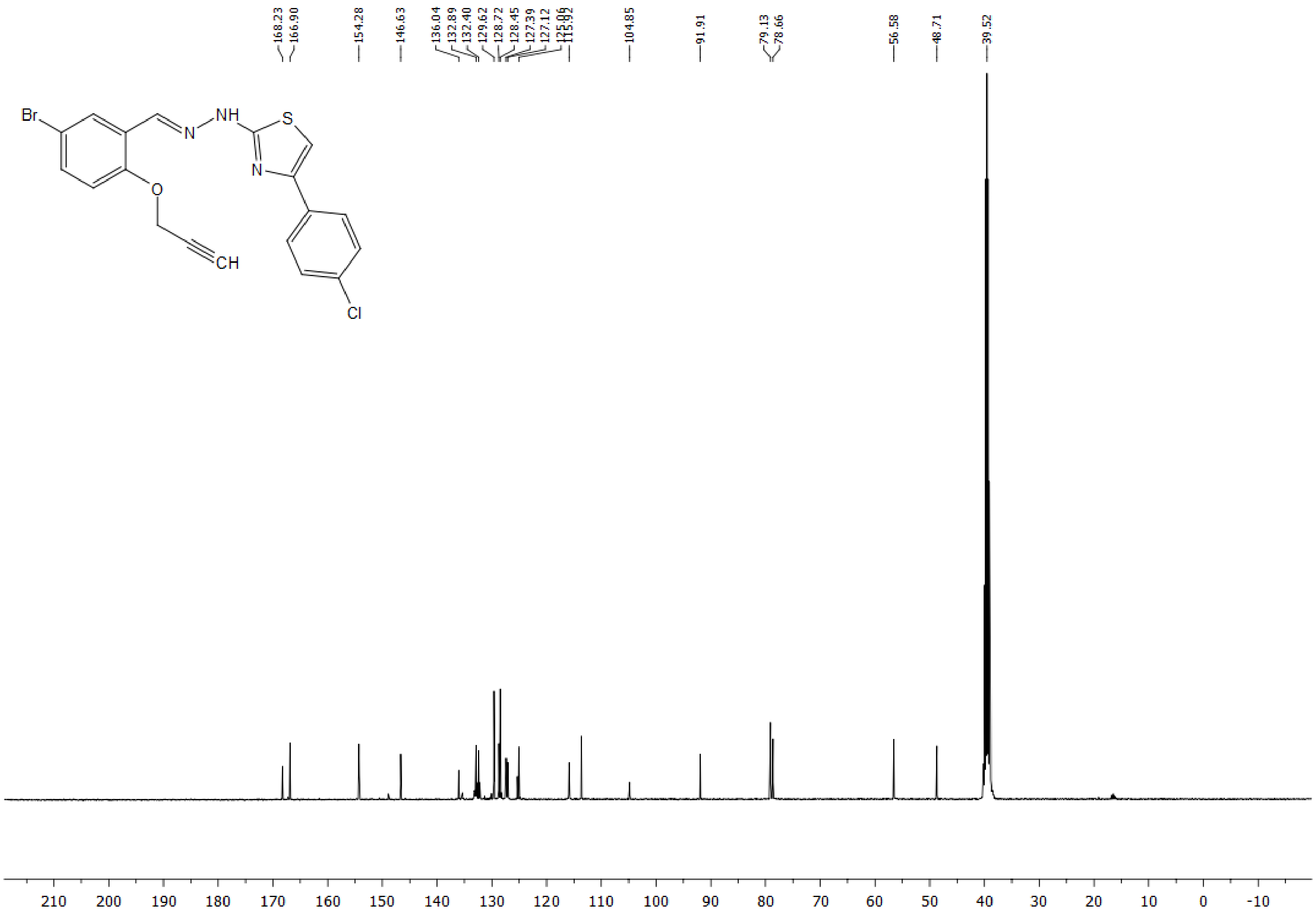

FT-IR spectrum of (*E*)-2-(2-(5-Bromo-2-(prop-2-yn-1-yloxy)benzylidene)hydrazinyl)-4-(4-chlorophenyl)thiazole (27)

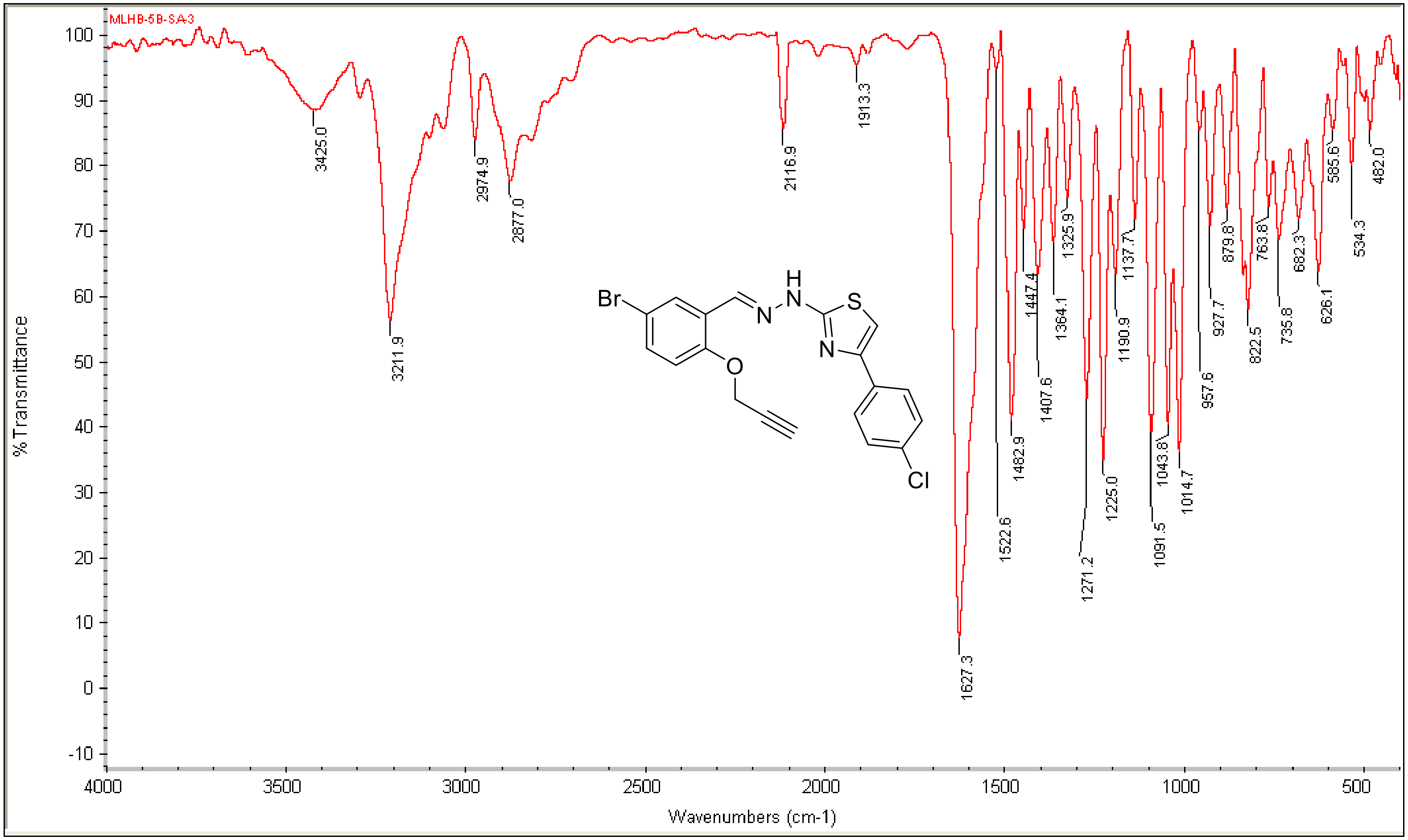

Mass spectrum of (*E*)-2-(2-(5-Bromo-2-(prop-2-yn-1-yloxy)benzylidene)hydrazinyl)-4-(3,4-dichlorophenyl)thiazole (28)

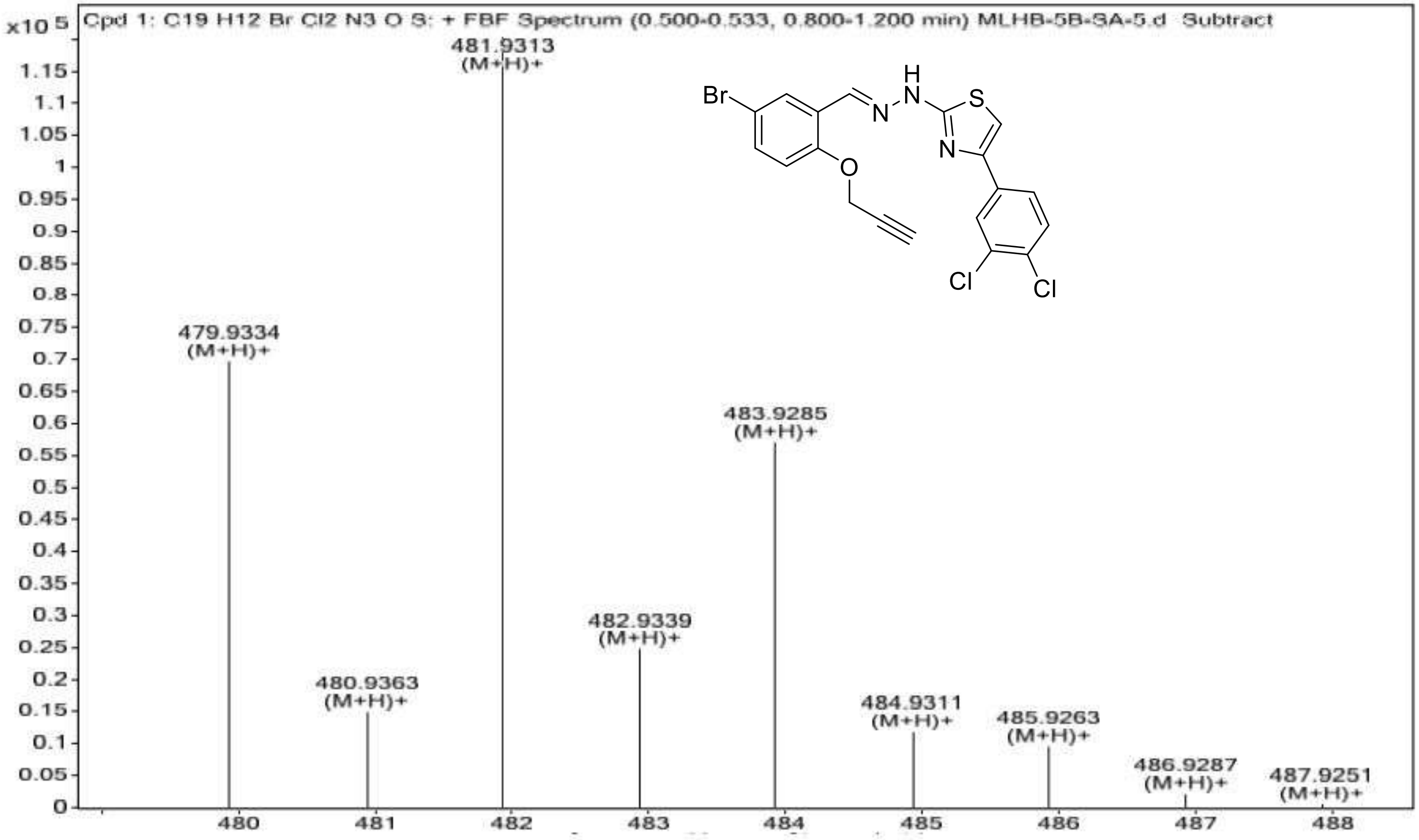

^{1}H NMR spectrum of (*E*)-2-(2-(5-Bromo-2-(prop-2-yn-1-yloxy)benzylidene)hydrazinyl)-4-(3,4-dichlorophenyl)thiazole (28)

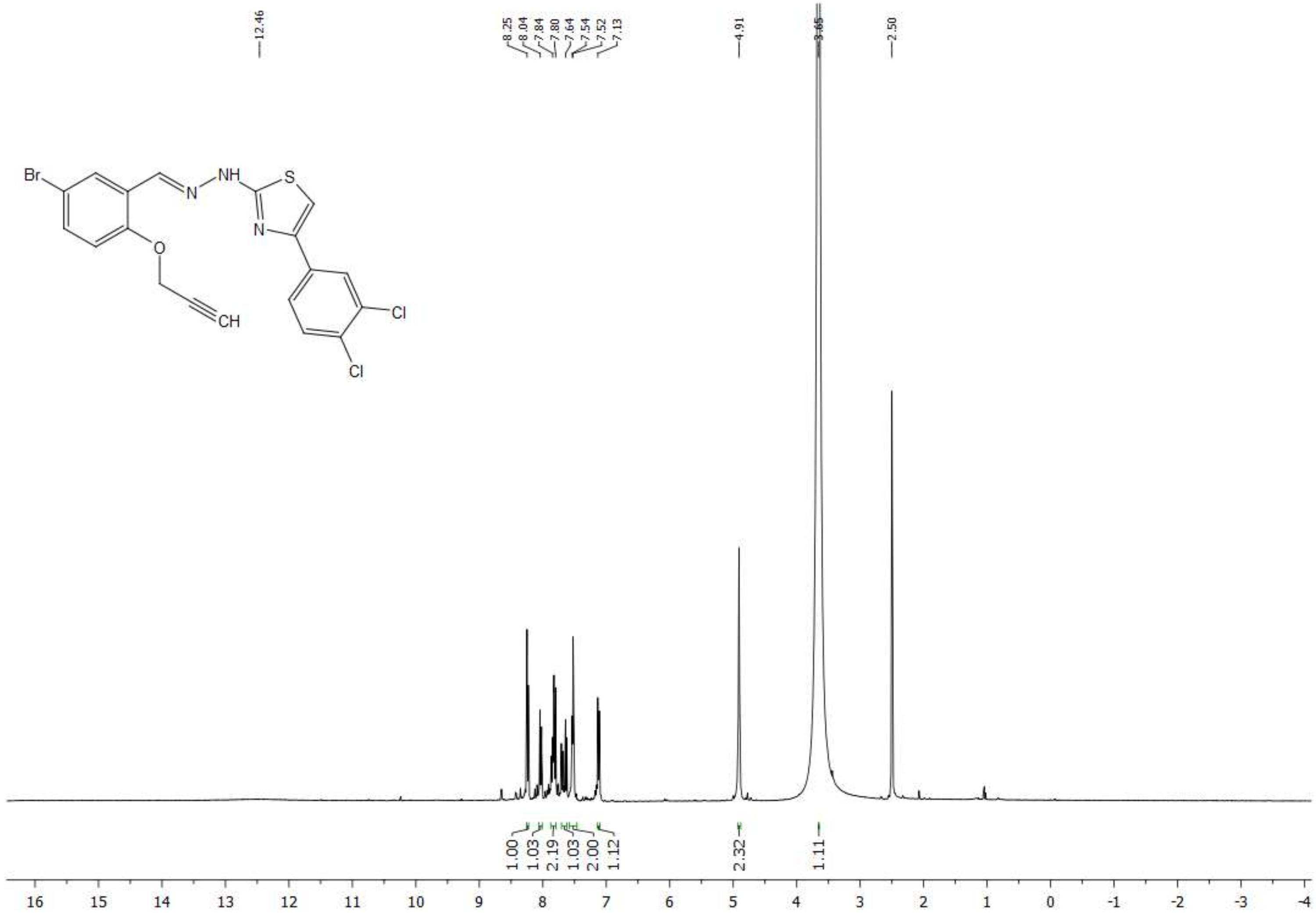

^{13}C NMR spectrum of (*E*)-2-(2-(5-Bromo-2-(prop-2-yn-1-yloxy)benzylidene)hydrazinyl)-4-(3,4-dichlorophenyl)thiazole (28)

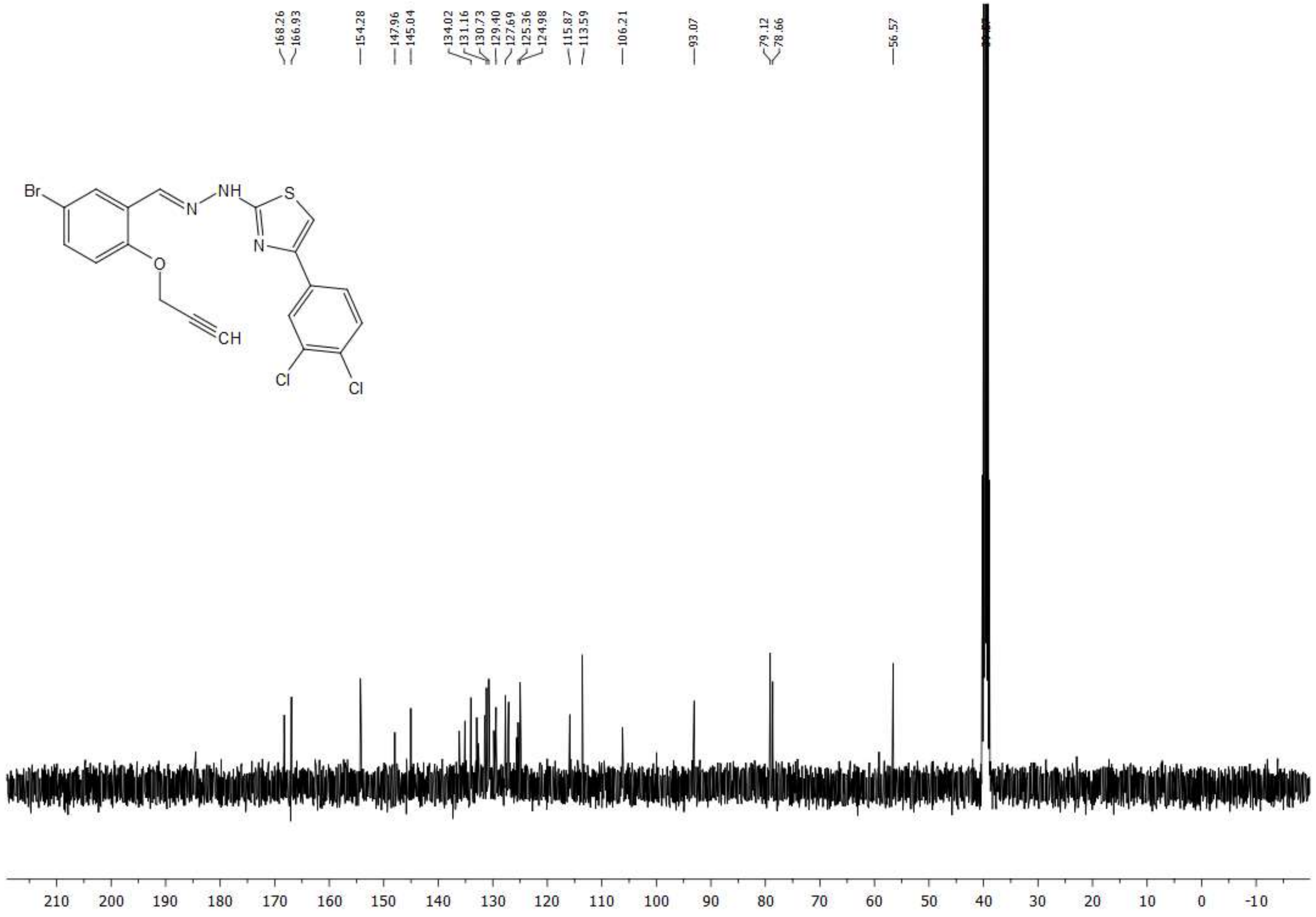

FT-IR spectrum of (*E*)-2-(2-(5-Bromo-2-(prop-2-yn-1-yloxy)benzylidene)hydrazinyl)-4-(3,4-dichlorophenyl)thiazole (28)

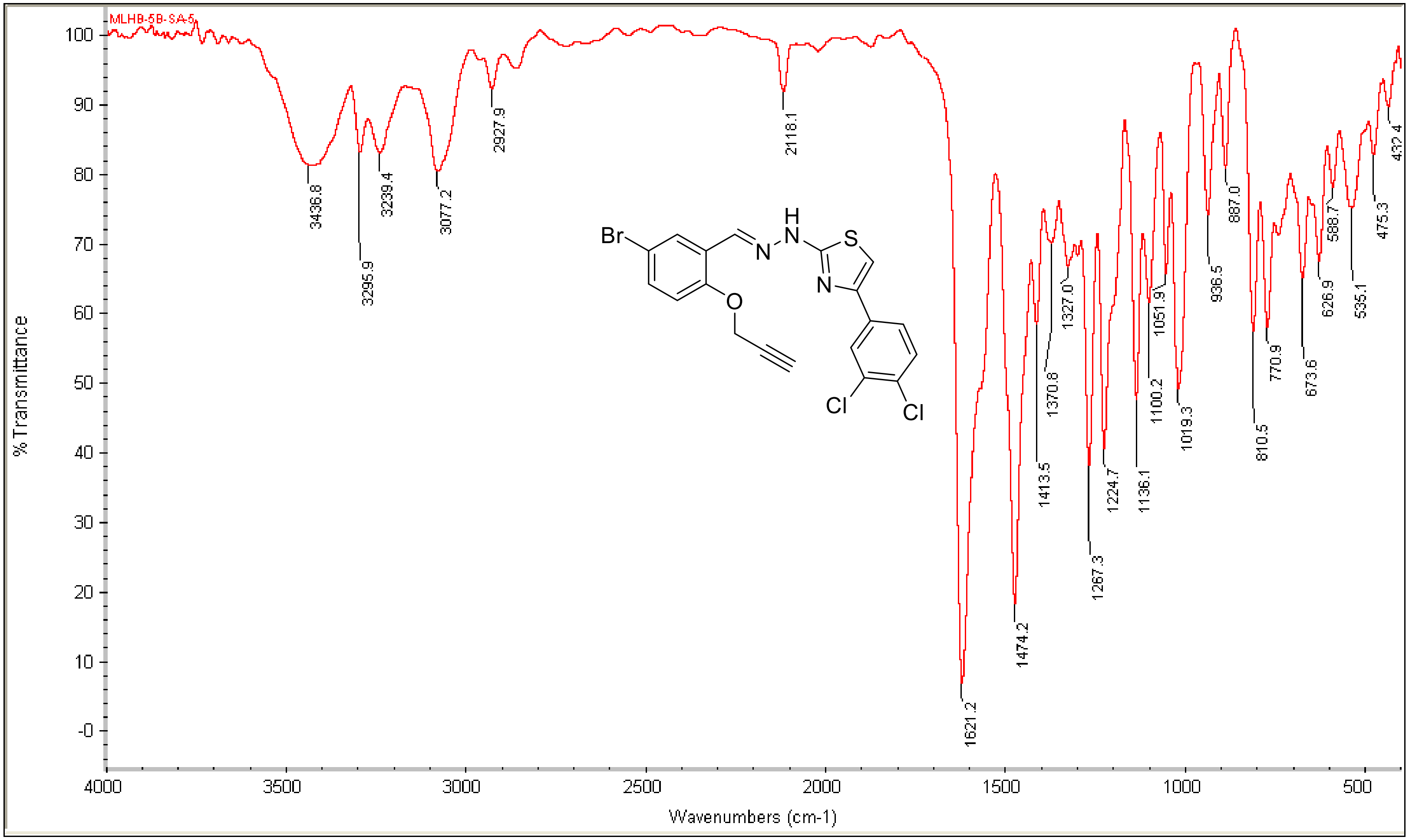

Mass spectrum of (*E*)-2-(2-(5-Bromo-2-(prop-2-yn-1-yloxy)benzylidene)hydrazinyl)-4-(4-bromophenyl)thiazole (29)

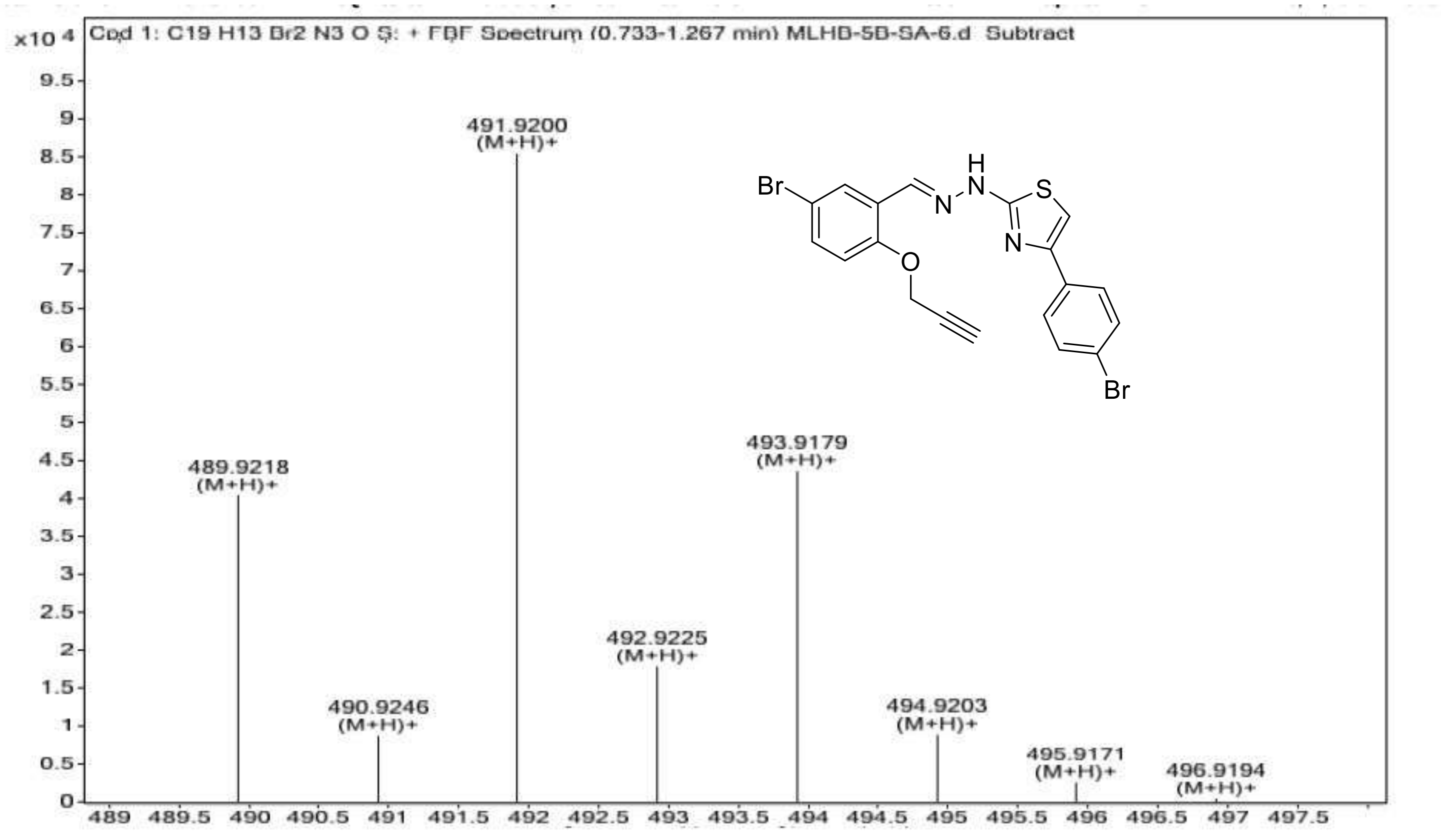

^{1}H NMR spectrum of (*E*)-2-(2-(5-Bromo-2-(prop-2-yn-1-yloxy)benzylidene)hydrazinyl)-4-(4-bromophenyl)thiazole (29)

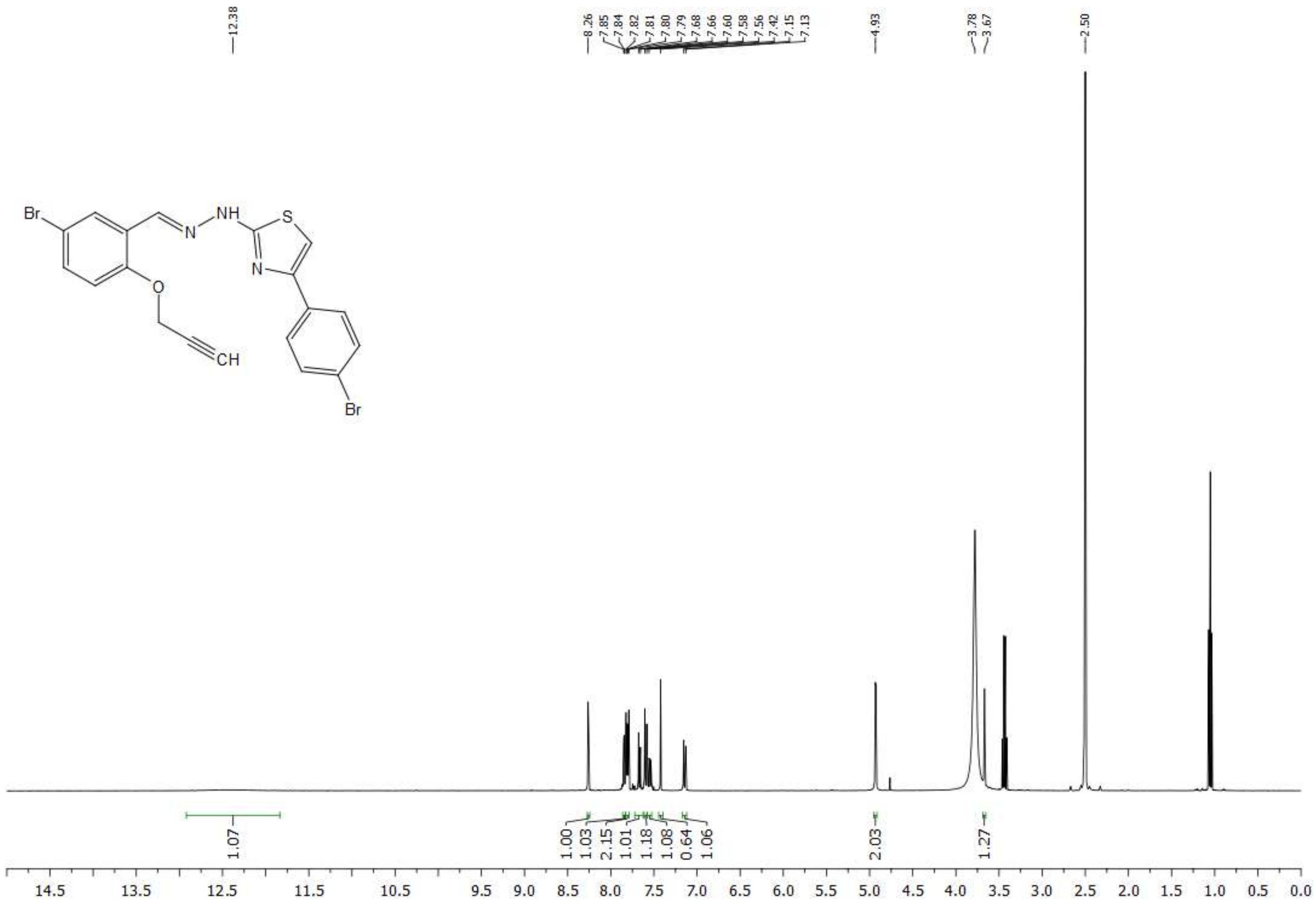

^{13}C NMR spectrum of (*E*)-2-(2-(5-Bromo-2-(prop-2-yn-1-yloxy)benzylidene)hydrazinyl)-4-(4-bromophenyl)thiazole (29)

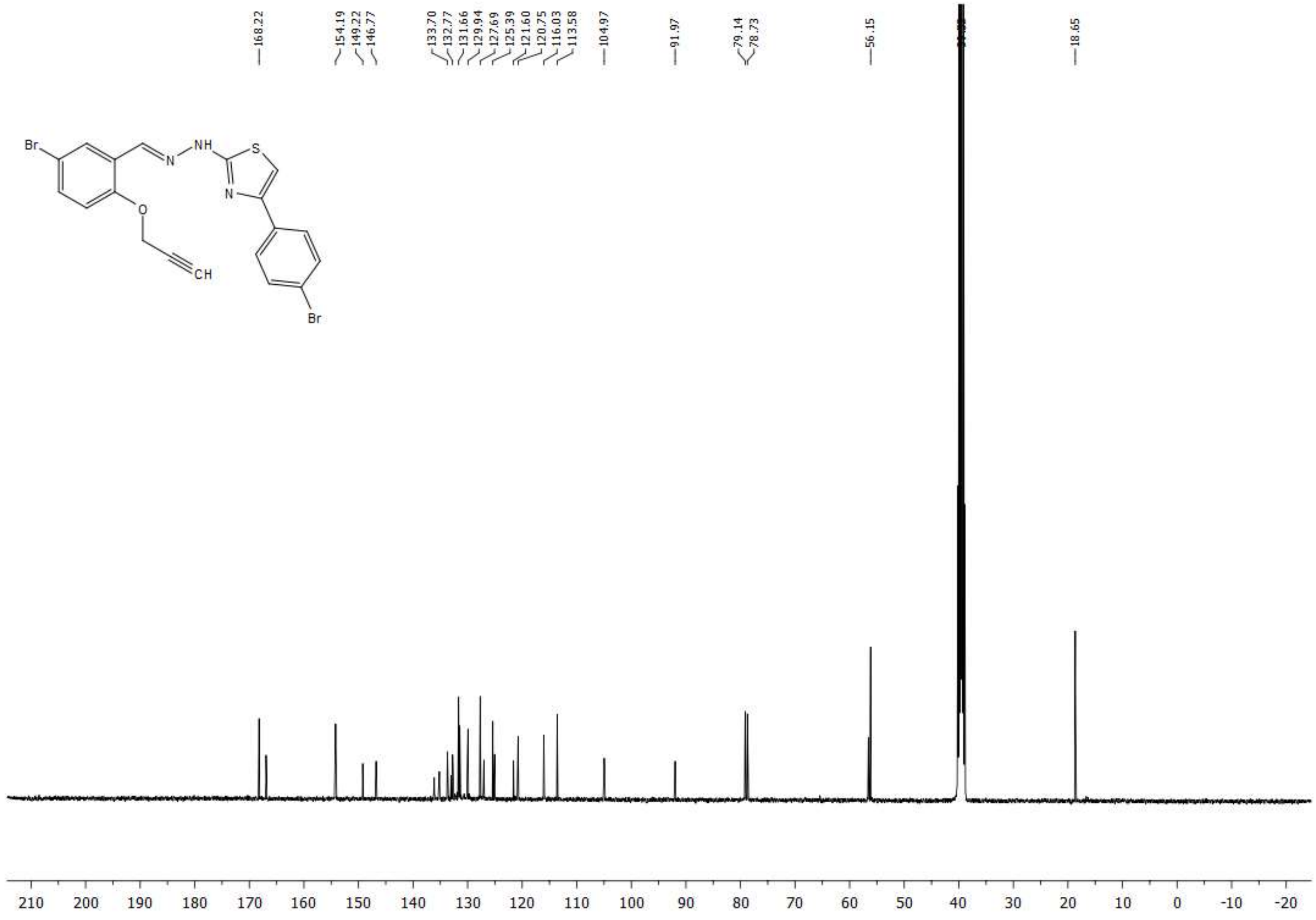

FT-IR spectrum of (*E*)-2-(2-(5-Bromo-2-(prop-2-yn-1-yloxy)benzylidene)hydrazinyl)-4-(4-bromophenyl)thiazole (29)

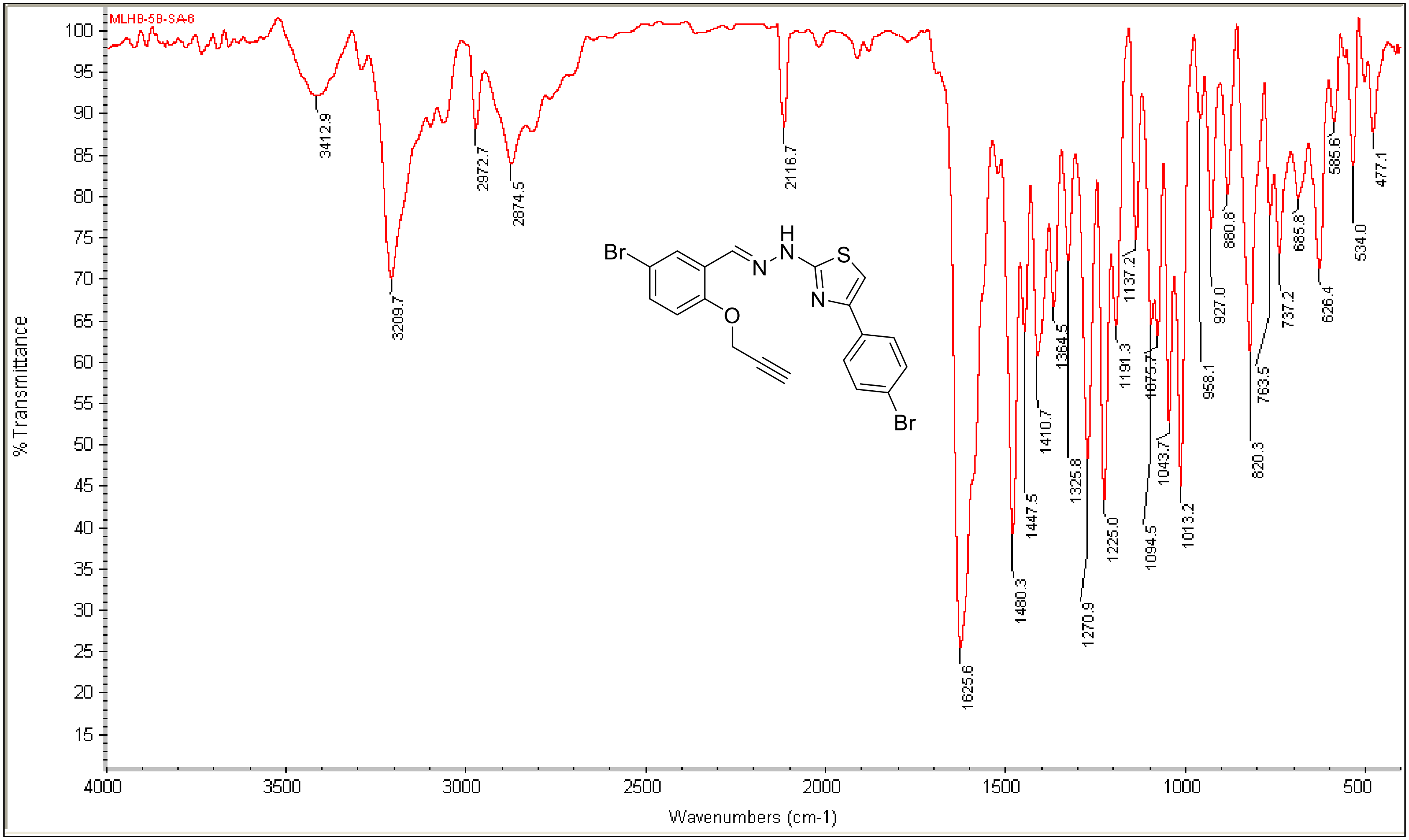

Mass spectrum of (*E*)-4-(2-(2-(5-Bromo-2-(prop-2-yn-1-yloxy)benzylidene)hydrazinyl)thiazol-4-yl)benzonitrile (30)

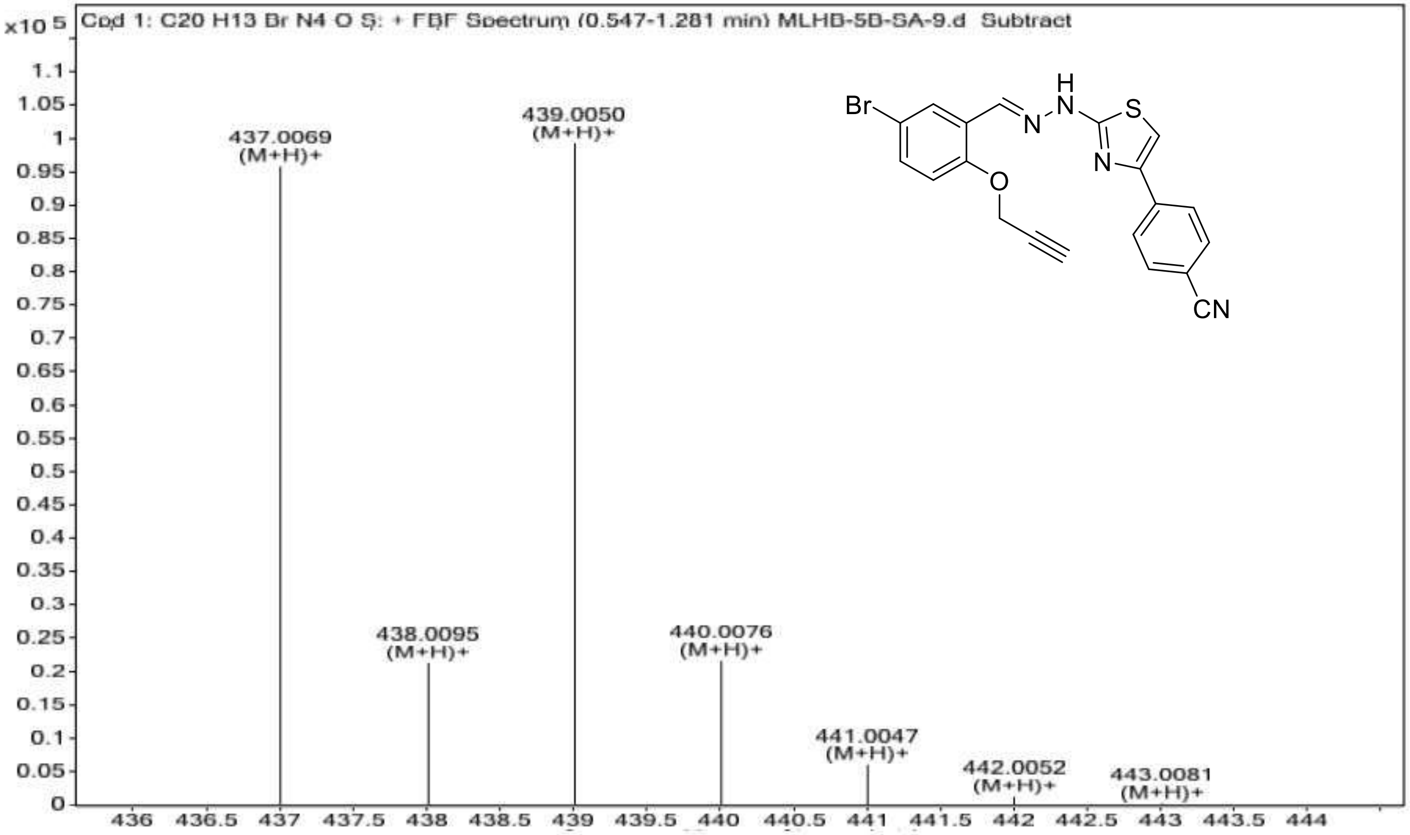

^{1}H NMR spectrum of (*E*)-4-(2-(2-(5-Bromo-2-(prop-2-yn-1-yloxy)benzylidene)hydrazinyl)thiazol-4-yl)benzonitrile (30)

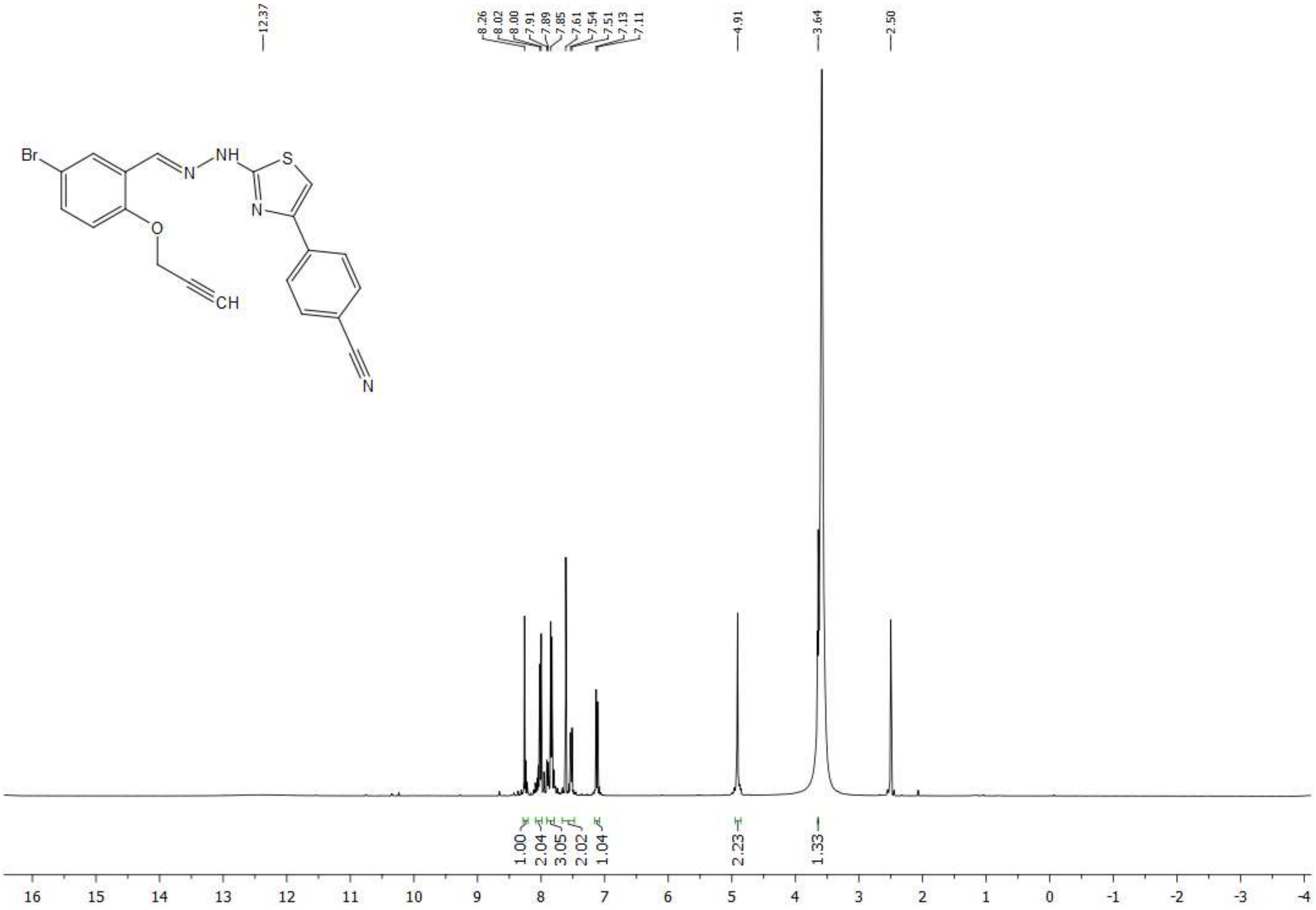

^{13}C NMR spectrum of (*E*)-4-(2-(2-(5-Bromo-2-(prop-2-yn-1-yloxy)benzylidene)hydrazinyl)thiazol-4-yl)benzonitrile (30)

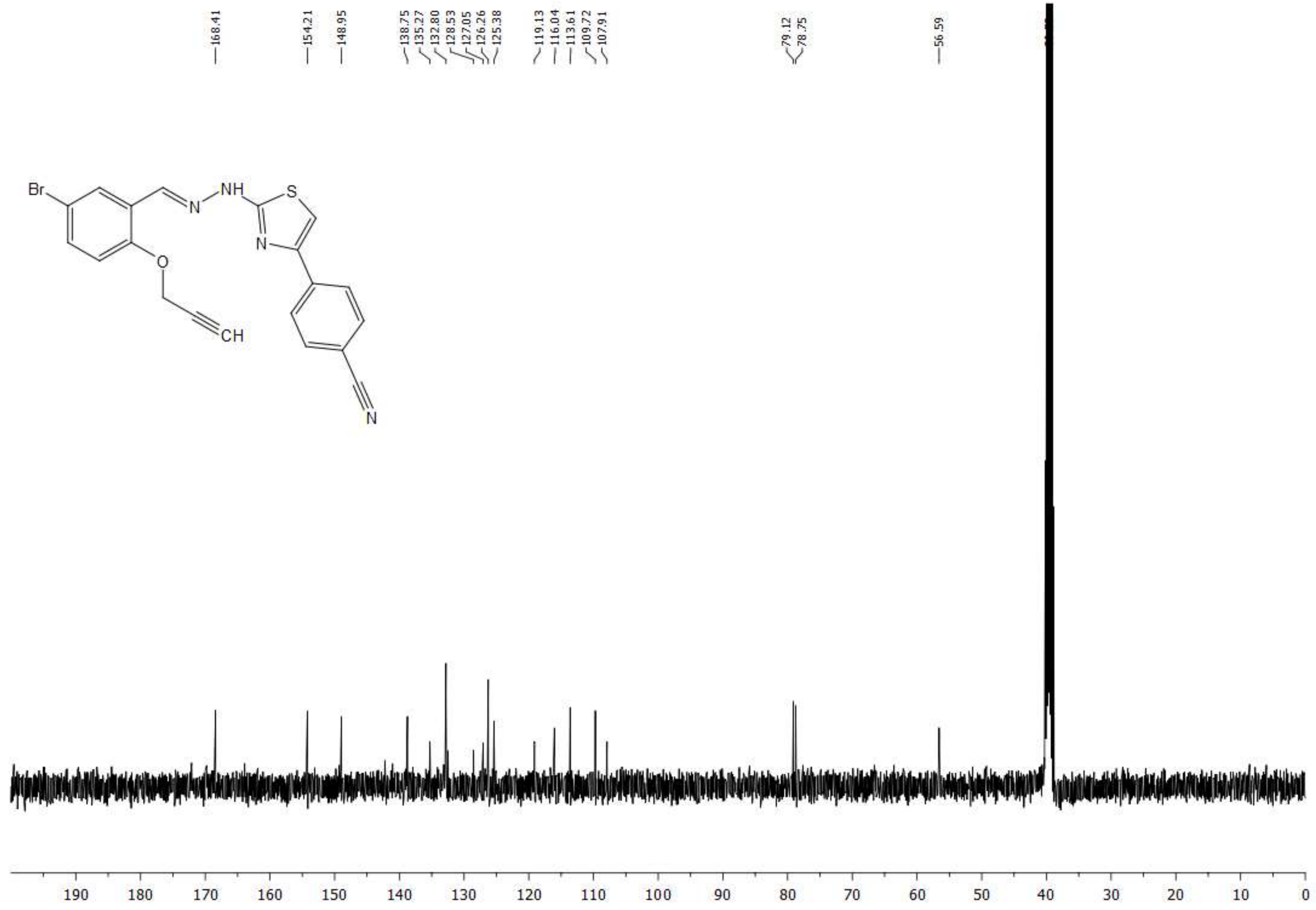

FT-IR spectrum of (*E*)-4-(2-(2-(5-Bromo-2-(prop-2-yn-1-yloxy)benzylidene)hydrazinyl)thiazol-4-yl)benzonitrile (30)

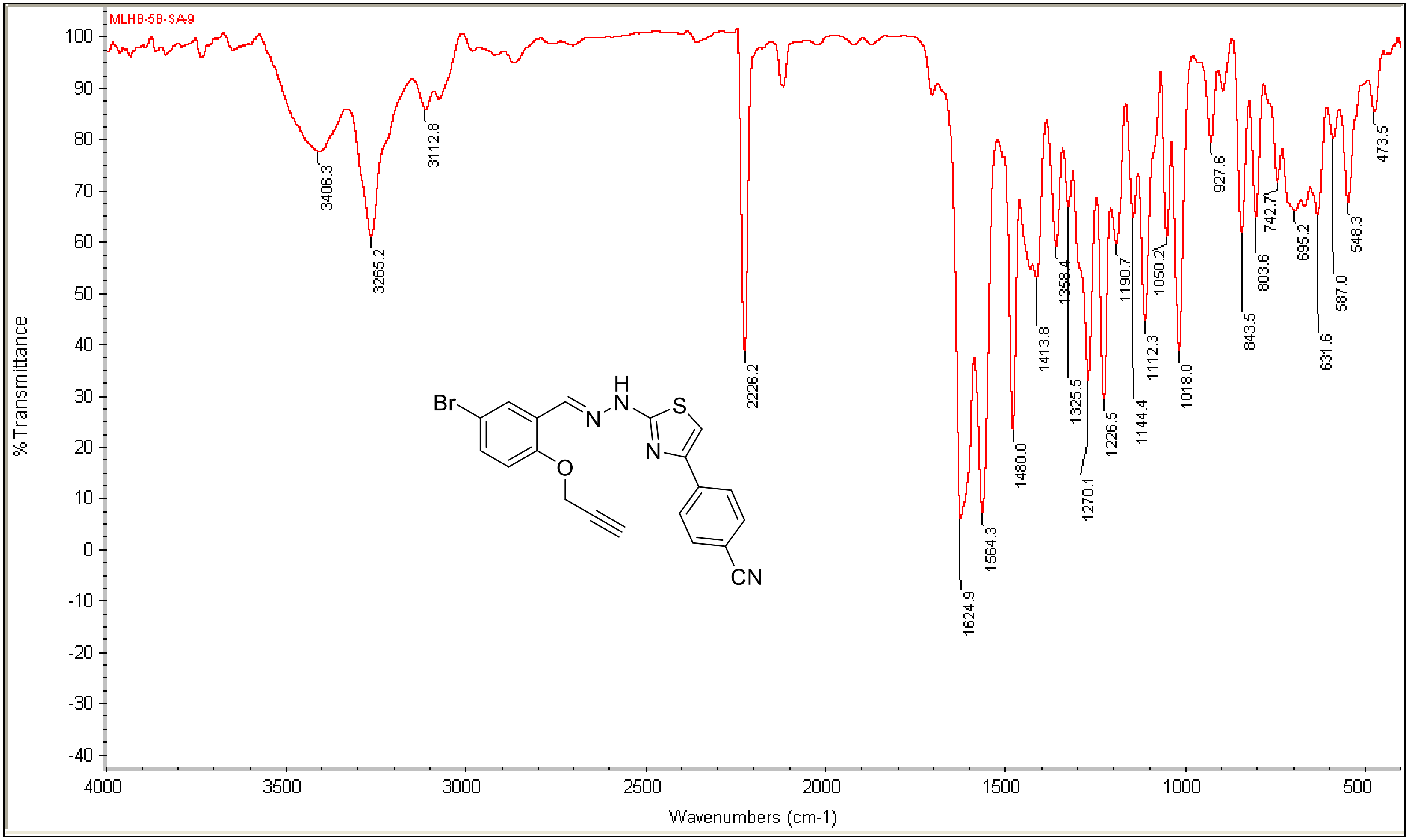

Mass spectrum of (*E*)-2-(2-(5-Bromo-2-(prop-2-yn-1-yloxy)benzylidene)hydrazinyl)-4-(p-tolyl)thiazole (31)

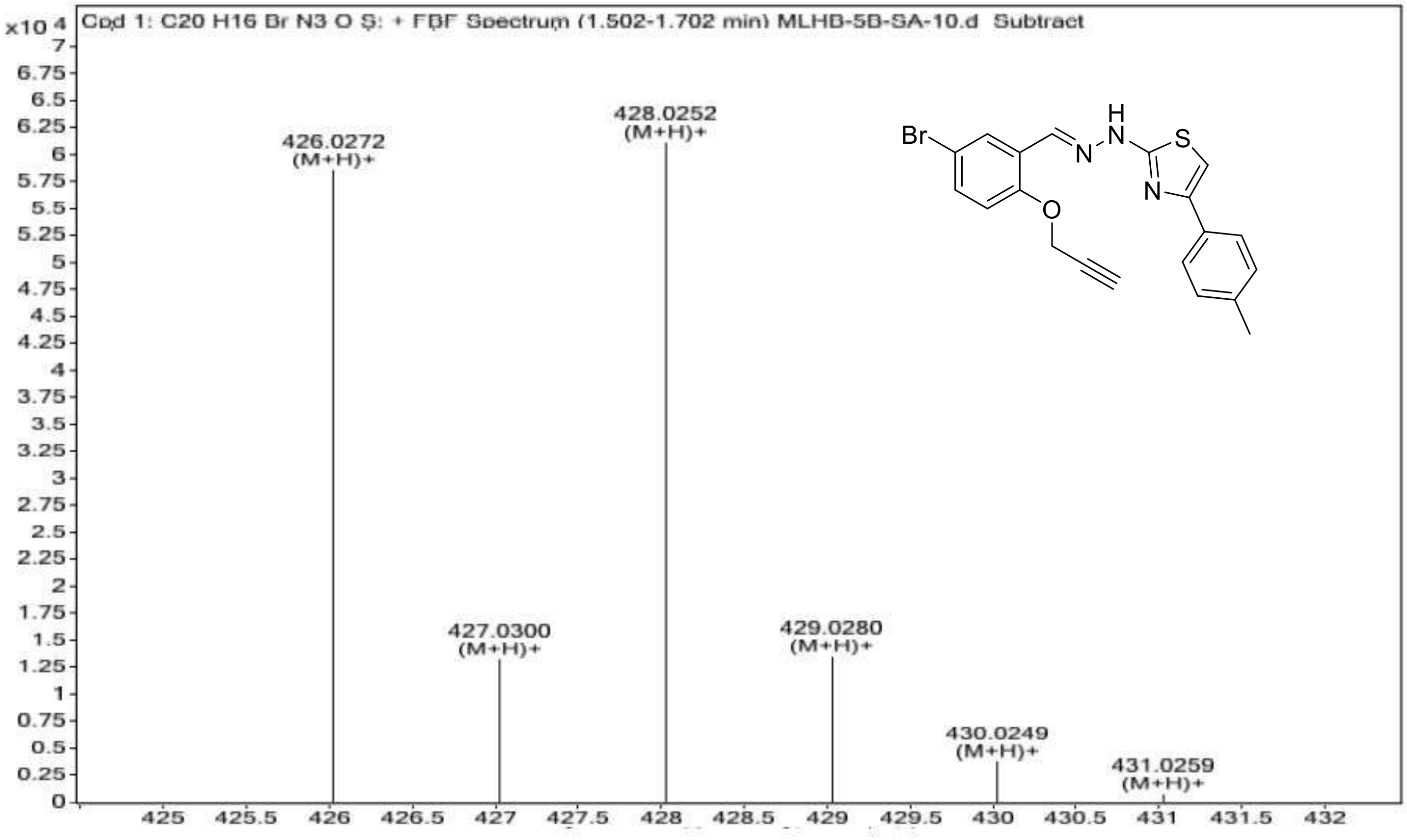

^{1}H NMR spectrum of (*E*)-2-(2-(5-Bromo-2-(prop-2-yn-1-yloxy)benzylidene)hydrazinyl)-4-(p-tolyl)thiazole (31)

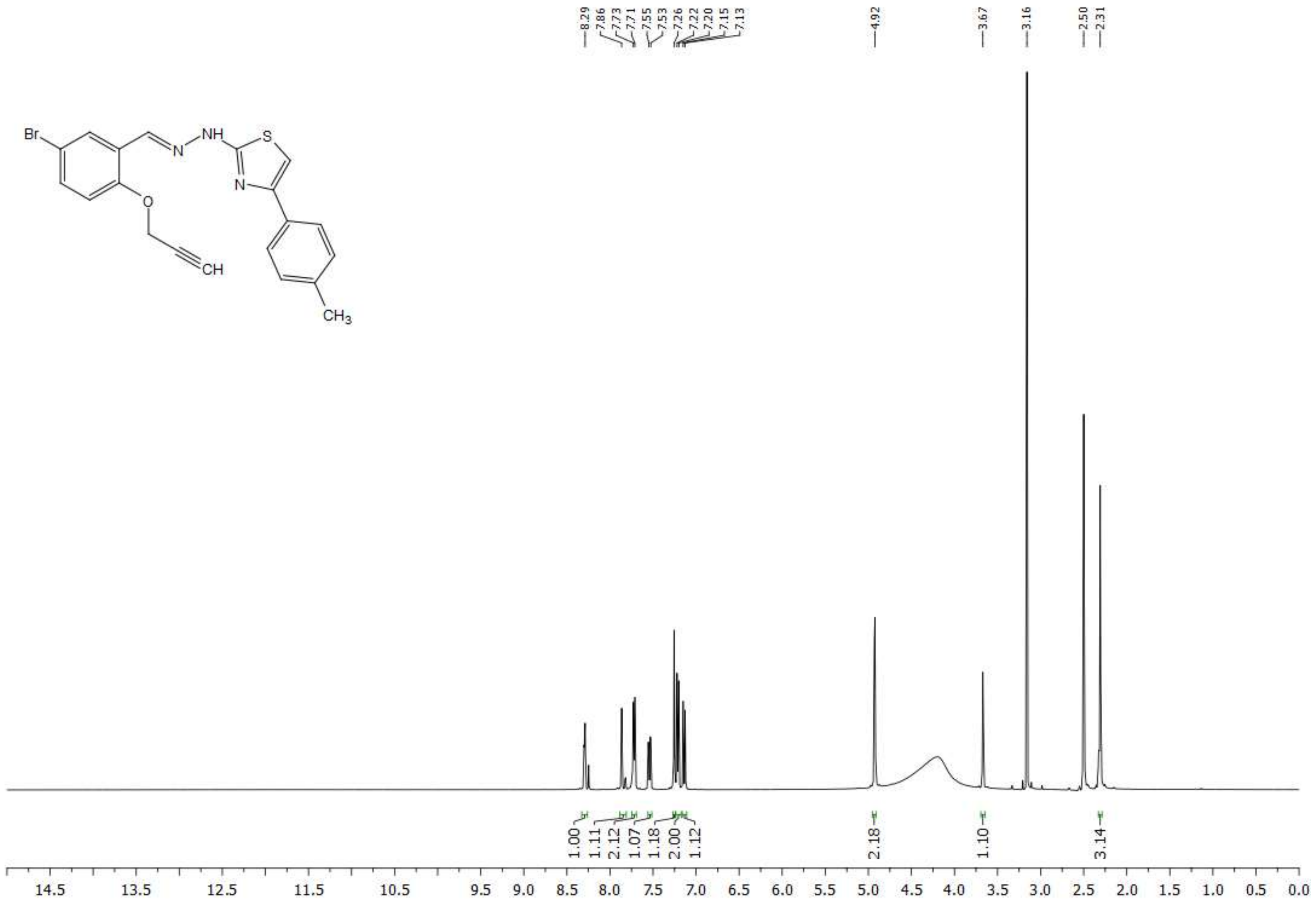

^{13}C NMR spectrum of (*E*)-2-(2-(5-Bromo-2-(prop-2-yn-1-yloxy)benzylidene)hydrazinyl)-4-(p-tolyl)thiazole (31)

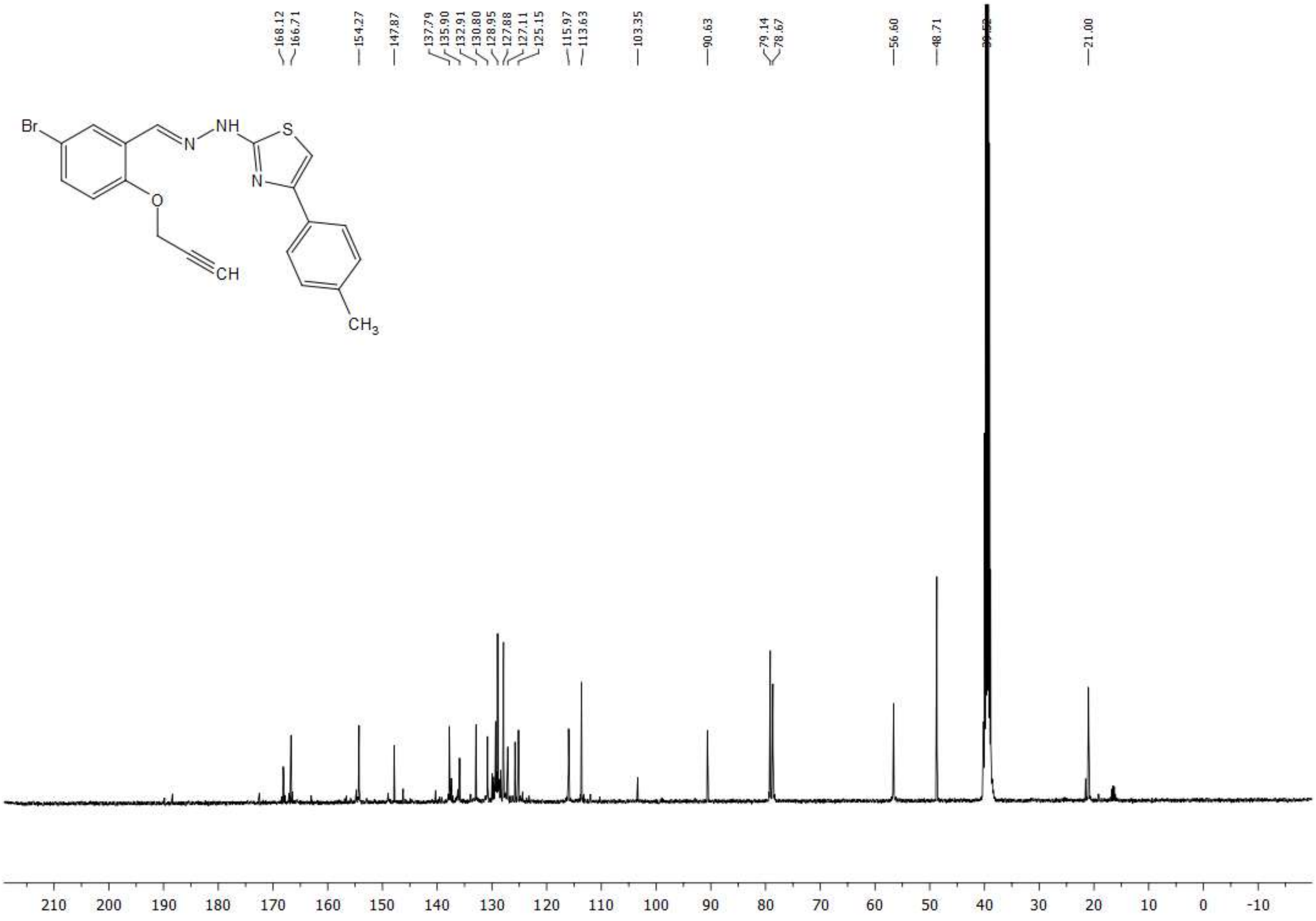

FT-IR spectrum of (*E*)-2-(2-(5-Bromo-2-(prop-2-yn-1-yloxy)benzylidene)hydrazinyl)-4-(p-tolyl)thiazole (31)

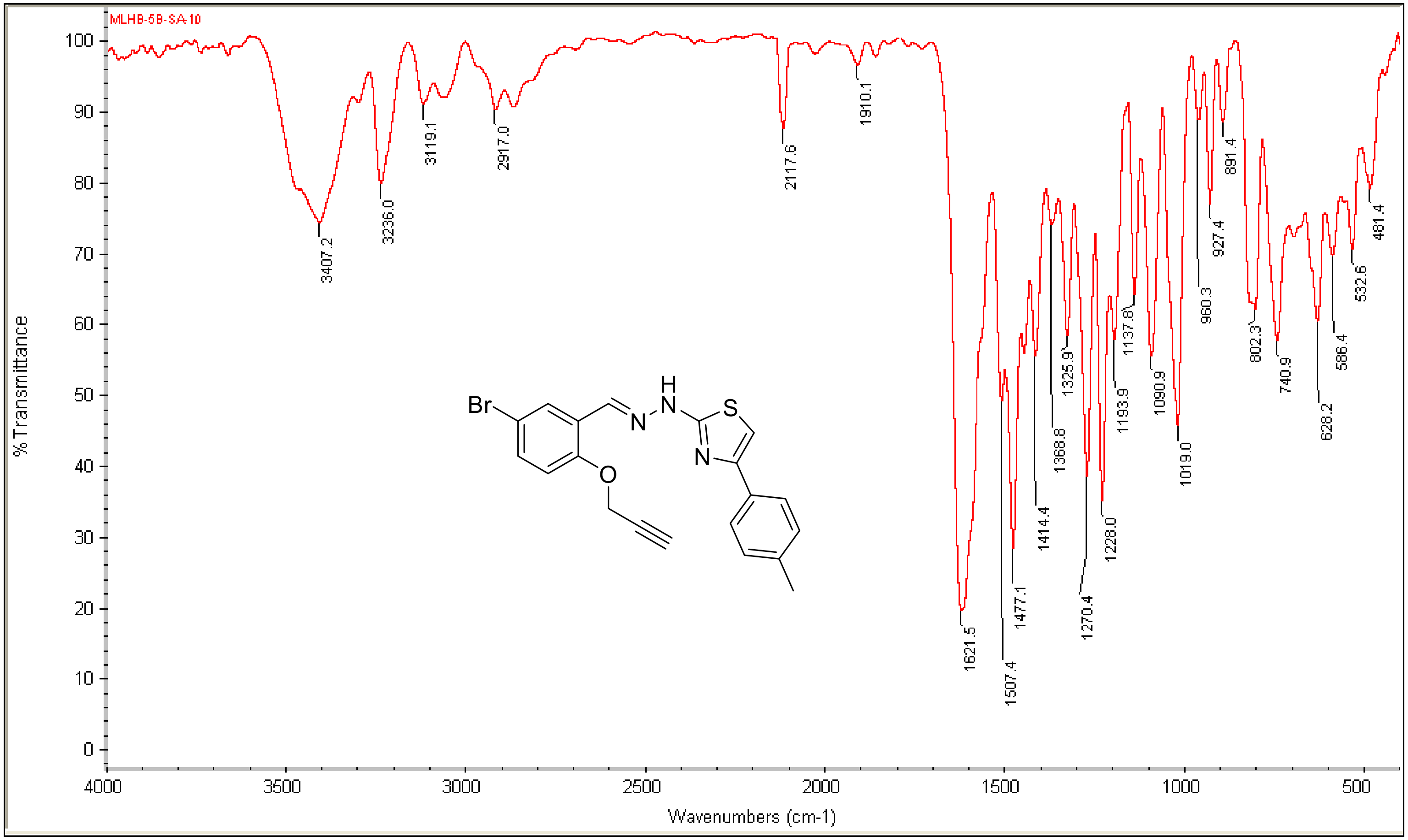

Mass spectrum of (*E*)-2-(2-(5-Bromo-2-(prop-2-yn-1-yloxy)benzylidene)hydrazinyl)-4-(4-methoxyphenyl)thiazole (32)

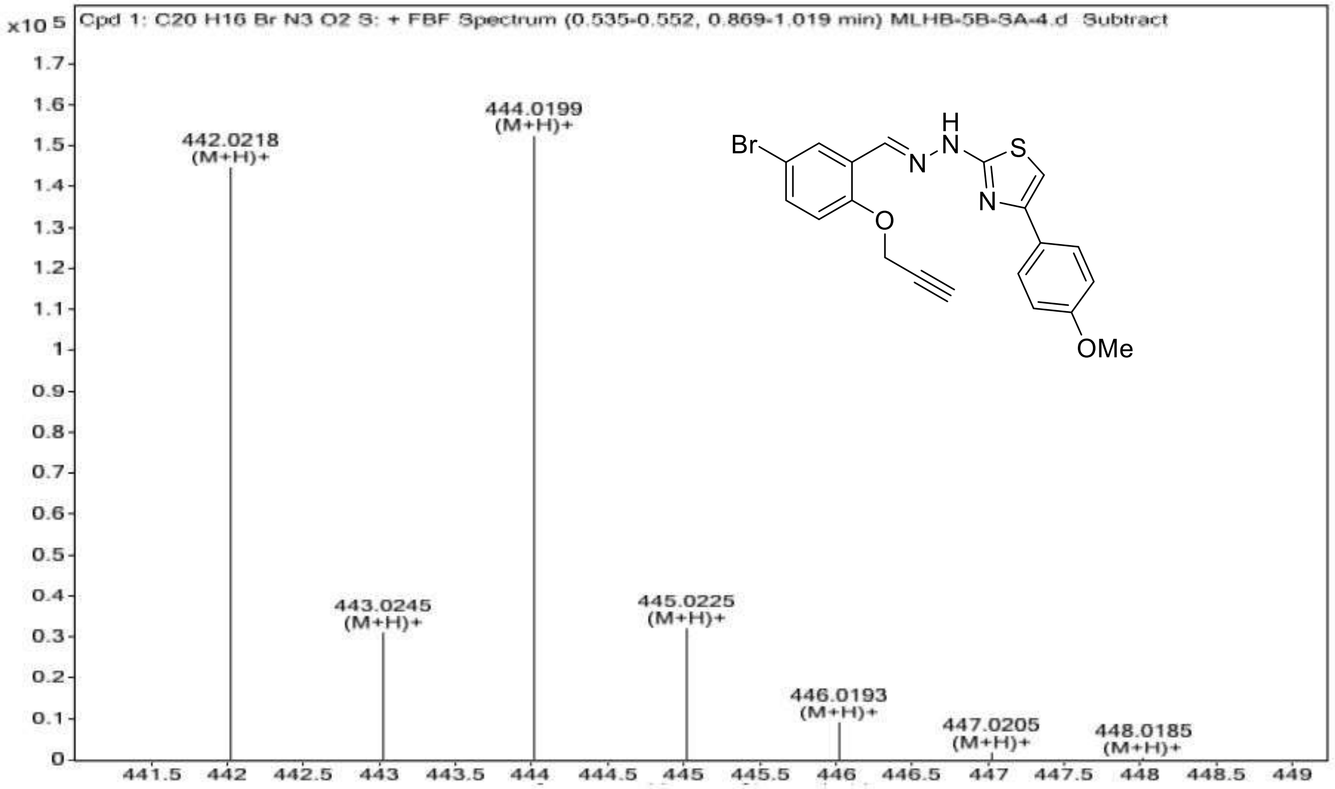

^{1}H NMR spectrum of (*E*)-2-(2-(5-Bromo-2-(prop-2-yn-1-yloxy)benzylidene)hydrazinyl)-4-(4-methoxyphenyl)thiazole (32)

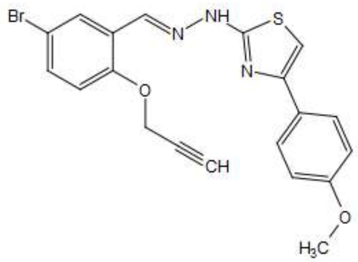

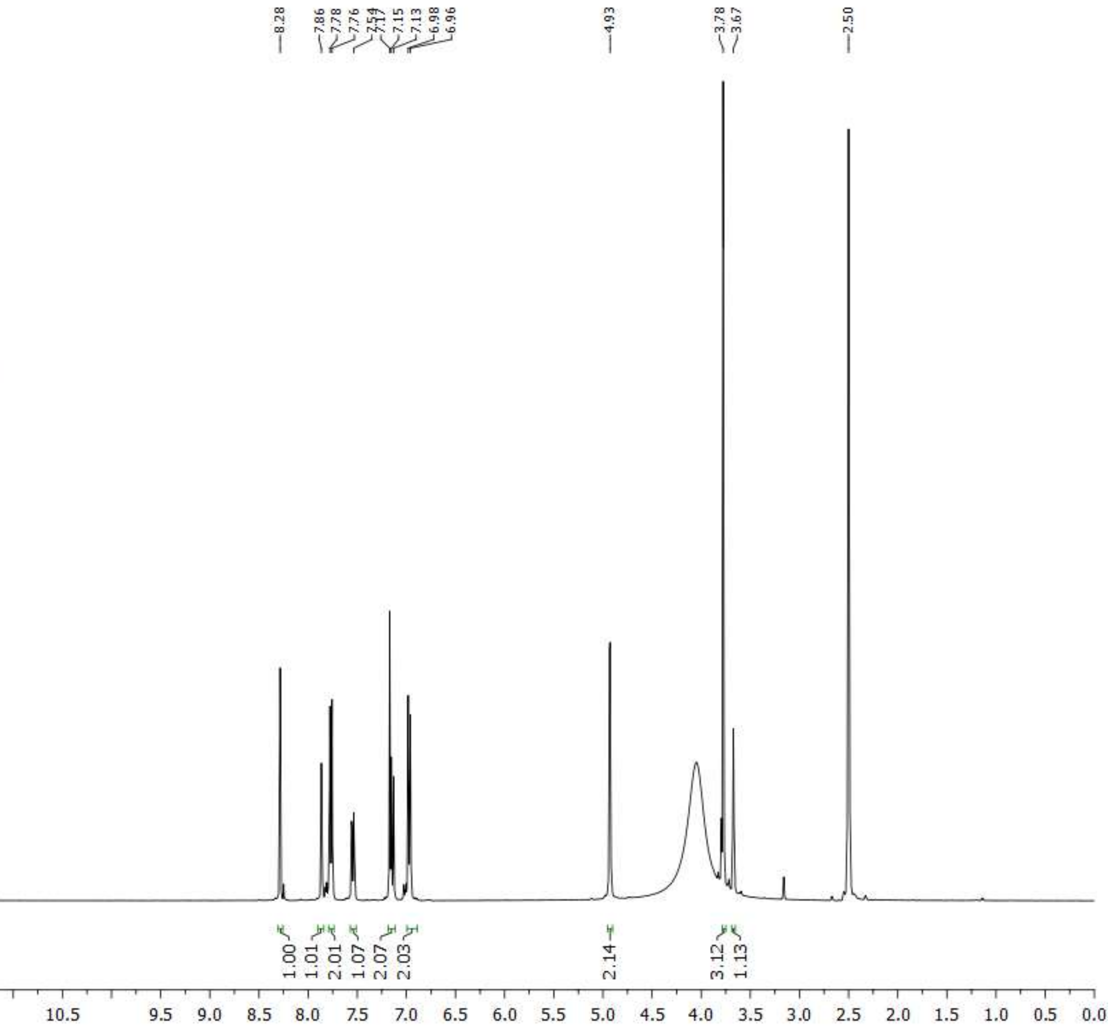

^{13}C NMR spectrum of (*E*)-2-(2-(5-Bromo-2-(prop-2-yn-1-yloxy)benzylidene)hydrazinyl)-4-(4-methoxyphenyl)thiazole (32)

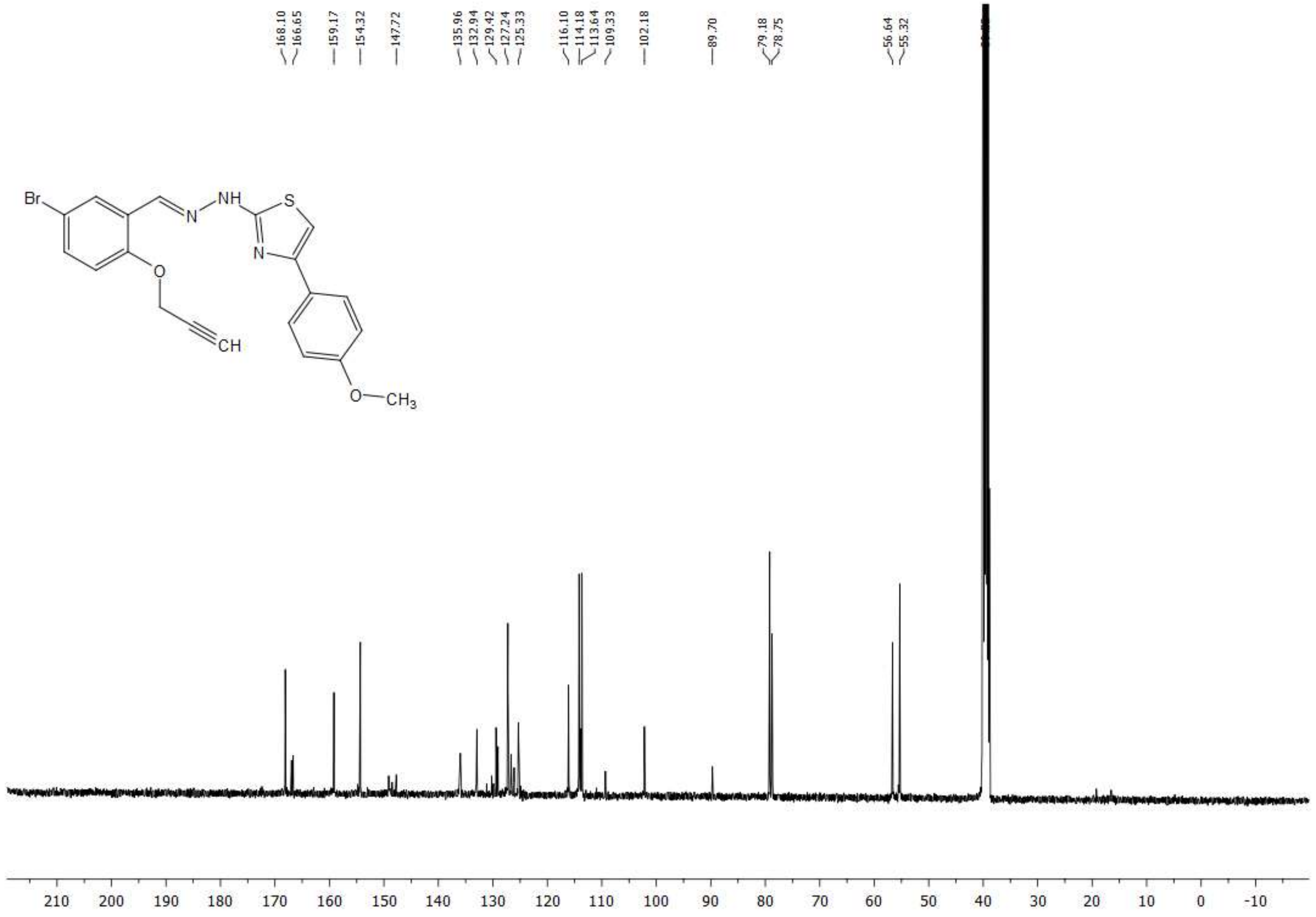

FT-IR spectrum of (*E*)-2-(2-(5-Bromo-2-(prop-2-yn-1-yloxy)benzylidene)hydrazinyl)-4-(4-methoxyphenyl)thiazole (32)

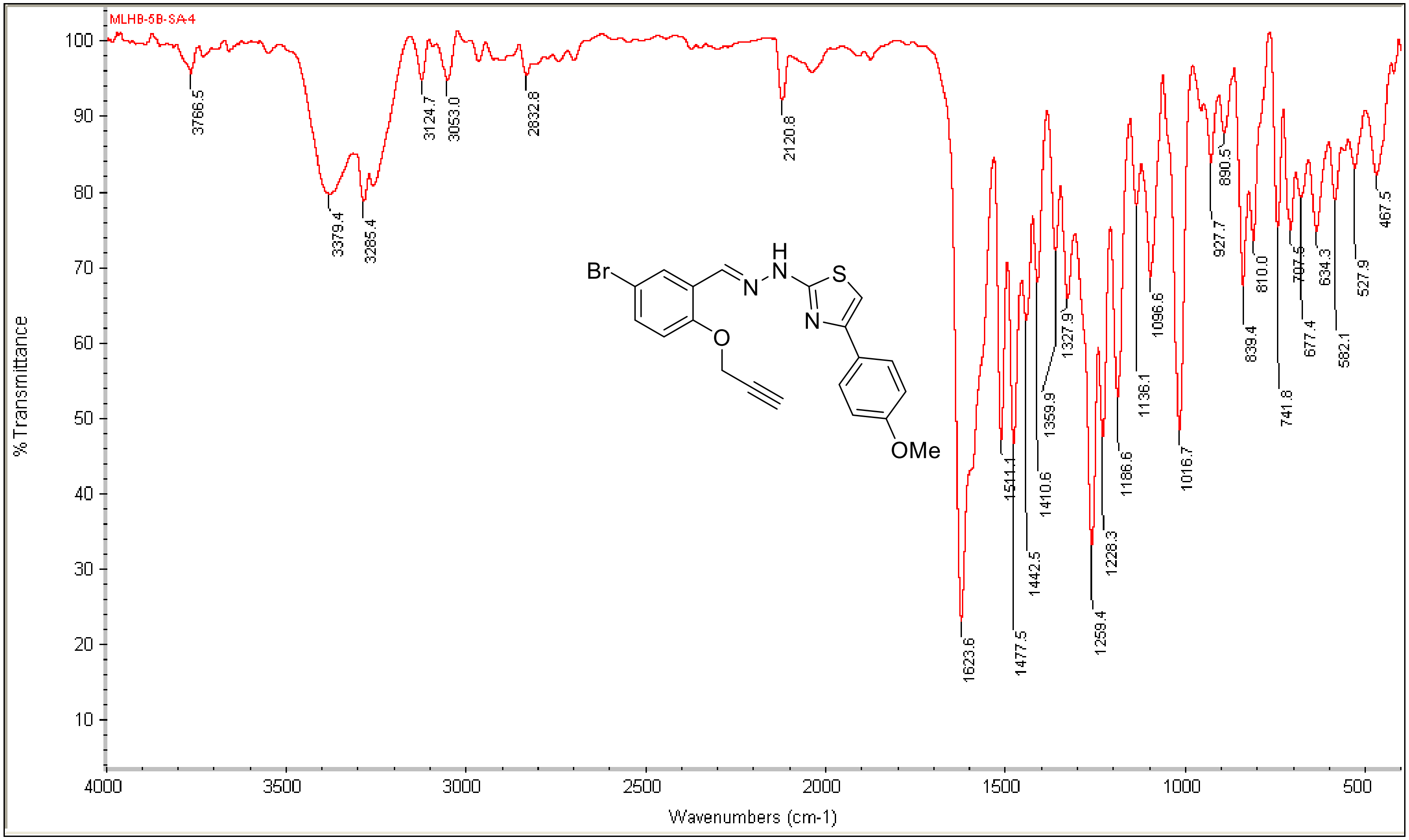

Table S1. Library of acetylene containing 2-(2-hydrazinyl)thiazole derivatives used in the present investigation

Category 1: Library of acetylene containing aldehyde derivatives (1-3)

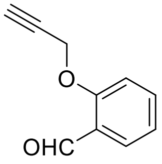

1

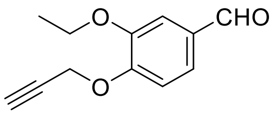

2

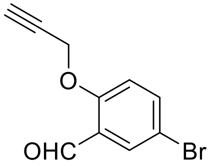

3

Category 2: Library of acetylene containing thiosemicarbazone derivatives (4-6)

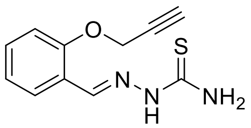

4

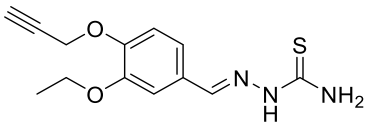

5

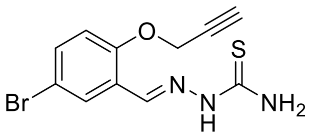

6

Category 3: Library of acetylene containing 2-(2-hydrazinyl)thiazole derivatives (7-32)

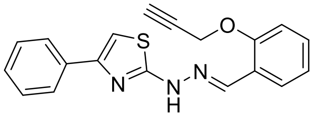

7

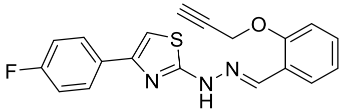

8

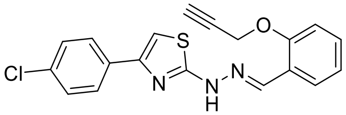

9

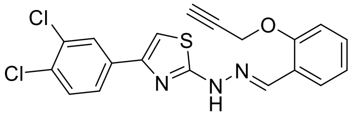

10

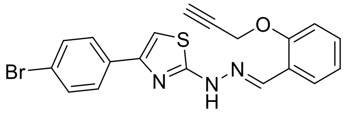

11

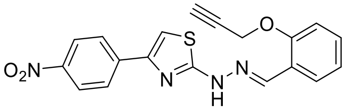

12

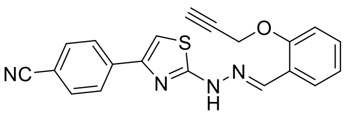

13

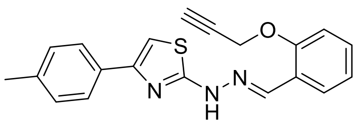

14

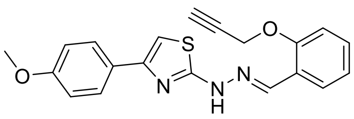

15

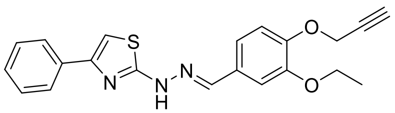

16

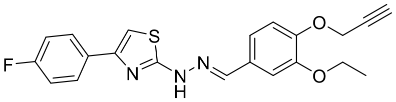

17

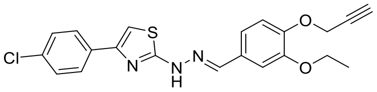

18

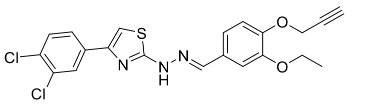

19

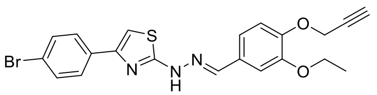

20

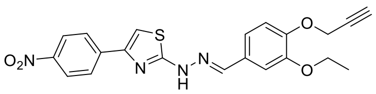

21

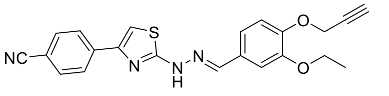

22

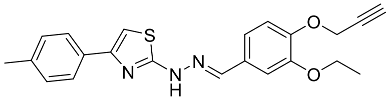

23

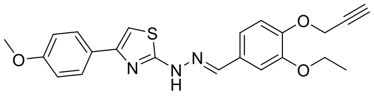

24

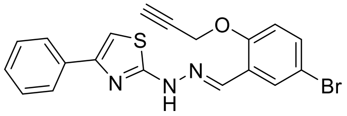

25

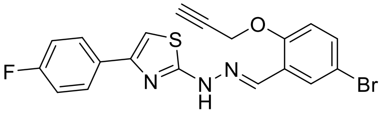

26

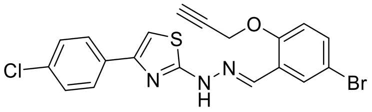

27

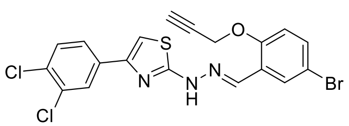

28

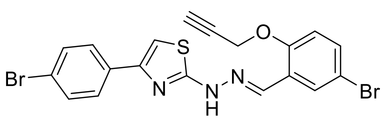

29

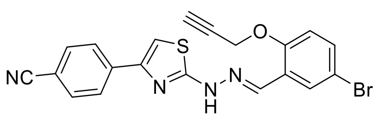

30

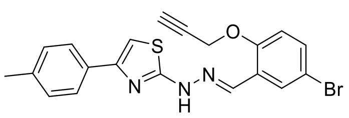

31

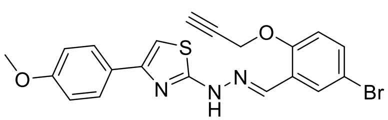

32

Table S2. Pharmacokinetic analysis and *in vitro* mycobacterial analysis results of acetylene containing 2-(2-hydrazinyl)thiazole derivatives.

**Code.**	**Lipinski's Rule of 5**					**Veber Rule**		**% Inhibition ± SD**	
	**Log P**	**MW**	**H-bond donors**	**H-bond acceptors A**	**Total no. of violations**	**TPSA (Å²)**	**Number of rotatable bonds**	**100 μg/ml**	**50 μg/ml**
**1.**	1.90	160.17	0	2	0	26.30	3	-	39.29 ± 1.17
**2.**	1.91	204.23	0	3	0	35.54	5	8.08 ± 0.24	7.73 ± 0.23
**3.**	2.68	239.07	0	2	0	26.30	3	50.85 ± 1.52	48.71 ± 1.46
**4.**	2.05	233.29	3	4	0	59.65	5	-	46.75 ± 1.40
**5.**	2.06	277.34	3	5	0	68.88	7	77.75 ± 2.33	74.58 ± 2.23
**6.**	2.84	312.19	3	4	0	59.65	5	56.86 ± 1.70	51.21 ± 1.53
**7.**	4.13	333.41	1	4	0	46.52	6	-	51.66 ± 1.54
**8.**	4.29	351.40	1	4	0	46.52	6	-	38.01 ± 1.14
**9.**	4.80	367.85	1	4	0	46.52	6	-	50.81 ± 1.52
**10.**	5.41	402.29	1	4	1	46.52	6	-	52.00 ± 1.56

**11.**	4.93	412.31	1	4	0	46.52	6	-	49.13 ± 1.47
**12.**	4.08	378.41	1	7	0	92.34	7	-	29.06 ± 0.87
**13.**	3.88	358.42	1	5	0	70.31	6	-	54.33 ± 1.62
**14.**	4.57	347.44	1	4	0	46.52	6	-	38.18 ± 1.14
**15.**	4.18	363.44	1	5	0	55.75	7	-	10.26 ± 0.30
**16.**	4.14	377.46	1	5	0	55.75	8	48.71 ± 1.46	40.49 ± 1.21
**17.**	4.30	395.45	1	5	0	55.75	8	11.41 ± 0.34	2.06 ± 0.06
**18.**	4.82	411.90	1	5	0	55.75	8	17.79 ± 0.53	6.36 ± 0.19
**19.**	5.42	446.35	1	5	1	55.75	8	7.21 ± 0.21	2.38 ± 0.07
**20.**	4.95	456.36	1	5	0	55.75	8	12.13 ± 0.36	7.35 ± 0.22
**21.**	4.10	422.46	1	8	0	101.58	9	50.85 ± 1.52	48.74 ± 1.46
**22.**	3.89	402.47	1	6	0	79.54	8	57.18 ± 1.71	50.86 ± 1.52
**23.**	4.59	391.49	1	5	0	55.75	8	10.49 ± 0.31	8.72 ± 0.26
**24.**	4.20	407.49	1	6	0	64.98	9	32.98 ± 0.98	5.64 ± 0.16
**25.**	4.91	412.31	1	4	0	46.52	6	66.22 ± 1.98	61.86 ± 1.85
**26.**	5.07	430.30	1	4	1	46.52	6	57.7 ± 1.73	50.7 ± 1.52

**27.**	5.59	446.75	1	4	1	46.52	6	52.69 ± 1.58	38.15 ± 1.14
**28.**	6.20	481.19	1	4	1	46.52	6	50.16 ± 1.50	43.8 ± 1.31
**29.**	5.72	491.20	1	4	1	46.52	6	37.24 ± 1.11	27.05 ± 0.81
**30.**	4.67	437.32	1	5	0	70.31	6	48.69 ± 1.46	43.73 ± 1.31
**31.**	5.36	426.33	1	4	1	46.52	6	56.32 ± 1.68	54.86 ± 1.64
**32.**	4.97	442.33	1	5	0	55.75	7	48.68 ± 1.46	43.69 ±1.31
**INH**	-0.96	137.14	3	4	0	68.01	1	> 99% *	
**RIF**	2.62	822.95	6	16	3	220.16	5	> 99% *	

Note: Pharmacokinetic analyses are obtained from the online server Molinspiration (http://www.molinspiration.com). * at 2 µg/ml.

Table S3. ADME properties of acetylene containing 2-(2-hydrazinyl)thiazole derivatives.

Code	Absorption			Distribution				Metabolism	Excretion
	Log S (log mol/L)	Caco-2 perm. (log Papp in 10^{-6} cm/s)	Int. absorption. (% absorbed)	VDss (log L/kg)	Fraction. Unbound (Fu)	BBB perm (log BB)	BBB predicted		Total clearance (logml/min/kg)
**1.**	-1.645	1.65	97.94	0.042	0.341	0.159	Yes	CYP1A2 inhibitor	0.274
**2.**	-2.604	1.3	99.692	0.088	0.337	0.03	Yes	CYP1A2 inhibitor	0.402
**3.**	-2.56	1.545	96.684	0.04	0.354	0.247	Yes	CYP1A2 inhibitor	0.234
**4.**	-2.579	1.216	87.469	-0.116	0.377	-0.22	No	CYP1A2 inhibitor	-0.021
**5.**	-2.418	0.719	73.221	-0.462	0.383	-0.438	No	-	-0.048
**6.**	-2.852	1.268	93.642	-0.217	0.357	-0.133	No	CYP1A2 inhibitor	-0.108
**7.**	-4.788	1.439	92.889	0.211	0.002	0.268	Yes	CYP3A4, substrate. CYP1A2, CYP2C19, CYP2C9, CYP3A4 inhibitor	0.376
**8.**	-4.872	1.332	92.508	0.212	0.046	0.093	No	CYP3A4 substrate. CYP1A2, CYP2C19, CYP2C9, CYP3A4 inhibitor	0.113
**9.**	-5.334	1.291	91.44	0.265	0.002	0.219	No	CYP3A4 substrate. CYP1A2, CYP2C19, CYP2C9, CYP3A4 inhibitor	0.244
**10.**	-5.912	1.28	91.164	0.361	0	0.196	No	CYP3A4 substrate. CYP1A2, CYP2C19, CYP2C9, CYP3A4	0.374

								inhibitor	
**11.**	-5.422	1.288	91.373	0.285	0	0.218	No	CYP3A4 substrate. CYP1A2, CYP2C19, CYP2C9, CYP3A4 inhibitor	0.223
**12.**	-5.411	0.529	91.021	0.102	0	-0.794	No	CYP3A4 substrate. CYP1A2, CYP2C19, CYP2C9, CYP3A4 inhibitor	0.28
**13.**	-4.896	0.672	93.917	0.19	0	-0.447	No	CYP3A4 substrate. CYP1A2, CYP2C19, CYP2C9, CYP3A4 inhibitor	0.342
**14.**	-5.051	1.293	92.898	0.296	0.014	0.232	No	CYP3A4 substrate. CYP1A2, CYP2C19, CYP2C9, CYP3A4 inhibitor	0.318
**15.**	-4.905	1.291	93.917	0.303	0.014	-0.03	No	CYP3A4 substrate. CYP1A2, CYP2C19, CYP2C9, CYP3A4 inhibitor	0.294
**16.**	-5.325	1.086	93.591	0.35	0.037	0.2	No	CYP3A4 substrate. CYP1A2, CYP2C19, CYP2C9, CYP3A4 inhibitor	0.345
**17.**	-5.216	1.118	93.191	0.357	0.095	0.025	No	CYP3A4 substrate. CYP1A2, CYP2C19, CYP2C9, CYP3A4 inhibitor	0.082
**18.**	-5.772	1.077	92.095	0.405	0.04	0.152	No	CYP3A4 substrate. CYP1A2, CYP2C19, CYP2C9, CYP3A4 inhibitor	0.213
**19.**	-6.193	1.068	91.819	0.498	0.027	0.142	No	CYP3A4 substrate. CYP1A2, CYP2C19, CYP2C9, CYP3A4	0.343

								inhibitor	
**20.**	-5.844	1.074	92.028	0.426	0.037	0.15	No	CYP3A4 substrate. CYP1A2, CYP2C19, CYP2C9, CYP3A4 inhibitor	0.192
**21.**	-6.039	0.766	93.403	0.18	0	-0.83	No	CYP3A4 substrate. CYP2C19, CYP2C9, CYP3A4 inhibitor	0.249
**22.**	-5.386	1.151	94.572	0.33	0.034	-0.501	No	CYP3A4 substrate. CYP1A2, CYP2C19, CYP2C9, CYP3A4 inhibitor	0.312
**23.**	-5.538	1.079	93.554	0.433	0.05	0.165	No	CYP3A4 substrate. CYP1A2, CYP2C19, CYP2C9, CYP3A4 inhibitor	0.287
**24.**	-5.221	1.092	94.599	0.444	0.057	-0.097	No	CYP3A4 substrate. CYP1A2, CYP2C19, CYP2C9, CYP3A4 inhibitor	0.263
**25.**	-5.4	1.284	91.396	0.273	0.006	0.221	No	CYP3A4 substrate. CYP1A2, CYP2C19, CYP2C9, CYP3A4 inhibitor	0.283
**26**	-5.373	1.316	91.015	0.262	0.058	0.046	No	CYP3A4 substrate. CYP1A2, CYP2C19, CYP2C9, CYP3A4 inhibitor	0.021
**27**	-5.941	1.275	89.947	0.324	0.007	0.173	No	CYP3A4 substrate. CYP1A2, CYP2C19, CYP2C9, CYP3A4 inhibitor	0.152
**28**	-6.495	1.264	89.67	0.413	0	0.15	No	CYP3A4 substrate. CYP1A2, CYP2C19, CYP2C9, CYP3A4 inhibitor	0.282

**29**	-6.023	1.273	89.88	0.344	0.003	0.171	No	CYP3A4 substrate. CYP1A2, CYP2C19, CYP2C9, CYP3A4 inhibitor	0.13
**30**	-5.527	0.683	92.424	0.24	0.005	-0.645	No	CYP3A4 substrate. CYP1A2, CYP2C19, CYP2C9, CYP3A4 inhibitor	0.25
**31**	-5.674	1.277	91.405	0.356	0.019	0.186	No	CYP3A4 substrate. CYP1A2, CYP2C19, CYP2C9, CYP3A4 inhibitor	0.226
**32**	-5.405	1.276	92.423	0.347	0.024	-0.077	No	CYP3A4 substrate. CYP1A2, CYP2C19, CYP2C9, CYP3A4 inhibitor	0.201
**INH**	-2.024	0.695	96.452	0.053	0.776	-0.002	No	-	0.703
**RIF**	-2.914	-0.219	41.095	1.49	0.209	-2.577	No	-	-0.624

Note: ADME properties were obtained from online servers pkCSM (http://biosig.unimelb.edu.au/pkcsm/) and SwissADME (http://www.swissadme.ch/).

Table S4. Single-crystal XRD data.

	**Compound 4**	**Compound 7**
**CCDC Number**	2038796	2038797
**Empirical formula**	$C_{22}H_{22}N_6O_2S_2$	$C_{18}H_{14}N_4OS$
**Formula weight**	466.58	334.39
**Temperature/K**	298	298
**Crystal system**	triclinic	monoclinic
**Space group**	P-1	P2/n
**a/Å**	8.6009(5)	10.6038(7)
**b/Å**	10.6365(7)	9.9316(7)
**c/Å**	13.6233(9)	16.1177(11)
**α/°**	95.201(5)	90
**β/°**	95.934(5)	93.716(7)
**γ/°**	107.575(5)	90
**Volume/Å³**	1171.95(12)	1693.8(2)
**Z**	2	4

**ρ_{calc}g/cm^3**	1.322	1.311
**μ/mm^{-1}**	0.258	0.203
**F(000)**	488.0	696.0
**Crystal size/mm^3**	0.4 × 0.38 × 0.24	0.64 × 0.58 × 0.26
**Radiation**	MoKα (λ = 0.71073)	MoKα (λ = 0.71073)
**2Θ range for data collection/°**	6.24 to 58.88	6.52 to 58.72
**Index ranges**	-11 ≤ h ≤ 11, -13 ≤ k ≤ 14, -18 ≤ l ≤ 18	-13 ≤ h ≤ 11, -13 ≤ k ≤ 12, -21 ≤ l ≤ 20
**Reflections collected**	12789	9712
**Independent reflections**	5520 [R_{int} = 0.0284, R_{sigma} = 0.0509]	3990 [Rint = 0.0198, Rsigma = 0.0242]
**Goodness-of-fit on F^2**	0.981	1.033

Table S5. Summary of active compounds % inhibition at 50 µg/ml.

Structure of thiosemicarbazone	Structure of acetylene containing 2-(2-hydrazinyl)thiazoles
	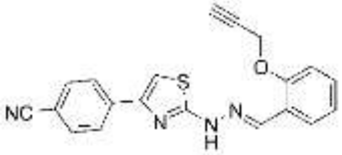<br>**13**<br>54%<br>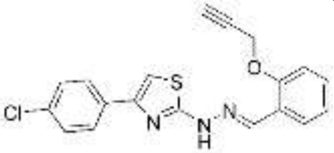<br>**9**<br>50%<br>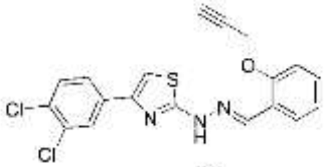<br>**10**<br>52%<br>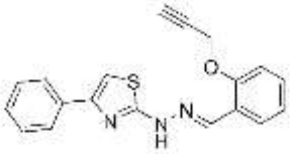<br>**7**<br>51%

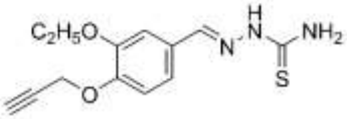<br>**5**<br>74%	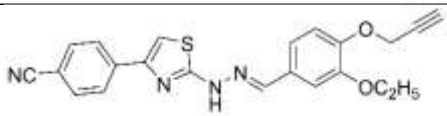<br>**22**<br>50%
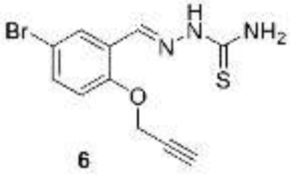<br>**6**<br>51%	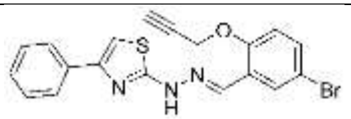<br>**25**<br>61%<br>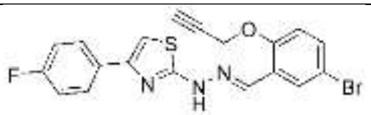<br>**26**<br>50%<br>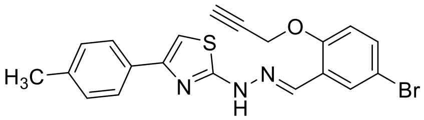<br>**31**<br>54%

Table S6. ***In silico*** **studies of active compounds with KasA protein.**

**Code**	***In silico*** **studies with KasA protein**		
	**Binding Affinity (kcal/mol)**	**Binding const. (Ki) (μM)**	**Interacting amino acids**
**1**	-6.1	33.39686	GLY403, GLY318, VAL278, PRO280, ALA287, ALA321, HIS311, PHE402, PHE404
**2**	-5.9	46.82409	THR315, ILE317, ALA279, MET213, ALA215
**3**	-5.0	214.2428	ALA215, ILE317, PRO280, MET213, VAL278, PHE404, ALA279
**4**	-5.6	77.73472	MET213, ILE317, ALA279, ILE317, MET213, ALA215
**5**	-5.2	152.8067	THR315, ILE317, HIS311, ALA215, ALA279
**6**	-5.5	92.0443	MET213, ARG214, ILE317, PRO280, PHE404, ALA215, ALA279
**7**	-6.4	20.11685	GLY39, GLU40, LEU371
**8**	-6.6	14.34816	MET213, ILE317, ALA215, ALA279
**9**	-6.5	16.9894	GLY39, GLU40, HIS44, LEU371

**10**	-7.7	2.236638	GLU374, ASN372, GLU224, GLY39, GLU40, PRO369, LEU371
**11**	-6.7	12.11754	MET213, VAL278, ILE317, MET277, ALA215, ALA279
**12**	-6.4	20.11685	MET213, ILE317, PHE404, ALA215, ALA279
**13**	-6.9<br><br>-6.1	8.642724<br><br>33.39686	TYR373, ALA38, GLY39, GLU40, LEU371<br><br>TYR373, GLY39, LEU371
**14**	-6.9	8.642724	MET213, ARG214, ILE317, ALA279, PRO280, PHE404
**15**	-6.8<br><br>-6.4	10.2337<br><br>20.11685	GLY387, GLY39, TYR373, GLU40, ASN372, LEU371<br><br>GLY387, GLY39, ASN372, GLU224, ARG225, ALA38, LEU371
**16**	-6.5	16.9894	GLU40, GLU224, GLY39, LEU371
**17**	-6.8	10.2337	GLU40, ASN372, GLY39, TYR373, GLU374, LEU371
**18**	-6.5	16.9894	GLY39, GLU40, LEU371
**19**	-7.2	5.20601	MET213, ARG214, MET212, HIS311, PHE404, ALA215, ALA279, ILE317
**20**	-6.9	8.642724	TYR373, GLY39, GLU40, LEU371, HIS44, ALA386
**21**	-6.7	12.11754	MET213, ARG214, ILE317, HIS311, PHE404, ALA215, ALA279
**22**	-6.9	8.642724	GLU40, PRO27
**23**	-6.6	14.34816	GLY39, SER41, ASN372, LEU371

**24**	-6.7	12.11754	VAL19, SER97, ASP28, LYS260, LEU262
**25**	-6.8	10.2337	ASN372, GLU40, LEU371, PRO369
**26**	-6.9	8.642724	GLU224, GLU224, GLY387, GLU40, LEU371, ARG225
**27**	-6.5	16.9894	GLU40, LEU371, PRO369
**28**	-7.0	7.299091	ASN372, GLU40, LEU371, PRO369
**29**	-6.6	14.34816	GLU40, LEU371
**30**	-6.9	8.642724	TYR373, GLU40, LEU371
**31**	-6.7	12.11754	GLU40, LEU371, PRO369
**32**	-6.9	8.642724	GLU40, LEU371, ASN372
**INH**	-6.1	33.39686	VAL 278, GLY 403, HIS 311, PRO 280
**RIF**	-7.8	1.888921	GLU 224, LEU 371

Table S7. Summary of the analysis of variance (ANOVA) data at 50 µg/ml.

**SUMMARY**						
Groups	***Count***	***Sum***	***Average***	***Variance***		
**Trial 1**	32	1178.15	36.81719	396.4437		
**Trial 2**	32	1145.17	35.78656	382.4966		
**Trail 3**	32	1206.55	37.70469	413.8735		
**ANOVA**						
Source of Variation	***SS***	***df***	***MS***	***F***	***P-value***	***F crit***
**Between Groups**	58.97651	2	29.48825	0.074165	0.928574	3.094337
**Within Groups**	36977.23	93	397.6046			
**Total**	37036.2	95				

Table S8. Summary of the analysis of variance (ANOVA) data at 100 µg/ml.

**SUMMARY**						
Groups	***Count***	***Sum***	***Average***	***Variance***		
**Trail 1**	21	859.99	40.9519	454.4107		
**Trail 2**	21	877.49	41.78524	465.459		
**Trail 3**	21	843.35	40.15952	437.3954		
**ANOVA**						
Source of Variation	***SS***	***df***	***MS***	***F***	***P-value***	***F crit***
**Between Groups**	27.75681	2	13.87841	0.030676	0.969805	3.150411
**Within Groups**	27145.3	60	452.4217			
**Total**	27173.06	62				

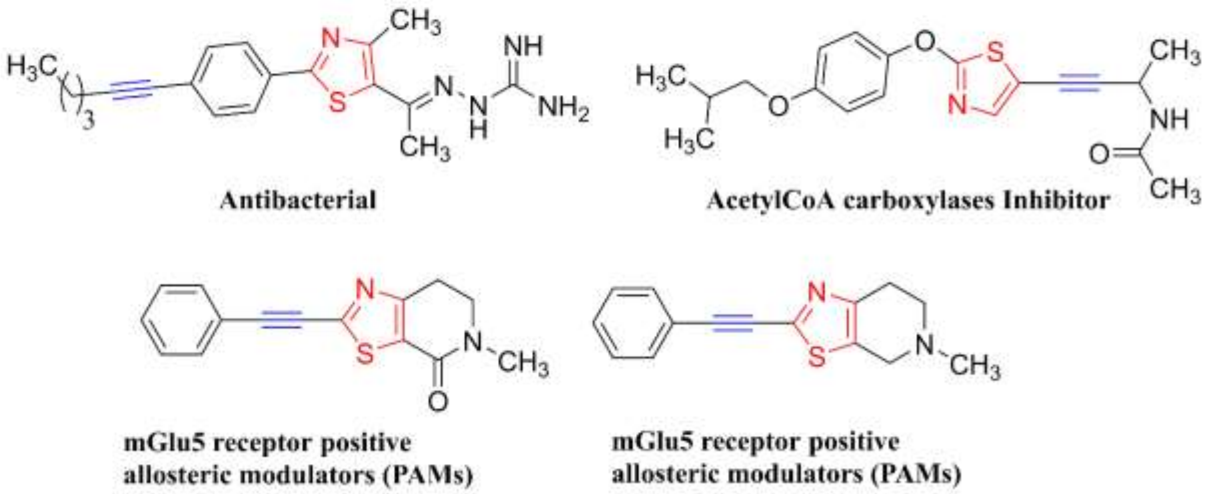

Fig. S1. Reported biological compounds containing thiazole and acetylene pharmacophores.
